# A Complex Heterogeneous Network Model of Disease Regulated by Noncoding RNAs: A Case Study of Unstable Angina Pectoris

**DOI:** 10.1155/2022/5852089

**Published:** 2022-12-23

**Authors:** Guanpeng Qi, Ze Xu, Hanyu Dan, Xiangnan Jia, Qiang Jiang, Aijun Zhang, Zhaohang Li, Xin Liu, Juman Ma, Xiaosong Zheng, Zuojing Li

**Affiliations:** ^1^School of Pharmacy, Shenyang Pharmaceutical University, Shenyang 110016, China; ^2^School of Medical Devices, Shenyang Pharmaceutical University, Shenyang 110016, China; ^3^School of Life Sciences and Biopharmaceuticals, Shenyang Pharmaceutical University, Shenyang 110016, China

## Abstract

MicroRNAs (miRNAs) are important types of noncoding RNAs, and there is a lack of holistic and systematic understanding of the functions they play in disease. We proposed a research strategy, including two parts network analysis and network modelling, to analyze, model, and predict the regulatory network of miRNAs from a network perspective, using unstable angina pectoris as an example. In the network analysis section, we proposed the WGCNA & SimCluster method using both correlation and similarity to find hub miRNAs, and validation on two datasets showed better results than the methods using correlation or similarity alone. In the network modelling section, we used six knowledge graph or graph neural network models for link prediction of three types of edges and multilabel classification of two types of nodes. Comparative experiments showed that the RotatE model was a good model for link prediction, while the RGCN model was the best model for multilabel classification. Potential target genes were predicted for hub miRNAs and validation of hub miRNA-target gene interactions, target genes as biomarkers and target gene functions were performed using a three-step validation approach. In conclusion, our study provides a new strategy to analyze and model miRNA regulatory networks.

## 1. Introduction

Noncoding RNAs play an important role in the development of complex diseases, and their functions can be elucidated to help us understand the complex processes of disease and develop appropriate drugs [1]. MicroRNAs (miRNAs) are important types of noncoding RNAs that are key regulators of a variety of biological pathways, both in disease and normal states of the body [2]. They mostly play a negative regulatory role in promoting the degradation of mRNAs or inhibiting translation [3]. An increasing number of studies have reported the role of miRNA-mRNA regulatory networks in disease development [4, 5], suggesting that miRNAs may systematically perform gene regulation through a number of regulatory networks.

Unstable angina (UA) is one of the acute coronary syndromes in which the frequency and duration of attacks are unstable and may lead to myocardial infarction in severe cases [6]. Unstable angina is a complex cardiovascular disease associated with multiple causative factors [7]. Current studies suggest that the disease is caused by myocardial ischemia and hypoxia following the formation of a thrombus in the coronary arteries [8], but the exact etiology and pathogenesis remain to be further elucidated. Many studies have shown that some miRNAs and some of the genes they regulate may be diagnostic markers or therapeutic targets for unstable angina [9, 10]. Exploring the role played by miRNAs is an effective means of understanding the mechanisms of the disease and developing relevant drugs.

WGCNA (weighted gene coexpression network analysis) allows the analysis of experimentally measured genes or RNA expression data [11]. After calculating the correlation of genes or RNAs using expression data, a weighted correlation matrix and a topological overlap matrix can be constructed, and then, hierarchical clustering can be performed to form modules. We can focus on key modules and key nodes, as they can not only act as markers or therapeutic targets but also perform essential functions and influence many other biomolecules, which can be useful in understanding the complex mechanisms of disease [12]. However, the WGCNA method only considers correlations between genes or RNAs, which is not sufficient, as there are not only correlations but also similarities between molecules [13]. Similarity networks, which consist of similarity relationships between molecules, reveal which molecules have similar mechanisms of action [14] in contrast to the coexpression networks constructed by the WGCNA approach. In a similarity network, the existence of edges between nodes indicates that nodes have similar mechanisms of action, and a hub node with numerous neighbor nodes contains most of the mechanisms of action of its neighbors [15]. Therefore, hub nodes in similarity networks often perform multiple functions, so interfering with or disrupting these core nodes is likely to affect multiple functions. Similar to the key modules and key nodes in the WGCNA approach, the key nodes in these similarity networks are also likely to be markers and therapeutic targets and contribute to understanding the mechanisms of disease [16, 17]. Constructing similarity networks and finding key nodes provide an alternative way of analyzing complex networks [18].

A knowledge graph is a multirelational graph consisting of entities (nodes) and relationships (edges) and is composed of a series of triples (*h*, *r*, and *t*), which is essentially a heterogeneous network graph [19]. A knowledge graph or heterogeneous network graph is a complex network graph containing different types of nodes and edges. After modelling this complex network, it is possible to make predictions about the existence of nodes or edges in the network, as well as to predict the labels of nodes and edges, and also to perform recommendation tasks [20]. Many miRNAs regulate many genes and in turn affect many pathways. This complex process can be graphically represented by the miRNA-target gene-pathway heterogeneous network (MTP), which we refer to as the miRNA regulatory network [21]. On the one hand, the constructed MTP networks can be used to construct miRNA similarity networks and find hub miRNAs by calculating the similarity of miRNA actions, i.e., network analysis. On the other hand, MTP networks can also be modelled with models, such as knowledge graphs or graph neural networks, i.e., network modelling, both for predicting nodes and connected edges as well as labels with classifiers such as fully connected neural networks (MLP) after obtaining embedding representation [22]. Network modelling is a deep learning task for modelling complex heterogeneous network graphs. Both the network analysis part and the network modelling part explore miRNA regulatory networks from a network perspective, and the combination of the two parts provides new ideas to analyze and predict the role of miRNAs in complex diseases in a holistic and systematic way.

**Figure 1:**
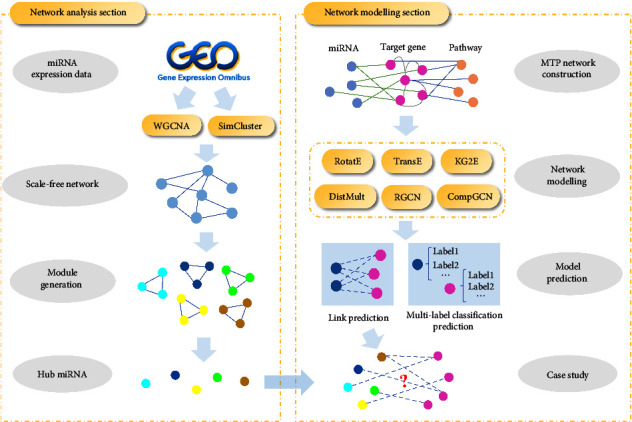
Flowchart of a model for the study of the complex heterogeneous network in unstable angina regulated by miRNAs.

**Figure 2:**
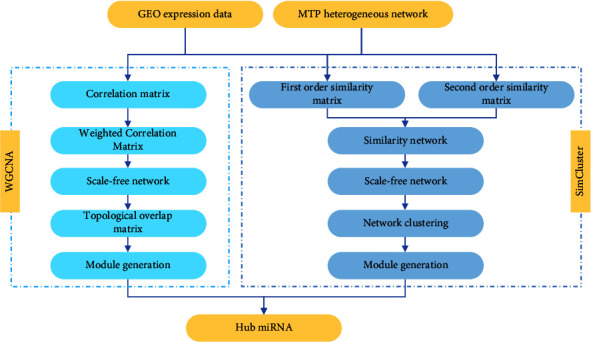
Flowchart of the SimCluster method and the WGCNA method.

At present, the functions of many miRNAs are still not well understood, and the regulatory roles that miRNAs play in complex diseases are not systematically elucidated [23]. To find better hub miRNAs, it is also worth investigating whether correlations and similarities in complex networks can be combined to obtain a better network analysis method than WGCNA [24]. We used miRNAs as a representative of noncoding RNAs and constructed a miRNA regulatory network, the MTP network, using unstable angina as an example. For this regulatory network of noncoding RNAs, we proposed a research method that contains a network analysis part based on multiple network analysis methods and a network modelling part based on knowledge graph algorithms. Hub miRNAs were obtained by an improved network analysis method, while the MTP network was modelled using a knowledge graph algorithm, and interactions in this regulatory network were predicted as a prediction task for edges, while the class of nodes was predicted as a prediction task for node labels [25]. Finally, the potential targets and functions of hub miRNAs were predicted. The overall study flowchart is shown in Figure 1, and the flowchart of the network analysis part is shown in Figure 2. The acronym table for this study is Table S1 in Supplementary Materials.

## 2. Materials and Methods

### 2.1. Data Preparation

#### 2.1.1. miRNAs and Their Target Genes

Expression data for noncoding RNAs analyzed by an array were obtained from the GEO database (GSE94605), specifically miRNA expression data in plasma from healthy subjects and patients with unstable angina, with 7 and 6 sample pools in the control and case groups, respectively [26]. Differential expression analysis by using the GEO2R tool was used to obtain differentially expressed plasma miRNAs, and then, we set |logFC| > 2 and adjusted *p* value <0.05 to screen for significantly differentially expressed miRNAs [27]. The miRecords, miRTarBase, and TarBase databases were used to find experimentally validated miRNA-target gene interactions [28], and the top 10% of target genes were taken as true target genes.

The GeneCards database and the DisGeNET database were used to find genes for unstable angina, and the target genes of miRNAs were intersected with disease genes. The intersecting genes were used as target genes regulated by miRNAs that were differentially expressed in the disease condition [29].

#### 2.1.2. Tissue Localization of Genes and Protein-Protein Interactions

Different genes are expressed differently in different tissues, and gene expression is tissue specific [30]. We used the expression of genes in different tissues to indicate the tissue localization of genes. The TISSUES database was used to find the expression of miRNA target genes in different tissues, with gene expression data for up to 21 tissues [31].

Protein-protein interaction data were retrieved from the String database, keeping default parameter settings [32].

#### 2.1.3. Functional Enrichment and Pathway Categorization

KEGG enrichment analysis of target genes was performed using the clusterProfiler package in R software [33], with *p* values set to less than 0.05. Pathways were categorized in the KEGG database, with the top level containing seven broad categories, and we used the KEGG database to classify pathways.

#### 2.1.4. Datasets for Validation

The dataset for miRNA expression in plasma used to construct the complex network is referred to as the original dataset. To make the results more convincing, two additional independent external datasets were used to validate miRNAs and target genes, respectively, which are referred to as new datasets.

In the new GSE49823 dataset [34], plasma miRNA expression data were recorded for the unstable angina patient group and the control group, with 13 samples for the disease group and 13 samples for the control group, making a total of 26 miRNA expression samples. This miRNA expression dataset was used to test the performance of the network analysis algorithm and the reliability of hub miRNA.

The new GSE60993 dataset [35] contains gene expression data from the peripheral blood of patients with unstable angina and normal controls, with 9 samples from the disease group and 7 samples from the control group. This gene expression dataset was used to test the performance of the network modelling algorithm and the reliability of hub miRNA target genes.

### 2.2. Network Analysis

The WGCNA analysis method is based on the expression correlation between genes or RNAs for network analysis, however, using only correlation networks to find hub miRNAs does not fully utilize the information of complex networks. Similarity is different from correlation, so we proposed a method to calculate the similarity of miRNAs based on MTP heterogeneous networks to construct similarity networks and find hub miRNAs, which is called the SimCluster analysis method. The flowchart of the network analysis part is shown in Figure 2.

#### 2.2.1. WGCNA

We used the WGCNA method to analyze the expression data of differentially expressed miRNAs and find hub miRNAs for unstable angina for subsequent studies [12]. The WGCNA method can transform the coexpression network into a scale-free network by setting the *β* parameter, with fewer nodes of a high degree and more nodes of a low degree [36].

Network analysis of miRNA expression data was performed using the ImageGP website [37] based on the WGCNA method, with parameters set to the signed network and Pearson correlation and *R*-squared set to 0.9. After calculation, the *β* parameter value was finally chosen as 18.

The obtained weighted miRNA coexpression network was hierarchically clustered to classify modules, and the top 10 most important miRNAs in each module were taken as hub miRNAs.

#### 2.2.2. SimCluster

The SimCluster algorithm consists of two main parts, similarity network construction and hub miRNA screening, and the flow of the algorithm consists of the following 3 steps:

1. We used the MTP network described in Section 3.1 to calculate the first-order similarity and second-order similarity of miRNAs, where first-order similarity refers to the similarity of miRNAs at the target gene network level (*M*-*T*), while second-order similarity refers to the similarity of miRNAs at the enriched pathway level (*M*-*P*). In the MTP network, each miRNA has its target set and pathway set, and the Jaccard similarity formula [38] is used to calculate the first-order similarity value and second-order similarity value of any two miRNAs.
2. We defined similarity values as thresholds and obtained different similarity matrices by setting different threshold lower limits, which were then converted into corresponding similarity networks. Drawing on the idea of the WGCNA method to construct a scale-free network [39], we calculated the distribution of degree values and degree frequencies of all miRNAs in the similarity network to determine whether the network was a scale-free network or not. Specifically, we used Pearson's correlation coefficient and *R*^2^ of linear regression to calculate whether the logarithm of degree values (lg (*K*)) and the logarithm of degree frequencies (lg (pK)) were highly correlated and linearly correlated.
3. For scale-free similarity networks, we used several network clustering algorithms to divide modules and selected the optimal network clustering algorithm using modularity values [40]. For each divided module, miRNA with the largest degree value was selected as hub miRNA.

#### 2.2.3. WGCNA & SimCluster

The WGCNA method uses correlation between miRNAs, while the SimCluster method uses similarity between miRNAs. The WGCNA & SimCluster method combines these two methods, containing both correlation and similarity information. Specifically, the results of the WGCNA method and the SimCluster method were intersected to obtain final hub miRNAs.

We compared the performance of hub miRNAs obtained by the WGCNA method, the SimCluster method, and the WGCNA & SimCluster method on original and new datasets, respectively, to evaluate their potential as biomarkers, using the AUC (area under the ROC curve) and AUPR (area under the PR curve) as metrics. A comparison of the above three network analysis methods is shown in Table 1, including data, methods, and some results.

### 2.3. Network Modelling

We used the data from the data preparation section to construct a miRNA-target gene-pathway heterogeneous network (MTP) containing three types of nodes and edges: *M*, *T*, and *P* and *M*-*T*, *T*-*T*, and *T*-*P*. Next, we modelled this complex heterogeneous network regulated by miRNAs using a series of knowledge graph models (including graph neural network models).

#### 2.3.1. Models for Knowledge Graphs

A knowledge graph is a set of many triples (*h*, *r*, and *t*) [41], each (*h*, *r*, and *t*) representing the head entity *h*, the tail entity *t*, and the relationship between them *r*. Knowledge graph models are advantageous in dealing with complex heterogeneous graphs consisting of different types of nodes and edges [42], and a number of models have been successively published.

We have modelled the constructed MTP network using a series of knowledge graph models or graph neural network models that have been published in recent years. After much experimentation, we selected the RotatE model [43] for the link prediction task and the RGCN model [44] for the multilabel classification task.

(1) The RotatE model maps entities and relationships to a complex vector space and defines each relationhip as a rotation between the head entity and the tail entity. It allows the modelling and inference of relationships such as symmetry, antisymmetry, inversion, and composition, which are difficult to accomplish with other models [43]. The core formulations of the RotatE model are shown as follows:
  (1) $$\begin{array}{c} \text{,} \end{array}$$
  (2) $$\begin{array}{c} \text{,} \end{array}$$
  (3) $$\begin{array}{c} \text{.} \end{array}$$
In the above four formulas, **h**, **r**, and **t** are the embedding representations of the head entity, relationship, and tail entity, respectively. The Hadamard product is denoted by the symbol ○, and **d**_**r**_(**h**, **t**) is the distance calculated for each triple. Once the distance values are obtained, they can be optimized using Equation (2). **d**_**r**_(**h**, **t**) is the distance value for positive samples, while **d**_**r**_(**h**_**i**_′, **t**_**i**_′) is the distance value for negative samples. **p**(**h**_**i**_′, **r**, **t**_**i**_′) represents a new negative sampling method proposed by the authors of the model, where they designed a distribution function and sampled negative triples, calculating a probability value to be the weight value of the negative sample. Equation (3) is the scoring function for triples, with a higher score indicating a more realistic triple.
(2) The RGCN model (relational graph convolutional network) is a graph neural network model for heterogeneous graphs. By following the idea of message passing network calculation [45], the formulas are shown as follows:
  (4) $$\begin{array}{c} \text{,} \end{array}$$
  (5) $$\begin{array}{c} f\left(h,r,t\right)=h^{L}\;{Rt}^{L}\text{.} \end{array}$$
**N**_**i**_^**r**^ denotes the neighboring nodes of **i** under the **r** relation, **c**_**i**,**r**_ is a regularization factor, *W* denotes the parameters of the layer, and *σ* is the activation function. The implication of Equation (4) is that, for the node **i**, the representation of the neighboring nodes under each relationship connected to it is aggregated and added to the representation of the node **i** itself as the final representation. The node update based on the heterogeneous network graph is thus completed by Equation (4), and triples are then scored by Equation (5). **h**^**L**^, R, and **t**^**L**^ represent the final embedded representations of the head entity, relationship, and tail entity, respectively, and a higher score in Equation (5) indicates a more realistic triple.
(3) In addition to the RotatE model and the RGCN model, other advanced models were selected for comparison experiments to find the best model in order to perform the subsequent prediction task. RotatE is essentially a distance transformation model, RGCN is a classical heterogeneous graph neural network model, and we also selected TransE [46], which is also a distance transformation model, the Gaussian embedding model KG2E [47], the semantic matching model DistMult [48], and CompGCN [49], a model that combines knowledge graph algorithms with graph neural network algorithms.

#### 2.3.2. Link Prediction Model

Based on the constructed MTP network, we used knowledge graph- or graph neural network models to perform link prediction, i.e., to predict whether there is an edge between two nodes or whether it is a fact triple [46]. In the prediction task, for each triple, there are both fixed head entity and relationship to predict tail entity, and fixed tail entity and relationship to predict head entity. We refer to the model that performs the link prediction task as the link prediction model.

The constructed MTP network for unstable angina as a dataset contains 573 nodes and 12629 edges. We used the above model to complete ten-fold cross-validation, training on the training set while validating on the testing set [50]. The performance of the model is evaluated using three metrics, Hits@k, MR, and MRR [46]:

(6) $$\begin{array}{c} \text{,} \end{array}$$

(7) $$\begin{array}{c} MR=\frac{1}{N}\overset{N}{\underset{i=1}{\sum }}{rank}_{i}\text{,} \end{array}$$

In Equation (6), the average of the number of correct predictions among the top *k* predictions for each triple is calculated. **I** is the indicator function, which is 1 if the condition is true and 0 otherwise. **rank**_**i**_ is the rank of the correct triple among the predicted triples. **N** denotes the number of all triples to be predicted. **MR** and **MRR** are the mean values of the correct triple rank and the inverse of the correct triple rank, respectively.

#### 2.3.3. Multilabel Classification Model

We used the six models described above to obtain the embeddings of nodes and then used a two-layer fully connected neural network (MLP) to predict multiple labels for target genes or pathways [25–51], with the sigmoid function as the final activation function. We refer to the model that performs the multi-label classification task as the multi-label classification model. For the multilabel classification task, we still used the MTP network as the dataset for ten-fold cross-validation to assess the performance of the model using accuracy as a metric. Accuracy was calculated by equations (8)–(10).

There are 21 labels for targets (*T*) and 5 labels for pathways (*P*), representing the tissue localization of targets and classification of pathways, respectively. We first converted the label values to 0 or 1 using Equation (8), and then, we used Equations (9) and (10) to calculate accuracy values.

(8) $$\begin{array}{c} \begin{array}{c} {Label}_{i}=I\left({Label}_{i}>0\right) \end{array}\text{,} \end{array}$$

(9) $$\begin{array}{c} \begin{array}{c} {ACC}_{j}=\frac{1}{K}\overset{K}{\underset{i=1}{\sum }}count\left({Pred}_{i}={Label}_{i}\right) \end{array}\text{.} \end{array}$$

(10) $$\begin{array}{c} \begin{array}{c} ACC=\frac{1}{N}\overset{N}{\underset{j=1}{\sum }}{ACC}_{j} \end{array}\text{.} \end{array}$$

**Label**_**i**_ and **Pred**_**i**_ are the label value and predicted value, respectively. **I** is the indicator function, and if the label value exists and is >0, then the label value is converted to 1; otherwise, it is converted to 0. **K** represents the number of label values, while **N** represents the total number of *T* or *P*. Equations (9) and (10) show that the accuracy of each *T* or *P* is calculated and then averaged.

#### 2.3.4. Comparison Experiments

The aim of the comparison experiments is to find the best link prediction model and the best multilabel classification model [52]. We used RotatE, TransE, KG2E, DistMult, RGCN, and CompGCN models to perform *M*-*T*, *T*-*T*, and *T*-*P* link prediction tasks, respectively, as well as the multilabel classification of *T* and the multilabel classification of *P*, respectively. The embedding dimension of nodes was 64, the learning rate was 0.001, and the epoch was 50.

#### 2.3.5. Parameter Optimization Experiments

The aim of the parameter optimization experiments is to find the optimal parameters for each model [53]. Three parameters were selected for the parameter optimization experiments, namely, the embedding dimension, epoch, and learning rate. The embedding dimension was set to 32, 64, 96, and 128, the epoch was set to 25, 50, 100, and 200, and the learning rate was set to 0.0001, 0.001, 0.01, and 0.1, respectively.

### 2.4. Case Studies

Elucidating the function of miRNAs can help us understand a disease more accurately. There is still a lack of systematic studies on miRNAs in unstable angina [4]. Therefore, to obtain hub miRNAs by the best network analysis method, we conducted a case study using the best link prediction model. Specifically, the potential target genes of these hub miRNAs were predicted, i.e., the *M*-*T* link prediction task. For the prediction results, we performed a three-step validation method.

### 2.5. Three-Step Validation Method

The importance and reliability of hub miRNAs have been validated in the network analysis section. In this section, we validate the results of the network modelling section using a designed three-step validation method.

#### 2.5.1. Validation of Hub miRNA-Target Gene Interactions

First, we assessed the reliability of the predicted hub miRNA-target gene interactions by searching the literature or other databases [54]. The assessment was performed using the TopK metric, meaning the proportion of predictions that was correct in the top *K* rankings [55]. The TopK results of all hub miRNAs were then averaged.

#### 2.5.2. Validation of the Potential of Target Genes as Biomarkers

In the second step, we validated the predicted new and existing target genes for hub miRNAs, which comprised two validation methods.

1. The target genes of hub miRNAs were validated for a new dataset (GSE60993) containing gene expression from unstable angina and healthy controls, using AUC and AUPR as evaluation metrics.
2. Transcription factors (TFs) are important proteins that regulate gene expression [56], and their dysregulation will cause abnormal gene expression, which is closely associated with the development and progression of complex diseases [57]. The TF-Marker database [58] provides cell- and tissue-specific TFs and related markers. We searched the TF-Marker database to verify whether the target genes of hub miRNAs are TFs or related markers in tissues such as the heart, blood vessels, and arteries, which are closely associated with the development of unstable angina.

#### 2.5.3. Validation of the Function of Target Genes

Finally, KEGG functional enrichment analysis was performed on these target genes [59]. The reliability of the predictions was further assessed to know whether enriched pathways were classical and critical pathways in unstable angina.

Through these three steps, the reliability of miRNA target genes, the reliability of target genes as biomarkers, and the reliability of the functions performed by the target genes were successively validated.

## 3. Results

### 3.1. MTP Network

There were 386 differentially expressed miRNAs in unstable angina, obtained by differential expression analysis and after screening. By searching the target genes of miRNAs and the genes of unstable angina and taking the intersection, 232 intersecting genes were obtained, corresponding to 238 miRNAs, with a total of 2706 miRNA-target gene interactions.

For these intersecting genes, after setting the species to *Homo sapiens* and the minimum interaction score to 0.4, a total of 8696 protein-protein interactions were found. Next, KEGG functional enrichment was performed, and a total of 103 pathways were screened, resulting in a total of 1361 gene-pathway interactions. The MTP network is summarized in Table 2, and the detailed data are available in Table S2 in Supplementary Materials. *M*, *T*, and *P* refer to miRNAs, target genes, and pathways, respectively. In the knowledge graph model, *M*-*T*, *T*-*T*, and *T*-*P* also denote the (*M*, MT, and *T*), (*T*, TT, and *T*), and (*T*, TP, and *P*) triples, respectively.

### 3.2. Network Analysis of Several Methods

#### 3.2.1. Modules and Hub miRNAs Based on the WGCNA Method

**Figure 3:**
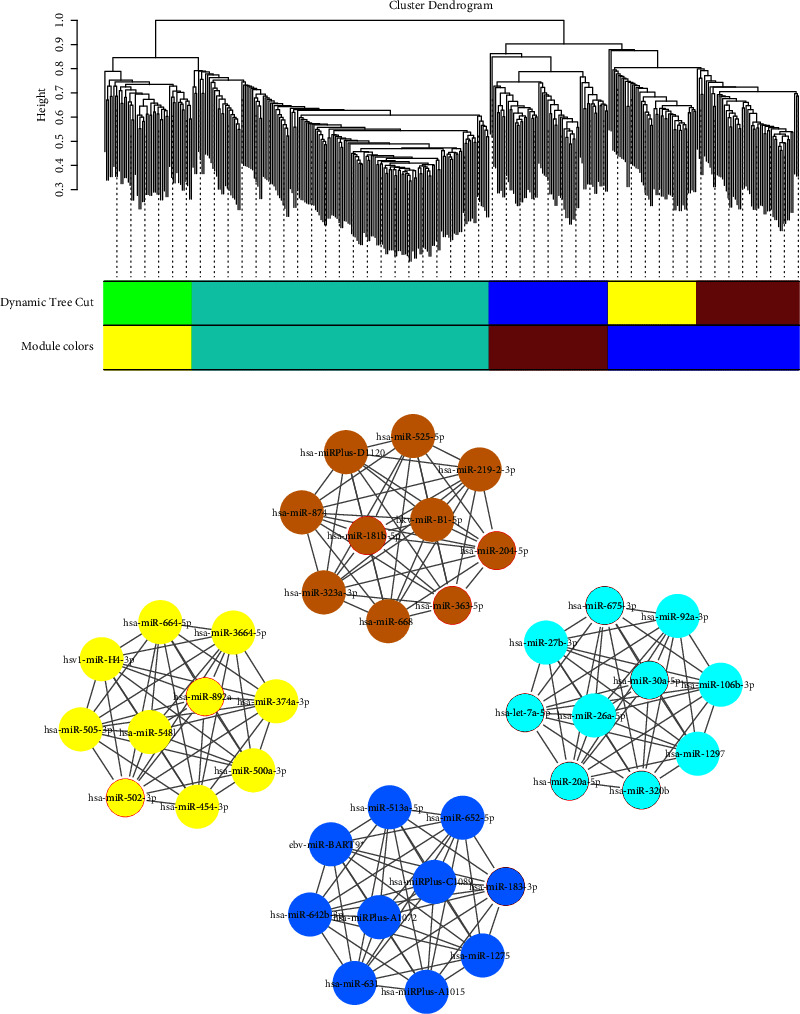
Results of the WGCNA method. (a) Generation of modules from the miRNA coexpression network. (b) The top 10 hub miRNAs in each module. The nodes marked with red borders are hub miRNAs obtained after the final screening.

Differentially expressed miRNAs may play an important role in disease states [60], and either overexpressed or underexpressed miRNAs were included in our study. We used the expression data from these miRNAs to construct a weighted miRNA coexpression network, divided modules using the dynamic tree cutting method, and merged the modules to finally obtain four modules. The results are shown in Figure 3(a).

A total of four colored modules, yellow, turquoise, brown, and blue, were generated, from which hub miRNAs were searched, respectively. The correlation between miRNAs was used as the weight of edges, and the importance of the nodes in each module was ranked according to the connectivity [61]. The top 10 nodes in each module are shown in Figure 3(b), and a total of 40 hub miRNAs were filtered out. The thickness of edges is proportional to the correlation value.

#### 3.2.2. Similarity Network and the Scale-Free Network Based on the SimCluster Method

**Figure 4:**
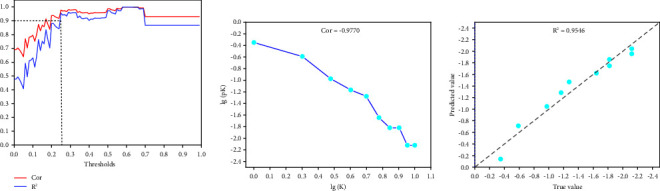
Generation of the scale-free first-order similarity network. (a) Pearson's correlation coefficient Cor and linear regression *R*^2^ between lg (*K*) and lg (*pK*) when setting different first-order similarity thresholds. *K* refers to the degree value, while *pK* refers to the frequency of the degree value. (b) The distribution of lg (*K*) and lg (*pK*) and the Pearson correlation coefficient. (c) *R*^2^ of the linear regression equations for lg (*K*) and lg (*pK*), and the relationship between the predicted and true values. The threshold value was set to 0.25.

As can be seen from Table 3, the results of first-order similarity are better than those of second-order similarity.  Figure 4(a) shows the variation of Pearson's correlation coefficient and linear regression (ordinary least squares) *R*^2^ when different first-order similarity thresholds are set. The similarity value at which both Cor and *R*^2^ are greater than 0.9 is used as the threshold (0.25), and links greater than this threshold are retained, while links less than this threshold are excluded.

**Figure 5:**
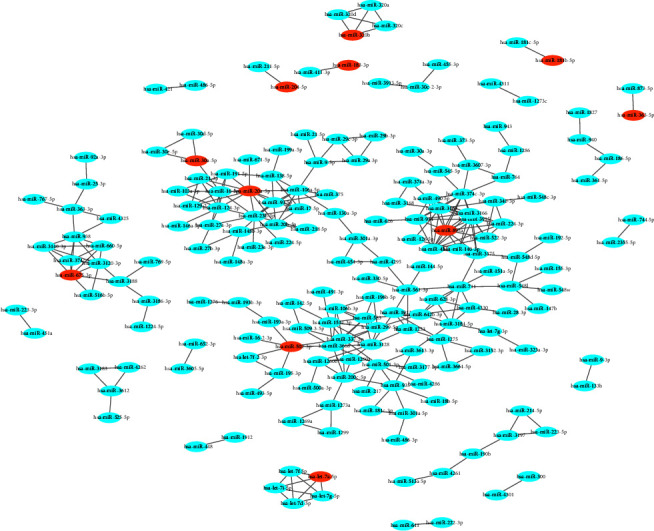
The scale-free first-order similarity network for miRNAs. The nodes in red are hub miRNAs obtained after the final screening.

Figures 4(b) and 4(c) show the evaluation of whether a similarity network greater than the threshold is a scale-free network. It can be seen that lg (*K*) is highly correlated with lg (*pK*) and that the distribution of the two is linear. There are more nodes with a small degree *K* and fewer nodes with a large degree *K*. Fewer nodes connect most of the nodes, which is a characteristic of scale-free networks. The scale-free first-order similarity network is shown in Figure 5.

The linear regression equations for the SimCluster_1 method (SimCluster based on first-order similarity) and the SimCluster_2 method (SimCluster based on second-order similarity) can be found in Table S3 in Supplementary Materials.

#### 3.2.3. Hub miRNAs Obtained Based on the SimCluster Method

Modules can be obtained after network clustering of the scale-free similarity network, and thus, hub miRNAs affecting each module can be obtained. We have demonstrated in a previous study that the fast greedy algorithm is a good network clustering algorithm [29], and in this study, we have also demonstrated that the fast greedy algorithm is the best among the four network clustering algorithms by calculating modularity values. The results of the comparison of network clustering algorithms are presented in Supplementary Materials.

We used the fast greedy algorithm to perform network clustering of the scale-free similarity network, finding the nodes with the largest degree value from each module as hub miRNAs. In some modules, there were many nodes sharing the largest degree value, and these were included in subsequent studies.

Final hub miRNAs were obtained by taking the intersection of the results of the SimCluster method and the WGCNA method (see the nodes marked with red borders in Figure 3(b) or the red nodes in Figure 5). Hub miRNAs obtained by the six network analysis methods, including the SimCluster method, are listed in Supplementary Materials.

#### 3.2.4. Performance Comparison of Network Analysis Methods

We compared the performance of these network analysis methods using the mean AUC values and mean AUPR values of hub miRNAs obtained from each method when distinguishing between the disease and control groups. Table 3 shows the performance of hub miRNAs from the six methods. It can be seen that SimCluster_1 has better mean AUC values than the WGCNA method for both datasets, and the WGCNA & SimCluster_1 method has significantly improved over the WGCNA method in terms of both mean AUC values and mean AUPR values. The important point is that these results reveal that the network analysis approach combining correlation and similarity (WGCNA & SimCluster) gives better results than the approach using correlation (WGCNA) or similarity (SimCluster) alone, which is a new approach to finding hub nodes in the network. In addition, the WGCNA & SimCluster_2 method and the FC_hub method performed well for the original dataset and mediocrely for the new dataset, suggesting that neither the second-order similarity results nor the FoldChange results generalize well.

**Figure 6:**
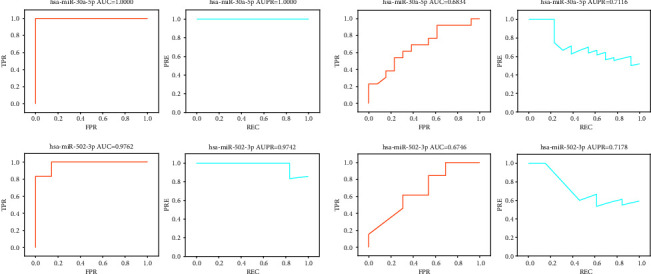
ROC curves and PR curves of two hub miRNAs for the original and new datasets. (a) Results of two hub miRNAs for the original dataset. (b) Results of two hub miRNAs for the new dataset.

Hub miRNAs obtained by the WGCNA & SimCluster_1 method achieved the best results for the new dataset. We next used hub miRNAs obtained based on the WGCNA & SimCluster_1 method for our subsequent study.  Figure 6 shows two of these hub miRNAs, hsa-miR-30a-5p and hsa-miR-502-3p. Both of these hub miRNAs had an AUC and AUPR above 0.97 for the original dataset, and both had an AUC above 0.67 and an AUPR above 0.71 for the new dataset. This shows the potential of these hub miRNAs as biomarkers, and it would be meaningful to conduct in-depth studies.

### 3.3. Link Prediction

The MTP network constructed is a small complex heterogeneous network, as shown in Table 2, containing three types of nodes and edges. A knowledge graph model or a graph neural network model can complete node-level, edge-level, or even graph-level modelling of complex heterogeneous networks, using existing data to predict unknown data [42].

We first performed link prediction for *M*-*T*, *T*-*T*, and *T*-*P*, which are predictions for the triples (*M*, MT, and *T*), (*T*, TT, and *T*), and (*T*, TP, and *P*) in the knowledge graph model. Comparison experiments and parameter optimization experiments were carried out using six evaluation metrics, Hits@5, Hits@10, Hits@,20, Hits@50, MR, and MRR. We refer to the models performing the link prediction task collectively as link prediction models.

#### 3.3.1. Comparison Experiments

**Figure 7:**
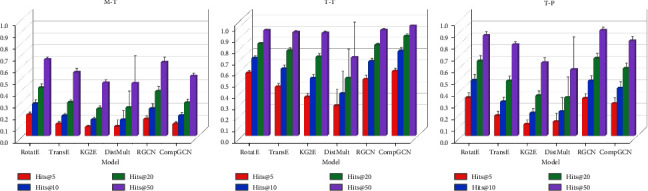
Link prediction results for *M*-*T*, *T*-*T*, and *T*-*P* calculated by Hits@k. (a) *M*-*T* link prediction. (b) *T*-*T* link prediction. (c) *T*-*P* link prediction.

Comparison experiments were conducted using six models, RotatE, TransE, KG2E, DistMult, RGCN, and CompGCN, to find the best link prediction model. Figures 7(a)–7(c) show the link prediction results for *M*-*T*, *T*-*T*, and *T*-*P* calculated by Hits@k, respectively.  Table 4 shows the link prediction results calculated by MR and MRR. The data in the figure and table are the means and standard deviations of the results after ten-fold cross-validation.

It can be seen that for *M*-*T* link prediction, the RotatE model achieved the best results for all six metrics. For *T*-*P* link prediction, the RotatE model achieved the best results in four of the six metrics, namely, Hits@5, Hits@10, MR, and MRR. Finally, in *T*-*T* link prediction, the RotatE model was slightly inferior to the best performing CompGCN model, but the run time of the RotatE model was only one-twentieth of that of the CompGCN model. Therefore, the RotatE model showed strong predictive power for *M*-*T*, *T*-*T*, and *T*-*P* predictions, and in particular, it showed the best prediction results for *M*-*T* and *T*-*P* predictions.

In addition, we can find that all these knowledge graph models or graph neural network models performed better for *T*-*T* link prediction and *T*-*P* link prediction, while they performed worse for *M*-*T* link prediction. In the *M*-*T* prediction task, Hits@5 of the best model was only 0.1789 ± 0.0167, while in the *T*-*T* and *T*-*P* prediction tasks, Hits@5 of the best model was 0.5751 ± 0.0227 and 0.3220 ± 0.0377, respectively. This may be due to the limitations of the model or insufficient information contained in the MTP network.

We finally selected the RotatE model as the link prediction model to perform subsequent link prediction tasks. The results of this section are also presented in Table S4 in Supplementary Materials.

#### 3.3.2. Parameter Optimization Experiments

We demonstrated through comparison experiments that the RotatE model has good performance on all three types of edge prediction and is therefore a good link prediction model. Next, we chose the RotatE model for parameter optimization experiments to find the most suitable parameters. The three parameters optimized were the embedding dimension, learning rate, and epoch. In this study, we focused more on the function played by miRNAs, so we only performed parameter optimization experiments for M-T link prediction.

**Figure 8:**
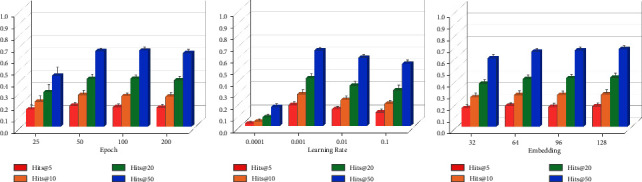
Results of parameter optimization experiments calculated by Hits@k. (a) Epoch experiments. (b) Learning rate experiments. (c) Embedding dimension experiments.

Figures 8(a)–8(c) show the changes in the results of Hits@k metrics after the parameters, the epoch, embedding dimension, or learning rate, were changed, respectively. When the parameters were changed, the RotatE model had the best results for Hits@5 and Hits@10 at an epoch of 50, for Hits@5, Hits@10, Hits@20, and Hits@50 at a learning rate of 0.001, and for Hits@5 at an embedding dimension of 64.

Table 5 shows the results calculated by MR and MRR. In the range of parameter variations, the RotatE model gave the best results for the metrics at an epoch of 50 and a learning rate of 0.001. The results were very close for both MR and MRR at 64 and 128 embedding dimensions, and again, since the best results were obtained for Hits@5 at an embedding dimension of 64, we still set the embedding dimension to 64.

Therefore, by optimizing the three parameters, we finally chose an epoch of 50, an embedding dimension of 64, and a learning rate of 0.001 as the final parameter settings for the *M*-*T* link prediction task.

### 3.4. Multilabel Classification

**Figure 9:**
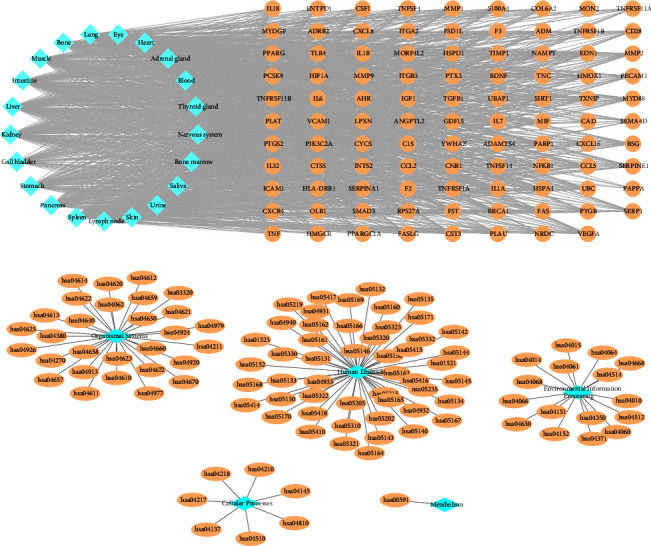
Multilabel classification of targets and pathways. (a) Tissue localization of target genes. (b) Classification of pathways.

The multilabel classification task that we have accomplished is to classify targets and pathways into multiple categories [25]. Specifically, the multilabel classification of targets refers to the classification of the tissue localization of targets. From the data obtained, targets can be distributed to as many as 21 tissues, with the top five being the blood, liver, nervous system, lungs, and heart.  Figure 9(a) shows the distribution of targets in different tissues, with the thickness of the lines proportional to the amount of gene expression.

The multilabel classification of pathways refers to the division of pathways into different broad categories. The KEGG database classifies all human pathways into 7 broad categories, and the pathways enriched by the target genes of these miRNAs can be classified into 5 of these 7 broad categories.  Figure 9(b) shows the classification of the enriched pathways into the five categories of organismal systems, cellular processes, human diseases, environmental information processing, and metabolism.

We first converted the label value of the target or pathway to 0 or 1, and if the target or pathway existed in a category, then the label value was 1; otherwise, it was 0. We then used a knowledge graph or graph neural network model to obtain the embedding representation of nodes and a 2-layer MLP to predict the multilabel classification of nodes. The means and standard deviations of the results after ten-fold cross-validation are shown in Table 6.

It can be seen that the RGCN model achieved the best performance in the multi-label classification prediction task for both *T* and *P*, with accuracy rates as high as 0.8312 ± 0.0220 and 0.8356 ± 0.0404, respectively. Therefore, the RGCN model is the best multilabel classification model for both types of nodes for the prediction task.

### 3.5. Case Studies

Through comparison experiments, we have demonstrated that the RotatE model is the best link prediction model, and through parameter optimization experiments, we have found the optimal parameters for the RotatE model. In this section, we conduct a case study of hub miRNAs obtained by the WGCNA & SimCluster_1 method.

The role of miRNAs in regulating gene expression is well worth investigating in depth. Unstable angina is a relatively complex disease, and the functions played by miRNAs in this disease are still not fully elucidated. Studying the function of hub miRNAs is a convenient way to understand the function of all miRNAs [62]. We used the RotatE model to predict the potential target genes of hub miRNAs, the unknown M-T link prediction task. This is also referred to as a complementary task for the knowledge graph.

Eleven hub miRNAs were obtained by the WGCNA & SimCluster_1 method. We performed M-T link prediction for these 11 hub miRNAs, and the detailed prediction results are shown in Table S5 in Supplementary Materials. When predicting potential M-T links, for each hub miRNA, the top 10 target genes in terms of predicted scores were taken, so there were 110 hub miRNA-target gene interactions in total. After de-duplication, only 38 genes of the predicted target genes remained.

### 3.6. Validation of the Results

**Figure 10:**
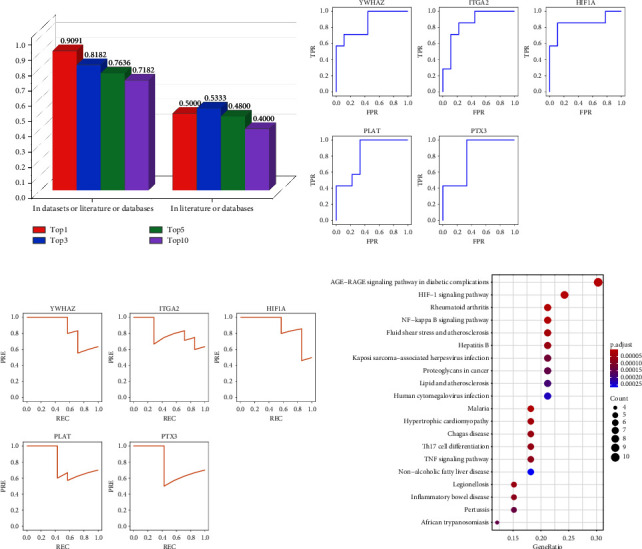
Validation results of the three-step validation method. (a) Validation of hub miRNA-target gene interactions. (b) ROC curves for the five target genes of hub miRNAs for the new dataset. (c) PR curves for the five target genes of hub miRNAs for the new dataset. (d) Validation of the function of the target genes.

In the network analysis section, we have used two datasets to validate the best network analysis method and hub miRNAs obtained by the method. In the network modelling section, the results for the potential target genes of hub miRNAs also need to be validated to demonstrate the reliability of model predictions [63]. Using the designed three-step validation method, we first validated the reliability of hub miRNA-target gene interactions. As can be seen in Figure 10(a), 50%, 53%, 48%, and 40% of the top 1, 3, 5, and 10 predictions, respectively, were validated by the literature or other databases. That is, after discarding those interactions that had already appeared in the dataset, a large proportion of the predicted unknown hub miRNA-target gene interactions were validated.

Second, the predicted target genes of hub miRNAs were validated using two different methods. On the one hand, based on a new gene expression dataset, we tested whether these target genes could distinguish unstable angina samples from control samples, i.e., whether they had potential as biomarkers. Figures 10(b) and 10(c) show the ROC curves and PR curves for 5 of the 38 target genes. It can be seen that these target genes can distinguish well between disease and control samples, and their differential expression between samples perhaps gives them the function of biomarkers. On the other hand, we searched the TF-Marker database to determine whether these target genes were transcription factors or related markers, or whether they were transcription factors or related markers that were closely associated with unstable angina.  Table 7 shows the search results of the TF-Marker database. It can be found that six of the ten newly predicted target genes are transcription factors or related markers, and three of them are directly associated with unstable angina, with the proportions of 60% and 30%, respectively, which are larger than the proportions in the training or validation sets. Therefore, we validated the potential of these target genes as biomarkers in two ways.

Finally, we performed KEGG functional enrichment on the predicted target genes of hub miRNAs, and the top 20 pathways at a *p* value are shown in bubble plots (see Figure 10(d)). The bubble size indicates the number of genes, with red colors indicating smaller *p* values, and GeneRatio is the ratio of the number of genes enriched into the pathway to the total number of genes used, with larger ratios indicating a greater number of genes involved in the pathway. As shown in Figure 10(d), these target genes were enriched in pathways such as lipid and atherosclerosis (gene number = 7, *p* < 0.0001), fluid shear stress and atherosclerosis (gene number = 7, *p* < 0.0001), and the NF-kappa *B* signaling pathway (gene number = 7, *p* < 0.0001) [64–66], which were associated with the development and progression of unstable angina. Therefore, the predicted target genes were validated from a functional enrichment perspective, and many of the enriched pathways are involved in the pathology of unstable angina. In conclusion, we validated the veracity and reliability of the model's results through a cascade of three steps.

## 4. Discussion

Many noncoding RNAs are regulators and play an important role in the process of gene expression in a regulatory manner [67]. Currently, miRNAs are considered important types of noncoding RNAs and mostly play a role in regulating gene expression in a negative regulatory manner [3]. The expression of some miRNAs and their regulated target genes (mRNAs) varies in different diseases and is disease-specific [68]. Therefore, it is theoretically feasible to find miRNAs or genes that are specific to a disease and act as biomarkers or diagnostic markers [69]. Unstable angina is a complex disease with multifactorial and multisystemic involvement, and the pathogenesis and treatment of this disease still require in-depth investigation. Previous studies on unstable angina have mostly focused on genes and function while neglecting regulatory functions played by regulatory factors such as miRNAs and are therefore inadequate and incomplete [70, 71]. In this study, we designed a research strategy using miRNA expression data and miRNA regulatory networks to first find the best performing WGCNA & SimCluster_1 method by comparing six network analysis methods for original and new datasets, and then, we used this method to obtain hub miRNAs that could be used as biomarkers. The best model from the network modelling section was then used to predict unknown functions based on the existing functions of hub miRNAs.

 The miRNA regulatory network is also a complex network and the WGCNA approach only analyzes the correlation network constructed based on the expression levels of miRNAs and does not take full advantage of the other information in the complex network. The similarity of nodes in a network is also important information that can be exploited [72]. We constructed a first-order similarity network and a second-order similarity network based on the similarity of miRNA actions and designed a network analysis algorithm to find hub miRNAs using the similarity network. Notably, we used the formation of scale-free networks as the judgment criterion when screening similarity thresholds, which coincided with the WGCNA method when screening soft thresholds [61]. A comparison of multiple network analysis methods showed that the WGCNA & SimCluster_1 method, which combines correlation and similarity, achieved the best results for the new dataset. This suggests that it is feasible to use correlation and similarity between miRNAs to screen for hub miRNAs, with better results than using similarity or correlation alone.

Knowledge graph models are mostly used for large, complex heterogeneous graphs, often containing hundreds or thousands of types of nodes and edges [73]. Because various heterogeneous graphs are so different from each other, researchers have developed a variety of dedicated knowledge graph models, so that each model has its own advantages [74]. In fact, the MTP network that we have constructed is a small, complex heterogeneous network, and the knowledge graph model performs equally well on small heterogeneous networks. Of the six models we used, RotatE, TransE, KG2E, and DistMult are knowledge graph models, RGCN is a classical graph neural network model, and CompGCN is a model combining graph neural networks with knowledge graphs, all of which are trained in the same way as knowledge graph modelling. The knowledge graph model uses a set of many triples as a dataset and evaluates the performance of the model by predicting missing head or tail entities and using a scoring function. The task of predicting missing head or tail entities can also be considered a link prediction task, and the discovery of unknown links is of practical importance [46].

In this study, we performed node-level and edge-level predictions for the MTP network using a knowledge graph or graph neural network model. In edge-level modelling, we made predictions for all three types of edges in the network. For the Hits@5 metric, the RotatE model yielded results of 0.1789 ± 0.0167, 0.5600 ± 0.0185, and 0.3220 ± 0.0377 for *M*-*T*, *T*-*T*, and *T*-*P* link prediction, respectively, with the best performance for *M*-*T* and *T*-*P* link predictions and the second-best performance for *T*-*T* link prediction after the CompGCN model. In terms of MR and MRR metrics, the RotatE model also performed best for *M*-*T* and *T*-*P* link predictions and worse than the CompGCN and RGCN models for *T*-*T* link prediction. This shows that the RotatE model has good predictive ability and performs better than other advanced models. In node-level modelling, for all six models, the RGCN model achieved the best performance for multilabel classification prediction for both *T* and *P*, with accuracy rates of 0.8312 ± 0.0220 and 0.8356 ± 0.0404, respectively. This suggests that the graph neural network model has an advantage in node-level prediction [75]. We also performed parameter optimization experiments on the RotatE model when performing the *M*-*T* link prediction task, and the best performance was achieved when setting the epoch, embedding dimension, and learning rate to 50, 64, and 0.001, respectively.

The hub miRNAs that we have selected as key nodes in each module may play a vital regulatory role in the disease [76]. The functions of these hub miRNAs are well worth investigating in depth. We performed an *M*-*T* link prediction task on 11 hub miRNAs using the best link prediction model and optimal parameters to predict the target genes of these hub miRNAs and perform functional enrichment. The reliability of the results can only be demonstrated after the model has been validated [77], so we developed a three-step validation method for the designed model and the content of this study to perform a three-part validation. In the first part, we validated a total of 110 predicted hub miRNA-target gene interactions by searching the literature or other databases. The percentage of correct predictions in the top 1, top 3, and top 5 was 50%, 53%, and 48%, respectively. In the second part, the target genes of hub miRNAs had the ability to distinguish well between different groups of samples for the gene expression dataset. Moreover, 60% of the predicted novel target genes were transcription factors or markers, and 30% were directly associated with the development of unstable angina. In the third part, many of the enriched pathways were associated with unstable angina, which, on the other hand, proved the reliability of predicted target genes. In terms of specific mechanisms, lipid levels in blood and fluid shear stress of local blood are important for the pathogenesis of coronary atherosclerosis [65, 66], which is an important pathological feature of unstable angina. NF-kappa *B* is a key transcription factor involved in many physiological and pathological processes, including immune response, apoptosis, and inflammation [64]. Studies [78, 79] have shown that in the pathology of atherosclerosis, NF-kappa *B* is critical for the crosstalk between cytokines, adhesion molecules, and growth factors, leading to the formation, growth, and eventual rupture of atherosclerotic plaques. This three-step validation method contains three parts in sequential order, validating the results of our model in a cascading manner by verifying miRNA, target gene, and function in succession.

It can be seen that although the WGCNA & SimCluster_1 method performs best for the new dataset, the AUC and AUPPR are still low, and further improvement on this basis is necessary. In addition, none of the six knowledge graphs or graph neural network models that we used performed very well for *M*-*T* link prediction, which may be due to limitations in the models themselves or insufficient information in the MTP networks used. In the future, we will seek to develop superior models or use larger heterogeneous networks and we will also conduct generalization ability experiments in order to generalize the present modelling strategy to other diseases.

## 5. Conclusions

We constructed a complex heterogeneous network regulated by miRNAs in unstable angina and then analyzed and modelled it, which explored the functions played by noncoding RNAs in complex diseases from the miRNA perspective. Among the six network analysis methods for finding hub miRNAs, the WGCNA & SimCluster_1 method yielded the best results for the new dataset, identifying hub miRNAs that could act as biomarkers. Comparative experiments with six knowledge graphs or graph neural network models demonstrated that RotatE is a good link prediction model and that RGCN is the best multilabel classification model for the miRNA regulatory network. Optimal parameters for the M-T link prediction task were obtained by parameter optimization experiments. The results of the predicted target genes of hub miRNAs based on the best model and the best parameters were validated by three methods. Our modelling strategy can be used as a reference for other disease and noncoding RNA studies.

## Figures and Tables

**Table 1:** Comparison of the three network analysis methods.

		WGCNA	SimCluster	WGCNA & SimCluster
Data	Data sources	miRNA expression data	MTP network	Data used in the WGCNA method and SimCluster method
	Constructed matrices	Correlation matrix and the topological overlap matrix	First-order similarity matrix or the second-order similarity matrix	

Methods	Core principles	Pearson's correlation of miRNA expression data	Jaccard similarity of miRNAs at the target gene level or pathway level	Intersection of the results of the WGCNA method and the SimCluster method
	Parameters of scale-free networks	Soft threshold	Similarity threshold	
		*R * ^2^ greater than 0.9	Both *R*^2^ and the correlation coefficient should be greater than 0.9	
	Module generation	Hierarchical clustering	Network clustering	

Results	Number of hub miRNAs	40	63	11
	Mean AUC values for the new dataset	0.5191	0.5319	0.6292
	Mean AUPR values for the new dataset	0.6019	0.6069	0.6773

Note. In this table, the results of the SimCluster method and the WGCNA & SimCluster method are calculated based on first-order similarity.

**Table 2:** Nodes and edges of the MTP network.

Nodes or edges	*M*	*T*	*P*	*M*-*T*	*T*-*T*	*T*-*P*
Number	238	232	103	2706	8562	1361

**Table 3:** Comparison of the performance of hub miRNAs obtained by several network analysis methods.

		WGCNA	FC_hub	SimCluster_1	WGCNA & SimCluster_1	SimCluster_2	WGCNA & SimCluster_2
Original dataset	Mean_AUC	0.5071	0.9083	0.5771	0.6190	0.5884	0.9905
	Mean_AUPR	0.6689	0.9118	0.6620	0.7112	0.6679	0.9911

New dataset	Mean_AUC	0.5191	0.5197	0.5319	**0.6292**	0.5166	0.5296
	Mean_AUPR	0.6019	0.5862	0.6069	**0.6773**	0.5960	0.6026

Note. FC_hub refers to the top 20 miRNAs ranked by |logFC|. SimCluster_1 and SimCluster_2 refer to the results obtained based on first-order similarity and second-order similarity, respectively. “&” refers to the results of the intersection of the two methods. Bold values indicate the best results on the new dataset.

**Table 4:** Link prediction results for *M*-*T*, *T*-*T*, and *T*-*P* calculated by MR and MRR.

		RotatE	TransE	KG2E	DistMult	RGCN	CompGCN
MT	MR	**7.9155** **±** **0.7216**	12.7434 ± 1.581	16.6473 ± 1.9285	29.1253 ± 31.6744	9.9069 ± 1.3969	12.7025 ± 1.3974
	MRR	**0.1274** **±** **0.0134**	0.0795 ± 0.0090	0.0608 ± 0.0071	0.0638 ± 0.0343	0.1031 ± 0.0171	0.0796 ± 0.0087

TT	MR	3.3667 ± 0.1477	8.9800 ± 0.2236	4.2304 ± 0.2909	16.3310 ± 24.3477	3.3050 ± 0.1418	**2.9691** **±** **0.1183**
	MRR	0.2976 ± 0.0135	0.1402 ± 0.0099	0.2373 ± 0.0154	0.1675 ± 0.0847	0.3031 ± 0.0132	**0.3373** **±** **0.0134**

TP	MR	**4.4050** **±** **0.4242**	7.8392 ± 1.0865	12.4661 ± 2.2471	24.3245 ± 31.8611	4.4954 ± 0.3793	5.2599 ± 0.6200
	MRR	**0.2288** **±** **0.0210**	0.1297 ± 0.0174	0.0826 ± 0.0151	0.0941 ± 0.0501	0.2239 ± 0.0189	0.1926 ± 0.0234

Bold values indicate the best results on that task.

**Table 5:** Results of parameter optimization experiments calculated by MR and MRR.

Epoch	25	**50**	100	200
MR	10.3123 ± 1.9440	**7.9155** **±** **0.7216**	8.3054 ± 0.8194	8.155 ± 0.7939
MRR	0.1000 ± 0.0177	**0.1274** **±** **0.0134**	0.1215 ± 0.0123	0.1236 ± 0.0115
Embedding	32	64	96	**128**

MR	9.0563 ± 0.7624	7.9155 ± 0.7216	8.1209 ± 0.8165	**7.9137** **±** **0.8641**
MRR	0.1111 ± 0.0091	0.1274 ± 0.0134	0.1243 ± 0.0131	**0.1276** **±** **0.0131**
Learning rate	0.0001	**0.001**	0.01	0.1

MR	44.1148 ± 7.67	**7.9155** **±** **0.7216**	9.0002 ± 0.8692	10.4498 ± 1.0269
MRR	0.0234 ± 0.0046	**0.1274** **±** **0.0134**	0.1120 ± 0.0107	0.0966 ± 0.0101

The bold values indicate the best parameters and the resulting best results.

**Table 6:** Accuracy of multilabel classification of targets and pathways.

		RotatE	TransE	KG2E	DistMult	RGCN	CompGCN
Accuracy	*T*	0.7902 ± 0.0115	0.7766 ± 0.0188	0.7704 ± 0.0235	0.8200 ± 0.0201	**0.8312** **±** **0.0220**	0.8205 ± 0.0248
	*P*	0.8245 ± 0.0400	0.8047 ± 0.0630	0.8127 ± 0.0459	0.8167 ± 0.0634	**0.8356** **±** **0.0404**	0.8307 ± 0.0336

Bold values indicate the best results on that task.

**Table 7:** Number of target genes that are transcription factors or related markers.

Target genes of hub miRNAs	Number of target genes	Number of transcription factors or related markers	Number of transcription factors or related markers directly associated with UA
Newly predicted (not present in the training or validation set)	10	6 (0.60)	3 (0.30)
Present in the training or validation set	28	14 (0.50)	7 (0.25)
Total	38	20 (0.53)	10 (0.26)

## Data Availability

All data can be found in this article or in Supplementary Materials. Codes can be requested from the corresponding author.

## Supplementary Materials

Supplementary Materials Table S1: Acronym explanation table. Table S2: MTP network. Table S3: Results of network analysis. Table S4: Results of network modelling. Table S5: Case studies.
