# Supplementary material for: A Complex Heterogeneous Network Model of Disease Regulated by Noncoding RNAs: A Case Study of Unstable Angina Pectoris
Source: Comput Intell Neurosci. 2022 Dec 23;2022:5852089. doi: 10.1155/2022/5852089 (PMC9803582; doi:10.1155/2022/5852089)
Supplement: Supplementary Materials — Table S1: Acronym explanation table. Table S2: MTP network. Table S3: Results of network analysis. Table S4: Results of network modelling. Table S5: Case studies. [file 5852089.f1.zip › Acronym Explanation Table.docx]

| Acronym | Explanation | Acronym | Explanation |
| --- | --- | --- | --- |
| WGCNA | Weighted Gene Co-expression Network Analysis | Hits@k | the average of the number of correct predictions among the top k predictions for each triple |
| MTP | miRNA-target gene-pathway heterogeneous network | MR | the mean value of the correct triple rank |
| UA | Unstable angina | MRR | the mean value of the inverse of the correct triple rank |
| M-T | miRNA-target gene | ACC | Accuracy of multi-label classification models |
| M-P | miRNA-target gene-pathway | FC_hub | the top 20 miRNAs ranked by \|logFC\| |
| T-T | target gene-target gene | SimCluster | Results obtained based on first-order similarity or second-order similarity |
| T-P | target gene-pathway | SimCluster_1 | Results obtained based on first-order similarity |
| lg(K) | the logarithm of degree values | SimCluster_2 | Results obtained based on second-order similarity |
| lg(pK) | the logarithm of degree frequencies | WGCNA & SimCluster | Results obtained based on WGCNA method and SimCluster method |
| AUC | area under the ROC curve | WGCNA & SimCluster_1 | Results obtained based on WGCNA method and SimCluster_1 method |
| AUPR | area under the PR curve | WGCNA & SimCluster_2 | Results obtained based on WGCNA method and SimCluster_2 method |
| h, r, t | the head entity, the tail entity, and the relationship between them | & | the results of the intersection of the two methods |
| 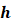   \|  \| \| --- \| | the embedding representations of the head entity | RotatE | the RotatE model |
| 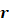   \|  \| \| --- \| | the embedding representations of the relationship | RGCN | the Relational Graph Convolutional Network model |
| 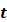   \|  \| \| --- \| | the embedding representations of the tail entity | TransE | the TransE model |
| ***W*** | the parameters of the layer | KG2E | the KG2E model |
| 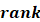   \|  \| \| --- \| | the rank of the correct triple among the predicted triples | DistMult | the DistMult model |
| MLP | fully connected neural network | CompGCN | the CompGCN model |
