# Supplementary material for: A Complex Heterogeneous Network Model of Disease Regulated by Noncoding RNAs: A Case Study of Unstable Angina Pectoris
Source: Comput Intell Neurosci. 2022 Dec 23;2022:5852089. doi: 10.1155/2022/5852089 (PMC9803582; doi:10.1155/2022/5852089)
Supplement: Supplementary Materials — Table S1: Acronym explanation table. Table S2: MTP network. Table S3: Results of network analysis. Table S4: Results of network modelling. Table S5: Case studies. [file 5852089.f1.zip › Case Studies.docx]

| head | tail | head | tail |
| --- | --- | --- | --- |
| hsa-miR-183-3p | NAMPT | hsa-miR-20a-5p | VEGFA |
| hsa-miR-183-3p | C1S | hsa-miR-20a-5p | HIF1A |
| hsa-miR-183-3p | CCN2 | hsa-miR-20a-5p | TXNIP |
| hsa-miR-183-3p | ADAMTS4 | hsa-miR-20a-5p | CYCS |
| hsa-miR-183-3p | TXNIP | hsa-miR-20a-5p | PTGS2 |
| hsa-miR-183-3p | PTGS2 | hsa-miR-20a-5p | ITGA2 |
| hsa-miR-183-3p | VEGFA | hsa-miR-20a-5p | CXCL8 |
| hsa-miR-183-3p | PTX3 | hsa-miR-20a-5p | HMOX1 |
| hsa-miR-183-3p | HMOX1 | hsa-miR-20a-5p | ICAM1 |
| hsa-miR-183-3p | IGF1 | hsa-miR-20a-5p | IL6 |
| hsa-miR-363-5p | VEGFA | hsa-miR-30a-5p | YWHAZ |
| hsa-miR-363-5p | TIMP1 | hsa-miR-30a-5p | TXNIP |
| hsa-miR-363-5p | TXNIP | hsa-miR-30a-5p | AHR |
| hsa-miR-363-5p | SERPINE1 | hsa-miR-30a-5p | VEGFA |
| hsa-miR-363-5p | HIF1A | hsa-miR-30a-5p | HIF1A |
| hsa-miR-363-5p | ITGA2 | hsa-miR-30a-5p | SERPINE1 |
| hsa-miR-363-5p | EDN1 | hsa-miR-30a-5p | HMOX1 |
| hsa-miR-363-5p | ICAM1 | hsa-miR-30a-5p | ITGA2 |
| hsa-miR-363-5p | PTGS2 | hsa-miR-30a-5p | MON2 |
| hsa-miR-363-5p | IGF1 | hsa-miR-30a-5p | PTX3 |
| hsa-miR-181b-5p | SIRT1 | hsa-miR-675-3p | ADM |
| hsa-miR-181b-5p | VEGFA | hsa-miR-675-3p | IL6 |
| hsa-miR-181b-5p | SMAD3 | hsa-miR-675-3p | HSPA4 |
| hsa-miR-181b-5p | PTGS2 | hsa-miR-675-3p | TNF |
| hsa-miR-181b-5p | TNFRSF11B | hsa-miR-675-3p | ADRB2 |
| hsa-miR-181b-5p | HSPA4 | hsa-miR-675-3p | IL1B |
| hsa-miR-181b-5p | HIF1A | hsa-miR-675-3p | PTGS2 |
| hsa-miR-181b-5p | TGFB1 | hsa-miR-675-3p | SMAD3 |
| hsa-miR-181b-5p | AHR | hsa-miR-675-3p | IGF1 |
| hsa-miR-181b-5p | ITGA2 | hsa-miR-675-3p | PARP1 |
| hsa-miR-204-5p | SERPINE1 | hsa-miR-892a | YWHAZ |
| hsa-miR-204-5p | VEGFA | hsa-miR-892a | SERPINE1 |
| hsa-miR-204-5p | PTGS2 | hsa-miR-892a | IL6 |
| hsa-miR-204-5p | TXNIP | hsa-miR-892a | TNF |
| hsa-miR-204-5p | PLAU | hsa-miR-892a | PLAU |
| hsa-miR-204-5p | PLAT | hsa-miR-892a | TXNIP |
| hsa-miR-204-5p | IL6 | hsa-miR-892a | GP5 |
| hsa-miR-204-5p | SIRT1 | hsa-miR-892a | CXCL8 |
| hsa-miR-204-5p | EDN1 | hsa-miR-892a | HIF1A |
| hsa-miR-204-5p | CXCL8 | hsa-miR-892a | VEGFA |
| hsa-let-7a-5p | TXNIP | hsa-miR-502-3p | CCN2 |
| hsa-let-7a-5p | AHR | hsa-miR-502-3p | PTX3 |
| hsa-let-7a-5p | YWHAZ | hsa-miR-502-3p | HIF1A |
| hsa-let-7a-5p | PARP1 | hsa-miR-502-3p | SIRT1 |
| hsa-let-7a-5p | VEGFA | hsa-miR-502-3p | SMAD3 |
| hsa-let-7a-5p | PTGS2 | hsa-miR-502-3p | TXNIP |
| hsa-let-7a-5p | HIF1A | hsa-miR-502-3p | HMOX1 |
| hsa-let-7a-5p | CXCL8 | hsa-miR-502-3p | VEGFA |
| hsa-let-7a-5p | PAPPA | hsa-miR-502-3p | TGFB1 |
| hsa-let-7a-5p | IL6 | hsa-miR-502-3p | IGF1 |
| hsa-miR-320b | SIRT1 |  |  |
| hsa-miR-320b | PARP1 |  |  |
| hsa-miR-320b | BDNF |  |  |
| hsa-miR-320b | SMAD3 |  |  |
| hsa-miR-320b | TXNIP |  |  |
| hsa-miR-320b | ADM |  |  |
| hsa-miR-320b | PTGS2 |  |  |
| hsa-miR-320b | UBAP1 |  |  |
| hsa-miR-320b | HSPA4 |  |  |
| hsa-miR-320b | CYCS |  |  |

|  | Top1 | Top3 | Top5 | Top10 |
| --- | --- | --- | --- | --- |
| In datasets or literature or databases | 0.909091 | 0.818182 | 0.763636 | 0.718182 |
| In literature or databases | 0.5 | 0.533333 | 0.48 | 0.4 |

| ID | Description |
| --- | --- |
| hsa04933 | AGE-RAGE signaling pathway in diabetic complications |
| hsa04066 | HIF-1 signaling pathway |
| hsa05144 | Malaria |
| hsa05323 | Rheumatoid arthritis |
| hsa04064 | NF-kappa B signaling pathway |
| hsa05418 | Fluid shear stress and atherosclerosis |
| hsa05410 | Hypertrophic cardiomyopathy |
| hsa05142 | Chagas disease |
| hsa05134 | Legionellosis |
| hsa05161 | Hepatitis B |
| hsa04659 | Th17 cell differentiation |
| hsa04668 | TNF signaling pathway |
| hsa05321 | Inflammatory bowel disease |
| hsa05167 | Kaposi sarcoma-associated herpesvirus infection |
| hsa05133 | Pertussis |
| hsa05143 | African trypanosomiasis |
| hsa05205 | Proteoglycans in cancer |
| hsa05417 | Lipid and atherosclerosis |
| hsa05163 | Human cytomegalovirus infection |
| hsa04932 | Non-alcoholic fatty liver disease |
