# Supplementary material for: A Complex Heterogeneous Network Model of Disease Regulated by Noncoding RNAs: A Case Study of Unstable Angina Pectoris
Source: Comput Intell Neurosci. 2022 Dec 23;2022:5852089. doi: 10.1155/2022/5852089 (PMC9803582; doi:10.1155/2022/5852089)
Supplement: Supplementary Materials — Table S1: Acronym explanation table. Table S2: MTP network. Table S3: Results of network analysis. Table S4: Results of network modelling. Table S5: Case studies. [file 5852089.f1.zip › MTP Network.docx]

| M | T | T1 | T2 | T | P |
| --- | --- | --- | --- | --- | --- |
| hsa-miR-20a-5p | VEGFA | ACE | CETP | ADM | hsa04270 |
| hsa-miR-101-3p | PTGS2 | ACE | SIRT1 | PARP1 | hsa04217 |
| hsa-miR-221-3p | ICAM1 | ACE | HMOX1 | PARP1 | hsa05415 |
| hsa-miR-124-3p | AHR | ACE | TIMP1 | PARP1 | hsa04210 |
| hsa-miR-124-3p | SERP1 | ACE | MMP2 | PARP1 | hsa04064 |
| hsa-miR-130a-3p | CSF1 | ACE | PLAT | ADRB2 | hsa04924 |
| hsa-miR-9-5p | NFKB1 | ACE | RETN | AGER | hsa04933 |
| hsa-miR-20b-5p | VEGFA | ACE | TGFB1 | AGER | hsa04613 |
| hsa-miR-193b-3p | PLAU | ACE | PON1 | AGER | hsa05415 |
| hsa-let-7a-5p | EDN1 | ACE | SERPINE1 | AGER | hsa05417 |
| hsa-let-7a-5p | GP5 | ACE | MPO | AGT | hsa04931 |
| hsa-let-7a-5p | ITGB3 | ACE | CCL2 | AGT | hsa04933 |
| hsa-let-7a-5p | PARP1 | ACE | IFNG | AGT | hsa05410 |
| hsa-let-7a-5p | YWHAZ | ACE | APOB | AGT | hsa04924 |
| hsa-let-7d-5p | AHR | ACE | APOA1 | AGT | hsa05414 |
| hsa-let-7d-5p | EDN1 | ACE | TNNT2 | AGT | hsa05415 |
| hsa-let-7f-5p | EDN1 | ACE | EPO | AGT | hsa04614 |
| hsa-let-7f-5p | YWHAZ | ACE | LGALS3 | AGT | hsa04270 |
| hsa-miR-20a-5p | CTSS | ACE | CRP | AGTR1 | hsa04270 |
| hsa-miR-20a-5p | F3 | ACE | VWF | AGTR1 | hsa04933 |
| hsa-miR-20a-5p | MORF4L2 | ACE | CYBA | AGTR1 | hsa04614 |
| hsa-miR-20a-5p | PPARG | ACE | ITGA2B | AGTR1 | hsa04371 |
| hsa-miR-20a-5p | TXNIP | ACE | IL1B | AGTR1 | hsa05171 |
| hsa-miR-20a-5p | UBC | ACE | SELP | AGTR1 | hsa05415 |
| hsa-miR-20a-5p | YWHAZ | ACE | ICAM1 | AGTR1 | hsa04924 |
| hsa-miR-23a-3p | CXCL8 | ACE | PPARGC1A | AHR | hsa04659 |
| hsa-miR-23a-3p | TXNIP | ACE | KNG1 | ANGPT1 | hsa04151 |
| hsa-miR-26b-5p | ADM | ACE | PIK3C2A | ANGPT1 | hsa04010 |
| hsa-miR-26b-5p | AGT | ACE | REN | ANGPT1 | hsa04014 |
| hsa-miR-26b-5p | AGTR1 | ACE | SERPING1 | ANGPT1 | hsa04015 |
| hsa-miR-26b-5p | BGLAP | ACE | PPARG | ANGPT1 | hsa05323 |
| hsa-miR-26b-5p | CAD | ACE | HMGCR | ANGPT1 | hsa04066 |
| hsa-miR-26b-5p | CCL2 | ACE | PCSK9 | ANGPT2 | hsa04014 |
| hsa-miR-26b-5p | CD28 | ACE | F7 | ANGPT2 | hsa04151 |
| hsa-miR-26b-5p | CPB2 | ACE | SMAD3 | ANGPT2 | hsa04010 |
| hsa-miR-26b-5p | CST3 | ACE | CYP2C19 | ANGPT2 | hsa05167 |
| hsa-miR-26b-5p | CXCL13 | ACE | HIF1A | ANGPT2 | hsa04066 |
| hsa-miR-26b-5p | CXCR4 | ACE | AGER | ANGPT2 | hsa04015 |
| hsa-miR-26b-5p | F3 | ACE | TLR4 | APOA1 | hsa05417 |
| hsa-miR-26b-5p | FASLG | ACE | MB | APOA1 | hsa03320 |
| hsa-miR-26b-5p | HGF | ACE | MMP1 | APOA1 | hsa05143 |
| hsa-miR-26b-5p | HMOX1 | ACE | TNNT1 | APOA1 | hsa04977 |
| hsa-miR-26b-5p | HSPA4 | ACE | THBD | APOA1 | hsa04979 |
| hsa-miR-26b-5p | HSPD1 | ACE | CXCL8 | APOB | hsa05417 |
| hsa-miR-26b-5p | IFNG | ACE | SERPINC1 | APOB | hsa04979 |
| hsa-miR-26b-5p | IL12A | ACE | MMP3 | APOB | hsa04977 |
| hsa-miR-26b-5p | ITGB2 | ACE | PECAM1 | FAS | hsa05417 |
| hsa-miR-26b-5p | MB | ACE | SELE | FAS | hsa05163 |
| hsa-miR-26b-5p | MMP8 | ACE | LCN2 | FAS | hsa05164 |
| hsa-miR-26b-5p | NFKB1 | ACE | IL10 | FAS | hsa05332 |
| hsa-miR-26b-5p | NRDC | ACE | CHIT1 | FAS | hsa04217 |
| hsa-miR-26b-5p | PAPPA | ACE | ADM | FAS | hsa04650 |
| hsa-miR-26b-5p | PRKN | ACE | AVP | FAS | hsa04010 |
| hsa-miR-26b-5p | PTGS2 | ACE | IGF1 | FAS | hsa05169 |
| hsa-miR-26b-5p | SERP1 | ACE | ADRB2 | FAS | hsa05170 |
| hsa-miR-26b-5p | TIMP1 | ACE | F3 | FAS | hsa04210 |
| hsa-miR-26b-5p | TNFRSF11A | ACE | PTGS2 | FAS | hsa05161 |
| hsa-miR-101-3p | VEGFA | ACE | CTGF | FAS | hsa05205 |
| hsa-miR-199a-5p | CCN2 | ACE | VCAM1 | FAS | hsa05142 |
| hsa-miR-199a-5p | NFKB1 | ACE | TNNI3 | FAS | hsa05143 |
| hsa-miR-204-5p | IL1B | ACE | F2 | FAS | hsa05330 |
| hsa-miR-204-5p | ITGB3 | ACE | MMP9 | FAS | hsa04932 |
| hsa-miR-204-5p | MMP9 | ACE | LEP | FAS | hsa05167 |
| hsa-miR-204-5p | PLAT | ACE | CST3 | FAS | hsa05130 |
| hsa-miR-204-5p | SERP1 | ACE | PLG | FAS | hsa04668 |
| hsa-miR-204-5p | SERPINE1 | ACE | VEGFA | FAS | hsa05160 |
| hsa-miR-204-5p | TXNIP | ACE | IL6 | FAS | hsa05165 |
| hsa-miR-218-5p | HLCS | ACE | TNF | FAS | hsa04940 |
| hsa-miR-218-5p | NAMPT | ACE | NPPB | FAS | hsa04060 |
| hsa-miR-218-5p | PPARG | ACE | DPP4 | FAS | hsa05320 |
| hsa-miR-218-5p | SEMA4D | ACE | NOS3 | FAS | hsa05168 |
| hsa-miR-221-3p | SELE | ACE | EDN1 | FAS | hsa05162 |
| hsa-miR-221-3p | UBC | ACE | INS | FASLG | hsa04210 |
| hsa-miR-222-3p | ICAM1 | ACE | AGTR1 | FASLG | hsa04014 |
| hsa-miR-222-3p | MMP1 | ACE | AGT | FASLG | hsa05160 |
| hsa-miR-222-3p | SELE | ADAMTS4 | TIMP1 | FASLG | hsa05332 |
| hsa-miR-224-5p | PTX3 | ADAMTS4 | MMP2 | FASLG | hsa04217 |
| hsa-miR-224-5p | SERPINE1 | ADAMTS4 | MMP8 | FASLG | hsa04151 |
| hsa-miR-224-5p | TXNIP | ADAMTS4 | IL37 | FASLG | hsa05170 |
| hsa-miR-124-3p | AGTR1 | ADAMTS4 | IL1A | FASLG | hsa05164 |
| hsa-miR-124-3p | BDNF | ADAMTS4 | IL1B | FASLG | hsa04010 |
| hsa-miR-124-3p | BMP6 | ADAMTS4 | MMP3 | FASLG | hsa04650 |
| hsa-miR-124-3p | CCN2 | ADAMTS4 | CXCL8 | FASLG | hsa05320 |
| hsa-miR-124-3p | CD86 | ADAMTS4 | MMP1 | FASLG | hsa05163 |
| hsa-miR-124-3p | COL6A2 | ADAMTS4 | PTGS2 | FASLG | hsa04068 |
| hsa-miR-124-3p | CXCL8 | ADAMTS4 | VEGFA | FASLG | hsa04060 |
| hsa-miR-124-3p | EDN1 | ADAMTS4 | IL6 | FASLG | hsa05205 |
| hsa-miR-124-3p | F3 | ADAMTS4 | TNF | FASLG | hsa05161 |
| hsa-miR-124-3p | GAS6 | ADAMTS4 | SERPINA1 | FASLG | hsa05417 |
| hsa-miR-124-3p | HMOX1 | ADAMTS4 | MMP9 | FASLG | hsa04940 |
| hsa-miR-124-3p | IGFBP1 | ADAMTS4 | ADAMTS7 | FASLG | hsa05142 |
| hsa-miR-124-3p | IL18R1 | ADAMTS7 | SERPING1 | FASLG | hsa05165 |
| hsa-miR-124-3p | IL6 | ADAMTS7 | COL6A2 | FASLG | hsa05130 |
| hsa-miR-124-3p | IL7 | ADAMTS7 | CTGF | FASLG | hsa05168 |
| hsa-miR-124-3p | PCSK9 | ADAMTS7 | ADAMTS4 | FASLG | hsa04932 |
| hsa-miR-124-3p | PGF | ADAMTS7 | TNF | FASLG | hsa05330 |
| hsa-miR-124-3p | PIK3C2A | ADM | SERPINE1 | FASLG | hsa05143 |
| hsa-miR-124-3p | PTGS2 | ADM | CCL2 | FASLG | hsa05162 |
| hsa-miR-124-3p | RAB3IL1 | ADM | EPO | SERPINC1 | hsa04610 |
| hsa-miR-124-3p | SERPINE1 | ADM | GDF15 | AVP | hsa04270 |
| hsa-miR-124-3p | SIRT1 | ADM | LGALS3 | BDNF | hsa04151 |
| hsa-miR-124-3p | SULT1E1 | ADM | CRP | BDNF | hsa04010 |
| hsa-miR-124-3p | TNFRSF11B | ADM | IL1B | BDNF | hsa04014 |
| hsa-miR-125b-5p | EPO | ADM | KNG1 | BMP6 | hsa04350 |
| hsa-miR-125b-5p | PARP1 | ADM | REN | BMP6 | hsa04913 |
| hsa-miR-130a-3p | EDN1 | ADM | ACE | BMP6 | hsa04060 |
| hsa-miR-130a-3p | PARP1 | ADM | NOS3 | BRCA1 | hsa04151 |
| hsa-miR-130a-3p | PPARG | ADM | IGF1 | SERPING1 | hsa04610 |
| hsa-miR-130a-3p | TNF | ADM | CXCL8 | SERPING1 | hsa05133 |
| hsa-miR-130a-3p | TXNIP | ADM | AGT | C1S | hsa05150 |
| hsa-miR-130a-3p | UBC | ADM | VIP | C1S | hsa05322 |
| hsa-miR-138-5p | HIF1A | ADM | NPPB | C1S | hsa05133 |
| hsa-miR-138-5p | MMP3 | ADM | EDN1 | C1S | hsa05171 |
| hsa-miR-138-5p | SERPINE1 | ADM | AVP | C1S | hsa04610 |
| hsa-miR-138-5p | SIRT1 | ADM | INS | CD14 | hsa05417 |
| hsa-miR-142-5p | HIF1A | ADM | CST3 | CD14 | hsa05132 |
| hsa-miR-9-5p | CXCR4 | ADM | IL6 | CD14 | hsa04145 |
| hsa-miR-9-5p | F2 | ADM | TNF | CD14 | hsa04640 |
| hsa-miR-9-5p | LPXN | ADM | ANGPT1 | CD14 | hsa05202 |
| hsa-miR-9-5p | MMP9 | ADM | HIF1A | CD14 | hsa04620 |
| hsa-miR-9-5p | SIRT1 | ADM | VEGFA | CD14 | hsa05131 |
| hsa-miR-9-5p | VEGFA | ADRB2 | IL1B | CD14 | hsa04064 |
| hsa-miR-126-5p | VEGFA | ADRB2 | PPARGC1A | CD14 | hsa05134 |
| hsa-miR-30e-5p | SERPINE1 | ADRB2 | REN | CD14 | hsa05133 |
| hsa-miR-148b-3p | ADAMTS4 | ADRB2 | PPARG | CD14 | hsa04010 |
| hsa-miR-148b-3p | CCL11 | ADRB2 | ACE | CD14 | hsa05152 |
| hsa-miR-148b-3p | CSF1 | ADRB2 | NOS3 | CD14 | hsa05146 |
| hsa-miR-148b-3p | HMOX1 | ADRB2 | CCL11 | CD28 | hsa05162 |
| hsa-miR-148b-3p | TXNIP | ADRB2 | CD14 | CD28 | hsa04672 |
| hsa-miR-20b-5p | BRCA1 | ADRB2 | CXCL8 | CD28 | hsa05332 |
| hsa-miR-20b-5p | F3 | ADRB2 | IL6 | CD28 | hsa05235 |
| hsa-miR-20b-5p | RPS27A | ADRB2 | BDNF | CD28 | hsa05322 |
| hsa-miR-20b-5p | TXNIP | ADRB2 | AVP | CD28 | hsa05416 |
| hsa-miR-20b-5p | YWHAZ | ADRB2 | LEP | CD28 | hsa05330 |
| hsa-miR-193b-3p | BRCA1 | ADRB2 | CNR1 | CD28 | hsa04660 |
| hsa-miR-193b-3p | HMGCR | ADRB2 | TNF | CD28 | hsa05323 |
| hsa-miR-193b-3p | SMAD3 | ADRB2 | AGTR1 | CD28 | hsa04940 |
| hsa-miR-193b-3p | TNFRSF1B | ADRB2 | INS | CD28 | hsa04514 |
| hsa-miR-193b-3p | YWHAZ | ADRB2 | BSG | CD28 | hsa05320 |
| hsa-miR-520c-3p | SIRT1 | ADRB2 | F2 | CD86 | hsa05330 |
| hsa-miR-505-3p | MORF4L2 | ADRB2 | EDN1 | CD86 | hsa05323 |
| hsa-miR-454-3p | EDN1 | ADRB2 | AGT | CD86 | hsa05332 |
| hsa-miR-454-3p | TXNIP | ADRB2 | CXCR4 | CD86 | hsa05167 |
| hsa-miR-454-3p | UBC | AGER | SERPINE1 | CD86 | hsa05320 |
| hsa-miR-490-5p | YWHAZ | AGER | MPO | CD86 | hsa04514 |
| hsa-miR-1297 | ADM | AGER | CCL2 | CD86 | hsa05416 |
| hsa-let-7a-5p | ACE | AGER | NFKB1 | CD86 | hsa04620 |
| hsa-let-7a-5p | ADRB2 | AGER | LGALS3 | CD86 | hsa05202 |
| hsa-let-7a-5p | AHR | AGER | CRP | CD86 | hsa05322 |
| hsa-let-7a-5p | ANGPTL2 | AGER | TLR2 | CD86 | hsa04940 |
| hsa-let-7a-5p | BMP6 | AGER | VWF | CD86 | hsa04672 |
| hsa-let-7a-5p | CAD | AGER | IL1B | ENTPD1 | hsa05169 |
| hsa-let-7a-5p | CXCL8 | AGER | ICAM1 | CD40LG | hsa04060 |
| hsa-let-7a-5p | FAS | AGER | IL18 | CD40LG | hsa04660 |
| hsa-let-7a-5p | IL18 | AGER | ACE | CD40LG | hsa04672 |
| hsa-let-7a-5p | IL6 | AGER | VCAM1 | CD40LG | hsa05310 |
| hsa-let-7a-5p | MON2 | AGER | NOS3 | CD40LG | hsa05145 |
| hsa-let-7a-5p | MYDGF | AGER | HSPA4 | CD40LG | hsa05416 |
| hsa-let-7a-5p | NFKB1 | AGER | CXCL8 | CD40LG | hsa05320 |
| hsa-let-7a-5p | PAPPA | AGER | PTGS2 | CD40LG | hsa05417 |
| hsa-let-7a-5p | PTGS2 | AGER | S100A4 | CD40LG | hsa05144 |
| hsa-let-7a-5p | PYGB | AGER | S100A8 | CD40LG | hsa05322 |
| hsa-let-7a-5p | SERP1 | AGER | S100A9 | CD40LG | hsa04514 |
| hsa-let-7a-5p | TEK | AGER | MMP9 | CD40LG | hsa04064 |
| hsa-let-7a-5p | TLR4 | AGER | TLR4 | CD40LG | hsa05330 |
| hsa-let-7a-5p | TNFRSF11A | AGER | THBD | CD63 | hsa05205 |
| hsa-let-7a-5p | UBAP1 | AGER | BDNF | CETP | hsa04979 |
| hsa-let-7a-5p | VCAM1 | AGER | CXCR4 | CNR1 | hsa04015 |
| hsa-let-7d-5p | ADRB2 | AGER | AGTR1 | COL6A2 | hsa04512 |
| hsa-let-7d-5p | BSG | AGER | IL10 | COL6A2 | hsa05165 |
| hsa-let-7d-5p | C1S | AGER | INS | COL6A2 | hsa04151 |
| hsa-let-7d-5p | CSF1 | AGER | VEGFA | COL6A2 | hsa04510 |
| hsa-let-7d-5p | CXCL8 | AGER | TNF | CPB2 | hsa04610 |
| hsa-let-7d-5p | CXCR4 | AGER | IL6 | CSF1 | hsa04380 |
| hsa-let-7d-5p | HIF1A | AGER | MYD88 | CSF1 | hsa04151 |
| hsa-let-7d-5p | MON2 | AGER | ITGB2 | CSF1 | hsa04061 |
| hsa-let-7d-5p | MYDGF | AGT | CPB2 | CSF1 | hsa04014 |
| hsa-let-7d-5p | SMAD3 | AGT | TIMP1 | CSF1 | hsa04668 |
| hsa-let-7d-5p | TLR4 | AGT | MMP2 | CSF1 | hsa05323 |
| hsa-let-7d-5p | TNC | AGT | RETN | CSF1 | hsa04010 |
| hsa-let-7d-5p | TNFRSF11A | AGT | TGFB1 | CSF1 | hsa04015 |
| hsa-let-7d-5p | VWF | AGT | NAMPT | CSF1 | hsa04060 |
| hsa-let-7d-5p | YWHAZ | AGT | CCL2 | CSF1 | hsa04640 |
| hsa-let-7f-5p | ADRB2 | AGT | APOB | CCN2 | hsa04371 |
| hsa-let-7f-5p | AHR | AGT | APOA1 | CTSS | hsa05152 |
| hsa-let-7f-5p | CXCL8 | AGT | CRP | CTSS | hsa04612 |
| hsa-let-7f-5p | FAS | AGT | CYBA | CTSS | hsa04145 |
| hsa-let-7f-5p | IL6 | AGT | IL1B | CTSS | hsa04210 |
| hsa-let-7f-5p | MON2 | AGT | CP | CX3CR1 | hsa04061 |
| hsa-let-7f-5p | MYDGF | AGT | ICAM1 | CX3CR1 | hsa04060 |
| hsa-let-7f-5p | PAPPA | AGT | KNG1 | CX3CR1 | hsa04062 |
| hsa-let-7f-5p | PARP1 | AGT | REN | CYBA | hsa04613 |
| hsa-let-7f-5p | PTGS2 | AGT | HABP2 | CYBA | hsa05140 |
| hsa-let-7f-5p | SERP1 | AGT | PPARG | CYBA | hsa05418 |
| hsa-let-7f-5p | TNFRSF11A | AGT | ACE | CYBA | hsa04145 |
| hsa-let-7f-5p | VCAM1 | AGT | VCAM1 | CYBA | hsa04670 |
| hsa-miR-20a-5p | AHR | AGT | NOS3 | CYBA | hsa05417 |
| hsa-miR-20a-5p | BSG | AGT | MMP3 | CYBA | hsa05415 |
| hsa-miR-20a-5p | C1S | AGT | CCL11 | CYBA | hsa04380 |
| hsa-miR-20a-5p | CCL2 | AGT | IGF1 | CYBA | hsa04621 |
| hsa-miR-20a-5p | CCL5 | AGT | ADRB2 | CYP2C19 | hsa00591 |
| hsa-miR-20a-5p | CCL5 | AGT | FGA | ACE | hsa05415 |
| hsa-miR-20a-5p | CCN2 | AGT | F2 | ACE | hsa05142 |
| hsa-miR-20a-5p | CD28 | AGT | PLG | ACE | hsa05410 |
| hsa-miR-20a-5p | CHI3L1 | AGT | PRG2 | ACE | hsa04924 |
| hsa-miR-20a-5p | CSF1 | AGT | LEP | ACE | hsa04614 |
| hsa-miR-20a-5p | CXCL8 | AGT | SELE | ACE | hsa05171 |
| hsa-miR-20a-5p | CYCS | AGT | SMAD3 | EDN1 | hsa05418 |
| hsa-miR-20a-5p | GDF15 | AGT | F7 | EDN1 | hsa04066 |
| hsa-miR-20a-5p | HIF1A | AGT | PTGS2 | EDN1 | hsa04668 |
| hsa-miR-20a-5p | HMOX1 | AGT | CST3 | EDN1 | hsa04924 |
| hsa-miR-20a-5p | ICAM1 | AGT | ADM | EDN1 | hsa04926 |
| hsa-miR-20a-5p | IL33 | AGT | LCN2 | EDN1 | hsa04933 |
| hsa-miR-20a-5p | IL6 | AGT | VEGFA | EDN1 | hsa04270 |
| hsa-miR-20a-5p | ITGA2 | AGT | MMP9 | EDN1 | hsa05410 |
| hsa-miR-20a-5p | LCN2 | AGT | SERPINC1 | EPO | hsa04066 |
| hsa-miR-20a-5p | LGALS3 | AGT | NPPB | EPO | hsa04630 |
| hsa-miR-20a-5p | LPXN | AGT | CTGF | EPO | hsa04151 |
| hsa-miR-20a-5p | MMP1 | AGT | TNF | EPO | hsa04640 |
| hsa-miR-20a-5p | MMP2 | AGT | INS | EPO | hsa04060 |
| hsa-miR-20a-5p | NAMPT | AGT | IL6 | F2 | hsa04810 |
| hsa-miR-20a-5p | OLR1 | AGT | AVP | F2 | hsa04610 |
| hsa-miR-20a-5p | PAPPA | AGT | CXCR4 | F2 | hsa04611 |
| hsa-miR-20a-5p | PLAU | AGT | EDN1 | F2 | hsa05130 |
| hsa-miR-20a-5p | PTGS2 | AGT | AGTR1 | F2 | hsa05171 |
| hsa-miR-20a-5p | PTX3 | AGTR1 | MMP2 | F3 | hsa04933 |
| hsa-miR-20a-5p | PYGB | AGTR1 | TGFB1 | F3 | hsa04610 |
| hsa-miR-20a-5p | RPS27A | AGTR1 | SERPINE1 | F7 | hsa04610 |
| hsa-miR-20a-5p | S100A4 | AGTR1 | CCL2 | F10 | hsa04610 |
| hsa-miR-20a-5p | S100A8 | AGTR1 | CRP | FABP3 | hsa03320 |
| hsa-miR-20a-5p | S100A9 | AGTR1 | TLR2 | FCGR2A | hsa04613 |
| hsa-miR-20a-5p | SERP1 | AGTR1 | CYBA | FCGR2A | hsa04145 |
| hsa-miR-20a-5p | SERPINA1 | AGTR1 | IL1B | FCGR2A | hsa04380 |
| hsa-miR-20a-5p | TNC | AGTR1 | ICAM1 | FCGR2A | hsa05150 |
| hsa-miR-20a-5p | TNFRSF11B | AGTR1 | KNG1 | FCGR2A | hsa05130 |
| hsa-miR-20a-5p | UBAP1 | AGTR1 | REN | FCGR2A | hsa05152 |
| hsa-miR-20a-5p | Vegfa | AGTR1 | PPARG | FCGR2A | hsa04611 |
| hsa-miR-23a-3p | AHR | AGTR1 | ACE | FCGR2A | hsa05171 |
| hsa-miR-23a-3p | FAS | AGTR1 | VCAM1 | FCGR2A | hsa05322 |
| hsa-miR-23a-3p | FSD1L | AGTR1 | NOS3 | FCGR2A | hsa05135 |
| hsa-miR-23a-3p | HIF1A | AGTR1 | IGF1 | FCGR2A | hsa05140 |
| hsa-miR-23a-3p | HMGCR | AGTR1 | ADRB2 | FGA | hsa04613 |
| hsa-miR-23a-3p | HRG | AGTR1 | CXCL8 | FGA | hsa04610 |
| hsa-miR-23a-3p | HSPD1 | AGTR1 | F2 | FGA | hsa05171 |
| hsa-miR-23a-3p | IFNG | AGTR1 | PLG | FGA | hsa04611 |
| hsa-miR-23a-3p | MORF4L2 | AGTR1 | OLR1 | GAS6 | hsa01521 |
| hsa-miR-23a-3p | MYD88 | AGTR1 | LEP | GP1BA | hsa04611 |
| hsa-miR-23a-3p | NRDC | AGTR1 | AGT | GP1BA | hsa04613 |
| hsa-miR-23a-3p | PECAM1 | AGTR1 | PTGS2 | GP1BA | hsa04512 |
| hsa-miR-23a-3p | PIK3C2A | AGTR1 | CTGF | GP1BA | hsa04640 |
| hsa-miR-23a-3p | PPARGC1A | AGTR1 | CNR1 | GP5 | hsa04640 |
| hsa-miR-23a-3p | PTX3 | AGTR1 | MMP9 | GP5 | hsa04512 |
| hsa-miR-23a-3p | PYGB | AGTR1 | AGER | GP5 | hsa04611 |
| hsa-miR-23a-3p | SELPLG | AGTR1 | EDN1 | GZMB | hsa04650 |
| hsa-miR-23a-3p | SMAD3 | AGTR1 | AVP | GZMB | hsa04940 |
| hsa-miR-23a-3p | TNFSF4 | AGTR1 | INS | GZMB | hsa05320 |
| hsa-miR-23a-3p | VCAM1 | AGTR1 | IL6 | GZMB | hsa05202 |
| hsa-miR-23a-3p | VIP | AGTR1 | TNF | GZMB | hsa05332 |
| hsa-miR-26b-5p | BRCA1 | AGTR1 | VEGFA | GZMB | hsa04210 |
| hsa-miR-26b-5p | CCN2 | AHR | HMOX1 | GZMB | hsa05330 |
| hsa-miR-26b-5p | IGF1 | AHR | IL2 | HGF | hsa04151 |
| hsa-miR-26b-5p | ITGA2 | AHR | IFNG | HGF | hsa05205 |
| hsa-miR-26b-5p | NAMPT | AHR | IL5 | HGF | hsa05144 |
| hsa-miR-26b-5p | PIK3C2A | AHR | CD28 | HGF | hsa04010 |
| hsa-miR-26b-5p | TLR4 | AHR | ENTPD1 | HGF | hsa04510 |
| hsa-miR-26b-5p | YWHAZ | AHR | IL18 | HGF | hsa01521 |
| hsa-miR-101-3p | ADM | AHR | IL33 | HGF | hsa04014 |
| hsa-miR-101-3p | ADRB2 | AHR | TLR4 | HGF | hsa04015 |
| hsa-miR-101-3p | ANGPT1 | AHR | CYP2C19 | HIF1A | hsa05235 |
| hsa-miR-101-3p | ANGPT2 | AHR | HIF1A | HIF1A | hsa04659 |
| hsa-miR-101-3p | CCL2 | AHR | BRCA1 | HIF1A | hsa05205 |
| hsa-miR-101-3p | CD86 | AHR | VEGFA | HIF1A | hsa05167 |
| hsa-miR-101-3p | CHGA | AHR | INS | HIF1A | hsa04137 |
| hsa-miR-101-3p | CHI3L1 | AHR | SMAD3 | HIF1A | hsa04066 |
| hsa-miR-101-3p | CSF1 | AHR | PTGS2 | HLA-DRB1 | hsa05152 |
| hsa-miR-101-3p | CXCL8 | AHR | CXCL8 | HLA-DRB1 | hsa05140 |
| hsa-miR-101-3p | EDN1 | AHR | PPARG | HLA-DRB1 | hsa04672 |
| hsa-miR-101-3p | FAS | AHR | IL10 | HLA-DRB1 | hsa04659 |
| hsa-miR-101-3p | FGF21 | AHR | IL1B | HLA-DRB1 | hsa05310 |
| hsa-miR-101-3p | FST | AHR | IL6 | HLA-DRB1 | hsa05320 |
| hsa-miR-101-3p | HMGCR | AHR | IL17A | HLA-DRB1 | hsa05330 |
| hsa-miR-101-3p | HMOX1 | AHR | TNF | HLA-DRB1 | hsa05332 |
| hsa-miR-101-3p | HSPD1 | ANGPT1 | TIMP1 | HLA-DRB1 | hsa04145 |
| hsa-miR-101-3p | IL12A | ANGPT1 | MMP2 | HLA-DRB1 | hsa05145 |
| hsa-miR-101-3p | IL1B | ANGPT1 | TGFB1 | HLA-DRB1 | hsa05164 |
| hsa-miR-101-3p | ITGA2 | ANGPT1 | HGF | HLA-DRB1 | hsa04640 |
| hsa-miR-101-3p | ITGB3 | ANGPT1 | SERPINE1 | HLA-DRB1 | hsa04514 |
| hsa-miR-101-3p | ITIH4 | ANGPT1 | CCL2 | HLA-DRB1 | hsa04940 |
| hsa-miR-101-3p | MLKL | ANGPT1 | NFKB1 | HLA-DRB1 | hsa05166 |
| hsa-miR-101-3p | MMP1 | ANGPT1 | EPO | HLA-DRB1 | hsa05323 |
| hsa-miR-101-3p | MMP3 | ANGPT1 | VWF | HLA-DRB1 | hsa05169 |
| hsa-miR-101-3p | MMP9 | ANGPT1 | IL1B | HLA-DRB1 | hsa04658 |
| hsa-miR-101-3p | MON2 | ANGPT1 | SELP | HLA-DRB1 | hsa05322 |
| hsa-miR-101-3p | MORF4L2 | ANGPT1 | ICAM1 | HLA-DRB1 | hsa05321 |
| hsa-miR-101-3p | NPPB | ANGPT1 | KNG1 | HLA-DRB1 | hsa05416 |
| hsa-miR-101-3p | PAPPA | ANGPT1 | VCAM1 | HLA-DRB1 | hsa05150 |
| hsa-miR-101-3p | PLAT | ANGPT1 | PF4 | HLA-DRB1 | hsa04612 |
| hsa-miR-101-3p | PLAU | ANGPT1 | NOS3 | HLA-DRB1 | hsa05168 |
| hsa-miR-101-3p | PPARGC1A | ANGPT1 | IGF1 | HMGCR | hsa04152 |
| hsa-miR-101-3p | PTX3 | ANGPT1 | CXCL8 | HMOX1 | hsa05418 |
| hsa-miR-101-3p | S100A9 | ANGPT1 | PLG | HMOX1 | hsa04066 |
| hsa-miR-101-3p | SAA4 | ANGPT1 | ANGPT2 | HSPA4 | hsa04612 |
| hsa-miR-101-3p | SERP1 | ANGPT1 | MMP1 | HSPA4 | hsa05417 |
| hsa-miR-101-3p | SERPINE1 | ANGPT1 | SELE | HSPD1 | hsa05134 |
| hsa-miR-101-3p | TAF1 | ANGPT1 | SMAD3 | HSPD1 | hsa04940 |
| hsa-miR-101-3p | THBD | ANGPT1 | MMP9 | HSPD1 | hsa05152 |
| hsa-miR-101-3p | THBS4 | ANGPT1 | THBD | HSPD1 | hsa05417 |
| hsa-miR-101-3p | TNFSF4 | ANGPT1 | EDN1 | TNC | hsa04151 |
| hsa-miR-101-3p | TNNT2 | ANGPT1 | TEK | TNC | hsa04512 |
| hsa-miR-101-3p | TXNIP | ANGPT1 | INS | TNC | hsa04510 |
| hsa-miR-101-3p | YWHAZ | ANGPT1 | IL6 | TNC | hsa05165 |
| hsa-miR-199a-5p | CAD | ANGPT1 | CXCR4 | ICAM1 | hsa05416 |
| hsa-miR-199a-5p | CCL2 | ANGPT1 | TNF | ICAM1 | hsa04064 |
| hsa-miR-199a-5p | EDN1 | ANGPT1 | IL10 | ICAM1 | hsa04933 |
| hsa-miR-199a-5p | HIF1A | ANGPT1 | BDNF | ICAM1 | hsa04514 |
| hsa-miR-199a-5p | MMP2 | ANGPT1 | ADM | ICAM1 | hsa05417 |
| hsa-miR-199a-5p | MMP3 | ANGPT1 | FGF21 | ICAM1 | hsa05169 |
| hsa-miR-199a-5p | PARP1 | ANGPT1 | HIF1A | ICAM1 | hsa05323 |
| hsa-miR-199a-5p | PLAU | ANGPT1 | PGF | ICAM1 | hsa04650 |
| hsa-miR-199a-5p | PPARG | ANGPT1 | PECAM1 | ICAM1 | hsa05166 |
| hsa-miR-199a-5p | PTGS2 | ANGPT1 | VEGFA | ICAM1 | hsa05164 |
| hsa-miR-199a-5p | SIRT1 | ANGPT2 | TIMP1 | ICAM1 | hsa04670 |
| hsa-miR-199a-5p | SMAD3 | ANGPT2 | MMP2 | ICAM1 | hsa05144 |
| hsa-miR-199a-5p | SULT1E1 | ANGPT2 | HGF | ICAM1 | hsa05143 |
| hsa-miR-199a-5p | TGFB1 | ANGPT2 | SERPINE1 | ICAM1 | hsa04668 |
| hsa-miR-199a-5p | VEGFA | ANGPT2 | CCL2 | ICAM1 | hsa05418 |
| hsa-miR-30d-5p | AHR | ANGPT2 | EPO | ICAM1 | hsa05150 |
| hsa-miR-30d-5p | BDNF | ANGPT2 | CRP | ICAM1 | hsa05167 |
| hsa-miR-30d-5p | CCL5 | ANGPT2 | FST | IFNA1 | hsa04217 |
| hsa-miR-30d-5p | CCN2 | ANGPT2 | VWF | IFNA1 | hsa04630 |
| hsa-miR-30d-5p | CHGA | ANGPT2 | IL1B | IFNA1 | hsa05170 |
| hsa-miR-30d-5p | CXCL8 | ANGPT2 | SELP | IFNA1 | hsa04151 |
| hsa-miR-30d-5p | ENTPD1 | ANGPT2 | ICAM1 | IFNA1 | hsa05169 |
| hsa-miR-30d-5p | FAS | ANGPT2 | VCAM1 | IFNA1 | hsa04650 |
| hsa-miR-30d-5p | FERMT3 | ANGPT2 | NOS3 | IFNA1 | hsa05417 |
| hsa-miR-30d-5p | FST | ANGPT2 | IGF1 | IFNA1 | hsa05160 |
| hsa-miR-30d-5p | HDAC9 | ANGPT2 | CXCL8 | IFNA1 | hsa05164 |
| hsa-miR-30d-5p | HMOX1 | ANGPT2 | PLG | IFNA1 | hsa04623 |
| hsa-miR-30d-5p | IL10 | ANGPT2 | LEP | IFNA1 | hsa05167 |
| hsa-miR-30d-5p | IL1A | ANGPT2 | MMP1 | IFNA1 | hsa04622 |
| hsa-miR-30d-5p | INTS2 | ANGPT2 | F3 | IFNA1 | hsa05171 |
| hsa-miR-30d-5p | MB | ANGPT2 | IL10 | IFNA1 | hsa05165 |
| hsa-miR-30d-5p | MMP1 | ANGPT2 | ITGB2 | IFNA1 | hsa05162 |
| hsa-miR-30d-5p | MON2 | ANGPT2 | CXCR4 | IFNA1 | hsa05152 |
| hsa-miR-30d-5p | MYDGF | ANGPT2 | THBD | IFNA1 | hsa05320 |
| hsa-miR-30d-5p | PARP1 | ANGPT2 | EDN1 | IFNA1 | hsa04060 |
| hsa-miR-30d-5p | PLAT | ANGPT2 | IL6 | IFNA1 | hsa04620 |
| hsa-miR-30d-5p | PPARGC1A | ANGPT2 | MMP9 | IFNA1 | hsa05168 |
| hsa-miR-30d-5p | SERPINC1 | ANGPT2 | SELE | IFNA1 | hsa04621 |
| hsa-miR-30d-5p | SERPINE1 | ANGPT2 | TNF | IFNA1 | hsa05161 |
| hsa-miR-30d-5p | TNC | ANGPT2 | HIF1A | IFNA1 | hsa05163 |
| hsa-miR-30d-5p | TXNIP | ANGPT2 | PECAM1 | IFNG | hsa05142 |
| hsa-miR-30d-5p | VEGFA | ANGPT2 | PGF | IFNG | hsa05235 |
| hsa-miR-30d-5p | YWHAZ | ANGPT2 | ANGPT1 | IFNG | hsa04380 |
| hsa-miR-204-5p | ANGPTL2 | ANGPT2 | VEGFA | IFNG | hsa04650 |
| hsa-miR-204-5p | BDNF | ANGPT2 | TEK | IFNG | hsa04350 |
| hsa-miR-204-5p | CD28 | ANGPTL2 | LEP | IFNG | hsa04060 |
| hsa-miR-204-5p | CXCL8 | ANGPTL2 | TEK | IFNG | hsa05332 |
| hsa-miR-204-5p | CXCR4 | APCS | HRG | IFNG | hsa05164 |
| hsa-miR-204-5p | HLA-DRB1 | APCS | APOA1 | IFNG | hsa05145 |
| hsa-miR-204-5p | HLA-DRB1 | APCS | CRP | IFNG | hsa05160 |
| hsa-miR-204-5p | HLA-DRB1 | APCS | C1S | IFNG | hsa04659 |
| hsa-miR-204-5p | HLA-DRB1 | APCS | TNF | IFNG | hsa04066 |
| hsa-miR-204-5p | HLA-DRB1 | APCS | IL1B | IFNG | hsa05146 |
| hsa-miR-204-5p | HLA-DRB1 | APCS | CP | IFNG | hsa05323 |
| hsa-miR-204-5p | HLCS | APCS | IL6 | IFNG | hsa04660 |
| hsa-miR-204-5p | MALAT1 | APCS | SERPINC1 | IFNG | hsa04658 |
| hsa-miR-204-5p | MMP3 | APCS | SERPINA1 | IFNG | hsa05140 |
| hsa-miR-204-5p | PLB1 | APCS | FGA | IFNG | hsa05143 |
| hsa-miR-204-5p | PTGS2 | APCS | ITIH4 | IFNG | hsa05418 |
| hsa-miR-204-5p | SERPINC1 | APCS | SAA4 | IFNG | hsa05168 |
| hsa-miR-204-5p | SERPINF2 | APCS | MBL2 | IFNG | hsa04630 |
| hsa-miR-204-5p | SERPINF2 | APCS | FCGR2A | IFNG | hsa05152 |
| hsa-miR-204-5p | SIRT1 | APOA1 | CETP | IFNG | hsa05321 |
| hsa-miR-218-5p | AHR | APOA1 | RETN | IFNG | hsa04940 |
| hsa-miR-218-5p | BRCA1 | APOA1 | PON1 | IFNG | hsa04217 |
| hsa-miR-218-5p | CCN2 | APOA1 | SERPINE1 | IFNG | hsa05330 |
| hsa-miR-218-5p | HIF1A | APOA1 | MPO | IFNG | hsa05144 |
| hsa-miR-218-5p | HMOX1 | APOA1 | CCL2 | IFNG | hsa05322 |
| hsa-miR-218-5p | MMP1 | APOA1 | HRG | IFNG | hsa04657 |
| hsa-miR-218-5p | MMP2 | APOA1 | APOB | IFNG | hsa04612 |
| hsa-miR-218-5p | MMP9 | APOA1 | IGF1 | IGF1 | hsa05205 |
| hsa-miR-218-5p | MORF4L2 | APOA1 | MMP9 | IGF1 | hsa04913 |
| hsa-miR-218-5p | MYD88 | APOA1 | IL10 | IGF1 | hsa05202 |
| hsa-miR-218-5p | PIK3C2A | APOA1 | SELP | IGF1 | hsa04014 |
| hsa-miR-218-5p | SERP1 | APOA1 | REN | IGF1 | hsa04151 |
| hsa-miR-218-5p | SERPINE1 | APOA1 | CXCL8 | IGF1 | hsa04010 |
| hsa-miR-218-5p | TIMP1 | APOA1 | F3 | IGF1 | hsa04066 |
| hsa-miR-218-5p | TNC | APOA1 | CD40LG | IGF1 | hsa04015 |
| hsa-miR-218-5p | UBAP1 | APOA1 | PLA2G2A | IGF1 | hsa01521 |
| hsa-miR-218-5p | VEGFA | APOA1 | FABP3 | IGF1 | hsa04152 |
| hsa-miR-218-5p | YWHAZ | APOA1 | MBL2 | IGF1 | hsa04211 |
| hsa-miR-221-3p | CAD | APOA1 | SELE | IGF1 | hsa04510 |
| hsa-miR-221-3p | CCL5 | APOA1 | VWF | IGF1 | hsa05215 |
| hsa-miR-221-3p | CNR1 | APOA1 | CXCL16 | IGF1 | hsa04068 |
| hsa-miR-221-3p | CXCL8 | APOA1 | SERPINA5 | IGF1 | hsa05414 |
| hsa-miR-221-3p | ENTPD1 | APOA1 | IL1B | IGF1 | hsa05410 |
| hsa-miR-221-3p | FASLG | APOA1 | OLR1 | IL1A | hsa05133 |
| hsa-miR-221-3p | FERMT3 | APOA1 | ICAM1 | IL1A | hsa04933 |
| hsa-miR-221-3p | HMOX1 | APOA1 | LPXN | IL1A | hsa04940 |
| hsa-miR-221-3p | IL6 | APOA1 | ACE | IL1A | hsa05332 |
| hsa-miR-221-3p | MMP2 | APOA1 | KNG1 | IL1A | hsa04060 |
| hsa-miR-221-3p | SERP1 | APOA1 | ITIH4 | IL1A | hsa05152 |
| hsa-miR-221-3p | SIRT1 | APOA1 | F7 | IL1A | hsa05162 |
| hsa-miR-222-3p | ENTPD1 | APOA1 | AGT | IL1A | hsa05164 |
| hsa-miR-223-3p | CCL3 | APOA1 | APCS | IL1A | hsa05323 |
| hsa-miR-223-3p | CCL3 | APOA1 | TLR4 | IL1A | hsa04218 |
| hsa-miR-223-3p | CCL3 | APOA1 | TNF | IL1A | hsa04217 |
| hsa-miR-223-3p | ENTPD1 | APOA1 | LEP | IL1A | hsa04640 |
| hsa-miR-223-3p | F3 | APOA1 | IL6 | IL1A | hsa05140 |
| hsa-miR-223-3p | IL6 | APOA1 | VCAM1 | IL1A | hsa04010 |
| hsa-miR-223-3p | MMP2 | APOA1 | SERPINF2 | IL1A | hsa04380 |
| hsa-miR-223-3p | MMP9 | APOA1 | HMGCR | IL1A | hsa05418 |
| hsa-miR-223-3p | NAMPT | APOA1 | CST3 | IL1A | hsa05321 |
| hsa-miR-223-3p | PARP1 | APOA1 | IGFBP1 | IL1A | hsa04932 |
| hsa-miR-223-3p | VEGFA | APOA1 | PPARG | IL1B | hsa05132 |
| hsa-miR-224-5p | BSG | APOA1 | CP | IL1B | hsa04933 |
| hsa-miR-224-5p | CCN2 | APOA1 | PCSK9 | IL1B | hsa04010 |
| hsa-miR-224-5p | CXCR4 | APOA1 | IFNA1 | IL1B | hsa05140 |
| hsa-miR-224-5p | EDN1 | APOA1 | VIMP | IL1B | hsa04932 |
| hsa-miR-224-5p | F3 | APOA1 | NOS3 | IL1B | hsa05171 |
| hsa-miR-224-5p | GAS6 | APOA1 | PLG | IL1B | hsa01523 |
| hsa-miR-224-5p | IL18 | APOA1 | CRP | IL1B | hsa04064 |
| hsa-miR-224-5p | ITGA2 | APOA1 | SERPINA1 | IL1B | hsa05143 |
| hsa-miR-224-5p | LGALS3 | APOA1 | SAA4 | IL1B | hsa04217 |
| hsa-miR-224-5p | MMP1 | APOA1 | SERPINC1 | IL1B | hsa04659 |
| hsa-miR-224-5p | MORF4L2 | APOA1 | INS | IL1B | hsa04657 |
| hsa-miR-224-5p | PARP1 | APOA1 | F2 | IL1B | hsa04621 |
| hsa-miR-224-5p | PIK3C2A | APOA1 | PPARGC1A | IL1B | hsa04640 |
| hsa-miR-224-5p | PLAT | APOA1 | FGA | IL1B | hsa04625 |
| hsa-miR-224-5p | PYGB | APOB | CPB2 | IL1B | hsa05142 |
| hsa-miR-224-5p | RIPK1 | APOB | CETP | IL1B | hsa05144 |
| hsa-miR-224-5p | SERP1 | APOB | RETN | IL1B | hsa05152 |
| hsa-miR-224-5p | SERPINF2 | APOB | PON1 | IL1B | hsa05162 |
| hsa-miR-224-5p | SERPINF2 | APOB | SERPINE1 | IL1B | hsa05130 |
| hsa-miR-224-5p | TNC | APOB | MPO | IL1B | hsa05135 |
| hsa-miR-224-5p | YWHAZ | APOB | CCL2 | IL1B | hsa05323 |
| hsa-miR-124-3p | ACE | APOB | HRG | IL1B | hsa05418 |
| hsa-miR-124-3p | ADM | APOB | CXCL8 | IL1B | hsa05164 |
| hsa-miR-124-3p | APOB | APOB | BRCA1 | IL1B | hsa05131 |
| hsa-miR-124-3p | BRCA1 | APOB | VEGFA | IL1B | hsa05133 |
| hsa-miR-124-3p | BSG | APOB | TLR2 | IL1B | hsa04060 |
| hsa-miR-124-3p | C1S | APOB | IL10 | IL1B | hsa05417 |
| hsa-miR-124-3p | CCL2 | APOB | IGF1 | IL1B | hsa05321 |
| hsa-miR-124-3p | CD14 | APOB | SELP | IL1B | hsa05146 |
| hsa-miR-124-3p | CETP | APOB | KNG1 | IL1B | hsa04380 |
| hsa-miR-124-3p | CHI3L1 | APOB | CST3 | IL1B | hsa04940 |
| hsa-miR-124-3p | CP | APOB | PLA2G2A | IL1B | hsa04668 |
| hsa-miR-124-3p | CPB2 | APOB | FGF21 | IL1B | hsa04623 |
| hsa-miR-124-3p | CSF1 | APOB | DPP4 | IL1B | hsa05332 |
| hsa-miR-124-3p | CX3CL1 | APOB | PPARGC1A | IL1B | hsa04620 |
| hsa-miR-124-3p | CXCL16 | APOB | IL1B | IL1B | hsa05134 |
| hsa-miR-124-3p | CYCS | APOB | SELE | IL1B | hsa05168 |
| hsa-miR-124-3p | DPP4 | APOB | TXNIP | IL1B | hsa05163 |
| hsa-miR-124-3p | F10 | APOB | SERPINF2 | IL1RN | hsa04060 |
| hsa-miR-124-3p | FAS | APOB | F3 | IL2 | hsa04660 |
| hsa-miR-124-3p | FGA | APOB | VWF | IL2 | hsa04061 |
| hsa-miR-124-3p | FST | APOB | NOS3 | IL2 | hsa05135 |
| hsa-miR-124-3p | GDF15 | APOB | ICAM1 | IL2 | hsa04672 |
| hsa-miR-124-3p | HABP2 | APOB | AGT | IL2 | hsa05162 |
| hsa-miR-124-3p | HIF1A | APOB | CP | IL2 | hsa05321 |
| hsa-miR-124-3p | HSPA4 | APOB | REN | IL2 | hsa04940 |
| hsa-miR-124-3p | ICAM1 | APOB | VCAM1 | IL2 | hsa04630 |
| hsa-miR-124-3p | IL18 | APOB | TNF | IL2 | hsa04659 |
| hsa-miR-124-3p | IL1RN | APOB | TYR | IL2 | hsa05171 |
| hsa-miR-124-3p | IL32 | APOB | MBL2 | IL2 | hsa04060 |
| hsa-miR-124-3p | ITGA2 | APOB | ACE | IL2 | hsa05320 |
| hsa-miR-124-3p | ITGB3 | APOB | PPARG | IL2 | hsa04625 |
| hsa-miR-124-3p | KNG1 | APOB | IL6 | IL2 | hsa05330 |
| hsa-miR-124-3p | LGALS3 | APOB | SERPINA1 | IL2 | hsa04151 |
| hsa-miR-124-3p | MB | APOB | F7 | IL2 | hsa05332 |
| hsa-miR-124-3p | MBL2 | APOB | LEP | IL2 | hsa04658 |
| hsa-miR-124-3p | MLKL | APOB | HSPA4 | IL2 | hsa05166 |
| hsa-miR-124-3p | MMP2 | APOB | LPXN | IL2 | hsa05142 |
| hsa-miR-124-3p | MMP3 | APOB | HMGCR | IL2RA | hsa04640 |
| hsa-miR-124-3p | MMP9 | APOB | SERPINC1 | IL2RA | hsa04060 |
| hsa-miR-124-3p | MORF4L2 | APOB | IGFBP1 | IL2RA | hsa05166 |
| hsa-miR-124-3p | MYD88 | APOB | SAA4 | IL2RA | hsa04061 |
| hsa-miR-124-3p | MYDGF | APOB | TLR4 | IL2RA | hsa04659 |
| hsa-miR-124-3p | NAMPT | APOB | FGA | IL2RA | hsa04658 |
| hsa-miR-124-3p | NFKB1 | APOB | VIMP | IL2RA | hsa04630 |
| hsa-miR-124-3p | NOS3 | APOB | F2 | IL2RA | hsa05162 |
| hsa-miR-124-3p | NPPB | APOB | INS | IL2RA | hsa04151 |
| hsa-miR-124-3p | PARP1 | APOB | ITGB2 | IL2RB | hsa04630 |
| hsa-miR-124-3p | PLA2G2A | APOB | PLG | IL2RB | hsa04060 |
| hsa-miR-124-3p | PLAT | APOB | CRP | IL2RB | hsa04151 |
| hsa-miR-124-3p | PPARGC1A | APOB | PECAM1 | IL2RB | hsa04061 |
| hsa-miR-124-3p | S100A4 | APOB | OLR1 | IL2RB | hsa04658 |
| hsa-miR-124-3p | SELENOS | APOB | PCSK9 | IL2RB | hsa05162 |
| hsa-miR-124-3p | SERPINC1 | APOB | APOA1 | IL2RB | hsa05166 |
| hsa-miR-124-3p | SERPING1 | AVP | TNNT2 | IL2RB | hsa04659 |
| hsa-miR-124-3p | SMAD3 | AVP | CRP | IL2RB | hsa05202 |
| hsa-miR-124-3p | TGFB1 | AVP | IL1B | IL5 | hsa04630 |
| hsa-miR-124-3p | TIMP1 | AVP | KNG1 | IL5 | hsa05330 |
| hsa-miR-124-3p | TNFSF4 | AVP | REN | IL5 | hsa04060 |
| hsa-miR-124-3p | TNNI3 | AVP | ACE | IL5 | hsa04657 |
| hsa-miR-124-3p | TXNIP | AVP | ADRB2 | IL5 | hsa04658 |
| hsa-miR-124-3p | UBAP1 | AVP | F2 | IL5 | hsa05320 |
| hsa-miR-124-3p | UBC | AVP | TNNI3 | IL5 | hsa04672 |
| hsa-miR-124-3p | VEGFA | AVP | AGT | IL5 | hsa04640 |
| hsa-miR-125b-5p | ADAMTS4 | AVP | VIP | IL5 | hsa05321 |
| hsa-miR-125b-5p | ADM | AVP | LCN2 | IL5 | hsa05310 |
| hsa-miR-125b-5p | ANGPT2 | AVP | FABP3 | IL5 | hsa04660 |
| hsa-miR-125b-5p | BSG | AVP | NPPB | IL6 | hsa05132 |
| hsa-miR-125b-5p | EDN1 | AVP | EDN1 | IL6 | hsa04931 |
| hsa-miR-125b-5p | Fas | AVP | MB | IL6 | hsa04621 |
| hsa-miR-125b-5p | HMGCR | AVP | IL6 | IL6 | hsa05168 |
| hsa-miR-125b-5p | HSPD1 | AVP | CST3 | IL6 | hsa05161 |
| hsa-miR-125b-5p | ICAM1 | AVP | INS | IL6 | hsa05133 |
| hsa-miR-125b-5p | IL1RN | AVP | AGTR1 | IL6 | hsa05169 |
| hsa-miR-125b-5p | IL6 | AVP | ADM | IL6 | hsa04932 |
| hsa-miR-125b-5p | ITGB3 | BDNF | CX3CL1 | IL6 | hsa04068 |
| hsa-miR-125b-5p | MMP2 | BDNF | TNFRSF1A | IL6 | hsa05163 |
| hsa-miR-125b-5p | MORF4L2 | BDNF | SIRT1 | IL6 | hsa04218 |
| hsa-miR-125b-5p | NRDC | BDNF | HMOX1 | IL6 | hsa04625 |
| hsa-miR-125b-5p | S100A8 | BDNF | MMP2 | IL6 | hsa05162 |
| hsa-miR-125b-5p | SELE | BDNF | PLAT | IL6 | hsa04672 |
| hsa-miR-125b-5p | SEMA4D | BDNF | TGFB1 | IL6 | hsa04630 |
| hsa-miR-125b-5p | SERPINE1 | BDNF | HGF | IL6 | hsa04659 |
| hsa-miR-125b-5p | TNF | BDNF | SERPINE1 | IL6 | hsa05130 |
| hsa-miR-125b-5p | TNF | BDNF | CCL2 | IL6 | hsa01523 |
| hsa-miR-125b-5p | TNF | BDNF | IL2 | IL6 | hsa05135 |
| hsa-miR-125b-5p | TNF | BDNF | IFNG | IL6 | hsa05166 |
| hsa-miR-125b-5p | TNF | BDNF | EPO | IL6 | hsa04623 |
| hsa-miR-125b-5p | TNF | BDNF | CRP | IL6 | hsa05202 |
| hsa-miR-125b-5p | TNF | BDNF | IL1A | IL6 | hsa05410 |
| hsa-miR-125b-5p | TNF | BDNF | IL1B | IL6 | hsa05167 |
| hsa-miR-125b-5p | TNFRSF1B | BDNF | ICAM1 | IL6 | hsa04620 |
| hsa-miR-125b-5p | TXNIP | BDNF | PPARGC1A | IL6 | hsa01521 |
| hsa-miR-125b-5p | VEGFA | BDNF | ITIH4 | IL6 | hsa05146 |
| hsa-miR-130a-3p | ARSA | BDNF | IL18 | IL6 | hsa04060 |
| hsa-miR-130a-3p | BMP6 | BDNF | PPARG | IL6 | hsa05417 |
| hsa-miR-130a-3p | CCL5 | BDNF | NOS3 | IL6 | hsa05152 |
| hsa-miR-130a-3p | CXCL8 | BDNF | MMP3 | IL6 | hsa05142 |
| hsa-miR-130a-3p | CXCR4 | BDNF | IGF1 | IL6 | hsa05323 |
| hsa-miR-130a-3p | FAS | BDNF | HSPA4 | IL6 | hsa04151 |
| hsa-miR-130a-3p | HIF1A | BDNF | ADRB2 | IL6 | hsa04066 |
| hsa-miR-130a-3p | HMGCR | BDNF | CXCL8 | IL6 | hsa05143 |
| hsa-miR-130a-3p | HMOX1 | BDNF | CYCS | IL6 | hsa05144 |
| hsa-miR-130a-3p | IL18 | BDNF | PLG | IL6 | hsa05164 |
| hsa-miR-130a-3p | LPXN | BDNF | LEP | IL6 | hsa05321 |
| hsa-miR-130a-3p | NFKB1 | BDNF | IL17A | IL6 | hsa05134 |
| hsa-miR-130a-3p | PECAM1 | BDNF | CX3CR1 | IL6 | hsa04933 |
| hsa-miR-130a-3p | PPARGC1A | BDNF | PARK2 | IL6 | hsa05332 |
| hsa-miR-130a-3p | RPS27A | BDNF | VIP | IL6 | hsa04668 |
| hsa-miR-130a-3p | TGFB1 | BDNF | PTGS2 | IL6 | hsa04061 |
| hsa-miR-130a-3p | TNF | BDNF | CNR1 | IL6 | hsa04640 |
| hsa-miR-130a-3p | TNF | BDNF | MMP9 | IL6 | hsa04657 |
| hsa-miR-130a-3p | TNF | BDNF | TLR4 | IL6 | hsa05171 |
| hsa-miR-130a-3p | TNF | BDNF | AGER | IL7 | hsa04060 |
| hsa-miR-130a-3p | TNF | BDNF | TEK | IL7 | hsa04630 |
| hsa-miR-130a-3p | TNF | BDNF | INS | IL7 | hsa04151 |
| hsa-miR-130a-3p | TNF | BDNF | IL6 | IL7 | hsa04640 |
| hsa-miR-130a-3p | TNFRSF1B | BDNF | CXCR4 | CXCL8 | hsa04621 |
| hsa-miR-138-5p | CCN2 | BDNF | TNF | CXCL8 | hsa05130 |
| hsa-miR-138-5p | CXCL8 | BDNF | IL10 | CXCL8 | hsa05219 |
| hsa-miR-138-5p | DPP4 | BDNF | CCL3 | CXCL8 | hsa05131 |
| hsa-miR-138-5p | F3 | BDNF | ANGPT1 | CXCL8 | hsa05417 |
| hsa-miR-138-5p | FST | BDNF | CCL5 | CXCL8 | hsa05164 |
| hsa-miR-138-5p | ICAM1 | BDNF | HIF1A | CXCL8 | hsa04657 |
| hsa-miR-138-5p | IL1A | BDNF | SEMA4D | CXCL8 | hsa04062 |
| hsa-miR-138-5p | IL1RN | BDNF | FGF21 | CXCL8 | hsa05142 |
| hsa-miR-138-5p | IL6 | BDNF | VEGFA | CXCL8 | hsa05132 |
| hsa-miR-138-5p | ITGA2 | BGLAP | MMP2 | CXCL8 | hsa04064 |
| hsa-miR-138-5p | LCN2 | BGLAP | RETN | CXCL8 | hsa05171 |
| hsa-miR-138-5p | MMP9 | BGLAP | TGFB1 | CXCL8 | hsa05163 |
| hsa-miR-138-5p | NFKB1 | BGLAP | NAMPT | CXCL8 | hsa05161 |
| hsa-miR-138-5p | NRDC | BGLAP | CRP | CXCL8 | hsa05146 |
| hsa-miR-138-5p | PLAU | BGLAP | VWF | CXCL8 | hsa05135 |
| hsa-miR-138-5p | PPARG | BGLAP | IL1B | CXCL8 | hsa04933 |
| hsa-miR-138-5p | PTGS2 | BGLAP | BMP6 | CXCL8 | hsa05202 |
| hsa-miR-138-5p | SELENOS | BGLAP | PPARG | CXCL8 | hsa04061 |
| hsa-miR-138-5p | SEMA4D | BGLAP | VCAM1 | CXCL8 | hsa05144 |
| hsa-miR-138-5p | SMAD3 | BGLAP | TNFRSF11B | CXCL8 | hsa04218 |
| hsa-miR-138-5p | TGFB1 | BGLAP | MMP3 | CXCL8 | hsa04932 |
| hsa-miR-138-5p | TNC | BGLAP | IGF1 | CXCL8 | hsa05323 |
| hsa-miR-138-5p | TXNIP | BGLAP | CXCL8 | CXCL8 | hsa05167 |
| hsa-miR-138-5p | UBC | BGLAP | F2 | CXCL8 | hsa05134 |
| hsa-miR-138-5p | VEGFA | BGLAP | LEP | CXCL8 | hsa05133 |
| hsa-miR-142-5p | NAMPT | BGLAP | CSF1 | CXCL8 | hsa04620 |
| hsa-miR-142-5p | NRDC | BGLAP | GAS6 | CXCL8 | hsa04060 |
| hsa-miR-142-5p | PIK3C2A | BGLAP | PTGS2 | CXCL8 | hsa04622 |
| hsa-miR-142-5p | SIRT1 | BGLAP | HIF1A | IL10 | hsa04672 |
| hsa-miR-142-5p | SMAD3 | BGLAP | FGF21 | IL10 | hsa05320 |
| hsa-miR-9-5p | BDNF | BGLAP | TNFRSF11A | IL10 | hsa05140 |
| hsa-miR-9-5p | CCL5 | BGLAP | MMP9 | IL10 | hsa05330 |
| hsa-miR-9-5p | CXCL8 | BGLAP | PECAM1 | IL10 | hsa05145 |
| hsa-miR-9-5p | CYCS | BGLAP | TNF | IL10 | hsa05310 |
| hsa-miR-9-5p | FAS | BGLAP | IL6 | IL10 | hsa05143 |
| hsa-miR-9-5p | GDF15 | BGLAP | VEGFA | IL10 | hsa05133 |
| hsa-miR-9-5p | HIF1A | BGLAP | INS | IL10 | hsa05142 |
| hsa-miR-9-5p | HMGCR | BMP6 | EPO | IL10 | hsa05150 |
| hsa-miR-9-5p | HSPD1 | BMP6 | FST | IL10 | hsa05321 |
| hsa-miR-9-5p | IL5 | BMP6 | CTGF | IL10 | hsa05135 |
| hsa-miR-9-5p | IL6 | BMP6 | INS | IL10 | hsa04630 |
| hsa-miR-9-5p | ITGA2 | BMP6 | TNFRSF11B | IL10 | hsa04060 |
| hsa-miR-9-5p | MALAT1 | BMP6 | MMP9 | IL10 | hsa04625 |
| hsa-miR-9-5p | MMP2 | BMP6 | IL6 | IL10 | hsa05146 |
| hsa-miR-9-5p | MYD88 | BMP6 | VEGFA | IL10 | hsa05322 |
| hsa-miR-9-5p | NRDC | BMP6 | IGF1 | IL10 | hsa05144 |
| hsa-miR-9-5p | OLR1 | BMP6 | SMAD3 | IL10 | hsa04660 |
| hsa-miR-9-5p | PTGS2 | BMP6 | BGLAP | IL10 | hsa04061 |
| hsa-miR-9-5p | PTX3 | BRCA1 | SIRT1 | IL10 | hsa05152 |
| hsa-miR-9-5p | TAF1 | BRCA1 | HMOX1 | IL10 | hsa04068 |
| hsa-miR-9-5p | UBC | BRCA1 | APOB | IL12A | hsa05146 |
| hsa-miR-9-3p | CXCR4 | BRCA1 | AHR | IL12A | hsa05168 |
| hsa-miR-9-3p | FSD1L | BRCA1 | VWF | IL12A | hsa05417 |
| hsa-miR-9-3p | MMP2 | BRCA1 | RPS27A | IL12A | hsa04622 |
| hsa-miR-9-3p | MMP9 | BRCA1 | PPARG | IL12A | hsa04630 |
| hsa-miR-9-3p | NFKB1 | BRCA1 | VCAM1 | IL12A | hsa04060 |
| hsa-miR-9-3p | SIRT1 | BRCA1 | IGF1 | IL12A | hsa05152 |
| hsa-miR-126-5p | CNR1 | BRCA1 | SMAD3 | IL12A | hsa05330 |
| hsa-miR-126-5p | SERPINA1 | BRCA1 | PARP1 | IL12A | hsa05321 |
| hsa-miR-126-5p | SERPINE1 | BRCA1 | INS | IL12A | hsa04620 |
| hsa-miR-126-5p | SIRT1 | BRCA1 | VEGFA | IL12A | hsa05140 |
| hsa-miR-126-5p | YWHAZ | BRCA1 | HIF1A | IL12A | hsa05143 |
| hsa-miR-296-5p | MMP2 | BRCA1 | UBC | IL12A | hsa05145 |
| hsa-miR-296-5p | SERPINA1 | BSG | TIMP1 | IL12A | hsa04658 |
| hsa-miR-296-5p | TAF1 | BSG | MMP2 | IL12A | hsa05133 |
| hsa-miR-296-5p | TNC | BSG | TGFB1 | IL12A | hsa04625 |
| hsa-miR-296-5p | UBAP1 | BSG | LGALS3 | IL12A | hsa05162 |
| hsa-miR-296-5p | VEGFA | BSG | ICAM1 | IL12A | hsa05164 |
| hsa-miR-30e-5p | AHR | BSG | CXCL16 | IL12A | hsa04940 |
| hsa-miR-30e-5p | CCN2 | BSG | ITGA2 | IL12A | hsa05171 |
| hsa-miR-30e-5p | ENTPD1 | BSG | MMP3 | IL12A | hsa05142 |
| hsa-miR-30e-5p | FAS | BSG | ADRB2 | IL12A | hsa05144 |
| hsa-miR-30e-5p | FST | BSG | PLG | IL12A | hsa05134 |
| hsa-miR-30e-5p | IL1A | BSG | MMP1 | IL17A | hsa05323 |
| hsa-miR-30e-5p | INTS2 | BSG | SELE | IL17A | hsa04060 |
| hsa-miR-30e-5p | MON2 | BSG | HIF1A | IL17A | hsa04659 |
| hsa-miR-30e-5p | MYDGF | BSG | ITGB3 | IL17A | hsa04657 |
| hsa-miR-30e-5p | PARP1 | BSG | CD63 | IL17A | hsa05321 |
| hsa-miR-30e-5p | PPARGC1A | BSG | VEGFA | IL18 | hsa05130 |
| hsa-miR-30e-5p | SERPINC1 | BSG | MMP9 | IL18 | hsa05143 |
| hsa-miR-30e-5p | VEGFA | C1S | LGALS3 | IL18 | hsa05321 |
| hsa-miR-30e-5p | YWHAZ | C1S | CRP | IL18 | hsa05132 |
| hsa-miR-151a-3p | HIF1A | C1S | APCS | IL18 | hsa05164 |
| hsa-miR-151a-3p | NAMPT | C1S | VWF | IL18 | hsa04623 |
| hsa-miR-148b-3p | BDNF | C1S | KNG1 | IL18 | hsa05131 |
| hsa-miR-148b-3p | CCL5 | C1S | SERPING1 | IL18 | hsa04061 |
| hsa-miR-148b-3p | CCN2 | C1S | PTX3 | IL18 | hsa05417 |
| hsa-miR-148b-3p | CX3CR1 | C1S | SERPINF2 | IL18 | hsa04621 |
| hsa-miR-148b-3p | CXCL8 | C1S | CD163 | IL18 | hsa05323 |
| hsa-miR-148b-3p | F3 | C1S | SCUBE1 | IL18 | hsa05144 |
| hsa-miR-148b-3p | GDF15 | C1S | SERPINC1 | IL18 | hsa05134 |
| hsa-miR-148b-3p | HIF1A | C1S | MBL2 | IL18 | hsa04060 |
| hsa-miR-148b-3p | HMGCR | C1S | SERPINA1 | IL18 | hsa05135 |
| hsa-miR-148b-3p | HSPD1 | CAD | MON2 | IL18 | hsa05152 |
| hsa-miR-148b-3p | IL6 | CCL11 | CX3CL1 | INS | hsa04151 |
| hsa-miR-148b-3p | MYDGF | CCL11 | TIMP1 | INS | hsa04014 |
| hsa-miR-148b-3p | NFKB1 | CCL11 | HGF | INS | hsa04932 |
| hsa-miR-148b-3p | PAPPA | CCL11 | IL2 | INS | hsa04810 |
| hsa-miR-148b-3p | PARP1 | CCL11 | IFNG | INS | hsa04913 |
| hsa-miR-148b-3p | PTGS2 | CCL11 | IL5 | INS | hsa04152 |
| hsa-miR-148b-3p | SERPINE1 | CCL11 | CRP | INS | hsa04066 |
| hsa-miR-148b-3p | UBAP1 | CCL11 | IL1RN | INS | hsa04211 |
| hsa-miR-20b-5p | CCL5 | CCL11 | TLR2 | INS | hsa04068 |
| hsa-miR-20b-5p | CCL5 | CCL11 | IL1A | INS | hsa04931 |
| hsa-miR-20b-5p | CCN2 | CCL11 | IL1B | INS | hsa05215 |
| hsa-miR-20b-5p | CD28 | CCL11 | IL7 | INS | hsa04015 |
| hsa-miR-20b-5p | CTSS | CCL11 | ICAM1 | INS | hsa05415 |
| hsa-miR-20b-5p | CXCL8 | CCL11 | IFNA1 | INS | hsa04940 |
| hsa-miR-20b-5p | CYCS | CCL11 | IL18 | INS | hsa04010 |
| hsa-miR-20b-5p | GDF15 | CCL11 | CXCL13 | ITGA2 | hsa04510 |
| hsa-miR-20b-5p | HIF1A | CCL11 | CXCL16 | ITGA2 | hsa04145 |
| hsa-miR-20b-5p | ITGA2 | CCL11 | VCAM1 | ITGA2 | hsa05165 |
| hsa-miR-20b-5p | LPXN | CCL11 | PPBP | ITGA2 | hsa04151 |
| hsa-miR-20b-5p | PPARG | CCL11 | PF4 | ITGA2 | hsa04512 |
| hsa-miR-20b-5p | SERP1 | CCL11 | MMP3 | ITGA2 | hsa04611 |
| hsa-miR-20b-5p | SERPINE1 | CCL11 | CD86 | ITGA2 | hsa05205 |
| hsa-miR-20b-5p | UBAP1 | CCL11 | SELE | ITGA2 | hsa04810 |
| hsa-miR-20b-5p | UBC | CCL11 | PRG2 | ITGA2 | hsa05414 |
| hsa-miR-490-3p | BRCA1 | CCL11 | MMP9 | ITGA2 | hsa04640 |
| hsa-miR-490-3p | CXCL16 | CCL11 | CD40LG | ITGA2 | hsa05410 |
| hsa-miR-490-3p | LGALS3 | CCL11 | LEP | ITGA2B | hsa04510 |
| hsa-miR-490-3p | PAPPA | CCL11 | IL2RA | ITGA2B | hsa04611 |
| hsa-miR-490-3p | TNNI1 | CCL11 | TLR4 | ITGA2B | hsa04613 |
| hsa-miR-193b-3p | FST | CCL11 | CSF1 | ITGA2B | hsa04640 |
| hsa-miR-193b-3p | HIF1A | CCL11 | IL33 | ITGA2B | hsa05418 |
| hsa-miR-193b-3p | HSPA4 | CCL11 | VEGFA | ITGA2B | hsa05410 |
| hsa-miR-193b-3p | MIF | CCL11 | RNASE3 | ITGA2B | hsa04151 |
| hsa-miR-193b-3p | MORF4L2 | CCL11 | CX3CR1 | ITGA2B | hsa04512 |
| hsa-miR-193b-3p | PARP1 | CCL11 | DPP4 | ITGA2B | hsa05165 |
| hsa-miR-193b-3p | PIK3C2A | CCL11 | TNF | ITGA2B | hsa05414 |
| hsa-miR-193b-3p | PTX3 | CCL11 | CXCL8 | ITGA2B | hsa04015 |
| hsa-miR-520c-3p | ADM | CCL11 | IL6 | ITGA2B | hsa04810 |
| hsa-miR-520c-3p | CCL5 | CCL11 | IL10 | ITGB2 | hsa05152 |
| hsa-miR-520c-3p | CCL5 | CCL11 | IL17A | ITGB2 | hsa05323 |
| hsa-miR-520c-3p | CD28 | CCL11 | AGT | ITGB2 | hsa05166 |
| hsa-miR-520c-3p | CSF1 | CCL11 | F2 | ITGB2 | hsa04670 |
| hsa-miR-520c-3p | CXCL8 | CCL11 | ADRB2 | ITGB2 | hsa05146 |
| hsa-miR-520c-3p | CYCS | CCL11 | EDN1 | ITGB2 | hsa04610 |
| hsa-miR-520c-3p | IL32 | CCL11 | CXCR4 | ITGB2 | hsa04650 |
| hsa-miR-520c-3p | IL37 | CCL2 | CX3CL1 | ITGB2 | hsa04613 |
| hsa-miR-520c-3p | ITGA2 | CCL2 | TNFRSF1A | ITGB2 | hsa05140 |
| hsa-miR-520c-3p | MMP1 | CCL2 | SIRT1 | ITGB2 | hsa05150 |
| hsa-miR-520c-3p | PARP1 | CCL2 | MIF | ITGB2 | hsa04145 |
| hsa-miR-520c-3p | PPARGC1A | CCL2 | HMOX1 | ITGB2 | hsa05416 |
| hsa-miR-520c-3p | PTX3 | CCL2 | IL2RB | ITGB2 | hsa05133 |
| hsa-miR-520c-3p | SERPINE1 | CCL2 | GZMB | ITGB2 | hsa04514 |
| hsa-miR-520c-3p | SH2D1A | CCL2 | TIMP1 | ITGB2 | hsa04810 |
| hsa-miR-520c-3p | THBD | CCL2 | MMP2 | ITGB2 | hsa05134 |
| hsa-miR-520c-3p | TNNT1 | CCL2 | PLAT | ITGB2 | hsa05144 |
| hsa-miR-520c-3p | TXNIP | CCL2 | RETN | ITGB2 | hsa04015 |
| hsa-miR-522-3p | MON2 | CCL2 | TGFB1 | ITGB3 | hsa04380 |
| hsa-miR-522-3p | MORF4L2 | CCL2 | PON1 | ITGB3 | hsa05168 |
| hsa-miR-522-3p | YWHAZ | CCL2 | HGF | ITGB3 | hsa04151 |
| hsa-miR-500a-3p | AHR | CCL2 | NAMPT | ITGB3 | hsa05414 |
| hsa-miR-500a-3p | CAD | CCL2 | SERPINE1 | ITGB3 | hsa05165 |
| hsa-miR-500a-3p | COL6A2 | CCL2 | MPO | ITGB3 | hsa04640 |
| hsa-miR-500a-3p | PARP1 | CCL2 | GDF15 | ITGB3 | hsa04512 |
| hsa-miR-500a-3p | PTGS2 | CCL2 | CNR1 | ITGB3 | hsa05205 |
| hsa-miR-500a-3p | TNFSF14 | CCL2 | FAS | ITGB3 | hsa04015 |
| hsa-miR-500a-3p | YWHAZ | CCL2 | S100A9 | ITGB3 | hsa05410 |
| hsa-miR-505-3p | CSF1 | CCL2 | SERPINC1 | ITGB3 | hsa04611 |
| hsa-miR-505-3p | IGF1 | CCL2 | S100A4 | ITGB3 | hsa04510 |
| hsa-miR-505-3p | TNC | CCL2 | FGF21 | ITGB3 | hsa04145 |
| hsa-miR-421 | HLCS | CCL2 | CD14 | ITGB3 | hsa05163 |
| hsa-miR-421 | HSPD1 | CCL2 | F2 | ITGB3 | hsa04810 |
| hsa-miR-421 | IGF1 | CCL2 | IL32 | ITGB3 | hsa05418 |
| hsa-miR-421 | MORF4L2 | CCL2 | ADM | ITGB3 | hsa04613 |
| hsa-miR-421 | NRDC | CCL2 | IL37 | KNG1 | hsa04610 |
| hsa-miR-421 | PPARGC1A | CCL2 | CYCS | KNG1 | hsa05142 |
| hsa-miR-421 | SELENOS | CCL2 | HSPA4 | KNG1 | hsa05143 |
| hsa-miR-421 | SERPINE1 | CCL2 | KNG1 | KNG1 | hsa04810 |
| hsa-miR-421 | SIRT1 | CCL2 | EPO | LCN2 | hsa04657 |
| hsa-miR-363-5p | ITGA2 | CCL2 | IL34 | LEP | hsa04932 |
| hsa-miR-363-5p | TIMP1 | CCL2 | CYBA | LEP | hsa04060 |
| hsa-miR-363-5p | TXNIP | CCL2 | ITGB2 | LEP | hsa04630 |
| hsa-miR-363-5p | VEGFA | CCL2 | CST3 | LEP | hsa04920 |
| hsa-miR-454-3p | ARSA | CCL2 | DPP4 | LEP | hsa04152 |
| hsa-miR-454-3p | FSD1L | CCL2 | AGT | SH2D1A | hsa04650 |
| hsa-miR-454-3p | PARP1 | CCL2 | SMAD3 | SMAD3 | hsa04350 |
| hsa-miR-454-3p | RPS27A | CCL2 | PPARGC1A | SMAD3 | hsa04659 |
| hsa-miR-769-5p | ADM | CCL2 | ITIH4 | SMAD3 | hsa04371 |
| hsa-miR-769-5p | CTSS | CCL2 | IL2RA | SMAD3 | hsa05415 |
| hsa-miR-769-5p | ICAM1 | CCL2 | APOB | SMAD3 | hsa05166 |
| hsa-miR-769-5p | MIF | CCL2 | PLAU | SMAD3 | hsa05161 |
| hsa-miR-769-5p | MIF | CCL2 | ANGPT1 | SMAD3 | hsa04933 |
| hsa-miR-769-5p | SERPINA1 | CCL2 | APOA1 | SMAD3 | hsa04218 |
| hsa-miR-769-5p | TXNIP | CCL2 | FCGR2A | SMAD3 | hsa05321 |
| hsa-miR-21-3p | BMP6 | CCL2 | TEK | SMAD3 | hsa04068 |
| hsa-miR-21-3p | BSG | CCL2 | THBD | MBL2 | hsa04610 |
| hsa-miR-21-3p | C1S | CCL2 | TNFRSF11B | MBL2 | hsa05150 |
| hsa-miR-21-3p | CCL2 | CCL2 | CD28 | MBL2 | hsa05171 |
| hsa-miR-21-3p | CCL5 | CCL2 | FASLG | MBL2 | hsa04145 |
| hsa-miR-21-3p | CNR1 | CCL2 | ANGPT2 | MMP1 | hsa05219 |
| hsa-miR-21-3p | CTSS | CCL2 | PGF | MMP1 | hsa04926 |
| hsa-miR-21-3p | CX3CL1 | CCL2 | AGER | MMP1 | hsa03320 |
| hsa-miR-21-3p | CXCL16 | CCL2 | TNFRSF1B | MMP1 | hsa05323 |
| hsa-miR-21-3p | CXCL8 | CCL2 | BDNF | MMP1 | hsa05417 |
| hsa-miR-21-3p | CYCS | CCL2 | PTX3 | MMP1 | hsa04657 |
| hsa-miR-21-3p | EBI3 | CCL2 | VWF | MMP1 | hsa05171 |
| hsa-miR-21-3p | ENTPD1 | CCL2 | AGTR1 | MMP2 | hsa04933 |
| hsa-miR-21-3p | FAS | CCL2 | OLR1 | MMP2 | hsa04926 |
| hsa-miR-21-3p | FASLG | CCL2 | PPBP | MMP2 | hsa05418 |
| hsa-miR-21-3p | FERMT3 | CCL2 | ACE | MMP2 | hsa05205 |
| hsa-miR-21-3p | FST | CCL2 | IGF1 | MMP2 | hsa04670 |
| hsa-miR-21-3p | GAS6 | CCL2 | PLG | MMP2 | hsa05415 |
| hsa-miR-21-3p | GDF15 | CCL2 | HIF1A | MMP2 | hsa05219 |
| hsa-miR-21-3p | HMOX1 | CCL2 | REN | MMP3 | hsa04668 |
| hsa-miR-21-3p | ICAM1 | CCL2 | CTGF | MMP3 | hsa05417 |
| hsa-miR-21-3p | IL12A | CCL2 | MMP8 | MMP3 | hsa05215 |
| hsa-miR-21-3p | IL17D | CCL2 | PECAM1 | MMP3 | hsa05171 |
| hsa-miR-21-3p | IL18R1 | CCL2 | CHI3L1 | MMP3 | hsa04657 |
| hsa-miR-21-3p | IL1A | CCL2 | LCN2 | MMP3 | hsa05323 |
| hsa-miR-21-3p | IL32 | CCL2 | IL1RN | MMP3 | hsa05202 |
| hsa-miR-21-3p | IL33 | CCL2 | F3 | MMP9 | hsa05415 |
| hsa-miR-21-3p | IL6 | CCL2 | IL33 | MMP9 | hsa05215 |
| hsa-miR-21-3p | IL7 | CCL2 | EDN1 | MMP9 | hsa04657 |
| hsa-miR-21-3p | ITGA2 | CCL2 | NOS3 | MMP9 | hsa05417 |
| hsa-miR-21-3p | LPXN | CCL2 | CXCL16 | MMP9 | hsa05418 |
| hsa-miR-21-3p | MLKL | CCL2 | CD86 | MMP9 | hsa04668 |
| hsa-miR-21-3p | MMP1 | CCL2 | CD40LG | MMP9 | hsa04926 |
| hsa-miR-21-3p | MMP2 | CCL2 | CD163 | MMP9 | hsa05219 |
| hsa-miR-21-3p | MMP28 | CCL2 | LGALS3 | MMP9 | hsa05202 |
| hsa-miR-21-3p | MON2 | CCL2 | SELP | MMP9 | hsa05205 |
| hsa-miR-21-3p | MYD88 | CCL2 | MYD88 | MMP9 | hsa05161 |
| hsa-miR-21-3p | PARP1 | CCL2 | MMP1 | MMP9 | hsa04670 |
| hsa-miR-21-3p | PLAU | CCL2 | PF4 | MPO | hsa04145 |
| hsa-miR-21-3p | PTGS2 | CCL2 | PPARG | MPO | hsa04613 |
| hsa-miR-21-3p | PTX3 | CCL2 | IFNA1 | MPO | hsa05202 |
| hsa-miR-21-3p | RAB3IL1 | CCL2 | INS | MYD88 | hsa05164 |
| hsa-miR-21-3p | SELP | CCL2 | IL7 | MYD88 | hsa05144 |
| hsa-miR-21-3p | SERPINE1 | CCL2 | LEP | MYD88 | hsa04010 |
| hsa-miR-21-3p | SMAD3 | CCL2 | PTGS2 | MYD88 | hsa05161 |
| hsa-miR-21-3p | THBD | CCL2 | CXCL13 | MYD88 | hsa05162 |
| hsa-miR-21-3p | TIMP1 | CCL2 | TLR2 | MYD88 | hsa05130 |
| hsa-miR-21-3p | TNFRSF11B | CCL2 | SELE | MYD88 | hsa05142 |
| hsa-miR-21-3p | TNFRSF1B | CCL2 | MMP9 | MYD88 | hsa05140 |
| hsa-miR-21-3p | VCAM1 | CCL2 | CSF1 | MYD88 | hsa05170 |
| hsa-miR-21-3p | VEGFA | CCL2 | MMP3 | MYD88 | hsa04620 |
| hsa-miR-21-3p | VWF | CCL2 | CRP | MYD88 | hsa05417 |
| hsa-miR-183-3p | C1S | CCL2 | IL2 | MYD88 | hsa05145 |
| hsa-miR-183-3p | CCN2 | CCL2 | IL5 | MYD88 | hsa05168 |
| hsa-miR-183-3p | NAMPT | CCL2 | TLR4 | MYD88 | hsa04621 |
| hsa-miR-183-3p | SERP1 | CCL2 | IFNG | MYD88 | hsa05131 |
| hsa-miR-124-5p | NFKB1 | CCL2 | IL17A | MYD88 | hsa05132 |
| hsa-miR-144-5p | PTGS2 | CCL2 | VEGFA | MYD88 | hsa05134 |
| hsa-miR-155-3p | IL17A | CCL2 | ICAM1 | MYD88 | hsa05169 |
| hsa-miR-155-3p | MYD88 | CCL2 | NFKB1 | MYD88 | hsa04064 |
| hsa-miR-155-3p | SIRT1 | CCL2 | VCAM1 | MYD88 | hsa05171 |
| hsa-miR-155-3p | TXNIP | CCL2 | IL12A | MYD88 | hsa05143 |
| hsa-miR-374a-3p | ENTPD1 | CCL2 | CXCR4 | MYD88 | hsa05235 |
| hsa-miR-374a-3p | VEGFA | CCL2 | CCL3 | MYD88 | hsa05152 |
| hsa-miR-490-5p | CX3CL1 | CCL2 | CX3CR1 | MYD88 | hsa05133 |
| hsa-miR-501-3p | HIF1A | CCL2 | CCL5 | MYD88 | hsa05135 |
| hsa-miR-501-3p | HSPA4 | CCL2 | IL18 | NFKB1 | hsa04932 |
| hsa-miR-501-3p | TNC | CCL2 | IL1A | NFKB1 | hsa05146 |
| hsa-miR-545-5p | AHR | CCL2 | IL10 | NFKB1 | hsa05415 |
| hsa-miR-545-5p | FST | CCL2 | TNF | NFKB1 | hsa05418 |
| hsa-miR-545-5p | HSPD1 | CCL2 | IL1B | NFKB1 | hsa04668 |
| hsa-miR-545-5p | PIK3C2A | CCL2 | IL6 | NFKB1 | hsa05171 |
| hsa-miR-545-5p | YWHAZ | CCL2 | CXCL8 | NFKB1 | hsa04625 |
| hsa-miR-892a | YWHAZ | CCL3 | CX3CL1 | NFKB1 | hsa04014 |
| hsa-miR-934 | SERPINC1 | CCL3 | TNFRSF1A | NFKB1 | hsa05130 |
| hsa-miR-934 | YWHAZ | CCL3 | MIF | NFKB1 | hsa05132 |
| hsa-miR-938 | ADM | CCL3 | HMOX1 | NFKB1 | hsa05164 |
| hsa-miR-938 | CYP2C19 | CCL3 | GZMB | NFKB1 | hsa05135 |
| hsa-miR-1224-5p | ICAM1 | CCL3 | TIMP1 | NFKB1 | hsa05133 |
| hsa-miR-1224-5p | TNFRSF11B | CCL3 | MMP2 | NFKB1 | hsa05167 |
| hsa-miR-1297 | BRCA1 | CCL3 | RETN | NFKB1 | hsa04659 |
| hsa-miR-1297 | HSPD1 | CCL3 | TGFB1 | NFKB1 | hsa05142 |
| hsa-miR-1297 | ITGA2 | CCL3 | HGF | NFKB1 | hsa04211 |
| hsa-miR-1297 | MALAT1 | CCL3 | SERPINE1 | NFKB1 | hsa05321 |
| hsa-miR-1297 | SERP1 | CCL3 | MPO | NFKB1 | hsa05202 |
| hsa-miR-548l | AHR | CCL3 | CCL2 | NFKB1 | hsa04613 |
| hsa-miR-548l | SIRT1 | CCL3 | IL2 | NFKB1 | hsa04623 |
| hsa-miR-548l | TXNIP | CCL3 | IFNG | NFKB1 | hsa05131 |
| hsa-miR-1253 | PARP1 | CCL3 | IL5 | NFKB1 | hsa05165 |
| hsa-miR-1269a | SMAD3 | CCL3 | MMP8 | NFKB1 | hsa05161 |
| hsa-miR-1269a | UBC | CCL3 | LGALS3 | NFKB1 | hsa04933 |
| hsa-miR-1275 | CD40LG | CCL3 | CRP | NFKB1 | hsa05169 |
| hsa-miR-1275 | FERMT3 | CCL3 | IL1RN | NFKB1 | hsa04620 |
| hsa-miR-1275 | PARP1 | CCL3 | TLR2 | NFKB1 | hsa04660 |
| hsa-miR-1827 | ARSA | CCL3 | IL1A | NFKB1 | hsa04621 |
| hsa-miR-1827 | CYCS | CCL3 | IL1B | NFKB1 | hsa05160 |
| hsa-miR-1827 | F2 | CCL3 | SELP | NFKB1 | hsa04066 |
| hsa-miR-1827 | FABP3 | CCL3 | IL7 | NFKB1 | hsa04926 |
| hsa-miR-1827 | LPXN | CCL3 | ICAM1 | NFKB1 | hsa05417 |
| hsa-miR-1827 | MBL2 | CCL3 | ITIH4 | NFKB1 | hsa05140 |
| hsa-miR-1827 | VCAM1 | CCL3 | FCGR2A | NFKB1 | hsa05163 |
| hsa-miR-1827 | YWHAZ | CCL3 | IFNA1 | NFKB1 | hsa04064 |
| hsa-miR-1915-3p | CYCS | CCL3 | IL18 | NFKB1 | hsa05145 |
| hsa-miR-1915-3p | HMGCR | CCL3 | CXCL13 | NFKB1 | hsa05168 |
| hsa-miR-1915-3p | PCSK9 | CCL3 | PPARG | NFKB1 | hsa04151 |
| hsa-miR-1915-3p | TNFSF14 | CCL3 | CXCL16 | NFKB1 | hsa05166 |
| hsa-miR-4318 | YWHAZ | CCL3 | VCAM1 | NFKB1 | hsa04920 |
| hsa-miR-4261 | SERP1 | CCL3 | PPBP | NFKB1 | hsa04380 |
| hsa-miR-3613-3p | ADM | CCL3 | PF4 | NFKB1 | hsa01523 |
| hsa-miR-3613-3p | HIF1A | CCL3 | TNFRSF11B | NFKB1 | hsa04622 |
| hsa-miR-3613-3p | HSPA4 | CCL3 | MMP3 | NFKB1 | hsa05134 |
| hsa-miR-3613-3p | OLR1 | CCL3 | IGF1 | NFKB1 | hsa04010 |
| hsa-miR-3613-3p | TXNIP | CCL3 | IL12A | NFKB1 | hsa05215 |
| hsa-miR-3622b-3p | CTSS | CCL3 | CD14 | NFKB1 | hsa05235 |
| hsa-miR-3664-5p | CD40LG | CCL3 | CXCL8 | NFKB1 | hsa05152 |
| hsa-miR-642b-3p | SERPINE1 | CCL3 | LEP | NFKB1 | hsa05162 |
| hsa-miR-642b-3p | TXNIP | CCL3 | MMP1 | NFKB1 | hsa04218 |
| hsa-miR-16-5p | VEGFA | CCL3 | CD28 | NFKB1 | hsa04062 |
| hsa-miR-17-5p | VEGFA | CCL3 | CSF1 | NFKB1 | hsa04657 |
| hsa-miR-21-5p | FAS | CCL3 | SELE | NFKB1 | hsa04658 |
| hsa-miR-93-5p | VEGFA | CCL3 | CD86 | NFKB1 | hsa04210 |
| hsa-miR-106a-5p | VEGFA | CCL3 | IL17A | NFKB1 | hsa04931 |
| hsa-miR-205-5p | VEGFA | CCL3 | CX3CR1 | NFKB1 | hsa05170 |
| hsa-let-7i-5p | TLR4 | CCL3 | CD163 | NOS3 | hsa05415 |
| hsa-miR-23b-3p | PLAU | CCL3 | PTGS2 | NOS3 | hsa04066 |
| hsa-miR-140-5p | VEGFA | CCL3 | FASLG | NOS3 | hsa04371 |
| hsa-miR-126-3p | VCAM1 | CCL3 | CD40LG | NOS3 | hsa04933 |
| hsa-miR-126-3p | VEGFA | CCL3 | MMP9 | NOS3 | hsa04151 |
| hsa-miR-146a-5p | CXCR4 | CCL3 | LCN2 | NOS3 | hsa04931 |
| hsa-miR-217 | HIF1A | CCL3 | TLR4 | NOS3 | hsa04611 |
| hsa-miR-320a | YWHAZ | CCL3 | TNFRSF1B | NOS3 | hsa05418 |
| hsa-miR-375 | CCN2 | CCL3 | IL2RA | NOS3 | hsa04926 |
| hsa-miR-375 | COG2 | CCL3 | IL33 | NOS3 | hsa05417 |
| hsa-miR-375 | F3 | CCL3 | INS | NPPB | hsa04270 |
| hsa-miR-375 | ITIH4 | CCL3 | ITGB2 | OLR1 | hsa05417 |
| hsa-miR-375 | NPPB | CCL3 | IL6 | OLR1 | hsa03320 |
| hsa-miR-375 | PARP1 | CCL3 | CXCR4 | OLR1 | hsa04145 |
| hsa-miR-375 | SERP1 | CCL3 | TNF | TNFRSF11B | hsa04060 |
| hsa-miR-375 | TNNI3 | CCL3 | MYD88 | TNFRSF11B | hsa04380 |
| hsa-miR-375 | YWHAZ | CCL3 | IL10 | SERPINE1 | hsa04933 |
| hsa-miR-16-5p | BDNF | CCL3 | BDNF | SERPINE1 | hsa05142 |
| hsa-miR-16-5p | BRCA1 | CCL3 | PECAM1 | SERPINE1 | hsa04371 |
| hsa-miR-16-5p | BSG | CCL3 | CCL5 | SERPINE1 | hsa04610 |
| hsa-miR-16-5p | CYCS | CCL3 | VEGFA | SERPINE1 | hsa04218 |
| hsa-miR-16-5p | F2 | CCL5 | CX3CL1 | SERPINE1 | hsa04066 |
| hsa-miR-16-5p | HGF | CCL5 | TNFRSF1A | PRKN | hsa04137 |
| hsa-miR-16-5p | HMOX1 | CCL5 | MIF | SERPINA5 | hsa04610 |
| hsa-miR-16-5p | HSPD1 | CCL5 | HMOX1 | PECAM1 | hsa05144 |
| hsa-miR-16-5p | ITGA2 | CCL5 | IL2RB | PECAM1 | hsa04514 |
| hsa-miR-16-5p | NAMPT | CCL5 | GZMB | PECAM1 | hsa05418 |
| hsa-miR-16-5p | NFKB1 | CCL5 | TIMP1 | PECAM1 | hsa04670 |
| hsa-miR-16-5p | PTGS2 | CCL5 | MMP2 | PF4 | hsa04061 |
| hsa-miR-16-5p | PYGB | CCL5 | RETN | PF4 | hsa04060 |
| hsa-miR-16-5p | SMAD3 | CCL5 | TGFB1 | PF4 | hsa04062 |
| hsa-miR-17-5p | CTSS | CCL5 | HGF | PGF | hsa04151 |
| hsa-miR-17-5p | F3 | CCL5 | SERPINE1 | PGF | hsa04010 |
| hsa-miR-17-5p | HIF1A | CCL5 | MPO | PGF | hsa04015 |
| hsa-miR-17-5p | ICAM1 | CCL5 | CCL2 | PGF | hsa04510 |
| hsa-miR-17-5p | MMP2 | CCL5 | NFKB1 | PGF | hsa04014 |
| hsa-miR-17-5p | MORF4L2 | CCL5 | IL2 | SERPINA1 | hsa04610 |
| hsa-miR-17-5p | RPS27A | CCL5 | SELPLG | PIK3C2A | hsa05132 |
| hsa-miR-17-5p | SELE | CCL5 | IFNG | PLA2G2A | hsa00591 |
| hsa-miR-17-5p | TXNIP | CCL5 | IL5 | PLA2G2A | hsa04014 |
| hsa-miR-17-5p | UBC | CCL5 | MMP8 | PLA2G2A | hsa04270 |
| hsa-miR-17-5p | YWHAZ | CCL5 | LGALS3 | PLAT | hsa05418 |
| hsa-miR-21-5p | BRCA1 | CCL5 | CRP | PLAT | hsa04610 |
| hsa-miR-21-5p | FASLG | CCL5 | IL1RN | PLAT | hsa04371 |
| hsa-miR-21-5p | ICAM1 | CCL5 | TLR2 | PLAT | hsa05215 |
| hsa-miR-21-5p | IL12A | CCL5 | VWF | PLAT | hsa05202 |
| hsa-miR-21-5p | IL1B | CCL5 | IL1A | PLAU | hsa04610 |
| hsa-miR-21-5p | MMP9 | CCL5 | IL1B | PLAU | hsa05202 |
| hsa-miR-21-5p | MON2 | CCL5 | SELP | PLAU | hsa04064 |
| hsa-miR-21-5p | OLR1 | CCL5 | IL7 | PLAU | hsa05215 |
| hsa-miR-21-5p | PARP1 | CCL5 | ICAM1 | PLAU | hsa05205 |
| hsa-miR-21-5p | PIK3C2A | CCL5 | ITIH4 | PLG | hsa05150 |
| hsa-miR-21-5p | PLAT | CCL5 | FCGR2A | PLG | hsa04610 |
| hsa-miR-21-5p | PTX3 | CCL5 | IFNA1 | PLG | hsa05164 |
| hsa-miR-21-5p | TAF1 | CCL5 | IL18 | SERPINF2 | hsa04610 |
| hsa-miR-21-5p | TGFB1 | CCL5 | TNFSF4 | PPARG | hsa04211 |
| hsa-miR-21-5p | TLR4 | CCL5 | CXCL13 | PPARG | hsa04152 |
| hsa-miR-21-5p | TNFRSF11B | CCL5 | PPARG | PPARG | hsa04380 |
| hsa-miR-21-5p | VEGFA | CCL5 | CXCL16 | PPARG | hsa05417 |
| hsa-miR-26a-5p | ADM | CCL5 | VCAM1 | PPARG | hsa05202 |
| hsa-miR-26a-5p | BRCA1 | CCL5 | PPBP | PPARG | hsa03320 |
| hsa-miR-26a-5p | HGF | CCL5 | PF4 | PPARG | hsa04932 |
| hsa-miR-26a-5p | HSPD1 | CCL5 | MMP3 | PPBP | hsa04062 |
| hsa-miR-26a-5p | PIK3C2A | CCL5 | RNASE3 | PPBP | hsa04061 |
| hsa-miR-26a-5p | PTGS2 | CCL5 | IGF1 | PPBP | hsa04060 |
| hsa-miR-26a-5p | SERP1 | CCL5 | IL12A | PRG2 | hsa05310 |
| hsa-miR-27a-3p | HIF1A | CCL5 | CXCL8 | PTGS2 | hsa04657 |
| hsa-miR-27a-3p | HMGCR | CCL5 | LEP | PTGS2 | hsa05165 |
| hsa-miR-27a-3p | IFNG | CCL5 | MMP1 | PTGS2 | hsa04625 |
| hsa-miR-27a-3p | IGF1 | CCL5 | CD28 | PTGS2 | hsa04668 |
| hsa-miR-27a-3p | OLR1 | CCL5 | CSF1 | PTGS2 | hsa05140 |
| hsa-miR-27a-3p | PPARG | CCL5 | SELE | PTGS2 | hsa04064 |
| hsa-miR-27a-3p | SERP1 | CCL5 | CD86 | PTGS2 | hsa04913 |
| hsa-miR-27a-3p | TXNIP | CCL5 | F3 | PTGS2 | hsa05163 |
| hsa-miR-29a-3p | AHR | CCL5 | IL17A | PTGS2 | hsa05167 |
| hsa-miR-29a-3p | ENTPD1 | CCL5 | CX3CR1 | PYGB | hsa04217 |
| hsa-miR-29a-3p | HMGCR | CCL5 | CD163 | PYGB | hsa04931 |
| hsa-miR-29a-3p | IGF1 | CCL5 | DPP4 | REN | hsa05415 |
| hsa-miR-29a-3p | MMP2 | CCL5 | PTGS2 | REN | hsa04924 |
| hsa-miR-29a-3p | MORF4L2 | CCL5 | FASLG | REN | hsa04614 |
| hsa-miR-29a-3p | VEGFA | CCL5 | CTGF | RNASE3 | hsa05310 |
| hsa-miR-30a-5p | BDNF | CCL5 | S100A4 | RPS27A | hsa05167 |
| hsa-miR-30a-5p | ITGA2 | CCL5 | CD40LG | RPS27A | hsa04137 |
| hsa-miR-30a-5p | YWHAZ | CCL5 | MMP9 | RPS27A | hsa05131 |
| hsa-miR-30a-3p | YWHAZ | CCL5 | LCN2 | RPS27A | hsa05171 |
| hsa-miR-92a-3p | HMGCR | CCL5 | TLR4 | S100A8 | hsa04657 |
| hsa-miR-93-5p | CTSS | CCL5 | TNFRSF1B | S100A9 | hsa04657 |
| hsa-miR-93-5p | CXCL8 | CCL5 | EDN1 | CCL2 | hsa05144 |
| hsa-miR-93-5p | F3 | CCL5 | IL2RA | CCL2 | hsa05418 |
| hsa-miR-93-5p | HIF1A | CCL5 | IL33 | CCL2 | hsa04657 |
| hsa-miR-93-5p | RPS27A | CCL5 | INS | CCL2 | hsa04062 |
| hsa-miR-93-5p | TXNIP | CCL5 | ITGB2 | CCL2 | hsa04933 |
| hsa-miR-93-5p | UBC | CCL5 | IL6 | CCL2 | hsa05142 |
| hsa-miR-93-5p | YWHAZ | CCL5 | CXCR4 | CCL2 | hsa04061 |
| hsa-miR-29b-3p | HMGCR | CCL5 | TNF | CCL2 | hsa05164 |
| hsa-miR-29b-3p | MMP2 | CCL5 | MYD88 | CCL2 | hsa05417 |
| hsa-miR-29b-3p | MORF4L2 | CCL5 | IL10 | CCL2 | hsa05323 |
| hsa-miR-29b-3p | VEGFA | CCL5 | BDNF | CCL2 | hsa04621 |
| hsa-miR-103a-3p | ITGA2 | CCL5 | IL32 | CCL2 | hsa05171 |
| hsa-miR-106a-5p | CXCL8 | CCL5 | HIF1A | CCL2 | hsa05135 |
| hsa-miR-106a-5p | F3 | CCL5 | PECAM1 | CCL2 | hsa05168 |
| hsa-miR-106a-5p | TXNIP | CCL5 | VEGFA | CCL2 | hsa05163 |
| hsa-miR-106a-5p | UBC | CCL5 | CCL3 | CCL2 | hsa04668 |
| hsa-miR-192-5p | BGLAP | CD14 | RIPK3 | CCL2 | hsa04060 |
| hsa-miR-192-5p | BRCA1 | CD14 | CCL2 | CCL3 | hsa04062 |
| hsa-miR-192-5p | IL7 | CD14 | IFNG | CCL3 | hsa05323 |
| hsa-miR-192-5p | PCSK9 | CD14 | IL1RN | CCL3 | hsa05417 |
| hsa-miR-192-5p | PLAU | CD14 | RIPK1 | CCL3 | hsa05142 |
| hsa-miR-192-5p | PPARG | CD14 | TLR2 | CCL3 | hsa04060 |
| hsa-miR-192-5p | SEMA4D | CD14 | IL1B | CCL3 | hsa04620 |
| hsa-miR-192-5p | SIRT1 | CD14 | FCGR2A | CCL3 | hsa04061 |
| hsa-miR-192-5p | THBD | CD14 | RPS27A | CCL3 | hsa05163 |
| hsa-miR-148a-3p | HSPA4 | CD14 | TNFRSF1B | CCL5 | hsa05164 |
| hsa-miR-148a-3p | SERPINE1 | CD14 | CTSS | CCL5 | hsa04062 |
| hsa-miR-148a-3p | TXNIP | CD14 | IL17A | CCL5 | hsa04621 |
| hsa-miR-181b-5p | IL1A | CD14 | MMP9 | CCL5 | hsa05163 |
| hsa-miR-181b-5p | SIRT1 | CD14 | IL2RA | CCL5 | hsa04061 |
| hsa-miR-181c-5p | IL1A | CD14 | CCL3 | CCL5 | hsa05131 |
| hsa-miR-181c-5p | IL2 | CD14 | ADRB2 | CCL5 | hsa04623 |
| hsa-miR-205-5p | CCN2 | CD14 | CX3CR1 | CCL5 | hsa05323 |
| hsa-miR-200b-3p | VEGFA | CD14 | CXCL8 | CCL5 | hsa05168 |
| hsa-let-7g-5p | EDN1 | CD14 | CSF1 | CCL5 | hsa04620 |
| hsa-let-7g-5p | YWHAZ | CD14 | PECAM1 | CCL5 | hsa05142 |
| hsa-let-7i-5p | EDN1 | CD14 | IL10 | CCL5 | hsa04060 |
| hsa-let-7i-5p | YWHAZ | CD14 | S100A9 | CCL5 | hsa05417 |
| hsa-miR-23b-3p | TXNIP | CD14 | IL6 | CCL5 | hsa04668 |
| hsa-miR-27b-3p | OLR1 | CD14 | S100A8 | CCL11 | hsa04062 |
| hsa-miR-27b-3p | PPARG | CD14 | CD86 | CCL11 | hsa04657 |
| hsa-miR-27b-3p | SERP1 | CD14 | TNF | CCL11 | hsa04061 |
| hsa-miR-27b-3p | TXNIP | CD14 | CD163 | CCL11 | hsa05310 |
| hsa-miR-122-5p | HMOX1 | CD14 | ITGB2 | CCL11 | hsa04060 |
| hsa-miR-122-5p | IFNA1 | CD14 | UBC | CX3CL1 | hsa04060 |
| hsa-miR-122-5p | OLR1 | CD14 | HSPD1 | CX3CL1 | hsa05163 |
| hsa-miR-132-3p | PTGS2 | CD14 | MYD88 | CX3CL1 | hsa04061 |
| hsa-miR-132-3p | SIRT1 | CD14 | TLR4 | CX3CL1 | hsa04062 |
| hsa-miR-143-3p | MMP2 | CD163 | CX3CL1 | CX3CL1 | hsa04668 |
| hsa-miR-143-3p | PTGS2 | CD163 | HMOX1 | SELE | hsa04933 |
| hsa-miR-143-3p | SERPINE1 | CD163 | GZMB | SELE | hsa05144 |
| hsa-miR-191-5p | IL1A | CD163 | PLAT | SELE | hsa05417 |
| hsa-miR-146a-5p | BGLAP | CD163 | MPO | SELE | hsa04668 |
| hsa-miR-146a-5p | BRCA1 | CD163 | CCL2 | SELE | hsa05143 |
| hsa-miR-146a-5p | CXCL8 | CD163 | IL2 | SELE | hsa05418 |
| hsa-miR-146a-5p | FAS | CD163 | IFNG | SELE | hsa04514 |
| hsa-miR-146a-5p | ICAM1 | CD163 | LGALS3 | SELP | hsa05417 |
| hsa-miR-146a-5p | ITGB2 | CD163 | CRP | SELP | hsa04514 |
| hsa-miR-146a-5p | PTGS2 | CD163 | CHI3L1 | SELP | hsa04613 |
| hsa-miR-146a-5p | TLR2 | CD163 | IL1RN | SELP | hsa05144 |
| hsa-miR-186-5p | HIF1A | CD163 | TLR2 | SELP | hsa05150 |
| hsa-miR-186-5p | VEGFA | CD163 | VWF | SELP | hsa05171 |
| hsa-miR-193a-3p | PLAU | CD163 | IL1A | SELPLG | hsa04514 |
| hsa-miR-193a-3p | TNFRSF1B | CD163 | IL1B | SELPLG | hsa04613 |
| hsa-miR-200c-3p | SIRT1 | CD163 | ICAM1 | SELPLG | hsa05150 |
| hsa-miR-200c-3p | VEGFA | CD163 | FCGR2A | TEK | hsa04014 |
| hsa-miR-29c-3p | COL6A2 | CD163 | IFNA1 | TEK | hsa04066 |
| hsa-miR-29c-3p | FGA | CD163 | IL18 | TEK | hsa04151 |
| hsa-miR-29c-3p | HMGCR | CD163 | CXCL13 | TEK | hsa04010 |
| hsa-miR-29c-3p | MMP2 | CD163 | PPARG | TEK | hsa05323 |
| hsa-miR-29c-3p | MORF4L2 | CD163 | VCAM1 | TEK | hsa04015 |
| hsa-miR-29c-3p | SIRT1 | CD163 | CD14 | TGFB1 | hsa05205 |
| hsa-miR-29c-3p | VEGFA | CD163 | CXCL8 | TGFB1 | hsa05323 |
| hsa-miR-301a-3p | EDN1 | CD163 | CSF1 | TGFB1 | hsa04380 |
| hsa-miR-301a-3p | PARP1 | CD163 | CD86 | TGFB1 | hsa04218 |
| hsa-miR-301a-3p | SERPINE1 | CD163 | IL17A | TGFB1 | hsa05410 |
| hsa-miR-301a-3p | TXNIP | CD163 | CX3CR1 | TGFB1 | hsa05166 |
| hsa-miR-301a-3p | UBC | CD163 | HIF1A | TGFB1 | hsa05321 |
| hsa-miR-361-5p | VEGFA | CD163 | PTGS2 | TGFB1 | hsa05144 |
| hsa-miR-340-3p | YWHAZ | CD163 | ITGB2 | TGFB1 | hsa05145 |
| hsa-miR-338-3p | HIF1A | CD163 | THBD | TGFB1 | hsa05152 |
| hsa-miR-338-3p | MMP2 | CD163 | IL34 | TGFB1 | hsa05140 |
| hsa-miR-196b-5p | FAS | CD163 | CXCR4 | TGFB1 | hsa05161 |
| hsa-miR-423-3p | VEGFA | CD163 | GP5 | TGFB1 | hsa04060 |
| hsa-miR-18b-5p | CAD | CD163 | CCL5 | TGFB1 | hsa05146 |
| hsa-miR-18b-5p | CCN2 | CD163 | VEGFA | TGFB1 | hsa04926 |
| hsa-miR-18b-5p | ITGA2 | CD163 | MMP9 | TGFB1 | hsa04659 |
| hsa-miR-429 | HIF1A | CD163 | CCL3 | TGFB1 | hsa05142 |
| hsa-miR-451a | MIF | CD163 | C1S | TGFB1 | hsa04672 |
| hsa-miR-652-3p | BSG | CD163 | TLR4 | TGFB1 | hsa05415 |
| hsa-miR-660-5p | ADM | CD163 | PECAM1 | TGFB1 | hsa04350 |
| hsa-miR-671-5p | YWHAZ | CD163 | S100A9 | TGFB1 | hsa04068 |
| hsa-miR-767-5p | MMP2 | CD163 | TNF | TGFB1 | hsa04932 |
| hsa-miR-129-2-3p | PARP1 | CD163 | IL6 | TGFB1 | hsa04010 |
| hsa-miR-411-3p | ADM | CD163 | IL10 | TGFB1 | hsa04933 |
| hsa-miR-744-5p | HSPD1 | CD28 | TNFRSF1A | TGFB1 | hsa05414 |
| hsa-miR-744-5p | MIF | CD28 | IL2RB | THBD | hsa05418 |
| hsa-miR-873-5p | ITGA2 | CD28 | GZMB | THBD | hsa04933 |
| hsa-miR-873-5p | TXNIP | CD28 | TGFB1 | THBD | hsa04610 |
| hsa-miR-3605-5p | MORF4L2 | CD28 | CCL2 | THBS4 | hsa05144 |
| hsa-miR-147b | AHR | CD28 | IL2 | THBS4 | hsa04510 |
| hsa-miR-190b | IGF1 | CD28 | IFNG | THBS4 | hsa04512 |
| hsa-miR-190b | SERP1 | CD28 | IL5 | THBS4 | hsa04151 |
| hsa-miR-190b | SERPINA1 | CD28 | AHR | THBS4 | hsa04145 |
| hsa-miR-1912 | PCSK9 | CD28 | CRP | THBS4 | hsa05165 |
| hsa-miR-1912 | SELENOS | CD28 | TLR2 | TIMP1 | hsa04066 |
| hsa-miR-217 | HSPA4 | CD28 | ITGA2B | TLR2 | hsa05145 |
| hsa-miR-217 | INTS2 | CD28 | IL1A | TLR2 | hsa05321 |
| hsa-miR-217 | MON2 | CD28 | IL1B | TLR2 | hsa05417 |
| hsa-miR-217 | SIRT1 | CD28 | SELP | TLR2 | hsa05170 |
| hsa-miR-320a | ADM | CD28 | IL7 | TLR2 | hsa05161 |
| hsa-miR-320a | CD63 | CD28 | ICAM1 | TLR2 | hsa05171 |
| hsa-miR-320a | CYBA | CD28 | ITIH4 | TLR2 | hsa05142 |
| hsa-miR-320a | FAS | CD28 | FCGR2A | TLR2 | hsa05168 |
| hsa-miR-320a | HSPA4 | CD28 | IFNA1 | TLR2 | hsa05146 |
| hsa-miR-320a | ITGB3 | CD28 | IL18 | TLR2 | hsa05144 |
| hsa-miR-320a | MIF | CD28 | TNFSF4 | TLR2 | hsa05132 |
| hsa-miR-320a | MIF | CD28 | VCAM1 | TLR2 | hsa04151 |
| hsa-miR-320a | MMP9 | CD28 | ITGA2 | TLR2 | hsa05162 |
| hsa-miR-320a | PARP1 | CD28 | CXCL8 | TLR2 | hsa05140 |
| hsa-miR-320a | SMAD3 | CD28 | CSF1 | TLR2 | hsa05152 |
| hsa-miR-320a | TNFSF14 | CD28 | TNFRSF1B | TLR2 | hsa04613 |
| hsa-miR-320a | TXNIP | CD28 | VEGFA | TLR2 | hsa04620 |
| hsa-miR-320a | UBAP1 | CD28 | IL33 | TLR2 | hsa05205 |
| hsa-miR-320a | VEGFA | CD28 | INS | TLR2 | hsa05134 |
| hsa-miR-375 | AHR | CD28 | CX3CR1 | TLR2 | hsa05235 |
| hsa-miR-375 | BDNF | CD28 | DPP4 | TLR2 | hsa05323 |
| hsa-miR-375 | CTSS | CD28 | PECAM1 | TLR2 | hsa05169 |
| hsa-miR-375 | EDN1 | CD28 | SH2D1A | TLR2 | hsa04145 |
| hsa-miR-375 | GDF15 | CD28 | MYD88 | TLR4 | hsa05135 |
| hsa-miR-375 | HIF1A | CD28 | CCL3 | TLR4 | hsa05131 |
| hsa-miR-375 | HMGCR | CD28 | CCL5 | TLR4 | hsa04620 |
| hsa-miR-375 | HSPA4 | CD28 | TLR4 | TLR4 | hsa05144 |
| hsa-miR-375 | ICAM1 | CD28 | ENTPD1 | TLR4 | hsa04066 |
| hsa-miR-375 | IL1B | CD28 | CXCR4 | TLR4 | hsa05134 |
| hsa-miR-375 | IL6 | CD28 | FASLG | TLR4 | hsa05235 |
| hsa-miR-375 | MALAT1 | CD28 | IL2RA | TLR4 | hsa05152 |
| hsa-miR-375 | MORF4L2 | CD28 | IL6 | TLR4 | hsa04621 |
| hsa-miR-375 | PECAM1 | CD28 | TNF | TLR4 | hsa05140 |
| hsa-miR-375 | PTX3 | CD28 | IL17A | TLR4 | hsa05417 |
| hsa-miR-375 | RNASE3 | CD28 | IL10 | TLR4 | hsa05133 |
| hsa-miR-375 | S100A4 | CD28 | FAS | TLR4 | hsa05161 |
| hsa-miR-375 | TXNIP | CD28 | CD40LG | TLR4 | hsa04217 |
| hsa-miR-375 | VEGFA | CD28 | ITGB3 | TLR4 | hsa05146 |
| hsa-miR-16-5p | ADM | CD28 | HLA-DRB1 | TLR4 | hsa05162 |
| hsa-miR-16-5p | ADRB2 | CD28 | ITGB2 | TLR4 | hsa04145 |
| hsa-miR-16-5p | AGER | CD28 | CD86 | TLR4 | hsa05171 |
| hsa-miR-16-5p | AGER | CD40LG | CX3CL1 | TLR4 | hsa05321 |
| hsa-miR-16-5p | AGER | CD40LG | TNFRSF1A | TLR4 | hsa05323 |
| hsa-miR-16-5p | AGER | CD40LG | IL2RB | TLR4 | hsa04613 |
| hsa-miR-16-5p | AGER | CD40LG | GZMB | TLR4 | hsa05164 |
| hsa-miR-16-5p | AGER | CD40LG | SERPINE1 | TLR4 | hsa05132 |
| hsa-miR-16-5p | AGER | CD40LG | MPO | TLR4 | hsa04151 |
| hsa-miR-16-5p | AGT | CD40LG | CCL2 | TLR4 | hsa05205 |
| hsa-miR-16-5p | AGTR1 | CD40LG | NFKB1 | TLR4 | hsa05170 |
| hsa-miR-16-5p | AHR | CD40LG | IL2 | TLR4 | hsa05142 |
| hsa-miR-16-5p | ANGPT1 | CD40LG | SELPLG | TLR4 | hsa05130 |
| hsa-miR-16-5p | APOB | CD40LG | IFNG | TLR4 | hsa05145 |
| hsa-miR-16-5p | C1S | CD40LG | IL5 | TLR4 | hsa04064 |
| hsa-miR-16-5p | CCL11 | CD40LG | APOA1 | TNF | hsa05323 |
| hsa-miR-16-5p | CCL2 | CD40LG | CRP | TNF | hsa04622 |
| hsa-miR-16-5p | CCN2 | CD40LG | IL1RN | TNF | hsa01523 |
| hsa-miR-16-5p | CXCL16 | CD40LG | TLR2 | TNF | hsa05160 |
| hsa-miR-16-5p | CXCL8 | CD40LG | VWF | TNF | hsa04380 |
| hsa-miR-16-5p | CXCR4 | CD40LG | ITGA2B | TNF | hsa04621 |
| hsa-miR-16-5p | EDN1 | CD40LG | IL1A | TNF | hsa04350 |
| hsa-miR-16-5p | ENTPD1 | CD40LG | IL1B | TNF | hsa05168 |
| hsa-miR-16-5p | F3 | CD40LG | SELP | TNF | hsa05310 |
| hsa-miR-16-5p | F7 | CD40LG | IL7 | TNF | hsa04217 |
| hsa-miR-16-5p | FAS | CD40LG | ICAM1 | TNF | hsa04064 |
| hsa-miR-16-5p | FST | CD40LG | ITIH4 | TNF | hsa04625 |
| hsa-miR-16-5p | HIF1A | CD40LG | FCGR2A | TNF | hsa05332 |
| hsa-miR-16-5p | HMGCR | CD40LG | IFNA1 | TNF | hsa05322 |
| hsa-miR-16-5p | HSPA4 | CD40LG | IL18 | TNF | hsa05135 |
| hsa-miR-16-5p | ICAM1 | CD40LG | TNFSF4 | TNF | hsa05166 |
| hsa-miR-16-5p | IFNG | CD40LG | CXCL13 | TNF | hsa04920 |
| hsa-miR-16-5p | IGF1 | CD40LG | MRAS | TNF | hsa05171 |
| hsa-miR-16-5p | IGFBP1 | CD40LG | VCAM1 | TNF | hsa05417 |
| hsa-miR-16-5p | IL17D | CD40LG | PPBP | TNF | hsa04668 |
| hsa-miR-16-5p | IL1A | CD40LG | PF4 | TNF | hsa05146 |
| hsa-miR-16-5p | IL2RB | CD40LG | TNFRSF11B | TNF | hsa05321 |
| hsa-miR-16-5p | IL32 | CD40LG | CCL11 | TNF | hsa04060 |
| hsa-miR-16-5p | IL6 | CD40LG | CXCL8 | TNF | hsa05134 |
| hsa-miR-16-5p | IL7 | CD40LG | F2 | TNF | hsa05145 |
| hsa-miR-16-5p | LCN2 | CD40LG | PLG | TNF | hsa04640 |
| hsa-miR-16-5p | LPXN | CD40LG | CD28 | TNF | hsa05164 |
| hsa-miR-16-5p | MMP3 | CD40LG | CSF1 | TNF | hsa04940 |
| hsa-miR-16-5p | MON2 | CD40LG | GP1BA | TNF | hsa05165 |
| hsa-miR-16-5p | MRAS | CD40LG | SELE | TNF | hsa05170 |
| hsa-miR-16-5p | MYD88 | CD40LG | CD86 | TNF | hsa05132 |
| hsa-miR-16-5p | MYDGF | CD40LG | F3 | TNF | hsa04612 |
| hsa-miR-16-5p | PAPPA | CD40LG | IL17A | TNF | hsa05418 |
| hsa-miR-16-5p | PARP1 | CD40LG | FAS | TNF | hsa04210 |
| hsa-miR-16-5p | PCSK9 | CD40LG | CX3CR1 | TNF | hsa05144 |
| hsa-miR-16-5p | PIK3C2A | CD40LG | PTGS2 | TNF | hsa05130 |
| hsa-miR-16-5p | PLAT | CD40LG | SERPINC1 | TNF | hsa04931 |
| hsa-miR-16-5p | PLAU | CD40LG | FASLG | TNF | hsa05152 |
| hsa-miR-16-5p | PPARG | CD40LG | IL33 | TNF | hsa04933 |
| hsa-miR-16-5p | PTX3 | CD40LG | PECAM1 | TNF | hsa05142 |
| hsa-miR-16-5p | REN | CD40LG | CD63 | TNF | hsa04657 |
| hsa-miR-16-5p | RIPK1 | CD40LG | INS | TNF | hsa04620 |
| hsa-miR-16-5p | S100A4 | CD40LG | ENTPD1 | TNF | hsa05414 |
| hsa-miR-16-5p | SELENOS | CD40LG | SH2D1A | TNF | hsa05133 |
| hsa-miR-16-5p | SERPINA1 | CD40LG | MMP9 | TNF | hsa05410 |
| hsa-miR-16-5p | SERPINE1 | CD40LG | THBD | TNF | hsa04010 |
| hsa-miR-16-5p | SULT1E1 | CD40LG | VEGFA | TNF | hsa05330 |
| hsa-miR-16-5p | TEK | CD40LG | MYD88 | TNF | hsa04932 |
| hsa-miR-16-5p | TGFB1 | CD40LG | CXCR4 | TNF | hsa05205 |
| hsa-miR-16-5p | THBD | CD40LG | IL2RA | TNF | hsa05131 |
| hsa-miR-16-5p | TLR4 | CD40LG | CCL3 | TNF | hsa05140 |
| hsa-miR-16-5p | TNC | CD40LG | CCL5 | TNF | hsa04650 |
| hsa-miR-16-5p | TNFRSF11B | CD40LG | TNFRSF11A | TNF | hsa05169 |
| hsa-miR-16-5p | TXNIP | CD40LG | IL6 | TNF | hsa05143 |
| hsa-miR-16-5p | UBAP1 | CD40LG | TNFSF14 | TNF | hsa05161 |
| hsa-miR-16-5p | VIP | CD40LG | TLR4 | TNF | hsa04061 |
| hsa-miR-16-5p | YWHAZ | CD40LG | TNFRSF1B | TNF | hsa05163 |
| hsa-miR-17-5p | ADRB2 | CD40LG | IL10 | TNF | hsa04660 |
| hsa-miR-17-5p | CAD | CD40LG | TNF | TNFRSF1A | hsa05164 |
| hsa-miR-17-5p | CCL5 | CD40LG | ITGB2 | TNFRSF1A | hsa04380 |
| hsa-miR-17-5p | CCL5 | CD63 | TIMP1 | TNFRSF1A | hsa05132 |
| hsa-miR-17-5p | CCN2 | CD63 | LGALS3 | TNFRSF1A | hsa04217 |
| hsa-miR-17-5p | CD28 | CD63 | VWF | TNFRSF1A | hsa05168 |
| hsa-miR-17-5p | CSF1 | CD63 | ITGA2B | TNFRSF1A | hsa05163 |
| hsa-miR-17-5p | CXCL8 | CD63 | IL1B | TNFRSF1A | hsa04064 |
| hsa-miR-17-5p | CYCS | CD63 | SELP | TNFRSF1A | hsa05167 |
| hsa-miR-17-5p | FAS | CD63 | ICAM1 | TNFRSF1A | hsa05152 |
| hsa-miR-17-5p | GDF15 | CD63 | VCAM1 | TNFRSF1A | hsa04932 |
| hsa-miR-17-5p | ITGA2 | CD63 | HSPA4 | TNFRSF1A | hsa05418 |
| hsa-miR-17-5p | ITGB3 | CD63 | CXCL8 | TNFRSF1A | hsa05417 |
| hsa-miR-17-5p | LPXN | CD63 | MMP1 | TNFRSF1A | hsa04010 |
| hsa-miR-17-5p | SERP1 | CD63 | GP1BA | TNFRSF1A | hsa05160 |
| hsa-miR-17-5p | SERPINE1 | CD63 | CD86 | TNFRSF1A | hsa04668 |
| hsa-miR-17-5p | SMAD3 | CD63 | BSG | TNFRSF1A | hsa04210 |
| hsa-miR-17-5p | TGFB1 | CD63 | F3 | TNFRSF1A | hsa04920 |
| hsa-miR-17-5p | TNC | CD63 | CD40LG | TNFRSF1A | hsa04931 |
| hsa-miR-17-5p | TNF | CD63 | MMP9 | TNFRSF1A | hsa05130 |
| hsa-miR-17-5p | TNF | CD63 | IL6 | TNFRSF1A | hsa05165 |
| hsa-miR-17-5p | TNF | CD63 | CXCR4 | TNFRSF1A | hsa05166 |
| hsa-miR-17-5p | TNF | CD63 | TNF | TNFRSF1A | hsa05171 |
| hsa-miR-17-5p | TNF | CD63 | VEGFA | TNFRSF1A | hsa04060 |
| hsa-miR-17-5p | TNF | CD63 | PECAM1 | TNFRSF1A | hsa04061 |
| hsa-miR-17-5p | TNF | CD63 | ITGB3 | TNFRSF1A | hsa05131 |
| hsa-miR-17-5p | TNF | CD86 | CX3CL1 | TNFRSF1A | hsa05170 |
| hsa-miR-17-5p | TNFSF14 | CD86 | TNFRSF1A | TNFRSF1A | hsa05142 |
| hsa-miR-17-5p | UBAP1 | CD86 | IL2RB | TNFRSF1A | hsa05145 |
| hsa-miR-21-5p | CXCL8 | CD86 | GZMB | TNFRSF1B | hsa05170 |
| hsa-miR-21-5p | GP5 | CD86 | TGFB1 | TNFRSF1B | hsa04061 |
| hsa-miR-21-5p | HIF1A | CD86 | MPO | TNFRSF1B | hsa04060 |
| hsa-miR-21-5p | HSPA4 | CD86 | CCL2 | TNFRSF1B | hsa04668 |
| hsa-miR-21-5p | IL10 | CD86 | IL2 | TNFRSF1B | hsa04920 |
| hsa-miR-21-5p | IL6 | CD86 | SELPLG | TNNI3 | hsa05414 |
| hsa-miR-21-5p | MMP2 | CD86 | IFNG | TNNI3 | hsa05415 |
| hsa-miR-21-5p | MYD88 | CD86 | IL5 | TNNI3 | hsa05410 |
| hsa-miR-21-5p | NFKB1 | CD86 | IL1RN | TNNT2 | hsa05410 |
| hsa-miR-21-5p | PAPPA | CD86 | TLR2 | TNNT2 | hsa05414 |
| hsa-miR-21-5p | RPS27A | CD86 | IL1A | TNFSF4 | hsa04060 |
| hsa-miR-21-5p | TEK | CD86 | IL1B | UBC | hsa04137 |
| hsa-miR-21-5p | TXNIP | CD86 | SELP | UBC | hsa05167 |
| hsa-miR-25-3p | ADM | CD86 | IL7 | UBC | hsa05131 |
| hsa-miR-25-3p | AHR | CD86 | ICAM1 | UBC | hsa03320 |
| hsa-miR-25-3p | CAD | CD86 | ITIH4 | VCAM1 | hsa05417 |
| hsa-miR-25-3p | CCN2 | CD86 | FCGR2A | VCAM1 | hsa05144 |
| hsa-miR-25-3p | CYP2C19 | CD86 | IFNA1 | VCAM1 | hsa04514 |
| hsa-miR-25-3p | FASLG | CD86 | IL18 | VCAM1 | hsa05418 |
| hsa-miR-25-3p | HMGCR | CD86 | TNFSF4 | VCAM1 | hsa04933 |
| hsa-miR-25-3p | HSPA4 | CD86 | CXCL13 | VCAM1 | hsa04668 |
| hsa-miR-25-3p | HSPD1 | CD86 | VCAM1 | VCAM1 | hsa04670 |
| hsa-miR-25-3p | PIK3C2A | CD86 | ITGA2 | VCAM1 | hsa04064 |
| hsa-miR-25-3p | SEMA4D | CD86 | CCL11 | VCAM1 | hsa05143 |
| hsa-miR-25-3p | SERP1 | CD86 | HSPA4 | VEGFA | hsa04066 |
| hsa-miR-25-3p | TAF1 | CD86 | CD14 | VEGFA | hsa04926 |
| hsa-miR-25-3p | TNFRSF11B | CD86 | CXCL8 | VEGFA | hsa01521 |
| hsa-miR-25-3p | TXNIP | CD86 | CD28 | VEGFA | hsa05165 |
| hsa-miR-26a-5p | AHR | CD86 | CSF1 | VEGFA | hsa04010 |
| hsa-miR-26a-5p | BSG | CD86 | SELE | VEGFA | hsa05323 |
| hsa-miR-26a-5p | C1S | CD86 | CD63 | VEGFA | hsa04510 |
| hsa-miR-26a-5p | CCN2 | CD86 | S100A9 | VEGFA | hsa05205 |
| hsa-miR-26a-5p | CNR1 | CD86 | PTGS2 | VEGFA | hsa04014 |
| hsa-miR-26a-5p | CTSS | CD86 | TNFRSF1B | VEGFA | hsa05163 |
| hsa-miR-26a-5p | CYCS | CD86 | INS | VEGFA | hsa05167 |
| hsa-miR-26a-5p | EBI3 | CD86 | IL33 | VEGFA | hsa05418 |
| hsa-miR-26a-5p | EDN1 | CD86 | CTSS | VEGFA | hsa04933 |
| hsa-miR-26a-5p | IGF1 | CD86 | MMP9 | VEGFA | hsa04015 |
| hsa-miR-26a-5p | IL6 | CD86 | VEGFA | VEGFA | hsa04151 |
| hsa-miR-26a-5p | IL7 | CD86 | THBD | VEGFA | hsa05219 |
| hsa-miR-26a-5p | INTS2 | CD86 | PECAM1 | VWF | hsa04610 |
| hsa-miR-26a-5p | ITGA2 | CD86 | ENTPD1 | VWF | hsa04611 |
| hsa-miR-26a-5p | ITGB3 | CD86 | FASLG | VWF | hsa04613 |
| hsa-miR-26a-5p | MIF | CD86 | IL2RA | VWF | hsa04512 |
| hsa-miR-26a-5p | MON2 | CD86 | CX3CR1 | VWF | hsa04151 |
| hsa-miR-26a-5p | MRAS | CD86 | CXCR4 | VWF | hsa05171 |
| hsa-miR-26a-5p | NAMPT | CD86 | FAS | VWF | hsa05165 |
| hsa-miR-26a-5p | NOS3 | CD86 | ITGB2 | VWF | hsa04510 |
| hsa-miR-26a-5p | PECAM1 | CD86 | CCL5 | YWHAZ | hsa05161 |
| hsa-miR-26a-5p | PTX3 | CD86 | MYD88 | YWHAZ | hsa04151 |
| hsa-miR-26a-5p | RPS27A | CD86 | IL17A | YWHAZ | hsa05160 |
| hsa-miR-26a-5p | S100A4 | CD86 | CCL3 | CXCR4 | hsa04670 |
| hsa-miR-26a-5p | TIMP1 | CD86 | CD163 | CXCR4 | hsa04060 |
| hsa-miR-26a-5p | TXNIP | CD86 | TLR4 | CXCR4 | hsa04061 |
| hsa-miR-26a-5p | VCAM1 | CD86 | IL6 | CXCR4 | hsa04672 |
| hsa-miR-26a-5p | YWHAZ | CD86 | TNF | CXCR4 | hsa05170 |
| hsa-miR-27a-3p | ADAMTS4 | CD86 | CD40LG | CXCR4 | hsa05163 |
| hsa-miR-27a-3p | ADRB2 | CD86 | HLA-DRB1 | CXCR4 | hsa04810 |
| hsa-miR-27a-3p | ANGPTL2 | CD86 | IL10 | CXCR4 | hsa04062 |
| hsa-miR-27a-3p | APCS | CETP | IL6 | RIPK1 | hsa05169 |
| hsa-miR-27a-3p | CAD | CETP | TXNIP | RIPK1 | hsa05160 |
| hsa-miR-27a-3p | CD14 | CETP | VCAM1 | RIPK1 | hsa04064 |
| hsa-miR-27a-3p | CD163 | CETP | LPXN | RIPK1 | hsa04621 |
| hsa-miR-27a-3p | CD28 | CETP | PPARG | RIPK1 | hsa04668 |
| hsa-miR-27a-3p | CD86 | CETP | ACE | RIPK1 | hsa04623 |
| hsa-miR-27a-3p | CHI3L1 | CETP | CRP | RIPK1 | hsa04210 |
| hsa-miR-27a-3p | CNR1 | CETP | INS | RIPK1 | hsa04622 |
| hsa-miR-27a-3p | CSF1 | CETP | HMGCR | RIPK1 | hsa05170 |
| hsa-miR-27a-3p | CST3 | CETP | SAA4 | RIPK1 | hsa05132 |
| hsa-miR-27a-3p | CX3CR1 | CETP | VIMP | RIPK1 | hsa04217 |
| hsa-miR-27a-3p | CXCL16 | CETP | PCSK9 | RIPK1 | hsa05131 |
| hsa-miR-27a-3p | CXCL8 | CETP | PON1 | RIPK1 | hsa05163 |
| hsa-miR-27a-3p | CXCR4 | CETP | APOA1 | RIPK1 | hsa04620 |
| hsa-miR-27a-3p | CYBA | CETP | APOB | RIPK1 | hsa05130 |
| hsa-miR-27a-3p | CYP2C19 | CHGA | PLG | TNFSF14 | hsa04064 |
| hsa-miR-27a-3p | EDN1 | CHGA | TNFRSF1B | TNFSF14 | hsa04061 |
| hsa-miR-27a-3p | F10 | CHGA | VEGFA | TNFSF14 | hsa05168 |
| hsa-miR-27a-3p | F3 | CHGA | SERPINA1 | TNFSF14 | hsa04060 |
| hsa-miR-27a-3p | FASLG | CHGA | INS | TNFRSF11A | hsa04060 |
| hsa-miR-27a-3p | FST | CHGA | VIP | TNFRSF11A | hsa04380 |
| hsa-miR-27a-3p | HABP2 | CHI3L1 | TIMP1 | TNFRSF11A | hsa05323 |
| hsa-miR-27a-3p | HDAC9 | CHI3L1 | MMP2 | TNFRSF11A | hsa04064 |
| hsa-miR-27a-3p | HGF | CHI3L1 | RETN | IL18R1 | hsa04060 |
| hsa-miR-27a-3p | HRG | CHI3L1 | CCL2 | IL18R1 | hsa04668 |
| hsa-miR-27a-3p | HSPD1 | CHI3L1 | LGALS3 | IL18R1 | hsa04061 |
| hsa-miR-27a-3p | IL10 | CHI3L1 | CRP | IL18R1 | hsa05321 |
| hsa-miR-27a-3p | IL18 | CHI3L1 | CXCL13 | IL32 | hsa04060 |
| hsa-miR-27a-3p | IL18R1 | CHI3L1 | VCAM1 | GDF15 | hsa04060 |
| hsa-miR-27a-3p | IL2RA | CHI3L1 | PTX3 | HDAC9 | hsa04613 |
| hsa-miR-27a-3p | IL2RB | CHI3L1 | CST3 | NAMPT | hsa04621 |
| hsa-miR-27a-3p | IL32 | CHI3L1 | CD163 | EBI3 | hsa04060 |
| hsa-miR-27a-3p | IL6 | CHI3L1 | IL10 | FST | hsa04350 |
| hsa-miR-27a-3p | ITGB2 | CHI3L1 | IL1B | CXCL13 | hsa04062 |
| hsa-miR-27a-3p | LEP | CHI3L1 | MMP1 | CXCL13 | hsa04061 |
| hsa-miR-27a-3p | MB | CHI3L1 | VEGFA | CXCL13 | hsa04060 |
| hsa-miR-27a-3p | MLKL | CHI3L1 | MMP3 | TXNIP | hsa04621 |
| hsa-miR-27a-3p | MMP1 | CHI3L1 | TNF | PPARGC1A | hsa04211 |
| hsa-miR-27a-3p | MYDGF | CHI3L1 | CXCL8 | PPARGC1A | hsa04371 |
| hsa-miR-27a-3p | NFKB1 | CHI3L1 | MMP9 | PPARGC1A | hsa04920 |
| hsa-miR-27a-3p | NPPB | CHI3L1 | LCN2 | PPARGC1A | hsa04152 |
| hsa-miR-27a-3p | NRDC | CHI3L1 | IL6 | PPARGC1A | hsa04931 |
| hsa-miR-27a-3p | PAPPA | CHI3L1 | IGF1 | RIPK3 | hsa04217 |
| hsa-miR-27a-3p | PARP1 | CHIT1 | ACE | RIPK3 | hsa04668 |
| hsa-miR-27a-3p | PCSK9 | CHIT1 | IL6 | RIPK3 | hsa04623 |
| hsa-miR-27a-3p | PECAM1 | CHIT1 | TNF | RIPK3 | hsa05132 |
| hsa-miR-27a-3p | PF4 | CNR1 | CCL2 | RIPK3 | hsa04621 |
| hsa-miR-27a-3p | PIK3C2A | CNR1 | VWF | MRAS | hsa05205 |
| hsa-miR-27a-3p | PLAT | CNR1 | IL1B | MRAS | hsa04010 |
| hsa-miR-27a-3p | PLG | CNR1 | PPARG | MRAS | hsa04810 |
| hsa-miR-27a-3p | PPARGC1A | CNR1 | ADRB2 | MRAS | hsa04015 |
| hsa-miR-27a-3p | PRKN | CNR1 | LEP | MRAS | hsa04137 |
| hsa-miR-27a-3p | PTX3 | CNR1 | PTGS2 | MRAS | hsa04014 |
| hsa-miR-27a-3p | PYGB | CNR1 | IL6 | MRAS | hsa04218 |
| hsa-miR-27a-3p | RNASE3 | CNR1 | TLR4 | MRAS | hsa04625 |
| hsa-miR-27a-3p | S100A4 | CNR1 | TNF | MRAS | hsa04371 |
| hsa-miR-27a-3p | SELPLG | CNR1 | AGTR1 | SIRT1 | hsa04152 |
| hsa-miR-27a-3p | SEMA4D | CNR1 | INS | SIRT1 | hsa04068 |
| hsa-miR-27a-3p | SERPING1 | CNR1 | BDNF | SIRT1 | hsa04218 |
| hsa-miR-27a-3p | SH2D1A | COG2 | INS | SIRT1 | hsa04211 |
| hsa-miR-27a-3p | SIRT1 | COL6A2 | MMP2 | FGF21 | hsa04010 |
| hsa-miR-27a-3p | SMAD3 | COL6A2 | ITGA2B | FGF21 | hsa04014 |
| hsa-miR-27a-3p | SULT1E1 | COL6A2 | SERPING1 | FGF21 | hsa04151 |
| hsa-miR-27a-3p | TAF1 | COL6A2 | ITGA2 | FGF21 | hsa04015 |
| hsa-miR-27a-3p | TGFB1 | COL6A2 | ADAMTS7 | FGF21 | hsa04810 |
| hsa-miR-27a-3p | THBS4 | COL6A2 | THBS4 | IL37 | hsa04061 |
| hsa-miR-27a-3p | TIMP1 | COL6A2 | ITGB3 | IL37 | hsa04060 |
| hsa-miR-27a-3p | TLR4 | CP | MIF | IL17D | hsa04060 |
| hsa-miR-27a-3p | TNC | CP | HMOX1 | IL17D | hsa04657 |
| hsa-miR-27a-3p | TNFRSF11A | CP | PON1 | IL17D | hsa04630 |
| hsa-miR-27a-3p | TNFSF14 | CP | MPO | IL17D | hsa04659 |
| hsa-miR-27a-3p | TNFSF4 | CP | HRG | IL17D | hsa04625 |
| hsa-miR-27a-3p | TNNI3 | CP | APOB | CYCS | hsa04210 |
| hsa-miR-27a-3p | TNNT1 | CP | APOA1 | CYCS | hsa05161 |
| hsa-miR-27a-3p | UBAP1 | CP | CRP | CYCS | hsa05145 |
| hsa-miR-27a-3p | VIP | CP | APCS | CYCS | hsa05131 |
| hsa-miR-27a-3p | YWHAZ | CP | TYR | CYCS | hsa05169 |
| hsa-miR-28-5p | ACE | CP | IL1B | CYCS | hsa05134 |
| hsa-miR-28-5p | COL6A2 | CP | TNF | CYCS | hsa05417 |
| hsa-miR-28-5p | IGF1 | CP | MB | CYCS | hsa05152 |
| hsa-miR-28-5p | IL34 | CP | LCN2 | CYCS | hsa05163 |
| hsa-miR-28-5p | MORF4L2 | CP | ITIH4 | CYCS | hsa05160 |
| hsa-miR-28-5p | PCSK9 | CP | FGA | CYCS | hsa05170 |
| hsa-miR-28-5p | RIPK1 | CP | AGT | CYCS | hsa05162 |
| hsa-miR-28-5p | VWF | CP | INS | CYCS | hsa05168 |
| hsa-miR-29a-3p | ANGPTL2 | CP | KNG1 | CYCS | hsa05164 |
| hsa-miR-29a-3p | BSG | CP | SERPING1 | CYCS | hsa05132 |
| hsa-miR-29a-3p | CNR1 | CP | IL6 | CYCS | hsa05167 |
| hsa-miR-29a-3p | COL6A2 | CP | SERPINC1 | CYCS | hsa04932 |
| hsa-miR-29a-3p | CXCL8 | CP | PLG | CYCS | hsa05130 |
| hsa-miR-29a-3p | CYCS | CP | F2 | CYCS | hsa05416 |
| hsa-miR-29a-3p | CYP2C19 | CP | SERPINA1 | CXCL16 | hsa04062 |
| hsa-miR-29a-3p | F10 | CPB2 | SERPINA1 | CXCL16 | hsa04060 |
| hsa-miR-29a-3p | FGA | CPB2 | SELP | FERMT3 | hsa04611 |
| hsa-miR-29a-3p | HIF1A | CPB2 | PLAU | IL33 | hsa04217 |
| hsa-miR-29a-3p | HSPA4 | CPB2 | SERPINA5 | IL33 | hsa04060 |
| hsa-miR-29a-3p | HSPD1 | CPB2 | CRP | IL33 | hsa05164 |
| hsa-miR-29a-3p | ITGA2 | CPB2 | MBL2 | IL33 | hsa04623 |
| hsa-miR-29a-3p | OLR1 | CPB2 | VWF | IL34 | hsa04061 |
| hsa-miR-29a-3p | PARP1 | CPB2 | KNG1 | IL34 | hsa04060 |
| hsa-miR-29a-3p | PTGS2 | CPB2 | F10 | PLB1 | hsa04977 |
| hsa-miR-29a-3p | PTX3 | CPB2 | HABP2 | PLB1 | hsa00591 |
| hsa-miR-29a-3p | RPS27A | CPB2 | APOB | MLKL | hsa04668 |
| hsa-miR-29a-3p | SELPLG | CPB2 | F3 | MLKL | hsa05132 |
| hsa-miR-29a-3p | SIRT1 | CPB2 | HRG | MLKL | hsa04217 |
| hsa-miR-29a-3p | TNFRSF1A | CPB2 | SERPINE1 | PCSK9 | hsa04979 |
| hsa-miR-29a-3p | TNFSF4 | CPB2 | PLAT |  |  |
| hsa-miR-29a-3p | TXNIP | CPB2 | SERPINF2 |  |  |
| hsa-miR-29a-3p | UBC | CPB2 | F7 |  |  |
| hsa-miR-29a-3p | VWF | CPB2 | FGA |  |  |
| hsa-miR-30a-5p | AHR | CPB2 | AGT |  |  |
| hsa-miR-30a-5p | APOA1 | CPB2 | SERPINC1 | |  |
| hsa-miR-30a-5p | APOB | CPB2 | PLG |  |  |
| hsa-miR-30a-5p | BSG | CPB2 | THBD |  |  |
| hsa-miR-30a-5p | CCN2 | CPB2 | F2 |  |  |
| hsa-miR-30a-5p | COG2 | CRP | CX3CL1 |  |  |
| hsa-miR-30a-5p | CXCL8 | CRP | TNFRSF1A | |  |
| hsa-miR-30a-5p | ENTPD1 | CRP | CPB2 |  |  |
| hsa-miR-30a-5p | F2 | CRP | CETP |  |  |
| hsa-miR-30a-5p | FAS | CRP | SIRT1 |  |  |
| hsa-miR-30a-5p | FST | CRP | MIF |  |  |
| hsa-miR-30a-5p | HMOX1 | CRP | HMOX1 |  |  |
| hsa-miR-30a-5p | IL1A | CRP | TIMP1 |  |  |
| hsa-miR-30a-5p | INTS2 | CRP | MMP2 |  |  |
| hsa-miR-30a-5p | ITGB3 | CRP | PLAT |  |  |
| hsa-miR-30a-5p | MON2 | CRP | RETN |  |  |
| hsa-miR-30a-5p | MORF4L2 | CRP | TGFB1 |  |  |
| hsa-miR-30a-5p | MYDGF | CRP | PON1 |  |  |
| hsa-miR-30a-5p | PARP1 | CRP | HGF |  |  |
| hsa-miR-30a-5p | PIK3C2A | CRP | NAMPT |  |  |
| hsa-miR-30a-5p | PPARGC1A | CRP | SERPINE1 |  |  |
| hsa-miR-30a-5p | PTX3 | CRP | MPO |  |  |
| hsa-miR-30a-5p | SERPINC1 | CRP | CCL2 |  |  |
| hsa-miR-30a-5p | SERPINE1 | CRP | IL2 |  |  |
| hsa-miR-30a-5p | TLR4 | CRP | IFNG |  |  |
| hsa-miR-30a-5p | TXNIP | CRP | IL5 |  |  |
| hsa-miR-30a-5p | VEGFA | CRP | APOB |  |  |
| hsa-miR-30a-3p | AHR | CRP | MMP8 |  |  |
| hsa-miR-30a-3p | FST | CRP | APOA1 |  |  |
| hsa-miR-30a-3p | HSPA4 | CRP | TNNT2 |  |  |
| hsa-miR-30a-3p | IL18R1 | CRP | EPO |  |  |
| hsa-miR-30a-3p | NFKB1 | CRP | GDF15 |  |  |
| hsa-miR-30a-3p | NRDC | CRP | LGALS3 |  |  |
| hsa-miR-30a-3p | PTGS2 | CRP | LPXN |  |  |
| hsa-miR-92a-3p | ADM | CRP | PPARGC1A | |  |
| hsa-miR-92a-3p | BSG | CRP | GP1BA |  |  |
| hsa-miR-92a-3p | CAD | CRP | MYD88 |  |  |
| hsa-miR-92a-3p | CCN2 | CRP | S100A9 |  |  |
| hsa-miR-92a-3p | CXCL16 | CRP | PECAM1 |  |  |
| hsa-miR-92a-3p | CYP2C19 | CRP | ITGA2B |  |  |
| hsa-miR-92a-3p | FASLG | CRP | HSPD1 |  |  |
| hsa-miR-92a-3p | HSPD1 | CRP | PRG2 |  |  |
| hsa-miR-92a-3p | ICAM1 | CRP | RNASE3 |  |  |
| hsa-miR-92a-3p | NFKB1 | CRP | CD28 |  |  |
| hsa-miR-92a-3p | PIK3C2A | CRP | IL33 |  |  |
| hsa-miR-92a-3p | S100A4 | CRP | PF4 |  |  |
| hsa-miR-92a-3p | SELP | CRP | PGF |  |  |
| hsa-miR-92a-3p | SERP1 | CRP | SERPING1 | |  |
| hsa-miR-92a-3p | SIRT1 | CRP | FABP3 |  |  |
| hsa-miR-92a-3p | TAF1 | CRP | AGTR1 |  |  |
| hsa-miR-92a-3p | TNFRSF11B | CRP | HLA-DRB1 | |  |
| hsa-miR-93-5p | CCL5 | CRP | SERPINF2 |  |  |
| hsa-miR-93-5p | CCL5 | CRP | FGF21 |  |  |
| hsa-miR-93-5p | CCN2 | CRP | ANGPT2 |  |  |
| hsa-miR-93-5p | CD28 | CRP | PLA2G2A |  |  |
| hsa-miR-93-5p | CSF1 | CRP | IGFBP1 |  |  |
| hsa-miR-93-5p | CYCS | CRP | PTX3 |  |  |
| hsa-miR-93-5p | FST | CRP | IL37 |  |  |
| hsa-miR-93-5p | GDF15 | CRP | IL7 |  |  |
| hsa-miR-93-5p | ICAM1 | CRP | HMGCR |  |  |
| hsa-miR-93-5p | ITGA2 | CRP | CCL11 |  |  |
| hsa-miR-93-5p | LPXN | CRP | SAA4 |  |  |
| hsa-miR-93-5p | MMP3 | CRP | IFNA1 |  |  |
| hsa-miR-93-5p | MORF4L2 | CRP | MMP1 |  |  |
| hsa-miR-93-5p | PARP1 | CRP | PCSK9 |  |  |
| hsa-miR-93-5p | SERP1 | CRP | SERPINA12 | |  |
| hsa-miR-93-5p | SERPINE1 | CRP | AGT |  |  |
| hsa-miR-93-5p | TGFB1 | CRP | BDNF |  |  |
| hsa-miR-93-5p | UBAP1 | CRP | PAPPA |  |  |
| hsa-miR-29b-3p | CNR1 | CRP | BGLAP |  |  |
| hsa-miR-29b-3p | COL6A2 | CRP | AGER |  |  |
| hsa-miR-29b-3p | CYCS | CRP | KNG1 |  |  |
| hsa-miR-29b-3p | ENTPD1 | CRP | CCL5 |  |  |
| hsa-miR-29b-3p | FGA | CRP | CCL3 |  |  |
| hsa-miR-29b-3p | HIF1A | CRP | CD163 |  |  |
| hsa-miR-29b-3p | HSPA4 | CRP | TLR2 |  |  |
| hsa-miR-29b-3p | HSPD1 | CRP | DPP4 |  |  |
| hsa-miR-29b-3p | IFNG | CRP | ADM |  |  |
| hsa-miR-29b-3p | IGF1 | CRP | PPARG |  |  |
| hsa-miR-29b-3p | IL32 | CRP | AVP |  |  |
| hsa-miR-29b-3p | MMP9 | CRP | CHI3L1 |  |  |
| hsa-miR-29b-3p | PARP1 | CRP | ITIH4 |  |  |
| hsa-miR-29b-3p | PTX3 | CRP | PTGS2 |  |  |
| hsa-miR-29b-3p | RPS27A | CRP | THBD |  |  |
| hsa-miR-29b-3p | SELPLG | CRP | TNFRSF1B | |  |
| hsa-miR-29b-3p | SIRT1 | CRP | F7 |  |  |
| hsa-miR-29b-3p | TGFB1 | CRP | MMP3 |  |  |
| hsa-miR-29b-3p | TNFRSF1A | CRP | TNNT1 |  |  |
| hsa-miR-29b-3p | UBC | CRP | IL1A |  |  |
| hsa-miR-103a-3p | ADM | CRP | TNFRSF11B | |  |
| hsa-miR-103a-3p | ANGPTL2 | CRP | CD40LG |  |  |
| hsa-miR-103a-3p | BDNF | CRP | LCN2 |  |  |
| hsa-miR-103a-3p | BRCA1 | CRP | ENTPD1 |  |  |
| hsa-miR-103a-3p | BSG | CRP | NOS3 |  |  |
| hsa-miR-103a-3p | CAD | CRP | PIK3C2A |  |  |
| hsa-miR-103a-3p | CCL2 | CRP | IGF1 |  |  |
| hsa-miR-103a-3p | CCN2 | CRP | IL1RN |  |  |
| hsa-miR-103a-3p | CD63 | CRP | MB |  |  |
| hsa-miR-103a-3p | CYCS | CRP | TLR4 |  |  |
| hsa-miR-103a-3p | EDN1 | CRP | IL17A |  |  |
| hsa-miR-103a-3p | ENHO | CRP | EDN1 |  |  |
| hsa-miR-103a-3p | ENTPD1 | CRP | MMP9 |  |  |
| hsa-miR-103a-3p | F3 | CRP | VEGFA |  |  |
| hsa-miR-103a-3p | FST | CRP | CP |  |  |
| hsa-miR-103a-3p | HDAC9 | CRP | IL18 |  |  |
| hsa-miR-103a-3p | HMOX1 | CRP | TNNI3 |  |  |
| hsa-miR-103a-3p | HSPA4 | CRP | REN |  |  |
| hsa-miR-103a-3p | IL10 | CRP | MBL2 |  |  |
| hsa-miR-103a-3p | IL18 | CRP | SERPINA1 | |  |
| hsa-miR-103a-3p | IL2RB | CRP | APCS |  |  |
| hsa-miR-103a-3p | ITGB3 | CRP | VWF |  |  |
| hsa-miR-103a-3p | LPXN | CRP | SERPINC1 | |  |
| hsa-miR-103a-3p | MIF | CRP | NPPB |  |  |
| hsa-miR-103a-3p | MMP9 | CRP | PLG |  |  |
| hsa-miR-103a-3p | MORF4L2 | CRP | VCAM1 |  |  |
| hsa-miR-103a-3p | MYD88 | CRP | ICAM1 |  |  |
| hsa-miR-103a-3p | NFKB1 | CRP | CST3 |  |  |
| hsa-miR-103a-3p | NOS3 | CRP | SELE |  |  |
| hsa-miR-103a-3p | NRDC | CRP | SELP |  |  |
| hsa-miR-103a-3p | OLR1 | CRP | F3 |  |  |
| hsa-miR-103a-3p | PIK3C2A | CRP | IL10 |  |  |
| hsa-miR-103a-3p | PLAT | CRP | ACE |  |  |
| hsa-miR-103a-3p | PLAU | CRP | CXCL8 |  |  |
| hsa-miR-103a-3p | PPARGC1A | CRP | IL1B |  |  |
| hsa-miR-103a-3p | PTGS2 | CRP | F2 |  |  |
| hsa-miR-103a-3p | SERP1 | CRP | TNF |  |  |
| hsa-miR-103a-3p | SERPINE1 | CRP | INS |  |  |
| hsa-miR-103a-3p | SULT1E1 | CRP | IL6 |  |  |
| hsa-miR-103a-3p | TGFB1 | CRP | C1S |  |  |
| hsa-miR-103a-3p | THBD | CRP | LEP |  |  |
| hsa-miR-103a-3p | TNFRSF11A | CRP | OLR1 |  |  |
| hsa-miR-103a-3p | TNFRSF1A | CRP | FCGR2A |  |  |
| hsa-miR-103a-3p | TNFRSF1B | CSF1 | CX3CL1 |  |  |
| hsa-miR-103a-3p | TNFSF4 | CSF1 | TNFRSF1A | |  |
| hsa-miR-103a-3p | UBAP1 | CSF1 | TIMP1 |  |  |
| hsa-miR-106a-5p | CCL5 | CSF1 | MMP2 |  |  |
| hsa-miR-106a-5p | CCL5 | CSF1 | TGFB1 |  |  |
| hsa-miR-106a-5p | CCN2 | CSF1 | HGF |  |  |
| hsa-miR-106a-5p | CD28 | CSF1 | CCL2 |  |  |
| hsa-miR-106a-5p | CTSS | CSF1 | IL2 |  |  |
| hsa-miR-106a-5p | CXCR4 | CSF1 | IFNG |  |  |
| hsa-miR-106a-5p | CYCS | CSF1 | IL5 |  |  |
| hsa-miR-106a-5p | FAS | CSF1 | EPO |  |  |
| hsa-miR-106a-5p | GDF15 | CSF1 | LGALS3 |  |  |
| hsa-miR-106a-5p | HIF1A | CSF1 | IL1RN |  |  |
| hsa-miR-106a-5p | IL10 | CSF1 | TLR2 |  |  |
| hsa-miR-106a-5p | IL1A | CSF1 | IL1A |  |  |
| hsa-miR-106a-5p | IL1B | CSF1 | IL1B |  |  |
| hsa-miR-106a-5p | IL6 | CSF1 | IL7 |  |  |
| hsa-miR-106a-5p | ITGA2 | CSF1 | ICAM1 |  |  |
| hsa-miR-106a-5p | LPXN | CSF1 | FCGR2A |  |  |
| hsa-miR-106a-5p | MORF4L2 | CSF1 | IFNA1 |  |  |
| hsa-miR-106a-5p | NAMPT | CSF1 | IL18 |  |  |
| hsa-miR-106a-5p | PECAM1 | CSF1 | CXCL13 |  |  |
| hsa-miR-106a-5p | RPS27A | CSF1 | PPARG |  |  |
| hsa-miR-106a-5p | SERP1 | CSF1 | VCAM1 |  |  |
| hsa-miR-106a-5p | SIRT1 | CSF1 | TNFRSF11B | |  |
| hsa-miR-106a-5p | SMAD3 | CSF1 | CCL11 |  |  |
| hsa-miR-106a-5p | TGFB1 | CSF1 | IGF1 |  |  |
| hsa-miR-106a-5p | YWHAZ | CSF1 | CD14 |  |  |
| hsa-miR-192-5p | BMP6 | CSF1 | CXCL8 |  |  |
| hsa-miR-192-5p | CCN2 | CSF1 | CD28 |  |  |
| hsa-miR-192-5p | CYCS | CSF1 | TNFRSF1B | |  |
| hsa-miR-192-5p | IL6 | CSF1 | PLAU |  |  |
| hsa-miR-192-5p | ITGB3 | CSF1 | PGF |  |  |
| hsa-miR-192-5p | SERPINE1 | CSF1 | IL33 |  |  |
| hsa-miR-192-5p | TXNIP | CSF1 | CD40LG |  |  |
| hsa-miR-192-5p | UBAP1 | CSF1 | INS |  |  |
| hsa-miR-129-5p | ADAMTS4 | CSF1 | HIF1A |  |  |
| hsa-miR-129-5p | C1S | CSF1 | PECAM1 |  |  |
| hsa-miR-129-5p | FST | CSF1 | BGLAP |  |  |
| hsa-miR-129-5p | HSPD1 | CSF1 | PTGS2 |  |  |
| hsa-miR-129-5p | IGF1 | CSF1 | TNFRSF11A | |  |
| hsa-miR-129-5p | ITGA2 | CSF1 | MMP9 |  |  |
| hsa-miR-129-5p | MBL2 | CSF1 | MYD88 |  |  |
| hsa-miR-129-5p | MON2 | CSF1 | CX3CR1 |  |  |
| hsa-miR-129-5p | PTGS2 | CSF1 | IL17A |  |  |
| hsa-miR-129-5p | TXNIP | CSF1 | TLR4 |  |  |
| hsa-miR-148a-3p | BDNF | CSF1 | CXCR4 |  |  |
| hsa-miR-148a-3p | CYCS | CSF1 | CD163 |  |  |
| hsa-miR-148a-3p | FAS | CSF1 | VEGFA |  |  |
| hsa-miR-148a-3p | HIF1A | CSF1 | CCL5 |  |  |
| hsa-miR-148a-3p | HSPD1 | CSF1 | CCL3 |  |  |
| hsa-miR-148a-3p | MYDGF | CSF1 | TEK |  |  |
| hsa-miR-148a-3p | NFKB1 | CSF1 | IL6 |  |  |
| hsa-miR-148a-3p | PAPPA | CSF1 | TNF |  |  |
| hsa-miR-148a-3p | PARP1 | CSF1 | ITGB3 |  |  |
| hsa-miR-148a-3p | PLAU | CSF1 | CD86 |  |  |
| hsa-miR-148a-3p | PTGS2 | CSF1 | IL10 |  |  |
| hsa-miR-148a-3p | VEGFA | CSF1 | IL34 |  |  |
| hsa-miR-181b-5p | ADM | CST3 | TIMP1 |  |  |
| hsa-miR-181b-5p | BRCA1 | CST3 | SERPINE1 |  |  |
| hsa-miR-181b-5p | CCL2 | CST3 | MPO |  |  |
| hsa-miR-181b-5p | CCN2 | CST3 | CCL2 |  |  |
| hsa-miR-181b-5p | FSD1L | CST3 | APOB |  |  |
| hsa-miR-181b-5p | HLA-DRB1 | CST3 | APOA1 |  |  |
| hsa-miR-181b-5p | HMGCR | CST3 | TNNT2 |  |  |
| hsa-miR-181b-5p | IL2RB | CST3 | GDF15 |  |  |
| hsa-miR-181b-5p | ITGA2 | CST3 | LGALS3 |  |  |
| hsa-miR-181b-5p | NRDC | CST3 | CRP |  |  |
| hsa-miR-181b-5p | PAPPA | CST3 | CHI3L1 |  |  |
| hsa-miR-181b-5p | PIK3C2A | CST3 | REN |  |  |
| hsa-miR-181b-5p | SERP1 | CST3 | IL18 |  |  |
| hsa-miR-181b-5p | SERPINE1 | CST3 | ACE |  |  |
| hsa-miR-181b-5p | TNFRSF11B | CST3 | FGA |  |  |
| hsa-miR-181b-5p | VCAM1 | CST3 | LEP |  |  |
| hsa-miR-181c-5p | ADM | CST3 | TNNI3 |  |  |
| hsa-miR-181c-5p | BRCA1 | CST3 | AGT |  |  |
| hsa-miR-181c-5p | CCN2 | CST3 | CTSS |  |  |
| hsa-miR-181c-5p | FSD1L | CST3 | MMP9 |  |  |
| hsa-miR-181c-5p | HLCS | CST3 | LCN2 |  |  |
| hsa-miR-181c-5p | HMGCR | CST3 | NPPB |  |  |
| hsa-miR-181c-5p | IL2RB | CST3 | AVP |  |  |
| hsa-miR-181c-5p | IL7 | CST3 | INS |  |  |
| hsa-miR-181c-5p | ITGA2 | CST3 | MB |  |  |
| hsa-miR-181c-5p | PTGS2 | CST3 | VEGFA |  |  |
| hsa-miR-181c-5p | SIRT1 | CST3 | ADM |  |  |
| hsa-miR-181c-5p | VCAM1 | CST3 | TNNT1 |  |  |
| hsa-miR-181c-5p | VEGFA | CST3 | TNF |  |  |
| hsa-miR-205-5p | BDNF | CST3 | SERPINA1 | |  |
| hsa-miR-205-5p | BRCA1 | CST3 | IL6 |  |  |
| hsa-miR-205-5p | CTSS | CTGF | HMOX1 |  |  |
| hsa-miR-205-5p | CXCL16 | CTGF | TIMP1 |  |  |
| hsa-miR-205-5p | HMGCR | CTGF | MMP2 |  |  |
| hsa-miR-205-5p | HMOX1 | CTGF | PLAT |  |  |
| hsa-miR-205-5p | IL32 | CTGF | TGFB1 |  |  |
| hsa-miR-205-5p | IL6 | CTGF | HGF |  |  |
| hsa-miR-205-5p | MMP1 | CTGF | SERPINE1 |  |  |
| hsa-miR-205-5p | NFKB1 | CTGF | CCL2 |  |  |
| hsa-miR-205-5p | NOS3 | CTGF | MMP8 |  |  |
| hsa-miR-205-5p | PLB1 | CTGF | GDF15 |  |  |
| hsa-miR-205-5p | PYGB | CTGF | LGALS3 |  |  |
| hsa-miR-205-5p | SERPINA1 | CTGF | FST |  |  |
| hsa-miR-205-5p | SERPINE1 | CTGF | VWF |  |  |
| hsa-miR-205-5p | TLR2 | CTGF | IL1B |  |  |
| hsa-miR-205-5p | TNFSF4 | CTGF | ICAM1 |  |  |
| hsa-miR-211-5p | ANGPTL2 | CTGF | REN |  |  |
| hsa-miR-211-5p | CD28 | CTGF | BMP6 |  |  |
| hsa-miR-211-5p | HLA-DRB1 | CTGF | PPARG |  |  |
| hsa-miR-211-5p | HLA-DRB1 | CTGF | ACE |  |  |
| hsa-miR-211-5p | HLA-DRB1 | CTGF | VCAM1 |  |  |
| hsa-miR-211-5p | HLA-DRB1 | CTGF | TNFRSF11B | |  |
| hsa-miR-211-5p | HLA-DRB1 | CTGF | NOS3 |  |  |
| hsa-miR-211-5p | HLA-DRB1 | CTGF | MMP3 |  |  |
| hsa-miR-211-5p | HLCS | CTGF | IGF1 |  |  |
| hsa-miR-211-5p | MMP9 | CTGF | CXCL8 |  |  |
| hsa-miR-211-5p | SERPINC1 | CTGF | PLG |  |  |
| hsa-miR-211-5p | SERPINF2 | CTGF | MMP1 |  |  |
| hsa-miR-211-5p | SERPINF2 | CTGF | SMAD3 |  |  |
| hsa-miR-211-5p | TGFB1 | CTGF | AGT |  |  |
| hsa-miR-211-5p | TXNIP | CTGF | PTGS2 |  |  |
| hsa-miR-212-3p | ANGPT1 | CTGF | CCL5 |  |  |
| hsa-miR-212-3p | BRCA1 | CTGF | TEK |  |  |
| hsa-miR-212-3p | CCL5 | CTGF | PLAU |  |  |
| hsa-miR-212-3p | CCN2 | CTGF | ADAMTS7 | |  |
| hsa-miR-212-3p | CHIT1 | CTGF | IL10 |  |  |
| hsa-miR-212-3p | CXCL8 | CTGF | CXCR4 |  |  |
| hsa-miR-212-3p | EDN1 | CTGF | PECAM1 |  |  |
| hsa-miR-212-3p | F2 | CTGF | AGTR1 |  |  |
| hsa-miR-212-3p | FSD1L | CTGF | S100A4 |  |  |
| hsa-miR-212-3p | HMGCR | CTGF | NPPB |  |  |
| hsa-miR-212-3p | IL32 | CTGF | INS |  |  |
| hsa-miR-212-3p | IL5 | CTGF | HIF1A |  |  |
| hsa-miR-212-3p | IL7 | CTGF | TNF |  |  |
| hsa-miR-212-3p | NPPB | CTGF | IL6 |  |  |
| hsa-miR-212-3p | PRKN | CTGF | MMP9 |  |  |
| hsa-miR-212-3p | PTGS2 | CTGF | EDN1 |  |  |
| hsa-miR-212-3p | RIPK3 | CTGF | ITGB2 |  |  |
| hsa-miR-212-3p | SELENOS | CTGF | VEGFA |  |  |
| hsa-miR-212-3p | SERP1 | CTSS | TLR2 |  |  |
| hsa-miR-212-3p | SERPINA1 | CTSS | CYBA |  |  |
| hsa-miR-212-3p | TIMP1 | CTSS | FCGR2A |  |  |
| hsa-miR-212-3p | TNC | CTSS | VCAM1 |  |  |
| hsa-miR-212-3p | TNFSF4 | CTSS | MMP3 |  |  |
| hsa-miR-212-3p | TNNT1 | CTSS | CD14 |  |  |
| hsa-miR-200b-3p | ADM | CTSS | FGA |  |  |
| hsa-miR-200b-3p | BRCA1 | CTSS | CD86 |  |  |
| hsa-miR-200b-3p | CXCR4 | CTSS | HLA-DRB1 | |  |
| hsa-miR-200b-3p | DPP4 | CTSS | S100A8 |  |  |
| hsa-miR-200b-3p | EDN1 | CTSS | S100A9 |  |  |
| hsa-miR-200b-3p | FSD1L | CTSS | TNF |  |  |
| hsa-miR-200b-3p | INS | CTSS | TLR4 |  |  |
| hsa-miR-200b-3p | LCN2 | CTSS | MMP9 |  |  |
| hsa-miR-200b-3p | MALAT1 | CTSS | CST3 |  |  |
| hsa-miR-200b-3p | MMP28 | CTSS | ITGB2 |  |  |
| hsa-miR-200b-3p | MMP8 | CX3CL1 | CD163 |  |  |
| hsa-miR-200b-3p | MRAS | CX3CL1 | IFNA1 |  |  |
| hsa-miR-200b-3p | NAMPT | CX3CL1 | GDF15 |  |  |
| hsa-miR-200b-3p | PLB1 | CX3CL1 | PECAM1 |  |  |
| hsa-miR-200b-3p | PPBP | CX3CL1 | LEP |  |  |
| hsa-miR-200b-3p | PTX3 | CX3CL1 | PTGS2 |  |  |
| hsa-miR-200b-3p | S100A4 | CX3CL1 | CRP |  |  |
| hsa-miR-200b-3p | SELENOS | CX3CL1 | IGF1 |  |  |
| hsa-miR-200b-3p | SH2D1A | CX3CL1 | PPBP |  |  |
| hsa-miR-200b-3p | SIRT1 | CX3CL1 | CD40LG |  |  |
| hsa-miR-200b-3p | SMAD3 | CX3CL1 | ITGB2 |  |  |
| hsa-let-7g-5p | ADRB2 | CX3CL1 | SELP |  |  |
| hsa-let-7g-5p | AHR | CX3CL1 | TLR2 |  |  |
| hsa-let-7g-5p | BSG | CX3CL1 | CD86 |  |  |
| hsa-let-7g-5p | CXCL8 | CX3CL1 | TIMP1 |  |  |
| hsa-let-7g-5p | FAS | CX3CL1 | IL34 |  |  |
| hsa-let-7g-5p | MON2 | CX3CL1 | PF4 |  |  |
| hsa-let-7g-5p | PAPPA | CX3CL1 | MMP9 |  |  |
| hsa-let-7g-5p | PARP1 | CX3CL1 | SELE |  |  |
| hsa-let-7g-5p | PTGS2 | CX3CL1 | TLR4 |  |  |
| hsa-let-7g-5p | SERP1 | CX3CL1 | CXCL13 |  |  |
| hsa-let-7g-5p | TLR4 | CX3CL1 | CSF1 |  |  |
| hsa-let-7g-5p | TNFRSF11A | CX3CL1 | IL7 |  |  |
| hsa-let-7g-5p | VCAM1 | CX3CL1 | IL18 |  |  |
| hsa-let-7i-5p | ADM | CX3CL1 | IL2 |  |  |
| hsa-let-7i-5p | ADRB2 | CX3CL1 | IL5 |  |  |
| hsa-let-7i-5p | AHR | CX3CL1 | IFNG |  |  |
| hsa-let-7i-5p | BSG | CX3CL1 | IL17A |  |  |
| hsa-let-7i-5p | CXCL8 | CX3CL1 | CXCL16 |  |  |
| hsa-let-7i-5p | FAS | CX3CL1 | ICAM1 |  |  |
| hsa-let-7i-5p | IGF1 | CX3CL1 | BDNF |  |  |
| hsa-let-7i-5p | IL2 | CX3CL1 | VCAM1 |  |  |
| hsa-let-7i-5p | MON2 | CX3CL1 | IL1A |  |  |
| hsa-let-7i-5p | PAPPA | CX3CL1 | ITGB3 |  |  |
| hsa-let-7i-5p | PARP1 | CX3CL1 | IL10 |  |  |
| hsa-let-7i-5p | PIK3C2A | CX3CL1 | VEGFA |  |  |
| hsa-let-7i-5p | PTGS2 | CX3CL1 | CCL11 |  |  |
| hsa-let-7i-5p | SERP1 | CX3CL1 | CXCL8 |  |  |
| hsa-let-7i-5p | TNFRSF11A | CX3CL1 | IL6 |  |  |
| hsa-miR-23b-3p | ADRB2 | CX3CL1 | IL1B |  |  |
| hsa-miR-23b-3p | AHR | CX3CL1 | TNF |  |  |
| hsa-miR-23b-3p | CCN2 | CX3CL1 | CCL3 |  |  |
| hsa-miR-23b-3p | CSF1 | CX3CL1 | CCL5 |  |  |
| hsa-miR-23b-3p | CXCL8 | CX3CL1 | CCL2 |  |  |
| hsa-miR-23b-3p | F3 | CX3CL1 | CXCR4 |  |  |
| hsa-miR-23b-3p | FSD1L | CX3CL1 | CX3CR1 |  |  |
| hsa-miR-23b-3p | GAS6 | CX3CR1 | CX3CL1 |  |  |
| hsa-miR-23b-3p | GDF15 | CX3CR1 | GZMB |  |  |
| hsa-miR-23b-3p | HABP2 | CX3CR1 | CCL2 |  |  |
| hsa-miR-23b-3p | HIF1A | CX3CR1 | IL2 |  |  |
| hsa-miR-23b-3p | HMGCR | CX3CR1 | SELPLG |  |  |
| hsa-miR-23b-3p | HMOX1 | CX3CR1 | IFNG |  |  |
| hsa-miR-23b-3p | HSPD1 | CX3CR1 | TLR2 |  |  |
| hsa-miR-23b-3p | IGFBP1 | CX3CR1 | IL1A |  |  |
| hsa-miR-23b-3p | IL17D | CX3CR1 | IL1B |  |  |
| hsa-miR-23b-3p | ITGA2 | CX3CR1 | SELP |  |  |
| hsa-miR-23b-3p | LGALS3 | CX3CR1 | ICAM1 |  |  |
| hsa-miR-23b-3p | MMP1 | CX3CR1 | FCGR2A |  |  |
| hsa-miR-23b-3p | MMP2 | CX3CR1 | IL18 |  |  |
| hsa-miR-23b-3p | MMP9 | CX3CR1 | CXCL13 |  |  |
| hsa-miR-23b-3p | MORF4L2 | CX3CR1 | CXCL16 |  |  |
| hsa-miR-23b-3p | MYD88 | CX3CR1 | VCAM1 |  |  |
| hsa-miR-23b-3p | MYDGF | CX3CR1 | PPBP |  |  |
| hsa-miR-23b-3p | NAMPT | CX3CR1 | PF4 |  |  |
| hsa-miR-23b-3p | NRDC | CX3CR1 | CCL11 |  |  |
| hsa-miR-23b-3p | PARP1 | CX3CR1 | IGF1 |  |  |
| hsa-miR-23b-3p | PECAM1 | CX3CR1 | CD14 |  |  |
| hsa-miR-23b-3p | PIK3C2A | CX3CR1 | CXCL8 |  |  |
| hsa-miR-23b-3p | PLAT | CX3CR1 | CD28 |  |  |
| hsa-miR-23b-3p | PTX3 | CX3CR1 | CSF1 |  |  |
| hsa-miR-23b-3p | PYGB | CX3CR1 | CD86 |  |  |
| hsa-miR-23b-3p | RIPK1 | CX3CR1 | IL17A |  |  |
| hsa-miR-23b-3p | RPS27A | CX3CR1 | CD40LG |  |  |
| hsa-miR-23b-3p | S100A4 | CX3CR1 | MMP9 |  |  |
| hsa-miR-23b-3p | SELENOS | CX3CR1 | MYD88 |  |  |
| hsa-miR-23b-3p | SELP | CX3CR1 | ITGB2 |  |  |
| hsa-miR-23b-3p | SELPLG | CX3CR1 | HIF1A |  |  |
| hsa-miR-23b-3p | SERP1 | CX3CR1 | BDNF |  |  |
| hsa-miR-23b-3p | SERPINE1 | CX3CR1 | VEGFA |  |  |
| hsa-miR-23b-3p | SIRT1 | CX3CR1 | PECAM1 |  |  |
| hsa-miR-23b-3p | SMAD3 | CX3CR1 | CD163 |  |  |
| hsa-miR-23b-3p | TGFB1 | CX3CR1 | IL34 |  |  |
| hsa-miR-23b-3p | THBD | CX3CR1 | TLR4 |  |  |
| hsa-miR-23b-3p | TNC | CX3CR1 | IL6 |  |  |
| hsa-miR-23b-3p | TNFSF4 | CX3CR1 | IL10 |  |  |
| hsa-miR-23b-3p | TNNT1 | CX3CR1 | TNF |  |  |
| hsa-miR-23b-3p | VCAM1 | CX3CR1 | CCL5 |  |  |
| hsa-miR-23b-3p | VEGFA | CX3CR1 | CCL3 |  |  |
| hsa-miR-23b-3p | VIP | CXCL13 | CX3CL1 |  |  |
| hsa-miR-23b-3p | YWHAZ | CXCL13 | TNFRSF1A | |  |
| hsa-miR-27b-3p | ANGPT1 | CXCL13 | GZMB |  |  |
| hsa-miR-27b-3p | CCL5 | CXCL13 | TIMP1 |  |  |
| hsa-miR-27b-3p | CNR1 | CXCL13 | CCL2 |  |  |
| hsa-miR-27b-3p | CSF1 | CXCL13 | IL2 |  |  |
| hsa-miR-27b-3p | CX3CL1 | CXCL13 | IFNG |  |  |
| hsa-miR-27b-3p | CXCL16 | CXCL13 | IL5 |  |  |
| hsa-miR-27b-3p | F3 | CXCL13 | CHI3L1 |  |  |
| hsa-miR-27b-3p | FST | CXCL13 | TLR2 |  |  |
| hsa-miR-27b-3p | HDAC9 | CXCL13 | IL1A |  |  |
| hsa-miR-27b-3p | HIF1A | CXCL13 | IL1B |  |  |
| hsa-miR-27b-3p | HMGCR | CXCL13 | IL7 |  |  |
| hsa-miR-27b-3p | HSPD1 | CXCL13 | ICAM1 |  |  |
| hsa-miR-27b-3p | IL10 | CXCL13 | IFNA1 |  |  |
| hsa-miR-27b-3p | MMP1 | CXCL13 | IL18 |  |  |
| hsa-miR-27b-3p | MYDGF | CXCL13 | IL33 |  |  |
| hsa-miR-27b-3p | NRDC | CXCL13 | VEGFA |  |  |
| hsa-miR-27b-3p | PAPPA | CXCL13 | TLR4 |  |  |
| hsa-miR-27b-3p | PARP1 | CXCL13 | CD163 |  |  |
| hsa-miR-27b-3p | PPARGC1A | CXCL13 | LEP |  |  |
| hsa-miR-27b-3p | SIRT1 | CXCL13 | MMP9 |  |  |
| hsa-miR-27b-3p | TAF1 | CXCL13 | CSF1 |  |  |
| hsa-miR-27b-3p | TNC | CXCL13 | PPBP |  |  |
| hsa-miR-27b-3p | UBAP1 | CXCL13 | CD86 |  |  |
| hsa-miR-27b-3p | VEGFA | CXCL13 | VCAM1 |  |  |
| hsa-miR-27b-3p | VIP | CXCL13 | CXCL16 |  |  |
| hsa-miR-122-5p | ARSA | CXCL13 | CD40LG |  |  |
| hsa-miR-122-5p | CXCR4 | CXCL13 | IL17A |  |  |
| hsa-miR-122-5p | CYCS | CXCL13 | CCL11 |  |  |
| hsa-miR-122-5p | ENTPD1 | CXCL13 | IL6 |  |  |
| hsa-miR-122-5p | F2 | CXCL13 | TNF |  |  |
| hsa-miR-122-5p | GDF15 | CXCL13 | IL10 |  |  |
| hsa-miR-122-5p | IL12A | CXCL13 | CCL5 |  |  |
| hsa-miR-122-5p | IL1A | CXCL13 | CCL3 |  |  |
| hsa-miR-122-5p | IL2RA | CXCL13 | CX3CR1 |  |  |
| hsa-miR-122-5p | IL6 | CXCL13 | PF4 |  |  |
| hsa-miR-122-5p | MORF4L2 | CXCL13 | CXCR4 |  |  |
| hsa-miR-122-5p | NRDC | CXCL16 | CX3CL1 |  |  |
| hsa-miR-122-5p | PAPPA | CXCL16 | CCL2 |  |  |
| hsa-miR-122-5p | PGF | CXCL16 | APOA1 |  |  |
| hsa-miR-122-5p | PIK3C2A | CXCL16 | TLR2 |  |  |
| hsa-miR-122-5p | PLAT | CXCL16 | IL1B |  |  |
| hsa-miR-122-5p | SEMA4D | CXCL16 | ICAM1 |  |  |
| hsa-miR-122-5p | TGFB1 | CXCL16 | IL18 |  |  |
| hsa-miR-122-5p | TNFSF14 | CXCL16 | CXCL13 |  |  |
| hsa-miR-132-3p | ANGPT1 | CXCL16 | MMP9 |  |  |
| hsa-miR-132-3p | BDNF | CXCL16 | CCL11 |  |  |
| hsa-miR-132-3p | CCN2 | CXCL16 | VCAM1 |  |  |
| hsa-miR-132-3p | CHGA | CXCL16 | IL10 |  |  |
| hsa-miR-132-3p | CNR1 | CXCL16 | PPBP |  |  |
| hsa-miR-132-3p | FGF21 | CXCL16 | BSG |  |  |
| hsa-miR-132-3p | HMGCR | CXCL16 | PF4 |  |  |
| hsa-miR-132-3p | MMP9 | CXCL16 | IL6 |  |  |
| hsa-miR-132-3p | PLAU | CXCL16 | OLR1 |  |  |
| hsa-miR-132-3p | SERP1 | CXCL16 | TNF |  |  |
| hsa-miR-140-5p | ENTPD1 | CXCL16 | CXCL8 |  |  |
| hsa-miR-140-5p | HIF1A | CXCL16 | CCL3 |  |  |
| hsa-miR-140-5p | LGALS3 | CXCL16 | CCL5 |  |  |
| hsa-miR-140-5p | NAMPT | CXCL16 | CX3CR1 |  |  |
| hsa-miR-140-5p | SELPLG | CXCL16 | CXCR4 |  |  |
| hsa-miR-140-5p | SERP1 | CXCL8 | CX3CL1 |  |  |
| hsa-miR-140-5p | SERPINE1 | CXCL8 | TNFRSF1A | |  |
| hsa-miR-140-5p | SMAD3 | CXCL8 | SIRT1 |  |  |
| hsa-miR-140-5p | TGFB1 | CXCL8 | MIF |  |  |
| hsa-miR-143-3p | ADAMTS4 | CXCL8 | HMOX1 |  |  |
| hsa-miR-143-3p | AHR | CXCL8 | GZMB |  |  |
| hsa-miR-143-3p | CCN2 | CXCL8 | TIMP1 |  |  |
| hsa-miR-143-3p | CD28 | CXCL8 | MMP2 |  |  |
| hsa-miR-143-3p | CNR1 | CXCL8 | PLAT |  |  |
| hsa-miR-143-3p | CYCS | CXCL8 | RETN |  |  |
| hsa-miR-143-3p | MMP9 | CXCL8 | TGFB1 |  |  |
| hsa-miR-143-3p | PAPPA | CXCL8 | HGF |  |  |
| hsa-miR-143-3p | PLAU | CXCL8 | NAMPT |  |  |
| hsa-miR-143-3p | SMAD3 | CXCL8 | SERPINE1 |  |  |
| hsa-miR-143-3p | TLR2 | CXCL8 | MPO |  |  |
| hsa-miR-143-3p | TNF | CXCL8 | CCL2 |  |  |
| hsa-miR-143-3p | TNF | CXCL8 | NFKB1 |  |  |
| hsa-miR-143-3p | TNF | CXCL8 | IL2 |  |  |
| hsa-miR-143-3p | TNF | CXCL8 | SELPLG |  |  |
| hsa-miR-143-3p | TNF | CXCL8 | IFNG |  |  |
| hsa-miR-143-3p | TNF | CXCL8 | IL5 |  |  |
| hsa-miR-143-3p | TNF | CXCL8 | APOB |  |  |
| hsa-miR-143-3p | TNF | CXCL8 | MMP8 |  |  |
| hsa-miR-191-5p | ADM | CXCL8 | APOA1 |  |  |
| hsa-miR-191-5p | AGER | CXCL8 | AHR |  |  |
| hsa-miR-191-5p | AGT | CXCL8 | EPO |  |  |
| hsa-miR-191-5p | AHR | CXCL8 | GDF15 |  |  |
| hsa-miR-191-5p | ANGPTL2 | CXCL8 | LGALS3 |  |  |
| hsa-miR-191-5p | APOA1 | CXCL8 | CRP |  |  |
| hsa-miR-191-5p | APOB | CXCL8 | CHI3L1 |  |  |
| hsa-miR-191-5p | CCN2 | CXCL8 | IL1RN |  |  |
| hsa-miR-191-5p | CP | CXCL8 | TLR2 |  |  |
| hsa-miR-191-5p | CST3 | CXCL8 | VWF |  |  |
| hsa-miR-191-5p | CYBA | CXCL8 | IL37 |  |  |
| hsa-miR-191-5p | CYCS | CXCL8 | IL1A |  |  |
| hsa-miR-191-5p | EDN1 | CXCL8 | IL1B |  |  |
| hsa-miR-191-5p | F10 | CXCL8 | SELP |  |  |
| hsa-miR-191-5p | FERMT3 | CXCL8 | IL7 |  |  |
| hsa-miR-191-5p | FGF21 | CXCL8 | ICAM1 |  |  |
| hsa-miR-191-5p | FST | CXCL8 | KNG1 |  |  |
| hsa-miR-191-5p | GDF15 | CXCL8 | ITIH4 |  |  |
| hsa-miR-191-5p | HMOX1 | CXCL8 | FCGR2A |  |  |
| hsa-miR-191-5p | ICAM1 | CXCL8 | REN |  |  |
| hsa-miR-191-5p | IGFBP1 | CXCL8 | IGFBP1 |  |  |
| hsa-miR-191-5p | IL18 | CXCL8 | IFNA1 |  |  |
| hsa-miR-191-5p | IL1RN | CXCL8 | IL18 |  |  |
| hsa-miR-191-5p | IL6 | CXCL8 | PPARG |  |  |
| hsa-miR-191-5p | INTS2 | CXCL8 | ACE |  |  |
| hsa-miR-191-5p | ITGA2 | CXCL8 | CXCL16 |  |  |
| hsa-miR-191-5p | LPXN | CXCL8 | VCAM1 |  |  |
| hsa-miR-191-5p | MIF | CXCL8 | PTX3 |  |  |
| hsa-miR-191-5p | MORF4L2 | CXCL8 | PPBP |  |  |
| hsa-miR-191-5p | NFKB1 | CXCL8 | PF4 |  |  |
| hsa-miR-191-5p | NPPB | CXCL8 | TNFRSF11B | |  |
| hsa-miR-191-5p | OLR1 | CXCL8 | NOS3 |  |  |
| hsa-miR-191-5p | PCSK9 | CXCL8 | MMP3 |  |  |
| hsa-miR-191-5p | PLA2G2A | CXCL8 | CCL11 |  |  |
| hsa-miR-191-5p | PLAU | CXCL8 | RNASE3 |  |  |
| hsa-miR-191-5p | PTGS2 | CXCL8 | IGF1 |  |  |
| hsa-miR-191-5p | PYGB | CXCL8 | IL17D |  |  |
| hsa-miR-191-5p | S100A4 | CXCL8 | HSPA4 |  |  |
| hsa-miR-191-5p | SELENOS | CXCL8 | IL12A |  |  |
| hsa-miR-191-5p | SERP1 | CXCL8 | CD14 |  |  |
| hsa-miR-191-5p | SERPINE1 | CXCL8 | ADRB2 |  |  |
| hsa-miR-191-5p | SERPINF2 | CXCL8 | CD63 |  |  |
| hsa-miR-191-5p | SMAD3 | CXCL8 | HSPD1 |  |  |
| hsa-miR-191-5p | TNFSF4 | CXCL8 | BGLAP |  |  |
| hsa-miR-191-5p | TXNIP | CXCL8 | ADAMTS4 | |  |
| hsa-miR-191-5p | UBAP1 | CXCL8 | ADM |  |  |
| hsa-miR-191-5p | UBC | CXCL8 | FGF21 |  |  |
| hsa-miR-191-5p | VEGFA | CXCL8 | S100A4 |  |  |
| hsa-miR-126-3p | ADM | CXCL8 | OLR1 |  |  |
| hsa-miR-126-3p | ANGPT2 | CXCL8 | MBL2 |  |  |
| hsa-miR-126-3p | BSG | CXCL8 | SMAD3 |  |  |
| hsa-miR-126-3p | CXCL8 | CXCL8 | FAS |  |  |
| hsa-miR-126-3p | CXCR4 | CXCL8 | CYCS |  |  |
| hsa-miR-126-3p | HGF | CXCL8 | THBS4 |  |  |
| hsa-miR-126-3p | IL1B | CXCL8 | SERPINC1 | |  |
| hsa-miR-126-3p | IL1RN | CXCL8 | BDNF |  |  |
| hsa-miR-126-3p | MPO | CXCL8 | TEK |  |  |
| hsa-miR-126-3p | NAMPT | CXCL8 | S100A9 |  |  |
| hsa-miR-126-3p | NRDC | CXCL8 | ITGB2 |  |  |
| hsa-miR-126-3p | PPARG | CXCL8 | F2 |  |  |
| hsa-miR-126-3p | S100A4 | CXCL8 | CTGF |  |  |
| hsa-miR-126-3p | S100A8 | CXCL8 | FASLG |  |  |
| hsa-miR-126-3p | S100A9 | CXCL8 | CD28 |  |  |
| hsa-miR-126-3p | SELP | CXCL8 | TNFRSF1B | |  |
| hsa-miR-126-3p | SERPINA1 | CXCL8 | ANGPT1 |  |  |
| hsa-miR-126-3p | SH2D1A | CXCL8 | PLAU |  |  |
| hsa-miR-126-3p | SIRT1 | CXCL8 | THBD |  |  |
| hsa-miR-126-3p | TEK | CXCL8 | IL2RA |  |  |
| hsa-miR-126-3p | TNFSF14 | CXCL8 | CD163 |  |  |
| hsa-miR-146a-5p | ADAMTS4 | CXCL8 | PLG |  |  |
| hsa-miR-146a-5p | ADAMTS7 | CXCL8 | IL32 |  |  |
| hsa-miR-146a-5p | AVP | CXCL8 | LCN2 |  |  |
| hsa-miR-146a-5p | CCL5 | CXCL8 | PGF |  |  |
| hsa-miR-146a-5p | CCL5 | CXCL8 | AGER |  |  |
| hsa-miR-146a-5p | CCN2 | CXCL8 | PECAM1 |  |  |
| hsa-miR-146a-5p | CD28 | CXCL8 | EDN1 |  |  |
| hsa-miR-146a-5p | CD40LG | CXCL8 | F3 |  |  |
| hsa-miR-146a-5p | CD86 | CXCL8 | ANGPT2 |  |  |
| hsa-miR-146a-5p | COL6A2 | CXCL8 | LEP |  |  |
| hsa-miR-146a-5p | CPB2 | CXCL8 | IL33 |  |  |
| hsa-miR-146a-5p | CST3 | CXCL8 | CD40LG |  |  |
| hsa-miR-146a-5p | CXCL16 | CXCL8 | HIF1A |  |  |
| hsa-miR-146a-5p | CYCS | CXCL8 | INS |  |  |
| hsa-miR-146a-5p | DPP4 | CXCL8 | CD86 |  |  |
| hsa-miR-146a-5p | EBI3 | CXCL8 | AGTR1 |  |  |
| hsa-miR-146a-5p | ENHO | CXCL8 | S100A8 |  |  |
| hsa-miR-146a-5p | F10 | CXCL8 | CSF1 |  |  |
| hsa-miR-146a-5p | F3 | CXCL8 | MMP1 |  |  |
| hsa-miR-146a-5p | FGF21 | CXCL8 | MYD88 |  |  |
| hsa-miR-146a-5p | FSD1L | CXCL8 | SELE |  |  |
| hsa-miR-146a-5p | GAS6 | CXCL8 | SERPINA1 | |  |
| hsa-miR-146a-5p | GDF15 | CXCL8 | CX3CR1 |  |  |
| hsa-miR-146a-5p | GP1BA | CXCL8 | MMP9 |  |  |
| hsa-miR-146a-5p | GZMB | CXCL8 | VEGFA |  |  |
| hsa-miR-146a-5p | IL18 | CXCL8 | IL17A |  |  |
| hsa-miR-146a-5p | IL32 | CXCL8 | TLR4 |  |  |
| hsa-miR-146a-5p | IL33 | CXCL8 | CXCR4 |  |  |
| hsa-miR-146a-5p | IL6 | CXCL8 | PTGS2 |  |  |
| hsa-miR-146a-5p | ITGA2 | CXCL8 | CCL5 |  |  |
| hsa-miR-146a-5p | ITGA2B | CXCL8 | CCL3 |  |  |
| hsa-miR-146a-5p | LCN2 | CXCL8 | TNF |  |  |
| hsa-miR-146a-5p | MIF | CXCL8 | IL10 |  |  |
| hsa-miR-146a-5p | MIF | CXCL8 | IL6 |  |  |
| hsa-miR-146a-5p | MMP28 | CXCR4 | CX3CL1 |  |  |
| hsa-miR-146a-5p | MMP9 | CXCR4 | TNFRSF1A | |  |
| hsa-miR-146a-5p | MORF4L2 | CXCR4 | MIF |  |  |
| hsa-miR-146a-5p | NFKB1 | CXCR4 | GZMB |  |  |
| hsa-miR-146a-5p | NOS3 | CXCR4 | TIMP1 |  |  |
| hsa-miR-146a-5p | NPPB | CXCR4 | MMP2 |  |  |
| hsa-miR-146a-5p | PIK3C2A | CXCR4 | TGFB1 |  |  |
| hsa-miR-146a-5p | PLAU | CXCR4 | HGF |  |  |
| hsa-miR-146a-5p | PRG2 | CXCR4 | CCL2 |  |  |
| hsa-miR-146a-5p | REN | CXCR4 | IL2 |  |  |
| hsa-miR-146a-5p | RETN | CXCR4 | SELPLG |  |  |
| hsa-miR-146a-5p | RNASE3 | CXCR4 | IFNG |  |  |
| hsa-miR-146a-5p | S100A4 | CXCR4 | IL5 |  |  |
| hsa-miR-146a-5p | SCUBE1 | CXCR4 | EPO |  |  |
| hsa-miR-146a-5p | SELP | CXCR4 | TLR2 |  |  |
| hsa-miR-146a-5p | SERPINA12 | CXCR4 | VWF |  |  |
| hsa-miR-146a-5p | SERPINA5 | CXCR4 | ITGA2B |  |  |
| hsa-miR-146a-5p | SERPINF2 | CXCR4 | IL1A |  |  |
| hsa-miR-146a-5p | TGFB1 | CXCR4 | IL1B |  |  |
| hsa-miR-146a-5p | THBS4 | CXCR4 | SELP |  |  |
| hsa-miR-146a-5p | TIMP1 | CXCR4 | IL7 |  |  |
| hsa-miR-146a-5p | TLR4 | CXCR4 | ICAM1 |  |  |
| hsa-miR-146a-5p | TNFRSF1A | CXCR4 | ITIH4 |  |  |
| hsa-miR-146a-5p | TNFRSF1B | CXCR4 | FCGR2A |  |  |
| hsa-miR-146a-5p | TNFSF4 | CXCR4 | IFNA1 |  |  |
| hsa-miR-146a-5p | TNNI1 | CXCR4 | IL18 |  |  |
| hsa-miR-146a-5p | TNNI3 | CXCR4 | CXCL13 |  |  |
| hsa-miR-146a-5p | TNNT1 | CXCR4 | PPARG |  |  |
| hsa-miR-146a-5p | TREML1 | CXCR4 | CXCL16 |  |  |
| hsa-miR-146a-5p | TXNIP | CXCR4 | VCAM1 |  |  |
| hsa-miR-146a-5p | VEGFA | CXCR4 | PPBP |  |  |
| hsa-miR-154-3p | HSPD1 | CXCR4 | PF4 |  |  |
| hsa-miR-154-3p | NAMPT | CXCR4 | NOS3 |  |  |
| hsa-miR-154-3p | PLAT | CXCR4 | MMP3 |  |  |
| hsa-miR-154-3p | PTX3 | CXCR4 | CCL11 |  |  |
| hsa-miR-186-5p | BRCA1 | CXCR4 | IGF1 |  |  |
| hsa-miR-186-5p | BSG | CXCR4 | HSPA4 |  |  |
| hsa-miR-186-5p | CAD | CXCR4 | ADRB2 |  |  |
| hsa-miR-186-5p | COG2 | CXCR4 | CXCL8 |  |  |
| hsa-miR-186-5p | CYCS | CXCR4 | F2 |  |  |
| hsa-miR-186-5p | HMGCR | CXCR4 | PLG |  |  |
| hsa-miR-186-5p | IL1A | CXCR4 | ANGPT2 |  |  |
| hsa-miR-186-5p | LPXN | CXCR4 | MMP1 |  |  |
| hsa-miR-186-5p | OLR1 | CXCR4 | CD28 |  |  |
| hsa-miR-186-5p | PIK3C2A | CXCR4 | CSF1 |  |  |
| hsa-miR-186-5p | RIPK1 | CXCR4 | SELE |  |  |
| hsa-miR-186-5p | SERP1 | CXCR4 | CD86 |  |  |
| hsa-miR-186-5p | SERPINA12 | CXCR4 | IL17A |  |  |
| hsa-miR-186-5p | TLR4 | CXCR4 | FAS |  |  |
| hsa-miR-186-5p | TNC | CXCR4 | CD163 |  |  |
| hsa-miR-186-5p | TXNIP | CXCR4 | DPP4 |  |  |
| hsa-miR-186-5p | VCAM1 | CXCR4 | AGT |  |  |
| hsa-miR-186-5p | YWHAZ | CXCR4 | PTGS2 |  |  |
| hsa-miR-193a-3p | CCN2 | CXCR4 | FASLG |  |  |
| hsa-miR-193a-3p | FST | CXCR4 | CTGF |  |  |
| hsa-miR-193a-3p | HIF1A | CXCR4 | S100A4 |  |  |
| hsa-miR-193a-3p | PTGS2 | CXCR4 | CD40LG |  |  |
| hsa-miR-193a-3p | PTX3 | CXCR4 | MMP9 |  |  |
| hsa-miR-193a-3p | TNC | CXCR4 | PLAU |  |  |
| hsa-miR-193a-3p | YWHAZ | CXCR4 | TLR4 |  |  |
| hsa-miR-200c-3p | ANGPTL2 | CXCR4 | AGER |  |  |
| hsa-miR-200c-3p | BRCA1 | CXCR4 | EDN1 |  |  |
| hsa-miR-200c-3p | CSF1 | CXCR4 | TEK |  |  |
| hsa-miR-200c-3p | CST3 | CXCR4 | INS |  |  |
| hsa-miR-200c-3p | CXCL16 | CXCR4 | ITGB2 |  |  |
| hsa-miR-200c-3p | EDN1 | CXCR4 | IL6 |  |  |
| hsa-miR-200c-3p | F3 | CXCR4 | PGF |  |  |
| hsa-miR-200c-3p | FSD1L | CXCR4 | ITGB3 |  |  |
| hsa-miR-200c-3p | GDF15 | CXCR4 | MYD88 |  |  |
| hsa-miR-200c-3p | IL18 | CXCR4 | BDNF |  |  |
| hsa-miR-200c-3p | IL1A | CXCR4 | ANGPT1 |  |  |
| hsa-miR-200c-3p | ITGB3 | CXCR4 | CD63 |  |  |
| hsa-miR-200c-3p | LCN2 | CXCR4 | IL10 |  |  |
| hsa-miR-200c-3p | LPXN | CXCR4 | PECAM1 |  |  |
| hsa-miR-200c-3p | MALAT1 | CXCR4 | TNF |  |  |
| hsa-miR-200c-3p | NOS3 | CXCR4 | HIF1A |  |  |
| hsa-miR-200c-3p | PAPPA | CXCR4 | CCL3 |  |  |
| hsa-miR-200c-3p | PECAM1 | CXCR4 | VEGFA |  |  |
| hsa-miR-200c-3p | PTGS2 | CXCR4 | CCL5 |  |  |
| hsa-miR-200c-3p | REN | CYBA | TNFRSF1A | |  |
| hsa-miR-200c-3p | S100A4 | CYBA | HMOX1 |  |  |
| hsa-miR-200c-3p | TGFB1 | CYBA | MMP2 |  |  |
| hsa-miR-200c-3p | TNC | CYBA | MPO |  |  |
| hsa-miR-200c-3p | TXNIP | CYBA | CCL2 |  |  |
| hsa-miR-29c-3p | AHR | CYBA | TLR2 |  |  |
| hsa-miR-29c-3p | CCL3 | CYBA | TLR4 |  |  |
| hsa-miR-29c-3p | CCL5 | CYBA | PTGS2 |  |  |
| hsa-miR-29c-3p | CD86 | CYBA | CYCS |  |  |
| hsa-miR-29c-3p | CNR1 | CYBA | INS |  |  |
| hsa-miR-29c-3p | CYCS | CYBA | EDN1 |  |  |
| hsa-miR-29c-3p | ENTPD1 | CYBA | ICAM1 |  |  |
| hsa-miR-29c-3p | F3 | CYBA | CTSS |  |  |
| hsa-miR-29c-3p | FAS | CYBA | IL6 |  |  |
| hsa-miR-29c-3p | GZMB | CYBA | ACE |  |  |
| hsa-miR-29c-3p | HIF1A | CYBA | IL1B |  |  |
| hsa-miR-29c-3p | HMOX1 | CYBA | AGTR1 |  |  |
| hsa-miR-29c-3p | HSPA4 | CYBA | REN |  |  |
| hsa-miR-29c-3p | HSPD1 | CYBA | AGT |  |  |
| hsa-miR-29c-3p | IGF1 | CYBA | TNF |  |  |
| hsa-miR-29c-3p | IGFBP1 | CYBA | S100A8 |  |  |
| hsa-miR-29c-3p | MYD88 | CYBA | S100A9 |  |  |
| hsa-miR-29c-3p | OLR1 | CYBA | NOS3 |  |  |
| hsa-miR-29c-3p | PARP1 | CYBA | MMP9 |  |  |
| hsa-miR-29c-3p | PCSK9 | CYBA | VCAM1 |  |  |
| hsa-miR-29c-3p | PTGS2 | CYCS | TNFRSF1A | |  |
| hsa-miR-29c-3p | PTX3 | CYCS | SIRT1 |  |  |
| hsa-miR-29c-3p | RPS27A | CYCS | HMOX1 |  |  |
| hsa-miR-29c-3p | SELPLG | CYCS | RIPK3 |  |  |
| hsa-miR-29c-3p | SERP1 | CYCS | GZMB |  |  |
| hsa-miR-29c-3p | TNFRSF11B | CYCS | MMP2 |  |  |
| hsa-miR-29c-3p | TNFRSF1A | CYCS | MPO |  |  |
| hsa-miR-29c-3p | TNNI3 | CYCS | CCL2 |  |  |
| hsa-miR-29c-3p | UBC | CYCS | IL2 |  |  |
| hsa-miR-301a-3p | ARSA | CYCS | EPO |  |  |
| hsa-miR-301a-3p | BMP6 | CYCS | RIPK1 |  |  |
| hsa-miR-301a-3p | CAD | CYCS | CYBA |  |  |
| hsa-miR-301a-3p | IL18 | CYCS | IL1B |  |  |
| hsa-miR-301a-3p | NFKB1 | CYCS | ICAM1 |  |  |
| hsa-miR-301a-3p | PPARG | CYCS | PPARGC1A | |  |
| hsa-miR-301a-3p | RPS27A | CYCS | IL18 |  |  |
| hsa-miR-361-5p | BSG | CYCS | PPARG |  |  |
| hsa-miR-361-5p | CYCS | CYCS | NOS3 |  |  |
| hsa-miR-361-5p | DPP4 | CYCS | IGF1 |  |  |
| hsa-miR-361-5p | ENTPD1 | CYCS | HSPA4 |  |  |
| hsa-miR-361-5p | FSD1L | CYCS | CXCL8 |  |  |
| hsa-miR-361-5p | HMGCR | CYCS | IL10 |  |  |
| hsa-miR-361-5p | MMP3 | CYCS | MLKL |  |  |
| hsa-miR-361-5p | PIK3C2A | CYCS | TLR4 |  |  |
| hsa-miR-361-5p | SERP1 | CYCS | BDNF |  |  |
| hsa-miR-361-5p | UBAP1 | CYCS | PARK2 |  |  |
| hsa-miR-361-5p | YWHAZ | CYCS | PTGS2 |  |  |
| hsa-miR-363-3p | ADM | CYCS | MMP9 |  |  |
| hsa-miR-363-3p | CAD | CYCS | IL6 |  |  |
| hsa-miR-363-3p | CYP2C19 | CYCS | HIF1A |  |  |
| hsa-miR-363-3p | FASLG | CYCS | VEGFA |  |  |
| hsa-miR-373-5p | CYCS | CYCS | INS |  |  |
| hsa-miR-373-5p | ITGA2 | CYCS | FAS |  |  |
| hsa-miR-373-5p | MON2 | CYCS | PARP1 |  |  |
| hsa-miR-373-5p | OLR1 | CYCS | FASLG |  |  |
| hsa-miR-373-5p | PIK3C2A | CYCS | TNF |  |  |
| hsa-miR-373-5p | PTX3 | CYCS | HSPD1 |  |  |
| hsa-miR-374a-5p | ADAMTS4 | CYCS | MB |  |  |
| hsa-miR-374a-5p | ADM | CYP2C19 | PON1 |  |  |
| hsa-miR-374a-5p | AHR | CYP2C19 | AHR |  |  |
| hsa-miR-374a-5p | BGLAP | CYP2C19 | ACE |  |  |
| hsa-miR-374a-5p | BMP6 | CYP2C19 | PLB1 |  |  |
| hsa-miR-374a-5p | C1S | CYP2C19 | PTGS2 |  |  |
| hsa-miR-374a-5p | CCL2 | CYP2C19 | ENTPD1 |  |  |
| hsa-miR-374a-5p | CCN2 | CYP2C19 | PLA2G2A |  |  |
| hsa-miR-374a-5p | CHI3L1 | DPP4 | MMP2 |  |  |
| hsa-miR-374a-5p | CNR1 | DPP4 | RETN |  |  |
| hsa-miR-374a-5p | CST3 | DPP4 | SERPINE1 |  |  |
| hsa-miR-374a-5p | CX3CL1 | DPP4 | CCL2 |  |  |
| hsa-miR-374a-5p | EDN1 | DPP4 | IL2 |  |  |
| hsa-miR-374a-5p | ENTPD1 | DPP4 | IFNG |  |  |
| hsa-miR-374a-5p | FAS | DPP4 | APOB |  |  |
| hsa-miR-374a-5p | FSD1 | DPP4 | LGALS3 |  |  |
| hsa-miR-374a-5p | FST | DPP4 | CRP |  |  |
| hsa-miR-374a-5p | HIF1A | DPP4 | TYR |  |  |
| hsa-miR-374a-5p | HLA-DRB1 | DPP4 | IL1B |  |  |
| hsa-miR-374a-5p | HMOX1 | DPP4 | ICAM1 |  |  |
| hsa-miR-374a-5p | ICAM1 | DPP4 | KNG1 |  |  |
| hsa-miR-374a-5p | IL18 | DPP4 | ITIH4 |  |  |
| hsa-miR-374a-5p | INTS2 | DPP4 | REN |  |  |
| hsa-miR-374a-5p | LEP | DPP4 | PPARG |  |  |
| hsa-miR-374a-5p | MB | DPP4 | ACE |  |  |
| hsa-miR-374a-5p | MLKL | DPP4 | VCAM1 |  |  |
| hsa-miR-374a-5p | MMP9 | DPP4 | NOS3 |  |  |
| hsa-miR-374a-5p | MRAS | DPP4 | CCL11 |  |  |
| hsa-miR-374a-5p | NAMPT | DPP4 | IGF1 |  |  |
| hsa-miR-374a-5p | PARP1 | DPP4 | PLG |  |  |
| hsa-miR-374a-5p | PLAT | DPP4 | LEP |  |  |
| hsa-miR-374a-5p | PLAU | DPP4 | CD28 |  |  |
| hsa-miR-374a-5p | PTGS2 | DPP4 | FGF21 |  |  |
| hsa-miR-374a-5p | PTX3 | DPP4 | IL10 |  |  |
| hsa-miR-374a-5p | S100A4 | DPP4 | MMP9 |  |  |
| hsa-miR-374a-5p | SELENOS | DPP4 | VEGFA |  |  |
| hsa-miR-374a-5p | SERPINA1 | DPP4 | PECAM1 |  |  |
| hsa-miR-374a-5p | SERPINA5 | DPP4 | ENTPD1 |  |  |
| hsa-miR-374a-5p | THBS4 | DPP4 | NPPB |  |  |
| hsa-miR-374a-5p | TNFRSF11A | DPP4 | TNF |  |  |
| hsa-miR-374a-5p | TNNT1 | DPP4 | VIP |  |  |
| hsa-miR-374a-5p | TXNIP | DPP4 | IL6 |  |  |
| hsa-miR-374a-5p | VEGFA | DPP4 | CCL5 |  |  |
| hsa-miR-378a-5p | CST3 | DPP4 | INS |  |  |
| hsa-miR-378a-5p | CYCS | DPP4 | CXCR4 |  |  |
| hsa-miR-378a-5p | HSPA4 | EBI3 | IL10 |  |  |
| hsa-miR-378a-5p | PARP1 | EBI3 | IL12A |  |  |
| hsa-miR-378a-5p | PIK3C2A | EDN1 | SIRT1 |  |  |
| hsa-miR-378a-5p | PPARGC1A | EDN1 | HMOX1 |  |  |
| hsa-miR-378a-5p | PYGB | EDN1 | TIMP1 |  |  |
| hsa-miR-378a-5p | TNFRSF1B | EDN1 | MMP2 |  |  |
| hsa-miR-378a-5p | VEGFA | EDN1 | PLAT |  |  |
| hsa-miR-340-3p | CHGA | EDN1 | RETN |  |  |
| hsa-miR-340-3p | MON2 | EDN1 | TGFB1 |  |  |
| hsa-miR-340-3p | PON1 | EDN1 | HGF |  |  |
| hsa-miR-340-3p | VEGFA | EDN1 | SERPINE1 |  |  |
| hsa-miR-323a-3p | CCL2 | EDN1 | MPO |  |  |
| hsa-miR-323a-3p | FST | EDN1 | CCL2 |  |  |
| hsa-miR-323a-3p | SMAD3 | EDN1 | IFNG |  |  |
| hsa-miR-323a-3p | TNFRSF11B | EDN1 | EPO |  |  |
| hsa-miR-323a-3p | TXNIP | EDN1 | CRP |  |  |
| hsa-miR-338-3p | AHR | EDN1 | TLR2 |  |  |
| hsa-miR-338-3p | CAD | EDN1 | VWF |  |  |
| hsa-miR-338-3p | CSF1 | EDN1 | CYBA |  |  |
| hsa-miR-338-3p | FSD1L | EDN1 | TYR |  |  |
| hsa-miR-338-3p | HMGCR | EDN1 | IL1A |  |  |
| hsa-miR-338-3p | ITGB3 | EDN1 | IL1B |  |  |
| hsa-miR-338-3p | MMP9 | EDN1 | SELP |  |  |
| hsa-miR-338-3p | MORF4L2 | EDN1 | ICAM1 |  |  |
| hsa-miR-338-3p | RIPK1 | EDN1 | KNG1 |  |  |
| hsa-miR-338-3p | VEGFA | EDN1 | REN |  |  |
| hsa-miR-133b | CCN2 | EDN1 | IL18 |  |  |
| hsa-miR-133b | CXCR4 | EDN1 | PPARG |  |  |
| hsa-miR-133b | FAS | EDN1 | ACE |  |  |
| hsa-miR-133b | MMP9 | EDN1 | VCAM1 |  |  |
| hsa-miR-133b | PARP1 | EDN1 | PTX3 |  |  |
| hsa-miR-133b | SIRT1 | EDN1 | NOS3 |  |  |
| hsa-miR-196b-5p | AHR | EDN1 | MMP3 |  |  |
| hsa-miR-196b-5p | HIF1A | EDN1 | CCL11 |  |  |
| hsa-miR-196b-5p | MYDGF | EDN1 | IGF1 |  |  |
| hsa-miR-196b-5p | PLAT | EDN1 | ADRB2 |  |  |
| hsa-miR-196b-5p | SERPINE1 | EDN1 | CXCL8 |  |  |
| hsa-miR-196b-5p | TNFRSF11B | EDN1 | F2 |  |  |
| hsa-miR-423-3p | CAD | EDN1 | PLG |  |  |
| hsa-miR-423-3p | CCN2 | EDN1 | OLR1 |  |  |
| hsa-miR-423-3p | F2 | EDN1 | LEP |  |  |
| hsa-miR-423-3p | HSPA4 | EDN1 | ANGPT2 |  |  |
| hsa-miR-423-3p | HSPD1 | EDN1 | MMP1 |  |  |
| hsa-miR-423-3p | MIF | EDN1 | SELE |  |  |
| hsa-miR-423-3p | SIRT1 | EDN1 | SMAD3 |  |  |
| hsa-miR-18b-5p | AHR | EDN1 | F3 |  |  |
| hsa-miR-18b-5p | F3 | EDN1 | TNNI3 |  |  |
| hsa-miR-18b-5p | HIF1A | EDN1 | AGT |  |  |
| hsa-miR-18b-5p | IGF1 | EDN1 | VIP |  |  |
| hsa-miR-18b-5p | MYDGF | EDN1 | PTGS2 |  |  |
| hsa-miR-18b-5p | TXNIP | EDN1 | SERPINC1 | |  |
| hsa-miR-18b-5p | UBAP1 | EDN1 | CTGF |  |  |
| hsa-miR-448 | FGF21 | EDN1 | MMP9 |  |  |
| hsa-miR-448 | SELENOS | EDN1 | PLAU |  |  |
| hsa-miR-429 | AHR | EDN1 | TLR4 |  |  |
| hsa-miR-429 | ARSA | EDN1 | NPPB |  |  |
| hsa-miR-429 | CCN2 | EDN1 | THBD |  |  |
| hsa-miR-429 | CXCL8 | EDN1 | CCL5 |  |  |
| hsa-miR-429 | CXCR4 | EDN1 | TEK |  |  |
| hsa-miR-429 | DPP4 | EDN1 | ANGPT1 |  |  |
| hsa-miR-429 | FSD1L | EDN1 | IL10 |  |  |
| hsa-miR-429 | GDF15 | EDN1 | PGF |  |  |
| hsa-miR-429 | HDAC9 | EDN1 | PECAM1 |  |  |
| hsa-miR-429 | HLCS | EDN1 | AGTR1 |  |  |
| hsa-miR-429 | HMOX1 | EDN1 | AVP |  |  |
| hsa-miR-429 | IL18 | EDN1 | IL6 |  |  |
| hsa-miR-429 | IL1B | EDN1 | TNF |  |  |
| hsa-miR-429 | IL6 | EDN1 | ADM |  |  |
| hsa-miR-429 | ITGA2 | EDN1 | INS |  |  |
| hsa-miR-429 | LCN2 | EDN1 | VEGFA |  |  |
| hsa-miR-429 | MALAT1 | EDN1 | CXCR4 |  |  |
| hsa-miR-429 | MMP1 | EDN1 | HIF1A |  |  |
| hsa-miR-429 | MMP28 | ENHO | INS |  |  |
| hsa-miR-429 | PLAU | ENSP00000485396 | SIRT1 |  |  |
| hsa-miR-429 | RAB3IL1 | ENSP00000485396 | HDAC9 |  |  |
| hsa-miR-429 | S100A9 | ENTPD1 | GZMB |  |  |
| hsa-miR-429 | SIRT1 | ENTPD1 | IL2 |  |  |
| hsa-miR-429 | TNFRSF11A | ENTPD1 | IFNG |  |  |
| hsa-miR-429 | VEGFA | ENTPD1 | AHR |  |  |
| hsa-miR-431-5p | APOA1 | ENTPD1 | CRP |  |  |
| hsa-miR-431-5p | IL7 | ENTPD1 | TLR2 |  |  |
| hsa-miR-451a | IL6 | ENTPD1 | VWF |  |  |
| hsa-miR-451a | MIF | ENTPD1 | IL1B |  |  |
| hsa-miR-451a | MMP2 | ENTPD1 | SELP |  |  |
| hsa-miR-451a | MMP9 | ENTPD1 | ICAM1 |  |  |
| hsa-miR-486-5p | HLCS | ENTPD1 | CD28 |  |  |
| hsa-miR-486-5p | SERPINE1 | ENTPD1 | CD86 |  |  |
| hsa-miR-486-5p | SIRT1 | ENTPD1 | F3 |  |  |
| hsa-miR-486-5p | TAF1 | ENTPD1 | IL17A |  |  |
| hsa-miR-146b-5p | CCL3 | ENTPD1 | DPP4 |  |  |
| hsa-miR-146b-5p | IL6 | ENTPD1 | FASLG |  |  |
| hsa-miR-146b-5p | MALAT1 | ENTPD1 | CD40LG |  |  |
| hsa-miR-146b-5p | NFKB1 | ENTPD1 | TLR4 |  |  |
| hsa-miR-146b-5p | PAPPA | ENTPD1 | PECAM1 |  |  |
| hsa-miR-146b-5p | PTGS2 | ENTPD1 | HIF1A |  |  |
| hsa-miR-146b-5p | TLR4 | ENTPD1 | IL2RA |  |  |
| hsa-miR-146b-5p | TXNIP | ENTPD1 | CYP2C19 |  |  |
| hsa-miR-493-5p | CCN2 | ENTPD1 | IL6 |  |  |
| hsa-miR-493-5p | CXCL8 | ENTPD1 | TNF |  |  |
| hsa-miR-493-5p | OLR1 | ENTPD1 | IL10 |  |  |
| hsa-miR-525-5p | HMOX1 | ENTPD1 | THBD |  |  |
| hsa-miR-516b-5p | ADM | EPO | HMOX1 |  |  |
| hsa-miR-516b-5p | BSG | EPO | IL2RB |  |  |
| hsa-miR-516b-5p | PLAT | EPO | HGF |  |  |
| hsa-miR-513a-5p | FAS | EPO | CCL2 |  |  |
| hsa-miR-513a-5p | OLR1 | EPO | NFKB1 |  |  |
| hsa-miR-513a-5p | SERP1 | EPO | IL2 |  |  |
| hsa-miR-532-5p | COL6A2 | EPO | IFNG |  |  |
| hsa-miR-532-5p | FAS | EPO | IL5 |  |  |
| hsa-miR-532-5p | FST | EPO | BMP6 |  |  |
| hsa-miR-532-5p | HSPD1 | EPO | CYCS |  |  |
| hsa-miR-532-5p | MYDGF | EPO | ICAM1 |  |  |
| hsa-miR-532-5p | NFKB1 | EPO | SERPINA1 | |  |
| hsa-miR-532-5p | PIK3C2A | EPO | SERPINC1 | |  |
| hsa-miR-532-5p | S100A4 | EPO | VCAM1 |  |  |
| hsa-miR-532-5p | UBC | EPO | F3 |  |  |
| hsa-miR-532-5p | VEGFA | EPO | MB |  |  |
| hsa-miR-299-5p | HIF1A | EPO | ADM |  |  |
| hsa-miR-299-5p | SERPINE1 | EPO | IFNA1 |  |  |
| hsa-miR-561-3p | PARP1 | EPO | ANGPT2 |  |  |
| hsa-miR-561-3p | PTGS2 | EPO | TEK |  |  |
| hsa-miR-561-3p | TXNIP | EPO | CSF1 |  |  |
| hsa-miR-583 | HIF1A | EPO | VWF |  |  |
| hsa-miR-583 | SERPINE1 | EPO | PGF |  |  |
| hsa-miR-548c-3p | ADM | EPO | MMP9 |  |  |
| hsa-miR-548c-3p | MON2 | EPO | CXCL8 |  |  |
| hsa-miR-548c-3p | MRAS | EPO | CXCR4 |  |  |
| hsa-miR-548c-3p | PON1 | EPO | PECAM1 |  |  |
| hsa-miR-626 | CCN2 | EPO | IL7 |  |  |
| hsa-miR-626 | MMP2 | EPO | ANGPT1 |  |  |
| hsa-miR-626 | SERPINC1 | EPO | PLG |  |  |
| hsa-miR-626 | YWHAZ | EPO | GDF15 |  |  |
| hsa-miR-628-3p | PF4 | EPO | ITGA2B |  |  |
| hsa-miR-628-3p | TXNIP | EPO | F2 |  |  |
| hsa-miR-641 | ENTPD1 | EPO | IL1B |  |  |
| hsa-miR-641 | ICAM1 | EPO | EDN1 |  |  |
| hsa-miR-641 | MON2 | EPO | IL10 |  |  |
| hsa-miR-641 | NRDC | EPO | NOS3 |  |  |
| hsa-miR-648 | EDN1 | EPO | BDNF |  |  |
| hsa-miR-652-3p | MORF4L2 | EPO | TNF |  |  |
| hsa-miR-652-3p | MRAS | EPO | IL2RA |  |  |
| hsa-miR-660-5p | FSD1 | EPO | IGF1 |  |  |
| hsa-miR-671-5p | ANGPT1 | EPO | REN |  |  |
| hsa-miR-671-5p | C1S | EPO | ACE |  |  |
| hsa-miR-671-5p | CCL2 | EPO | INS |  |  |
| hsa-miR-671-5p | CD63 | EPO | CRP |  |  |
| hsa-miR-671-5p | CHI3L1 | EPO | IL6 |  |  |
| hsa-miR-671-5p | CSF1 | EPO | VEGFA |  |  |
| hsa-miR-671-5p | CXCL8 | EPO | LEP |  |  |
| hsa-miR-671-5p | ENTPD1 | EPO | HIF1A |  |  |
| hsa-miR-671-5p | FAS | F10 | CPB2 |  |  |
| hsa-miR-671-5p | ICAM1 | F10 | PLAT |  |  |
| hsa-miR-671-5p | IL10 | F10 | SERPINE1 |  |  |
| hsa-miR-671-5p | IL6 | F10 | VWF |  |  |
| hsa-miR-671-5p | LCN2 | F10 | ITGA2B |  |  |
| hsa-miR-671-5p | MMP2 | F10 | SELP |  |  |
| hsa-miR-671-5p | NAMPT | F10 | KNG1 |  |  |
| hsa-miR-671-5p | OLR1 | F10 | HABP2 |  |  |
| hsa-miR-671-5p | PAPPA | F10 | PF4 |  |  |
| hsa-miR-671-5p | PLAU | F10 | FGA |  |  |
| hsa-miR-671-5p | PTX3 | F10 | F2 |  |  |
| hsa-miR-671-5p | S100A4 | F10 | PLG |  |  |
| hsa-miR-671-5p | S100A8 | F10 | SERPINF2 |  |  |
| hsa-miR-671-5p | S100A9 | F10 | GP1BA |  |  |
| hsa-miR-671-5p | SCUBE1 | F10 | SERPINA5 | |  |
| hsa-miR-671-5p | SERPINA1 | F10 | F3 |  |  |
| hsa-miR-671-5p | SIRT1 | F10 | SERPINC1 | |  |
| hsa-miR-671-5p | SMAD3 | F10 | IL6 |  |  |
| hsa-miR-671-5p | THBD | F10 | TNF |  |  |
| hsa-miR-671-5p | TIMP1 | F10 | SERPINA1 | |  |
| hsa-miR-671-5p | TXNIP | F10 | PLA2G2A |  |  |
| hsa-miR-767-5p | CAD | F10 | THBD |  |  |
| hsa-miR-767-5p | CYP2C19 | F10 | ITGB2 |  |  |
| hsa-miR-767-5p | MORF4L2 | F10 | F7 |  |  |
| hsa-miR-766-3p | AHR | F2 | CPB2 |  |  |
| hsa-miR-766-3p | CAD | F2 | PLAT |  |  |
| hsa-miR-766-3p | CHGA | F2 | TGFB1 |  |  |
| hsa-miR-766-3p | EBI3 | F2 | SERPINE1 |  |  |
| hsa-miR-766-3p | ENTPD1 | F2 | MPO |  |  |
| hsa-miR-766-3p | HSPD1 | F2 | CCL2 |  |  |
| hsa-miR-766-3p | ICAM1 | F2 | IFNG |  |  |
| hsa-miR-766-3p | MYDGF | F2 | HRG |  |  |
| hsa-miR-766-3p | PARP1 | F2 | APOB |  |  |
| hsa-miR-766-3p | PON1 | F2 | MMP8 |  |  |
| hsa-miR-766-3p | SERPING1 | F2 | APOA1 |  |  |
| hsa-miR-766-3p | TNC | F2 | EPO |  |  |
| hsa-miR-766-3p | TNFRSF1A | F2 | CRP |  |  |
| hsa-miR-766-3p | TNNT1 | F2 | VWF |  |  |
| hsa-miR-766-3p | UBC | F2 | ITGA2B |  |  |
| hsa-miR-765 | CSF1 | F2 | TYR |  |  |
| hsa-miR-765 | GP5 | F2 | IL1B |  |  |
| hsa-miR-765 | PAPPA | F2 | SELP |  |  |
| hsa-miR-765 | PCSK9 | F2 | CP |  |  |
| hsa-miR-765 | PLAT | F2 | KNG1 |  |  |
| hsa-miR-765 | SERPINE1 | F2 | PIK3C2A |  |  |
| hsa-miR-765 | TNFRSF11B | F2 | ITIH4 |  |  |
| hsa-miR-765 | YWHAZ | F2 | REN |  |  |
| hsa-miR-675-5p | ARSA | F2 | IGFBP1 |  |  |
| hsa-miR-675-5p | NFKB1 | F2 | HABP2 |  |  |
| hsa-miR-675-5p | TGFB1 | F2 | SAA4 |  |  |
| hsa-miR-675-5p | TYR | F2 | SERPING1 | |  |
| hsa-let-7f-2-3p | CCN2 | F2 | ACE |  |  |
| hsa-let-7f-2-3p | IL7 | F2 | VCAM1 |  |  |
| hsa-let-7f-2-3p | PTX3 | F2 | PPBP |  |  |
| hsa-miR-28-3p | INTS2 | F2 | PF4 |  |  |
| hsa-miR-16-2-3p | CCN2 | F2 | NOS3 |  |  |
| hsa-miR-16-2-3p | CD86 | F2 | CCL11 |  |  |
| hsa-miR-16-2-3p | CXCR4 | F2 | IGF1 |  |  |
| hsa-miR-16-2-3p | HIF1A | F2 | ADRB2 |  |  |
| hsa-miR-16-2-3p | SMAD3 | F2 | FGA |  |  |
| hsa-miR-30c-2-3p | ADAMTS4 | F2 | CXCL8 |  |  |
| hsa-miR-30c-2-3p | AHR | F2 | NPPB |  |  |
| hsa-miR-30c-2-3p | CCN2 | F2 | TLR4 |  |  |
| hsa-miR-30c-2-3p | CXCL16 | F2 | IL10 |  |  |
| hsa-miR-30c-2-3p | FST | F2 | CD40LG |  |  |
| hsa-miR-30c-2-3p | GP5 | F2 | BGLAP |  |  |
| hsa-miR-30c-2-3p | HMOX1 | F2 | PLG |  |  |
| hsa-miR-30c-2-3p | IGF1 | F2 | PECAM1 |  |  |
| hsa-miR-30c-2-3p | ITGB3 | F2 | F7 |  |  |
| hsa-miR-30c-2-3p | TNC | F2 | MB |  |  |
| hsa-miR-30c-2-3p | TXNIP | F2 | SELE |  |  |
| hsa-miR-181c-3p | IL1B | F2 | ITGB3 |  |  |
| hsa-miR-181c-3p | SERPINC1 | F2 | MBL2 |  |  |
| hsa-miR-181c-3p | TNC | F2 | MMP9 |  |  |
| hsa-miR-181c-3p | TXNIP | F2 | INS |  |  |
| hsa-miR-214-5p | IGF1 | F2 | VEGFA |  |  |
| hsa-miR-214-5p | RIPK1 | F2 | TNF |  |  |
| hsa-miR-221-5p | ADM | F2 | AVP |  |  |
| hsa-miR-221-5p | BSG | F2 | IL6 |  |  |
| hsa-miR-221-5p | FERMT3 | F2 | SERPINA1 | |  |
| hsa-miR-221-5p | IL1A | F2 | GP5 |  |  |
| hsa-miR-221-5p | ITGB3 | F2 | AGTR1 |  |  |
| hsa-miR-221-5p | MMP2 | F2 | SERPINF2 |  |  |
| hsa-miR-221-5p | MORF4L2 | F2 | CXCR4 |  |  |
| hsa-miR-221-5p | PARP1 | F2 | PLAU |  |  |
| hsa-miR-221-5p | PECAM1 | F2 | F10 |  |  |
| hsa-miR-221-5p | PTX3 | F2 | EDN1 |  |  |
| hsa-miR-221-5p | SELENOS | F2 | AGT |  |  |
| hsa-miR-221-5p | UBAP1 | F2 | F3 |  |  |
| hsa-miR-221-5p | VWF | F2 | GP1BA |  |  |
| hsa-miR-221-5p | YWHAZ | F2 | SERPINA5 | |  |
| hsa-miR-223-5p | FSD1L | F2 | THBD |  |  |
| hsa-miR-223-5p | IGF1 | F2 | SERPINC1 | |  |
| hsa-let-7g-3p | CAD | F3 | CPB2 |  |  |
| hsa-let-7g-3p | CCL2 | F3 | TIMP1 |  |  |
| hsa-let-7g-3p | CCL5 | F3 | MMP2 |  |  |
| hsa-let-7g-3p | CCL5 | F3 | PLAT |  |  |
| hsa-let-7g-3p | FST | F3 | SERPINE1 |  |  |
| hsa-let-7g-3p | OLR1 | F3 | MPO |  |  |
| hsa-let-7g-3p | TXNIP | F3 | CCL2 |  |  |
| hsa-miR-15b-3p | MORF4L2 | F3 | IL2 |  |  |
| hsa-miR-15b-3p | SULT1E1 | F3 | SELPLG |  |  |
| hsa-miR-27b-5p | LPXN | F3 | IFNG |  |  |
| hsa-miR-27b-5p | PAPPA | F3 | HRG |  |  |
| hsa-miR-27b-5p | PARP1 | F3 | APOB |  |  |
| hsa-miR-27b-5p | RIPK1 | F3 | APOA1 |  |  |
| hsa-miR-27b-5p | SEMA4D | F3 | EPO |  |  |
| hsa-miR-27b-5p | TXNIP | F3 | CRP |  |  |
| hsa-miR-27b-5p | YWHAZ | F3 | TLR2 |  |  |
| hsa-miR-138-2-3p | BDNF | F3 | VWF |  |  |
| hsa-miR-140-3p | GDF15 | F3 | ITGA2B |  |  |
| hsa-miR-140-3p | SERPINE1 | F3 | IL1A |  |  |
| hsa-miR-140-3p | YWHAZ | F3 | IL1B |  |  |
| hsa-miR-129-2-3p | ADM | F3 | SELP |  |  |
| hsa-miR-129-2-3p | ADRB2 | F3 | ICAM1 |  |  |
| hsa-miR-129-2-3p | AGT | F3 | KNG1 |  |  |
| hsa-miR-129-2-3p | AGTR1 | F3 | PIK3C2A |  |  |
| hsa-miR-129-2-3p | AHR | F3 | REN |  |  |
| hsa-miR-129-2-3p | ANGPT1 | F3 | SERPING1 | |  |
| hsa-miR-129-2-3p | ANGPTL2 | F3 | ACE |  |  |
| hsa-miR-129-2-3p | BDNF | F3 | VCAM1 |  |  |
| hsa-miR-129-2-3p | BRCA1 | F3 | PTX3 |  |  |
| hsa-miR-129-2-3p | C1S | F3 | PPBP |  |  |
| hsa-miR-129-2-3p | CCL2 | F3 | PF4 |  |  |
| hsa-miR-129-2-3p | CCL5 | F3 | NOS3 |  |  |
| hsa-miR-129-2-3p | CCN2 | F3 | FGA |  |  |
| hsa-miR-129-2-3p | CD14 | F3 | CXCL8 |  |  |
| hsa-miR-129-2-3p | CD63 | F3 | F2 |  |  |
| hsa-miR-129-2-3p | CHI3L1 | F3 | PLG |  |  |
| hsa-miR-129-2-3p | COG2 | F3 | OLR1 |  |  |
| hsa-miR-129-2-3p | COL6A2 | F3 | ANGPT2 |  |  |
| hsa-miR-129-2-3p | CP | F3 | SERPINF2 |  |  |
| hsa-miR-129-2-3p | CSF1 | F3 | GP1BA |  |  |
| hsa-miR-129-2-3p | CST3 | F3 | SELE |  |  |
| hsa-miR-129-2-3p | CTSS | F3 | SERPINA5 | |  |
| hsa-miR-129-2-3p | CX3CL1 | F3 | PGF |  |  |
| hsa-miR-129-2-3p | CXCL16 | F3 | MB |  |  |
| hsa-miR-129-2-3p | CXCL8 | F3 | CD63 |  |  |
| hsa-miR-129-2-3p | CYCS | F3 | CCL5 |  |  |
| hsa-miR-129-2-3p | DPP4 | F3 | SERPINA1 | |  |
| hsa-miR-129-2-3p | EBI3 | F3 | ENTPD1 |  |  |
| hsa-miR-129-2-3p | EDN1 | F3 | PTGS2 |  |  |
| hsa-miR-129-2-3p | F3 | F3 | ITGB3 |  |  |
| hsa-miR-129-2-3p | FAS | F3 | TLR4 |  |  |
| hsa-miR-129-2-3p | FST | F3 | PECAM1 |  |  |
| hsa-miR-129-2-3p | GDF15 | F3 | IL10 |  |  |
| hsa-miR-129-2-3p | HLCS | F3 | INS |  |  |
| hsa-miR-129-2-3p | ICAM1 | F3 | MMP9 |  |  |
| hsa-miR-129-2-3p | IGFBP1 | F3 | PLAU |  |  |
| hsa-miR-129-2-3p | IL12A | F3 | EDN1 |  |  |
| hsa-miR-129-2-3p | IL17D | F3 | CD40LG |  |  |
| hsa-miR-129-2-3p | IL1A | F3 | VEGFA |  |  |
| hsa-miR-129-2-3p | IL1B | F3 | TNF |  |  |
| hsa-miR-129-2-3p | IL1RN | F3 | IL6 |  |  |
| hsa-miR-129-2-3p | IL32 | F3 | THBD |  |  |
| hsa-miR-129-2-3p | IL7 | F3 | SERPINC1 | |  |
| hsa-miR-129-2-3p | ITGA2 | F3 | F10 |  |  |
| hsa-miR-129-2-3p | ITGB3 | F3 | F7 |  |  |
| hsa-miR-129-2-3p | LGALS3 | F7 | CPB2 |  |  |
| hsa-miR-129-2-3p | LPXN | F7 | PLAT |  |  |
| hsa-miR-129-2-3p | MB | F7 | SERPINE1 |  |  |
| hsa-miR-129-2-3p | MLKL | F7 | APOB |  |  |
| hsa-miR-129-2-3p | MON2 | F7 | APOA1 |  |  |
| hsa-miR-129-2-3p | MRAS | F7 | CRP |  |  |
| hsa-miR-129-2-3p | MYD88 | F7 | VWF |  |  |
| hsa-miR-129-2-3p | MYDGF | F7 | ITGA2B |  |  |
| hsa-miR-129-2-3p | NAMPT | F7 | SELP |  |  |
| hsa-miR-129-2-3p | NPPB | F7 | KNG1 |  |  |
| hsa-miR-129-2-3p | PAPPA | F7 | HABP2 |  |  |
| hsa-miR-129-2-3p | PECAM1 | F7 | ACE |  |  |
| hsa-miR-129-2-3p | PF4 | F7 | PPBP |  |  |
| hsa-miR-129-2-3p | PLAT | F7 | PF4 |  |  |
| hsa-miR-129-2-3p | PPARG | F7 | FGA |  |  |
| hsa-miR-129-2-3p | PTGS2 | F7 | F2 |  |  |
| hsa-miR-129-2-3p | PTX3 | F7 | PLG |  |  |
| hsa-miR-129-2-3p | RAB3IL1 | F7 | SERPINF2 |  |  |
| hsa-miR-129-2-3p | RIPK1 | F7 | F3 |  |  |
| hsa-miR-129-2-3p | S100A4 | F7 | AGT |  |  |
| hsa-miR-129-2-3p | SELE | F7 | SERPINC1 | |  |
| hsa-miR-129-2-3p | SERPINE1 | F7 | MBL2 |  |  |
| hsa-miR-129-2-3p | SERPING1 | F7 | F10 |  |  |
| hsa-miR-129-2-3p | TEK | F7 | TNF |  |  |
| hsa-miR-129-2-3p | THBD | F7 | IL6 |  |  |
| hsa-miR-129-2-3p | TLR2 | F7 | INS |  |  |
| hsa-miR-129-2-3p | TLR4 | F7 | SERPINA1 | |  |
| hsa-miR-129-2-3p | TNC | F7 | THBD |  |  |
| hsa-miR-129-2-3p | TNFRSF11A | FABP3 | PYGB |  |  |
| hsa-miR-129-2-3p | TNFRSF11B | FABP3 | APOA1 |  |  |
| hsa-miR-129-2-3p | TNFRSF1A | FABP3 | TNNT2 |  |  |
| hsa-miR-129-2-3p | TNFRSF1B | FABP3 | GDF15 |  |  |
| hsa-miR-129-2-3p | TNFSF4 | FABP3 | CRP |  |  |
| hsa-miR-129-2-3p | TNNI1 | FABP3 | PPARGC1A | |  |
| hsa-miR-129-2-3p | TNNT1 | FABP3 | PPARG |  |  |
| hsa-miR-129-2-3p | TREML1 | FABP3 | ITGA2 |  |  |
| hsa-miR-129-2-3p | TXNIP | FABP3 | TNNI3 |  |  |
| hsa-miR-129-2-3p | UBAP1 | FABP3 | LCN2 |  |  |
| hsa-miR-129-2-3p | VWF | FABP3 | INS |  |  |
| hsa-miR-129-2-3p | YWHAZ | FABP3 | AVP |  |  |
| hsa-miR-195-3p | CCN2 | FABP3 | NPPB |  |  |
| hsa-miR-200c-5p | HIF1A | FABP3 | TNNT1 |  |  |
| hsa-miR-200c-5p | TNC | FABP3 | MB |  |  |
| hsa-miR-200c-5p | UBC | FAS | TNFRSF1A | |  |
| hsa-miR-106b-3p | HIF1A | FAS | IL2RB |  |  |
| hsa-miR-106b-3p | PLAT | FAS | RIPK3 |  |  |
| hsa-miR-106b-3p | SERPINE1 | FAS | GZMB |  |  |
| hsa-miR-330-5p | ITGA2 | FAS | CCL2 |  |  |
| hsa-miR-330-5p | PARP1 | FAS | IL2 |  |  |
| hsa-miR-330-5p | PTGS2 | FAS | IFNG |  |  |
| hsa-miR-330-5p | TNC | FAS | IL5 |  |  |
| hsa-miR-330-5p | TYR | FAS | LGALS3 |  |  |
| hsa-miR-337-5p | HIF1A | FAS | RIPK1 |  |  |
| hsa-miR-151a-5p | SELENOS | FAS | IL1B |  |  |
| hsa-miR-151a-5p | TXNIP | FAS | IL7 |  |  |
| hsa-miR-151a-5p | VWF | FAS | ICAM1 |  |  |
| hsa-miR-486-3p | CAD | FAS | FCGR2A |  |  |
| hsa-miR-486-3p | CST3 | FAS | IFNA1 |  |  |
| hsa-miR-486-3p | ICAM1 | FAS | IL18 |  |  |
| hsa-miR-486-3p | ITGB3 | FAS | HSPA4 |  |  |
| hsa-miR-486-3p | TXNIP | FAS | CXCL8 |  |  |
| hsa-miR-491-3p | NAMPT | FAS | CYCS |  |  |
| hsa-miR-502-3p | CCN2 | FAS | MLKL |  |  |
| hsa-miR-502-3p | HIF1A | FAS | CD28 |  |  |
| hsa-miR-502-3p | PTX3 | FAS | CD86 |  |  |
| hsa-miR-455-3p | AHR | FAS | IL17A |  |  |
| hsa-miR-455-3p | CAD | FAS | MYD88 |  |  |
| hsa-miR-455-3p | CXCL16 | FAS | VEGFA |  |  |
| hsa-miR-455-3p | CYP2C19 | FAS | IL2RA |  |  |
| hsa-miR-455-3p | IGF1 | FAS | TLR4 |  |  |
| hsa-miR-455-3p | PTX3 | FAS | TNFRSF1B | |  |
| hsa-miR-455-3p | SERPINA1 | FAS | CXCR4 |  |  |
| hsa-miR-455-3p | SIRT1 | FAS | IL10 |  |  |
| hsa-miR-455-3p | TNC | FAS | IL6 |  |  |
| hsa-miR-455-3p | TNNI1 | FAS | CD40LG |  |  |
| hsa-miR-455-3p | TXNIP | FAS | TNF |  |  |
| hsa-miR-455-3p | VCAM1 | FAS | FASLG |  |  |
| hsa-miR-455-3p | YWHAZ | FASLG | TNFRSF1A | |  |
| hsa-miR-548d-5p | BGLAP | FASLG | IL2RB |  |  |
| hsa-miR-548d-5p | CCN2 | FASLG | RIPK3 |  |  |
| hsa-miR-548d-5p | PPARG | FASLG | GZMB |  |  |
| hsa-miR-548d-5p | SIRT1 | FASLG | MMP2 |  |  |
| hsa-miR-548d-5p | TXNIP | FASLG | HGF |  |  |
| hsa-miR-548d-5p | YWHAZ | FASLG | CCL2 |  |  |
| hsa-miR-411-3p | CCN2 | FASLG | NFKB1 |  |  |
| hsa-miR-411-3p | FSD1L | FASLG | IL2 |  |  |
| hsa-miR-411-3p | FST | FASLG | IFNG |  |  |
| hsa-miR-411-3p | SERP1 | FASLG | IL5 |  |  |
| hsa-miR-671-3p | ADAMTS7 | FASLG | RIPK1 |  |  |
| hsa-miR-300 | SEMA4D | FASLG | TLR2 |  |  |
| hsa-miR-744-5p | ANGPTL2 | FASLG | IL1A |  |  |
| hsa-miR-744-5p | CXCL16 | FASLG | IL1B |  |  |
| hsa-miR-744-5p | LGALS3 | FASLG | IL7 |  |  |
| hsa-miR-744-5p | MIF | FASLG | ICAM1 |  |  |
| hsa-miR-744-5p | PYGB | FASLG | IFNA1 |  |  |
| hsa-miR-744-5p | SERP1 | FASLG | IL18 |  |  |
| hsa-miR-744-5p | SIRT1 | FASLG | TNFSF4 |  |  |
| hsa-miR-744-5p | SMAD3 | FASLG | VCAM1 |  |  |
| hsa-miR-744-5p | TGFB1 | FASLG | TNFRSF11B | |  |
| hsa-miR-744-5p | TNC | FASLG | HSPA4 |  |  |
| hsa-miR-873-5p | CAD | FASLG | CXCL8 |  |  |
| hsa-miR-873-5p | CETP | FASLG | CYCS |  |  |
| hsa-miR-873-5p | EDN1 | FASLG | MLKL |  |  |
| hsa-miR-873-5p | PTX3 | FASLG | CD28 |  |  |
| hsa-miR-873-5p | SERP1 | FASLG | CD86 |  |  |
| hsa-miR-873-5p | SIRT1 | FASLG | IL17A |  |  |
| hsa-miR-873-5p | TIMP1 | FASLG | FAS |  |  |
| hsa-miR-873-5p | TNFRSF11A | FASLG | PTGS2 |  |  |
| hsa-miR-873-5p | TNFRSF1A | FASLG | HIF1A |  |  |
| hsa-miR-873-5p | TNFRSF1B | FASLG | CXCR4 |  |  |
| hsa-miR-873-5p | VEGFA | FASLG | INS |  |  |
| hsa-miR-509-3-5p | CRP | FASLG | ENTPD1 |  |  |
| hsa-miR-509-3-5p | HIF1A | FASLG | MMP9 |  |  |
| hsa-miR-509-3-5p | NRDC | FASLG | IL2RA |  |  |
| hsa-miR-935 | F3 | FASLG | TLR4 |  |  |
| hsa-miR-935 | HIF1A | FASLG | CCL3 |  |  |
| hsa-miR-935 | TNC | FASLG | TNFRSF1B | |  |
| hsa-miR-935 | TXNIP | FASLG | CCL5 |  |  |
| hsa-miR-940 | ARSA | FASLG | VEGFA |  |  |
| hsa-miR-940 | BSG | FASLG | IL6 |  |  |
| hsa-miR-940 | CAD | FASLG | CD40LG |  |  |
| hsa-miR-940 | CYCS | FASLG | IL10 |  |  |
| hsa-miR-940 | EBI3 | FCGR2A | IL2RB |  |  |
| hsa-miR-940 | F2 | FCGR2A | GZMB |  |  |
| hsa-miR-940 | HIF1A | FCGR2A | MPO |  |  |
| hsa-miR-940 | LPXN | FCGR2A | CCL2 |  |  |
| hsa-miR-940 | MBL2 | FCGR2A | IL2 |  |  |
| hsa-miR-940 | MMP2 | FCGR2A | IFNG |  |  |
| hsa-miR-940 | SERPINA1 | FCGR2A | IL5 |  |  |
| hsa-miR-940 | VCAM1 | FCGR2A | LGALS3 |  |  |
| hsa-miR-940 | YWHAZ | FCGR2A | CRP |  |  |
| hsa-miR-943 | CAD | FCGR2A | APCS |  |  |
| hsa-miR-320b | ADM | FCGR2A | IL1RN |  |  |
| hsa-miR-320b | BDNF | FCGR2A | TLR2 |  |  |
| hsa-miR-320b | CD63 | FCGR2A | VWF |  |  |
| hsa-miR-320b | MMP28 | FCGR2A | ITGA2B |  |  |
| hsa-miR-320b | PARP1 | FCGR2A | IL1A |  |  |
| hsa-miR-320b | SIRT1 | FCGR2A | IL1B |  |  |
| hsa-miR-320b | SMAD3 | FCGR2A | SELP |  |  |
| hsa-miR-320b | SULT1E1 | FCGR2A | IL7 |  |  |
| hsa-miR-320b | TNFRSF1A | FCGR2A | ICAM1 |  |  |
| hsa-miR-320b | TNFSF14 | FCGR2A | ITIH4 |  |  |
| hsa-miR-320b | TXNIP | FCGR2A | IL33 |  |  |
| hsa-miR-320b | UBAP1 | FCGR2A | MBL2 |  |  |
| hsa-miR-320c | ADM | FCGR2A | TNFSF4 |  |  |
| hsa-miR-320c | CD63 | FCGR2A | FAS |  |  |
| hsa-miR-320c | CYCS | FCGR2A | HLA-DRB1 | |  |
| hsa-miR-320c | PARP1 | FCGR2A | IL18 |  |  |
| hsa-miR-320c | SIRT1 | FCGR2A | MYD88 |  |  |
| hsa-miR-320c | SMAD3 | FCGR2A | CXCR4 |  |  |
| hsa-miR-320c | TNFRSF1A | FCGR2A | CTSS |  |  |
| hsa-miR-320c | TNFSF14 | FCGR2A | CCL5 |  |  |
| hsa-miR-320c | TXNIP | FCGR2A | CSF1 |  |  |
| hsa-miR-320c | UBAP1 | FCGR2A | VEGFA |  |  |
| hsa-miR-1185-5p | MRAS | FCGR2A | IFNA1 |  |  |
| hsa-miR-1185-5p | PAPPA | FCGR2A | GP1BA |  |  |
| hsa-miR-1286 | CAD | FCGR2A | CCL3 |  |  |
| hsa-miR-1286 | GDF15 | FCGR2A | CX3CR1 |  |  |
| hsa-miR-1299 | BSG | FCGR2A | CD40LG |  |  |
| hsa-miR-1299 | UBC | FCGR2A | ITGA2 |  |  |
| hsa-miR-1260a | F2 | FCGR2A | IL2RA |  |  |
| hsa-miR-1260a | HIF1A | FCGR2A | CD28 |  |  |
| hsa-miR-1260a | PARP1 | FCGR2A | IL17A |  |  |
| hsa-miR-1260a | TNFSF14 | FCGR2A | IL6 |  |  |
| hsa-miR-1260a | UBC | FCGR2A | CD163 |  |  |
| hsa-miR-1273a | UBC | FCGR2A | IGF1 |  |  |
| hsa-miR-1276 | HIF1A | FCGR2A | IL10 |  |  |
| hsa-miR-1276 | HMGCR | FCGR2A | PTX3 |  |  |
| hsa-miR-1276 | IL1A | FCGR2A | PECAM1 |  |  |
| hsa-miR-1276 | INTS2 | FCGR2A | TLR4 |  |  |
| hsa-miR-1276 | MORF4L2 | FCGR2A | TNF |  |  |
| hsa-miR-1276 | SMAD3 | FCGR2A | CD14 |  |  |
| hsa-miR-1276 | YWHAZ | FCGR2A | CD86 |  |  |
| hsa-miR-320d | ADM | FCGR2A | PF4 |  |  |
| hsa-miR-320d | CD63 | FCGR2A | CXCL8 |  |  |
| hsa-miR-320d | PARP1 | FCGR2A | ITGB2 |  |  |
| hsa-miR-320d | SIRT1 | FERMT3 | SELPLG |  |  |
| hsa-miR-320d | SMAD3 | FERMT3 | ITGA2B |  |  |
| hsa-miR-320d | TNFRSF1A | FERMT3 | SELP |  |  |
| hsa-miR-320d | TNFSF14 | FERMT3 | ICAM1 |  |  |
| hsa-miR-320d | TXNIP | FERMT3 | LPXN |  |  |
| hsa-miR-320d | UBAP1 | FERMT3 | ITGB2 |  |  |
| hsa-miR-675-3p | ADM | FERMT3 | ITGB3 |  |  |
| hsa-miR-1913 | PARP1 | FGA | CPB2 |  |  |
| hsa-miR-224-3p | MON2 | FGA | PLAT |  |  |
| hsa-miR-224-3p | MORF4L2 | FGA | HRG |  |  |
| hsa-miR-224-3p | YWHAZ | FGA | APOB |  |  |
| hsa-miR-764 | GDF15 | FGA | APOA1 |  |  |
| hsa-miR-764 | PIK3C2A | FGA | LGALS3 |  |  |
| hsa-miR-711 | TXNIP | FGA | APCS |  |  |
| hsa-miR-3120-3p | ADM | FGA | VWF |  |  |
| hsa-miR-3125 | FABP3 | FGA | ITGA2B |  |  |
| hsa-miR-3128 | HIF1A | FGA | CP |  |  |
| hsa-miR-3128 | PARP1 | FGA | KNG1 |  |  |
| hsa-miR-3134 | MYDGF | FGA | ITIH4 |  |  |
| hsa-miR-3140-3p | ADM | FGA | IGFBP1 |  |  |
| hsa-miR-3140-3p | HMGCR | FGA | HABP2 |  |  |
| hsa-miR-1273c | MBL2 | FGA | SAA4 |  |  |
| hsa-miR-3148 | HLA-DRB1 | FGA | CST3 |  |  |
| hsa-miR-3148 | HLA-DRB1 | FGA | IL6 |  |  |
| hsa-miR-3148 | HLA-DRB1 | FGA | AGT |  |  |
| hsa-miR-3148 | HLA-DRB1 | FGA | MBL2 |  |  |
| hsa-miR-3148 | HLA-DRB1 | FGA | CTSS |  |  |
| hsa-miR-3148 | HLA-DRB1 | FGA | F3 |  |  |
| hsa-miR-3152-3p | CD40LG | FGA | INS |  |  |
| hsa-miR-3163 | VEGFA | FGA | TLR4 |  |  |
| hsa-miR-3163 | YWHAZ | FGA | SERPINA1 | |  |
| hsa-miR-3166 | YWHAZ | FGA | F10 |  |  |
| hsa-miR-1260b | CAD | FGA | F7 |  |  |
| hsa-miR-1260b | F2 | FGA | PLG |  |  |
| hsa-miR-1260b | HIF1A | FGA | ITGB2 |  |  |
| hsa-miR-1260b | PARP1 | FGA | SERPINF2 |  |  |
| hsa-miR-1260b | TNFSF14 | FGA | PLAU |  |  |
| hsa-miR-1260b | UBC | FGA | ITGB3 |  |  |
| hsa-miR-3168 | VEGFA | FGA | SERPINA5 | |  |
| hsa-miR-3175 | PCSK9 | FGA | SERPINC1 | |  |
| hsa-miR-3175 | TXNIP | FGA | F2 |  |  |
| hsa-miR-3175 | YWHAZ | FGF21 | SIRT1 |  |  |
| hsa-miR-3177-3p | HSPA4 | FGF21 | RETN |  |  |
| hsa-miR-548w | SIRT1 | FGF21 | TGFB1 |  |  |
| hsa-miR-3183 | VCAM1 | FGF21 | HGF |  |  |
| hsa-miR-3184-5p | OLR1 | FGF21 | NAMPT |  |  |
| hsa-miR-3184-5p | TXNIP | FGF21 | CCL2 |  |  |
| hsa-miR-3186-3p | ICAM1 | FGF21 | APOB |  |  |
| hsa-miR-3188 | ADM | FGF21 | GDF15 |  |  |
| hsa-miR-3188 | COG2 | FGF21 | CRP |  |  |
| hsa-miR-3188 | ICAM1 | FGF21 | IL1B |  |  |
| hsa-miR-320e | S100A4 | FGF21 | PPARGC1A | |  |
| hsa-miR-320e | TIMP1 | FGF21 | IGFBP1 |  |  |
| hsa-miR-3197 | IGF1 | FGF21 | PPARG |  |  |
| hsa-miR-4295 | ARSA | FGF21 | MMP3 |  |  |
| hsa-miR-4295 | EDN1 | FGF21 | IGF1 |  |  |
| hsa-miR-4295 | PARP1 | FGF21 | CXCL8 |  |  |
| hsa-miR-4295 | RPS27A | FGF21 | LEP |  |  |
| hsa-miR-4295 | TXNIP | FGF21 | SERPINA12 | |  |
| hsa-miR-4295 | UBC | FGF21 | DPP4 |  |  |
| hsa-miR-4294 | ADAMTS4 | FGF21 | BGLAP |  |  |
| hsa-miR-4294 | APOA1 | FGF21 | TEK |  |  |
| hsa-miR-4294 | BSG | FGF21 | INS |  |  |
| hsa-miR-4301 | SEMA4D | FGF21 | IL6 |  |  |
| hsa-miR-4311 | MBL2 | FGF21 | TNF |  |  |
| hsa-miR-4311 | PPARGC1A | FGF21 | IL10 |  |  |
| hsa-miR-4325 | CYP2C19 | FGF21 | BDNF |  |  |
| hsa-miR-4325 | FASLG | FGF21 | ANGPT1 |  |  |
| hsa-miR-4262 | VCAM1 | FGF21 | PGF |  |  |
| hsa-miR-2355-5p | HMGCR | FGF21 | PECAM1 |  |  |
| hsa-miR-2355-5p | HSPA4 | FGF21 | VEGFA |  |  |
| hsa-miR-2355-5p | LGALS3 | FSD1 | FST |  |  |
| hsa-miR-2355-5p | PARP1 | FST | FSD1 |  |  |
| hsa-miR-2355-5p | SIRT1 | FST | TGFB1 |  |  |
| hsa-miR-2355-5p | SMAD3 | FST | HGF |  |  |
| hsa-miR-2355-5p | TNC | FST | GDF15 |  |  |
| hsa-miR-4275 | FCGR2A | FST | IL6 |  |  |
| hsa-miR-4286 | HSPA4 | FST | ANGPT2 |  |  |
| hsa-miR-4286 | SEMA4D | FST | CTGF |  |  |
| hsa-miR-4286 | TNC | FST | LEP |  |  |
| hsa-miR-4286 | YWHAZ | FST | VEGFA |  |  |
| hsa-miR-4330 | INTS2 | FST | INS |  |  |
| hsa-miR-4330 | TXNIP | FST | IGFBP1 |  |  |
| hsa-miR-3605-5p | CXCR4 | FST | SMAD3 |  |  |
| hsa-miR-3605-5p | MRAS | FST | IGF1 |  |  |
| hsa-miR-3605-5p | TXNIP | FST | BMP6 |  |  |
| hsa-miR-3607-3p | CYCS | GAS6 | HGF |  |  |
| hsa-miR-3607-3p | PIK3C2A | GAS6 | LGALS3 |  |  |
| hsa-miR-3612 | HMOX1 | GAS6 | BGLAP |  |  |
| hsa-miR-3612 | PCSK9 | GAS6 | TNF |  |  |
| hsa-miR-3612 | VCAM1 | GAS6 | IL6 |  |  |
| hsa-miR-1273e | AHR | GDF15 | CX3CL1 |  |  |
| hsa-miR-1273e | ITGA2 | GDF15 | TIMP1 |  |  |
| hsa-miR-1273e | LPXN | GDF15 | SERPINE1 |  |  |
| hsa-miR-1273e | VCAM1 | GDF15 | MPO |  |  |
| hsa-miR-3668 | HIF1A | GDF15 | CCL2 |  |  |
| hsa-miR-3913-5p | ADAMTS4 | GDF15 | TNNT2 |  |  |
| hsa-miR-3913-5p | AHR | GDF15 | EPO |  |  |
| hsa-miR-3913-5p | GP5 | GDF15 | IL1B |  |  |
| hsa-miR-3913-5p | IGF1 | GDF15 | TNF |  |  |
| hsa-miR-3913-5p | ITGB3 | GDF15 | FABP3 |  |  |
| hsa-miR-3913-5p | TNC | GDF15 | IGFBP1 |  |  |
| hsa-miR-3915 | YWHAZ | GDF15 | TNNI3 |  |  |
| hsa-miR-374c-5p | ADM | GDF15 | PTX3 |  |  |
| hsa-miR-374c-5p | TNC | GDF15 | VEGFA |  |  |
| hsa-miR-301a-5p | BSG | GDF15 | CXCL8 |  |  |
| hsa-miR-301a-5p | CAD | GDF15 | LCN2 |  |  |
| hsa-miR-301a-5p | TNC | GDF15 | FST |  |  |
| hsa-miR-301a-5p | TXNIP | GDF15 | TNNT1 |  |  |
| hsa-miR-374c-3p | PIK3C2A | GDF15 | ADM |  |  |
| hsa-miR-374c-3p | YWHAZ | GDF15 | NPPB |  |  |
|  |  | GDF15 | CTGF |  |  |
|  |  | GDF15 | IL6 |  |  |
|  |  | GDF15 | CST3 |  |  |
|  |  | GDF15 | FGF21 |  |  |
|  |  | GDF15 | CRP |  |  |
|  |  | GDF15 | LGALS3 |  |  |
|  |  | GP1BA | SELPLG |  |  |
|  |  | GP1BA | CRP |  |  |
|  |  | GP1BA | VWF |  |  |
|  |  | GP1BA | ITGA2B |  |  |
|  |  | GP1BA | SELP |  |  |
|  |  | GP1BA | ICAM1 |  |  |
|  |  | GP1BA | KNG1 |  |  |
|  |  | GP1BA | FCGR2A |  |  |
|  |  | GP1BA | PPBP |  |  |
|  |  | GP1BA | PF4 |  |  |
|  |  | GP1BA | ITGA2 |  |  |
|  |  | GP1BA | F2 |  |  |
|  |  | GP1BA | TREML1 |  |  |
|  |  | GP1BA | F10 |  |  |
|  |  | GP1BA | THBD |  |  |
|  |  | GP1BA | CD40LG |  |  |
|  |  | GP1BA | SELE |  |  |
|  |  | GP1BA | CD63 |  |  |
|  |  | GP1BA | PECAM1 |  |  |
|  |  | GP1BA | F3 |  |  |
|  |  | GP1BA | ITGB3 |  |  |
|  |  | GP1BA | SERPINA5 | |  |
|  |  | GP1BA | GP5 |  |  |
|  |  | GP1BA | SERPINC1 | |  |
|  |  | GP1BA | ITGB2 |  |  |
|  |  | GP1BA | YWHAZ |  |  |
|  |  | GP5 | IFNG |  |  |
|  |  | GP5 | VWF |  |  |
|  |  | GP5 | ITGA2B |  |  |
|  |  | GP5 | SELP |  |  |
|  |  | GP5 | KNG1 |  |  |
|  |  | GP5 | F2 |  |  |
|  |  | GP5 | GP1BA |  |  |
|  |  | GP5 | SERPINA5 | |  |
|  |  | GP5 | CD163 |  |  |
|  |  | GP5 | SERPINC1 | |  |
|  |  | GP5 | HSPD1 |  |  |
|  |  | GP5 | YWHAZ |  |  |
|  |  | GZMB | TNFRSF1A | |  |
|  |  | GZMB | IL2RB |  |  |
|  |  | GZMB | ITGA2 |  |  |
|  |  | GZMB | PECAM1 |  |  |
|  |  | GZMB | TNFSF4 |  |  |
|  |  | GZMB | PPBP |  |  |
|  |  | GZMB | VEGFA |  |  |
|  |  | GZMB | IGF1 |  |  |
|  |  | GZMB | CXCL13 |  |  |
|  |  | GZMB | ICAM1 |  |  |
|  |  | GZMB | CYCS |  |  |
|  |  | GZMB | CXCR4 |  |  |
|  |  | GZMB | TLR4 |  |  |
|  |  | GZMB | FCGR2A |  |  |
|  |  | GZMB | TLR2 |  |  |
|  |  | GZMB | RAB3IL1 |  |  |
|  |  | GZMB | CCL2 |  |  |
|  |  | GZMB | CD163 |  |  |
|  |  | GZMB | CX3CR1 |  |  |
|  |  | GZMB | CXCL8 |  |  |
|  |  | GZMB | IL5 |  |  |
|  |  | GZMB | IL1B |  |  |
|  |  | GZMB | IFNA1 |  |  |
|  |  | GZMB | FAS |  |  |
|  |  | GZMB | IL18 |  |  |
|  |  | GZMB | IL7 |  |  |
|  |  | GZMB | IL2RA |  |  |
|  |  | GZMB | ENTPD1 |  |  |
|  |  | GZMB | IL6 |  |  |
|  |  | GZMB | CCL3 |  |  |
|  |  | GZMB | CD40LG |  |  |
|  |  | GZMB | IL17A |  |  |
|  |  | GZMB | CD86 |  |  |
|  |  | GZMB | IL10 |  |  |
|  |  | GZMB | CCL5 |  |  |
|  |  | GZMB | CD28 |  |  |
|  |  | GZMB | HSPA4 |  |  |
|  |  | GZMB | TNF |  |  |
|  |  | GZMB | FASLG |  |  |
|  |  | GZMB | IL2 |  |  |
|  |  | GZMB | IFNG |  |  |
|  |  | GZMB | PARP1 |  |  |
|  |  | HABP2 | CPB2 |  |  |
|  |  | HABP2 | SERPINE1 |  |  |
|  |  | HABP2 | HRG |  |  |
|  |  | HABP2 | KNG1 |  |  |
|  |  | HABP2 | ITIH4 |  |  |
|  |  | HABP2 | THBD |  |  |
|  |  | HABP2 | AGT |  |  |
|  |  | HABP2 | F2 |  |  |
|  |  | HABP2 | F10 |  |  |
|  |  | HABP2 | SERPINF2 |  |  |
|  |  | HABP2 | F7 |  |  |
|  |  | HABP2 | SERPINC1 | |  |
|  |  | HABP2 | MBL2 |  |  |
|  |  | HABP2 | FGA |  |  |
|  |  | HDAC9 | SIRT1 |  |  |
|  |  | HDAC9 | PARP1 |  |  |
|  |  | HDAC9 | MORF4L2 | |  |
|  |  | HDAC9 | ENSP00000485396 | |  |
|  |  | HDAC9 | HIF1A |  |  |
|  |  | HGF | MIF |  |  |
|  |  | HGF | HMOX1 |  |  |
|  |  | HGF | TIMP1 |  |  |
|  |  | HGF | MMP2 |  |  |
|  |  | HGF | RETN |  |  |
|  |  | HGF | TGFB1 |  |  |
|  |  | HGF | IL18 |  |  |
|  |  | HGF | SELE |  |  |
|  |  | HGF | GAS6 |  |  |
|  |  | HGF | IL1RN |  |  |
|  |  | HGF | PPARG |  |  |
|  |  | HGF | S100A4 |  |  |
|  |  | HGF | CCL11 |  |  |
|  |  | HGF | MMP8 |  |  |
|  |  | HGF | FST |  |  |
|  |  | HGF | NOS3 |  |  |
|  |  | HGF | SEMA4D |  |  |
|  |  | HGF | SMAD3 |  |  |
|  |  | HGF | CRP |  |  |
|  |  | HGF | TLR4 |  |  |
|  |  | HGF | CSF1 |  |  |
|  |  | HGF | SERPINA1 | |  |
|  |  | HGF | EPO |  |  |
|  |  | HGF | FASLG |  |  |
|  |  | HGF | CCL3 |  |  |
|  |  | HGF | IL2 |  |  |
|  |  | HGF | EDN1 |  |  |
|  |  | HGF | IL1A |  |  |
|  |  | HGF | LEP |  |  |
|  |  | HGF | IFNG |  |  |
|  |  | HGF | VWF |  |  |
|  |  | HGF | ICAM1 |  |  |
|  |  | HGF | VCAM1 |  |  |
|  |  | HGF | CCL5 |  |  |
|  |  | HGF | CTGF |  |  |
|  |  | HGF | PTGS2 |  |  |
|  |  | HGF | BDNF |  |  |
|  |  | HGF | SERPINE1 |  |  |
|  |  | HGF | HIF1A |  |  |
|  |  | HGF | IL32 |  |  |
|  |  | HGF | ANGPT2 |  |  |
|  |  | HGF | PECAM1 |  |  |
|  |  | HGF | IL1B |  |  |
|  |  | HGF | CCL2 |  |  |
|  |  | HGF | PGF |  |  |
|  |  | HGF | FGF21 |  |  |
|  |  | HGF | TNF |  |  |
|  |  | HGF | CXCR4 |  |  |
|  |  | HGF | CXCL8 |  |  |
|  |  | HGF | ANGPT1 |  |  |
|  |  | HGF | INS |  |  |
|  |  | HGF | IGF1 |  |  |
|  |  | HGF | TEK |  |  |
|  |  | HGF | RPS27A |  |  |
|  |  | HGF | UBC |  |  |
|  |  | HGF | ITGA2 |  |  |
|  |  | HGF | VEGFA |  |  |
|  |  | HGF | PLAU |  |  |
|  |  | HGF | LCN2 |  |  |
|  |  | HGF | IL17A |  |  |
|  |  | HGF | MMP3 |  |  |
|  |  | HGF | MMP1 |  |  |
|  |  | HGF | IL10 |  |  |
|  |  | HGF | MMP9 |  |  |
|  |  | HGF | IL6 |  |  |
|  |  | HGF | IL7 |  |  |
|  |  | HIF1A | SIRT1 |  |  |
|  |  | HIF1A | MIF |  |  |
|  |  | HIF1A | HMOX1 |  |  |
|  |  | HIF1A | TIMP1 |  |  |
|  |  | HIF1A | MMP2 |  |  |
|  |  | HIF1A | TGFB1 |  |  |
|  |  | HIF1A | HGF |  |  |
|  |  | HIF1A | NAMPT |  |  |
|  |  | HIF1A | SERPINE1 |  |  |
|  |  | HIF1A | CCL2 |  |  |
|  |  | HIF1A | NFKB1 |  |  |
|  |  | HIF1A | IL2 |  |  |
|  |  | HIF1A | IFNG |  |  |
|  |  | HIF1A | AHR |  |  |
|  |  | HIF1A | EPO |  |  |
|  |  | HIF1A | TLR2 |  |  |
|  |  | HIF1A | VWF |  |  |
|  |  | HIF1A | IL1A |  |  |
|  |  | HIF1A | IL1B |  |  |
|  |  | HIF1A | ICAM1 |  |  |
|  |  | HIF1A | PPARGC1A | |  |
|  |  | HIF1A | REN |  |  |
|  |  | HIF1A | RPS27A |  |  |
|  |  | HIF1A | IL18 |  |  |
|  |  | HIF1A | PPARG |  |  |
|  |  | HIF1A | ACE |  |  |
|  |  | HIF1A | VCAM1 |  |  |
|  |  | HIF1A | NOS3 |  |  |
|  |  | HIF1A | MMP3 |  |  |
|  |  | HIF1A | IGF1 |  |  |
|  |  | HIF1A | HSPA4 |  |  |
|  |  | HIF1A | CXCL8 |  |  |
|  |  | HIF1A | CYCS |  |  |
|  |  | HIF1A | PLG |  |  |
|  |  | HIF1A | LEP |  |  |
|  |  | HIF1A | ANGPT2 |  |  |
|  |  | HIF1A | MMP1 |  |  |
|  |  | HIF1A | CSF1 |  |  |
|  |  | HIF1A | SELE |  |  |
|  |  | HIF1A | SMAD3 |  |  |
|  |  | HIF1A | BSG |  |  |
|  |  | HIF1A | IL17A |  |  |
|  |  | HIF1A | CX3CR1 |  |  |
|  |  | HIF1A | CD163 |  |  |
|  |  | HIF1A | PARP1 |  |  |
|  |  | HIF1A | PARK2 |  |  |
|  |  | HIF1A | PTGS2 |  |  |
|  |  | HIF1A | FASLG |  |  |
|  |  | HIF1A | CTGF |  |  |
|  |  | HIF1A | BGLAP |  |  |
|  |  | HIF1A | ENTPD1 |  |  |
|  |  | HIF1A | MMP9 |  |  |
|  |  | HIF1A | PLAU |  |  |
|  |  | HIF1A | TLR4 |  |  |
|  |  | HIF1A | EDN1 |  |  |
|  |  | HIF1A | TEK |  |  |
|  |  | HIF1A | YWHAZ |  |  |
|  |  | HIF1A | INS |  |  |
|  |  | HIF1A | MB |  |  |
|  |  | HIF1A | IL6 |  |  |
|  |  | HIF1A | CXCR4 |  |  |
|  |  | HIF1A | TNF |  |  |
|  |  | HIF1A | MYD88 |  |  |
|  |  | HIF1A | HDAC9 |  |  |
|  |  | HIF1A | IL10 |  |  |
|  |  | HIF1A | BDNF |  |  |
|  |  | HIF1A | BRCA1 |  |  |
|  |  | HIF1A | ANGPT1 |  |  |
|  |  | HIF1A | ADM |  |  |
|  |  | HIF1A | CCL5 |  |  |
|  |  | HIF1A | TXNIP |  |  |
|  |  | HIF1A | PGF |  |  |
|  |  | HIF1A | PECAM1 |  |  |
|  |  | HIF1A | UBC |  |  |
|  |  | HIF1A | VEGFA |  |  |
|  |  | HLA-DRB1 | IL2RB |  |  |
|  |  | HLA-DRB1 | IL2 |  |  |
|  |  | HLA-DRB1 | IFNG |  |  |
|  |  | HLA-DRB1 | CRP |  |  |
|  |  | HLA-DRB1 | IL1B |  |  |
|  |  | HLA-DRB1 | FCGR2A |  |  |
|  |  | HLA-DRB1 | CD28 |  |  |
|  |  | HLA-DRB1 | CD86 |  |  |
|  |  | HLA-DRB1 | IL17A |  |  |
|  |  | HLA-DRB1 | IL6 |  |  |
|  |  | HLA-DRB1 | ITGB2 |  |  |
|  |  | HLA-DRB1 | IL10 |  |  |
|  |  | HLA-DRB1 | IL2RA |  |  |
|  |  | HLA-DRB1 | S100A8 |  |  |
|  |  | HLA-DRB1 | TNF |  |  |
|  |  | HLA-DRB1 | INS |  |  |
|  |  | HLA-DRB1 | CTSS |  |  |
|  |  | HMGCR | CETP |  |  |
|  |  | HMGCR | SIRT1 |  |  |
|  |  | HMGCR | APOB |  |  |
|  |  | HMGCR | APOA1 |  |  |
|  |  | HMGCR | CRP |  |  |
|  |  | HMGCR | PPARGC1A | |  |
|  |  | HMGCR | PPARG |  |  |
|  |  | HMGCR | TNF |  |  |
|  |  | HMGCR | LEP |  |  |
|  |  | HMGCR | IL6 |  |  |
|  |  | HMGCR | NOS3 |  |  |
|  |  | HMGCR | ACE |  |  |
|  |  | HMGCR | INS |  |  |
|  |  | HMGCR | PCSK9 |  |  |
|  |  | HMOX1 | TNFRSF1A | |  |
|  |  | HMOX1 | SIRT1 |  |  |
|  |  | HMOX1 | CCL5 |  |  |
|  |  | HMOX1 | LEP |  |  |
|  |  | HMOX1 | THBD |  |  |
|  |  | HMOX1 | CCL3 |  |  |
|  |  | HMOX1 | CTGF |  |  |
|  |  | HMOX1 | MMP3 |  |  |
|  |  | HMOX1 | IL2 |  |  |
|  |  | HMOX1 | IL1A |  |  |
|  |  | HMOX1 | MMP1 |  |  |
|  |  | HMOX1 | TXNIP |  |  |
|  |  | HMOX1 | YWHAZ |  |  |
|  |  | HMOX1 | BRCA1 |  |  |
|  |  | HMOX1 | HGF |  |  |
|  |  | HMOX1 | PECAM1 |  |  |
|  |  | HMOX1 | IL17A |  |  |
|  |  | HMOX1 | TGFB1 |  |  |
|  |  | HMOX1 | SELP |  |  |
|  |  | HMOX1 | IL18 |  |  |
|  |  | HMOX1 | ACE |  |  |
|  |  | HMOX1 | IGF1 |  |  |
|  |  | HMOX1 | REN |  |  |
|  |  | HMOX1 | IFNG |  |  |
|  |  | HMOX1 | TIMP1 |  |  |
|  |  | HMOX1 | CRP |  |  |
|  |  | HMOX1 | MMP2 |  |  |
|  |  | HMOX1 | CYBA |  |  |
|  |  | HMOX1 | MYD88 |  |  |
|  |  | HMOX1 | SELE |  |  |
|  |  | HMOX1 | LCN2 |  |  |
|  |  | HMOX1 | AHR |  |  |
|  |  | HMOX1 | SERPINE1 |  |  |
|  |  | HMOX1 | EPO |  |  |
|  |  | HMOX1 | MB |  |  |
|  |  | HMOX1 | BDNF |  |  |
|  |  | HMOX1 | TLR2 |  |  |
|  |  | HMOX1 | EDN1 |  |  |
|  |  | HMOX1 | INS |  |  |
|  |  | HMOX1 | VCAM1 |  |  |
|  |  | HMOX1 | CD163 |  |  |
|  |  | HMOX1 | MMP9 |  |  |
|  |  | HMOX1 | MPO |  |  |
|  |  | HMOX1 | PPARG |  |  |
|  |  | HMOX1 | ICAM1 |  |  |
|  |  | HMOX1 | CXCL8 |  |  |
|  |  | HMOX1 | TLR4 |  |  |
|  |  | HMOX1 | VEGFA |  |  |
|  |  | HMOX1 | HSPA4 |  |  |
|  |  | HMOX1 | CCL2 |  |  |
|  |  | HMOX1 | IL10 |  |  |
|  |  | HMOX1 | HIF1A |  |  |
|  |  | HMOX1 | CYCS |  |  |
|  |  | HMOX1 | PPARGC1A | |  |
|  |  | HMOX1 | PTGS2 |  |  |
|  |  | HMOX1 | IL1B |  |  |
|  |  | HMOX1 | TNF |  |  |
|  |  | HMOX1 | NOS3 |  |  |
|  |  | HMOX1 | IL6 |  |  |
|  |  | HMOX1 | SERP1 |  |  |
|  |  | HMOX1 | CP |  |  |
|  |  | HRG | CPB2 |  |  |
|  |  | HRG | PLAT |  |  |
|  |  | HRG | SERPINE1 |  |  |
|  |  | HRG | CP |  |  |
|  |  | HRG | THBD |  |  |
|  |  | HRG | IL6 |  |  |
|  |  | HRG | PF4 |  |  |
|  |  | HRG | PGF |  |  |
|  |  | HRG | APOA1 |  |  |
|  |  | HRG | ITIH4 |  |  |
|  |  | HRG | SERPINA1 | |  |
|  |  | HRG | APCS |  |  |
|  |  | HRG | F3 |  |  |
|  |  | HRG | F2 |  |  |
|  |  | HRG | MBL2 |  |  |
|  |  | HRG | SERPINF2 |  |  |
|  |  | HRG | APOB |  |  |
|  |  | HRG | KNG1 |  |  |
|  |  | HRG | HABP2 |  |  |
|  |  | HRG | PLAU |  |  |
|  |  | HRG | FGA |  |  |
|  |  | HRG | SERPINC1 | |  |
|  |  | HRG | PLG |  |  |
|  |  | HSPA4 | TNFRSF1A | |  |
|  |  | HSPA4 | SIRT1 |  |  |
|  |  | HSPA4 | HMOX1 |  |  |
|  |  | HSPA4 | GZMB |  |  |
|  |  | HSPA4 | MMP2 |  |  |
|  |  | HSPA4 | MPO |  |  |
|  |  | HSPA4 | CCL2 |  |  |
|  |  | HSPA4 | NFKB1 |  |  |
|  |  | HSPA4 | IL2 |  |  |
|  |  | HSPA4 | APOB |  |  |
|  |  | HSPA4 | TLR2 |  |  |
|  |  | HSPA4 | IL1B |  |  |
|  |  | HSPA4 | ICAM1 |  |  |
|  |  | HSPA4 | RPS27A |  |  |
|  |  | HSPA4 | NOS3 |  |  |
|  |  | HSPA4 | IGF1 |  |  |
|  |  | HSPA4 | FASLG |  |  |
|  |  | HSPA4 | MB |  |  |
|  |  | HSPA4 | CD86 |  |  |
|  |  | HSPA4 | SERPINA1 | |  |
|  |  | HSPA4 | THBD |  |  |
|  |  | HSPA4 | BDNF |  |  |
|  |  | HSPA4 | AGER |  |  |
|  |  | HSPA4 | CXCR4 |  |  |
|  |  | HSPA4 | MYD88 |  |  |
|  |  | HSPA4 | FAS |  |  |
|  |  | HSPA4 | CXCL8 |  |  |
|  |  | HSPA4 | IL10 |  |  |
|  |  | HSPA4 | PTGS2 |  |  |
|  |  | HSPA4 | INS |  |  |
|  |  | HSPA4 | YWHAZ |  |  |
|  |  | HSPA4 | IL6 |  |  |
|  |  | HSPA4 | MMP9 |  |  |
|  |  | HSPA4 | TNF |  |  |
|  |  | HSPA4 | S100A4 |  |  |
|  |  | HSPA4 | CD63 |  |  |
|  |  | HSPA4 | VEGFA |  |  |
|  |  | HSPA4 | PARP1 |  |  |
|  |  | HSPA4 | CYCS |  |  |
|  |  | HSPA4 | SMAD3 |  |  |
|  |  | HSPA4 | HSPD1 |  |  |
|  |  | HSPA4 | PARK2 |  |  |
|  |  | HSPA4 | OLR1 |  |  |
|  |  | HSPA4 | TLR4 |  |  |
|  |  | HSPA4 | HIF1A |  |  |
|  |  | HSPD1 | IFNG |  |  |
|  |  | HSPD1 | CRP |  |  |
|  |  | HSPD1 | TLR2 |  |  |
|  |  | HSPD1 | IL1B |  |  |
|  |  | HSPD1 | HSPA4 |  |  |
|  |  | HSPD1 | CD14 |  |  |
|  |  | HSPD1 | CXCL8 |  |  |
|  |  | HSPD1 | CYCS |  |  |
|  |  | HSPD1 | PLG |  |  |
|  |  | HSPD1 | OLR1 |  |  |
|  |  | HSPD1 | PARK2 |  |  |
|  |  | HSPD1 | TLR4 |  |  |
|  |  | HSPD1 | INS |  |  |
|  |  | HSPD1 | IL6 |  |  |
|  |  | HSPD1 | GP5 |  |  |
|  |  | HSPD1 | TNF |  |  |
|  |  | HSPD1 | IL10 |  |  |
|  |  | HSPD1 | MYD88 |  |  |
|  |  | ICAM1 | CX3CL1 |  |  |
|  |  | ICAM1 | TNFRSF1A | |  |
|  |  | ICAM1 | SIRT1 |  |  |
|  |  | ICAM1 | MIF |  |  |
|  |  | ICAM1 | HMOX1 |  |  |
|  |  | ICAM1 | GZMB |  |  |
|  |  | ICAM1 | TIMP1 |  |  |
|  |  | ICAM1 | MMP2 |  |  |
|  |  | ICAM1 | PLAT |  |  |
|  |  | ICAM1 | RETN |  |  |
|  |  | ICAM1 | TGFB1 |  |  |
|  |  | ICAM1 | HGF |  |  |
|  |  | ICAM1 | SERPINE1 |  |  |
|  |  | ICAM1 | MPO |  |  |
|  |  | ICAM1 | CCL2 |  |  |
|  |  | ICAM1 | NFKB1 |  |  |
|  |  | ICAM1 | IL2 |  |  |
|  |  | ICAM1 | SELPLG |  |  |
|  |  | ICAM1 | IFNG |  |  |
|  |  | ICAM1 | IL5 |  |  |
|  |  | ICAM1 | APOB |  |  |
|  |  | ICAM1 | MMP8 |  |  |
|  |  | ICAM1 | APOA1 |  |  |
|  |  | ICAM1 | EPO |  |  |
|  |  | ICAM1 | LGALS3 |  |  |
|  |  | ICAM1 | CRP |  |  |
|  |  | ICAM1 | IL1RN |  |  |
|  |  | ICAM1 | TLR2 |  |  |
|  |  | ICAM1 | VWF |  |  |
|  |  | ICAM1 | CYBA |  |  |
|  |  | ICAM1 | ITGA2B |  |  |
|  |  | ICAM1 | IL1A |  |  |
|  |  | ICAM1 | IL1B |  |  |
|  |  | ICAM1 | SELP |  |  |
|  |  | ICAM1 | IL7 |  |  |
|  |  | ICAM1 | SERPINA1 | |  |
|  |  | ICAM1 | ENTPD1 |  |  |
|  |  | ICAM1 | BDNF |  |  |
|  |  | ICAM1 | PTX3 |  |  |
|  |  | ICAM1 | TNFRSF11B | |  |
|  |  | ICAM1 | AGTR1 |  |  |
|  |  | ICAM1 | PLAU |  |  |
|  |  | ICAM1 | CXCL16 |  |  |
|  |  | ICAM1 | BSG |  |  |
|  |  | ICAM1 | CYCS |  |  |
|  |  | ICAM1 | TNFSF4 |  |  |
|  |  | ICAM1 | SERPINC1 | |  |
|  |  | ICAM1 | RNASE3 |  |  |
|  |  | ICAM1 | KNG1 |  |  |
|  |  | ICAM1 | GP1BA |  |  |
|  |  | ICAM1 | IL33 |  |  |
|  |  | ICAM1 | HSPA4 |  |  |
|  |  | ICAM1 | DPP4 |  |  |
|  |  | ICAM1 | PF4 |  |  |
|  |  | ICAM1 | CD163 |  |  |
|  |  | ICAM1 | LCN2 |  |  |
|  |  | ICAM1 | PGF |  |  |
|  |  | ICAM1 | ITGA2 |  |  |
|  |  | ICAM1 | AGT |  |  |
|  |  | ICAM1 | IGF1 |  |  |
|  |  | ICAM1 | CD63 |  |  |
|  |  | ICAM1 | ANGPT1 |  |  |
|  |  | ICAM1 | CX3CR1 |  |  |
|  |  | ICAM1 | FCGR2A |  |  |
|  |  | ICAM1 | CTGF |  |  |
|  |  | ICAM1 | ITGB3 |  |  |
|  |  | ICAM1 | CXCL13 |  |  |
|  |  | ICAM1 | AGER |  |  |
|  |  | ICAM1 | LEP |  |  |
|  |  | ICAM1 | TEK |  |  |
|  |  | ICAM1 | REN |  |  |
|  |  | ICAM1 | FAS |  |  |
|  |  | ICAM1 | MMP1 |  |  |
|  |  | ICAM1 | MYD88 |  |  |
|  |  | ICAM1 | IFNA1 |  |  |
|  |  | ICAM1 | ANGPT2 |  |  |
|  |  | ICAM1 | CCL11 |  |  |
|  |  | ICAM1 | OLR1 |  |  |
|  |  | ICAM1 | PPARG |  |  |
|  |  | ICAM1 | ACE |  |  |
|  |  | ICAM1 | MMP3 |  |  |
|  |  | ICAM1 | FERMT3 |  |  |
|  |  | ICAM1 | IL2RA |  |  |
|  |  | ICAM1 | FASLG |  |  |
|  |  | ICAM1 | PLG |  |  |
|  |  | ICAM1 | HIF1A |  |  |
|  |  | ICAM1 | ITIH4 |  |  |
|  |  | ICAM1 | CXCR4 |  |  |
|  |  | ICAM1 | IL18 |  |  |
|  |  | ICAM1 | IL17A |  |  |
|  |  | ICAM1 | F3 |  |  |
|  |  | ICAM1 | CD28 |  |  |
|  |  | ICAM1 | EDN1 |  |  |
|  |  | ICAM1 | CD40LG |  |  |
|  |  | ICAM1 | CCL3 |  |  |
|  |  | ICAM1 | PTGS2 |  |  |
|  |  | ICAM1 | THBD |  |  |
|  |  | ICAM1 | NOS3 |  |  |
|  |  | ICAM1 | CCL5 |  |  |
|  |  | ICAM1 | VEGFA |  |  |
|  |  | ICAM1 | INS |  |  |
|  |  | ICAM1 | MMP9 |  |  |
|  |  | ICAM1 | TLR4 |  |  |
|  |  | ICAM1 | PECAM1 |  |  |
|  |  | ICAM1 | CXCL8 |  |  |
|  |  | ICAM1 | TNF |  |  |
|  |  | ICAM1 | IL6 |  |  |
|  |  | ICAM1 | SELE |  |  |
|  |  | ICAM1 | VCAM1 |  |  |
|  |  | ICAM1 | TNFRSF1B | |  |
|  |  | ICAM1 | CSF1 |  |  |
|  |  | ICAM1 | IL10 |  |  |
|  |  | ICAM1 | CD86 |  |  |
|  |  | ICAM1 | ITGB2 |  |  |
|  |  | IFNA1 | CX3CL1 |  |  |
|  |  | IFNA1 | TNFRSF1A | |  |
|  |  | IFNA1 | IL2RB |  |  |
|  |  | IFNA1 | GZMB |  |  |
|  |  | IFNA1 | CCL2 |  |  |
|  |  | IFNA1 | NFKB1 |  |  |
|  |  | IFNA1 | IL2 |  |  |
|  |  | IFNA1 | IFNG |  |  |
|  |  | IFNA1 | IL5 |  |  |
|  |  | IFNA1 | APOA1 |  |  |
|  |  | IFNA1 | EPO |  |  |
|  |  | IFNA1 | CRP |  |  |
|  |  | IFNA1 | IL1RN |  |  |
|  |  | IFNA1 | TLR2 |  |  |
|  |  | IFNA1 | IL1A |  |  |
|  |  | IFNA1 | IL1B |  |  |
|  |  | IFNA1 | IL7 |  |  |
|  |  | IFNA1 | ICAM1 |  |  |
|  |  | IFNA1 | ITIH4 |  |  |
|  |  | IFNA1 | FCGR2A |  |  |
|  |  | IFNA1 | VCAM1 |  |  |
|  |  | IFNA1 | CD163 |  |  |
|  |  | IFNA1 | CXCL13 |  |  |
|  |  | IFNA1 | MMP9 |  |  |
|  |  | IFNA1 | PTGS2 |  |  |
|  |  | IFNA1 | FAS |  |  |
|  |  | IFNA1 | TNFSF4 |  |  |
|  |  | IFNA1 | IL33 |  |  |
|  |  | IFNA1 | THBD |  |  |
|  |  | IFNA1 | CXCR4 |  |  |
|  |  | IFNA1 | FASLG |  |  |
|  |  | IFNA1 | CSF1 |  |  |
|  |  | IFNA1 | INS |  |  |
|  |  | IFNA1 | CD28 |  |  |
|  |  | IFNA1 | CCL11 |  |  |
|  |  | IFNA1 | VEGFA |  |  |
|  |  | IFNA1 | CD40LG |  |  |
|  |  | IFNA1 | CCL3 |  |  |
|  |  | IFNA1 | CD86 |  |  |
|  |  | IFNA1 | IL17A |  |  |
|  |  | IFNA1 | CCL5 |  |  |
|  |  | IFNA1 | MYD88 |  |  |
|  |  | IFNA1 | CXCL8 |  |  |
|  |  | IFNA1 | TLR4 |  |  |
|  |  | IFNA1 | IL18 |  |  |
|  |  | IFNA1 | IL2RA |  |  |
|  |  | IFNA1 | IL10 |  |  |
|  |  | IFNA1 | IL6 |  |  |
|  |  | IFNA1 | TNF |  |  |
|  |  | IFNG | CX3CL1 |  |  |
|  |  | IFNG | TNFRSF1A | |  |
|  |  | IFNG | MIF |  |  |
|  |  | IFNG | HMOX1 |  |  |
|  |  | IFNG | IL2RB |  |  |
|  |  | IFNG | GZMB |  |  |
|  |  | IFNG | TIMP1 |  |  |
|  |  | IFNG | MMP2 |  |  |
|  |  | IFNG | RETN |  |  |
|  |  | IFNG | TGFB1 |  |  |
|  |  | IFNG | HGF |  |  |
|  |  | IFNG | SERPINE1 |  |  |
|  |  | IFNG | MPO |  |  |
|  |  | IFNG | CCL2 |  |  |
|  |  | IFNG | NFKB1 |  |  |
|  |  | IFNG | IL2 |  |  |
|  |  | IFNG | F2 |  |  |
|  |  | IFNG | DPP4 |  |  |
|  |  | IFNG | VWF |  |  |
|  |  | IFNG | SMAD3 |  |  |
|  |  | IFNG | VIP |  |  |
|  |  | IFNG | ITIH4 |  |  |
|  |  | IFNG | MMP1 |  |  |
|  |  | IFNG | LCN2 |  |  |
|  |  | IFNG | PLG |  |  |
|  |  | IFNG | EDN1 |  |  |
|  |  | IFNG | MMP3 |  |  |
|  |  | IFNG | ACE |  |  |
|  |  | IFNG | GP5 |  |  |
|  |  | IFNG | F3 |  |  |
|  |  | IFNG | IL18R1 |  |  |
|  |  | IFNG | BDNF |  |  |
|  |  | IFNG | AHR |  |  |
|  |  | IFNG | HSPD1 |  |  |
|  |  | IFNG | HLA-DRB1 | |  |
|  |  | IFNG | IL37 |  |  |
|  |  | IFNG | NOS3 |  |  |
|  |  | IFNG | SELP |  |  |
|  |  | IFNG | TNFRSF1B | |  |
|  |  | IFNG | EPO |  |  |
|  |  | IFNG | PPBP |  |  |
|  |  | IFNG | HIF1A |  |  |
|  |  | IFNG | PECAM1 |  |  |
|  |  | IFNG | TNFSF4 |  |  |
|  |  | IFNG | ENTPD1 |  |  |
|  |  | IFNG | CX3CR1 |  |  |
|  |  | IFNG | ITGA2 |  |  |
|  |  | IFNG | CXCL13 |  |  |
|  |  | IFNG | CXCR4 |  |  |
|  |  | IFNG | LEP |  |  |
|  |  | IFNG | IL12A |  |  |
|  |  | IFNG | PPARG |  |  |
|  |  | IFNG | SELE |  |  |
|  |  | IFNG | CD163 |  |  |
|  |  | IFNG | LGALS3 |  |  |
|  |  | IFNG | IL33 |  |  |
|  |  | IFNG | INS |  |  |
|  |  | IFNG | CD14 |  |  |
|  |  | IFNG | IGF1 |  |  |
|  |  | IFNG | FAS |  |  |
|  |  | IFNG | IL1RN |  |  |
|  |  | IFNG | PTGS2 |  |  |
|  |  | IFNG | CSF1 |  |  |
|  |  | IFNG | VCAM1 |  |  |
|  |  | IFNG | MMP9 |  |  |
|  |  | IFNG | FCGR2A |  |  |
|  |  | IFNG | IFNA1 |  |  |
|  |  | IFNG | FASLG |  |  |
|  |  | IFNG | VEGFA |  |  |
|  |  | IFNG | CCL11 |  |  |
|  |  | IFNG | MYD88 |  |  |
|  |  | IFNG | ICAM1 |  |  |
|  |  | IFNG | CRP |  |  |
|  |  | IFNG | IL1A |  |  |
|  |  | IFNG | IL7 |  |  |
|  |  | IFNG | TLR2 |  |  |
|  |  | IFNG | TLR4 |  |  |
|  |  | IFNG | CD86 |  |  |
|  |  | IFNG | CCL5 |  |  |
|  |  | IFNG | CCL3 |  |  |
|  |  | IFNG | IL18 |  |  |
|  |  | IFNG | CD28 |  |  |
|  |  | IFNG | CXCL8 |  |  |
|  |  | IFNG | CD40LG |  |  |
|  |  | IFNG | IL5 |  |  |
|  |  | IFNG | IL17A |  |  |
|  |  | IFNG | IL1B |  |  |
|  |  | IFNG | IL2RA |  |  |
|  |  | IFNG | TNF |  |  |
|  |  | IFNG | IL6 |  |  |
|  |  | IFNG | IL10 |  |  |
|  |  | IGF1 | CX3CL1 |  |  |
|  |  | IGF1 | TNFRSF1A | |  |
|  |  | IGF1 | SIRT1 |  |  |
|  |  | IGF1 | HMOX1 |  |  |
|  |  | IGF1 | GZMB |  |  |
|  |  | IGF1 | TIMP1 |  |  |
|  |  | IGF1 | MMP2 |  |  |
|  |  | IGF1 | RETN |  |  |
|  |  | IGF1 | TGFB1 |  |  |
|  |  | IGF1 | HGF |  |  |
|  |  | IGF1 | NAMPT |  |  |
|  |  | IGF1 | SERPINE1 |  |  |
|  |  | IGF1 | CCL2 |  |  |
|  |  | IGF1 | IL2 |  |  |
|  |  | IGF1 | IFNG |  |  |
|  |  | IGF1 | APOB |  |  |
|  |  | IGF1 | MMP8 |  |  |
|  |  | IGF1 | APOA1 |  |  |
|  |  | IGF1 | EPO |  |  |
|  |  | IGF1 | CRP |  |  |
|  |  | IGF1 | CHI3L1 |  |  |
|  |  | IGF1 | FST |  |  |
|  |  | IGF1 | IL1RN |  |  |
|  |  | IGF1 | VWF |  |  |
|  |  | IGF1 | IL1A |  |  |
|  |  | IGF1 | IL1B |  |  |
|  |  | IGF1 | IL7 |  |  |
|  |  | IGF1 | ICAM1 |  |  |
|  |  | IGF1 | PPARGC1A | |  |
|  |  | IGF1 | FCGR2A |  |  |
|  |  | IGF1 | REN |  |  |
|  |  | IGF1 | IGFBP1 |  |  |
|  |  | IGF1 | IL18 |  |  |
|  |  | IGF1 | BMP6 |  |  |
|  |  | IGF1 | PPARG |  |  |
|  |  | IGF1 | ACE |  |  |
|  |  | IGF1 | VCAM1 |  |  |
|  |  | IGF1 | TNFRSF11B | |  |
|  |  | IGF1 | NOS3 |  |  |
|  |  | IGF1 | MMP3 |  |  |
|  |  | IGF1 | SERPINA1 | |  |
|  |  | IGF1 | VIP |  |  |
|  |  | IGF1 | AGTR1 |  |  |
|  |  | IGF1 | ADM |  |  |
|  |  | IGF1 | CX3CR1 |  |  |
|  |  | IGF1 | PLAU |  |  |
|  |  | IGF1 | CCL3 |  |  |
|  |  | IGF1 | BRCA1 |  |  |
|  |  | IGF1 | IL17A |  |  |
|  |  | IGF1 | ANGPT2 |  |  |
|  |  | IGF1 | HSPA4 |  |  |
|  |  | IGF1 | SMAD3 |  |  |
|  |  | IGF1 | TLR4 |  |  |
|  |  | IGF1 | CCL5 |  |  |
|  |  | IGF1 | CYCS |  |  |
|  |  | IGF1 | AGT |  |  |
|  |  | IGF1 | PGF |  |  |
|  |  | IGF1 | CSF1 |  |  |
|  |  | IGF1 | DPP4 |  |  |
|  |  | IGF1 | PTGS2 |  |  |
|  |  | IGF1 | ANGPT1 |  |  |
|  |  | IGF1 | PECAM1 |  |  |
|  |  | IGF1 | EDN1 |  |  |
|  |  | IGF1 | CXCL8 |  |  |
|  |  | IGF1 | MMP9 |  |  |
|  |  | IGF1 | IL10 |  |  |
|  |  | IGF1 | HIF1A |  |  |
|  |  | IGF1 | CTGF |  |  |
|  |  | IGF1 | CXCR4 |  |  |
|  |  | IGF1 | BGLAP |  |  |
|  |  | IGF1 | PLG |  |  |
|  |  | IGF1 | TEK |  |  |
|  |  | IGF1 | TNF |  |  |
|  |  | IGF1 | FGF21 |  |  |
|  |  | IGF1 | IL6 |  |  |
|  |  | IGF1 | LEP |  |  |
|  |  | IGF1 | VEGFA |  |  |
|  |  | IGF1 | BDNF |  |  |
|  |  | IGF1 | F2 |  |  |
|  |  | IGF1 | MMP1 |  |  |
|  |  | IGF1 | PAPPA |  |  |
|  |  | IGF1 | ITGB3 |  |  |
|  |  | IGF1 | INS |  |  |
|  |  | IGFBP1 | TIMP1 |  |  |
|  |  | IGFBP1 | SERPINE1 |  |  |
|  |  | IGFBP1 | APOB |  |  |
|  |  | IGFBP1 | APOA1 |  |  |
|  |  | IGFBP1 | GDF15 |  |  |
|  |  | IGFBP1 | CRP |  |  |
|  |  | IGFBP1 | FST |  |  |
|  |  | IGFBP1 | TYR |  |  |
|  |  | IGFBP1 | IL1B |  |  |
|  |  | IGFBP1 | PGF |  |  |
|  |  | IGFBP1 | FGF21 |  |  |
|  |  | IGFBP1 | TNF |  |  |
|  |  | IGFBP1 | CXCL8 |  |  |
|  |  | IGFBP1 | PTX3 |  |  |
|  |  | IGFBP1 | SERPINC1 | |  |
|  |  | IGFBP1 | IL6 |  |  |
|  |  | IGFBP1 | VEGFA |  |  |
|  |  | IGFBP1 | PAPPA |  |  |
|  |  | IGFBP1 | FGA |  |  |
|  |  | IGFBP1 | F2 |  |  |
|  |  | IGFBP1 | LEP |  |  |
|  |  | IGFBP1 | MMP9 |  |  |
|  |  | IGFBP1 | INS |  |  |
|  |  | IGFBP1 | IGF1 |  |  |
|  |  | IL10 | CX3CL1 |  |  |
|  |  | IL10 | TNFRSF1A | |  |
|  |  | IL10 | SIRT1 |  |  |
|  |  | IL10 | MIF |  |  |
|  |  | IL10 | HMOX1 |  |  |
|  |  | IL10 | IL2RB |  |  |
|  |  | IL10 | GZMB |  |  |
|  |  | IL10 | TIMP1 |  |  |
|  |  | IL10 | MMP2 |  |  |
|  |  | IL10 | RETN |  |  |
|  |  | IL10 | EBI3 |  |  |
|  |  | IL10 | TGFB1 |  |  |
|  |  | IL10 | HGF |  |  |
|  |  | IL10 | NAMPT |  |  |
|  |  | IL10 | SERPINE1 |  |  |
|  |  | IL10 | MPO |  |  |
|  |  | IL10 | CCL2 |  |  |
|  |  | IL10 | NFKB1 |  |  |
|  |  | IL10 | IL2 |  |  |
|  |  | IL10 | IFNG |  |  |
|  |  | IL10 | IL5 |  |  |
|  |  | IL10 | APOB |  |  |
|  |  | IL10 | MMP8 |  |  |
|  |  | IL10 | APOA1 |  |  |
|  |  | IL10 | AHR |  |  |
|  |  | IL10 | EPO |  |  |
|  |  | IL10 | LGALS3 |  |  |
|  |  | IL10 | CRP |  |  |
|  |  | IL10 | CHI3L1 |  |  |
|  |  | IL10 | IL1RN |  |  |
|  |  | IL10 | TLR2 |  |  |
|  |  | IL10 | VWF |  |  |
|  |  | IL10 | IL37 |  |  |
|  |  | IL10 | IL1A |  |  |
|  |  | IL10 | IL1B |  |  |
|  |  | IL10 | SELP |  |  |
|  |  | IL10 | IL7 |  |  |
|  |  | IL10 | ICAM1 |  |  |
|  |  | IL10 | KNG1 |  |  |
|  |  | IL10 | ITIH4 |  |  |
|  |  | IL10 | FCGR2A |  |  |
|  |  | IL10 | REN |  |  |
|  |  | IL10 | IFNA1 |  |  |
|  |  | IL10 | IL18 |  |  |
|  |  | IL10 | TNFSF4 |  |  |
|  |  | IL10 | CXCL13 |  |  |
|  |  | IL10 | PPARG |  |  |
|  |  | IL10 | ACE |  |  |
|  |  | IL10 | CXCL16 |  |  |
|  |  | IL10 | VCAM1 |  |  |
|  |  | IL10 | PTX3 |  |  |
|  |  | IL10 | PPBP |  |  |
|  |  | IL10 | PF4 |  |  |
|  |  | IL10 | ITGA2 |  |  |
|  |  | IL10 | TNFRSF11B | |  |
|  |  | IL10 | NOS3 |  |  |
|  |  | IL10 | MMP3 |  |  |
|  |  | IL10 | CCL11 |  |  |
|  |  | IL10 | RNASE3 |  |  |
|  |  | IL10 | IGF1 |  |  |
|  |  | IL10 | IL17D |  |  |
|  |  | IL10 | HSPA4 |  |  |
|  |  | IL10 | IL12A |  |  |
|  |  | IL10 | CD14 |  |  |
|  |  | IL10 | CXCL8 |  |  |
|  |  | IL10 | CYCS |  |  |
|  |  | IL10 | F2 |  |  |
|  |  | IL10 | PLG |  |  |
|  |  | IL10 | OLR1 |  |  |
|  |  | IL10 | LEP |  |  |
|  |  | IL10 | ANGPT2 |  |  |
|  |  | IL10 | MMP1 |  |  |
|  |  | IL10 | CD28 |  |  |
|  |  | IL10 | CSF1 |  |  |
|  |  | IL10 | SELE |  |  |
|  |  | IL10 | CD86 |  |  |
|  |  | IL10 | SMAD3 |  |  |
|  |  | IL10 | F3 |  |  |
|  |  | IL10 | IL17A |  |  |
|  |  | IL10 | FAS |  |  |
|  |  | IL10 | CX3CR1 |  |  |
|  |  | IL10 | CD163 |  |  |
|  |  | IL10 | HLA-DRB1 | |  |
|  |  | IL10 | DPP4 |  |  |
|  |  | IL10 | PARP1 |  |  |
|  |  | IL10 | VIP |  |  |
|  |  | IL10 | PTGS2 |  |  |
|  |  | IL10 | SERPINC1 | |  |
|  |  | IL10 | FASLG |  |  |
|  |  | IL10 | CTGF |  |  |
|  |  | IL10 | S100A8 |  |  |
|  |  | IL10 | S100A9 |  |  |
|  |  | IL10 | CD40LG |  |  |
|  |  | IL10 | ENTPD1 |  |  |
|  |  | IL10 | MMP9 |  |  |
|  |  | IL10 | LCN2 |  |  |
|  |  | IL10 | MBL2 |  |  |
|  |  | IL10 | TLR4 |  |  |
|  |  | IL10 | AGER |  |  |
|  |  | IL10 | TNFRSF1B | |  |
|  |  | IL10 | THBD |  |  |
|  |  | IL10 | EDN1 |  |  |
|  |  | IL10 | IL2RA |  |  |
|  |  | IL10 | TEK |  |  |
|  |  | IL10 | IL33 |  |  |
|  |  | IL10 | HSPD1 |  |  |
|  |  | IL10 | INS |  |  |
|  |  | IL10 | ITGB2 |  |  |
|  |  | IL10 | IL6 |  |  |
|  |  | IL10 | CXCR4 |  |  |
|  |  | IL10 | IL18R1 |  |  |
|  |  | IL10 | IL34 |  |  |
|  |  | IL10 | TNF |  |  |
|  |  | IL10 | MYD88 |  |  |
|  |  | IL10 | FGF21 |  |  |
|  |  | IL10 | SERPINA1 | |  |
|  |  | IL10 | PGF |  |  |
|  |  | IL10 | ANGPT1 |  |  |
|  |  | IL10 | IL32 |  |  |
|  |  | IL10 | HIF1A |  |  |
|  |  | IL10 | PECAM1 |  |  |
|  |  | IL10 | BDNF |  |  |
|  |  | IL10 | VEGFA |  |  |
|  |  | IL10 | CCL5 |  |  |
|  |  | IL10 | CCL3 |  |  |
|  |  | IL12A | IL2RB |  |  |
|  |  | IL12A | EBI3 |  |  |
|  |  | IL12A | CCL2 |  |  |
|  |  | IL12A | IFNG |  |  |
|  |  | IL12A | IL1A |  |  |
|  |  | IL12A | IL1B |  |  |
|  |  | IL12A | IL18 |  |  |
|  |  | IL12A | TLR4 |  |  |
|  |  | IL12A | IL17A |  |  |
|  |  | IL12A | IL2RA |  |  |
|  |  | IL12A | TNF |  |  |
|  |  | IL12A | CCL3 |  |  |
|  |  | IL12A | CCL5 |  |  |
|  |  | IL12A | CXCL8 |  |  |
|  |  | IL12A | IL6 |  |  |
|  |  | IL12A | IL10 |  |  |
|  |  | IL17A | CX3CL1 |  |  |
|  |  | IL17A | TNFRSF1A | |  |
|  |  | IL17A | MIF |  |  |
|  |  | IL17A | HMOX1 |  |  |
|  |  | IL17A | IL2RB |  |  |
|  |  | IL17A | GZMB |  |  |
|  |  | IL17A | TIMP1 |  |  |
|  |  | IL17A | MMP2 |  |  |
|  |  | IL17A | RETN |  |  |
|  |  | IL17A | TGFB1 |  |  |
|  |  | IL17A | HGF |  |  |
|  |  | IL17A | MPO |  |  |
|  |  | IL17A | CCL2 |  |  |
|  |  | IL17A | IL2 |  |  |
|  |  | IL17A | IFNG |  |  |
|  |  | IL17A | IL5 |  |  |
|  |  | IL17A | MMP8 |  |  |
|  |  | IL17A | AHR |  |  |
|  |  | IL17A | LGALS3 |  |  |
|  |  | IL17A | CRP |  |  |
|  |  | IL17A | IL1RN |  |  |
|  |  | IL17A | TLR2 |  |  |
|  |  | IL17A | IL37 |  |  |
|  |  | IL17A | IL1A |  |  |
|  |  | IL17A | IL1B |  |  |
|  |  | IL17A | SELP |  |  |
|  |  | IL17A | IL7 |  |  |
|  |  | IL17A | ICAM1 |  |  |
|  |  | IL17A | FCGR2A |  |  |
|  |  | IL17A | IFNA1 |  |  |
|  |  | IL17A | IL18 |  |  |
|  |  | IL17A | TNFSF4 |  |  |
|  |  | IL17A | CXCL13 |  |  |
|  |  | IL17A | PPARG |  |  |
|  |  | IL17A | VCAM1 |  |  |
|  |  | IL17A | PPBP |  |  |
|  |  | IL17A | ITGA2 |  |  |
|  |  | IL17A | TNFRSF11B | |  |
|  |  | IL17A | MMP3 |  |  |
|  |  | IL17A | CCL11 |  |  |
|  |  | IL17A | RNASE3 |  |  |
|  |  | IL17A | IGF1 |  |  |
|  |  | IL17A | IL12A |  |  |
|  |  | IL17A | CD14 |  |  |
|  |  | IL17A | CXCL8 |  |  |
|  |  | IL17A | LEP |  |  |
|  |  | IL17A | MMP1 |  |  |
|  |  | IL17A | CD28 |  |  |
|  |  | IL17A | CSF1 |  |  |
|  |  | IL17A | SELE |  |  |
|  |  | IL17A | CD86 |  |  |
|  |  | IL17A | SMAD3 |  |  |
|  |  | IL17A | BDNF |  |  |
|  |  | IL17A | IL18R1 |  |  |
|  |  | IL17A | HLA-DRB1 | |  |
|  |  | IL17A | PECAM1 |  |  |
|  |  | IL17A | TNFRSF1B | |  |
|  |  | IL17A | CD163 |  |  |
|  |  | IL17A | IL32 |  |  |
|  |  | IL17A | CX3CR1 |  |  |
|  |  | IL17A | FAS |  |  |
|  |  | IL17A | FASLG |  |  |
|  |  | IL17A | CXCR4 |  |  |
|  |  | IL17A | INS |  |  |
|  |  | IL17A | HIF1A |  |  |
|  |  | IL17A | PTGS2 |  |  |
|  |  | IL17A | ENTPD1 |  |  |
|  |  | IL17A | MYD88 |  |  |
|  |  | IL17A | IL2RA |  |  |
|  |  | IL17A | IL33 |  |  |
|  |  | IL17A | CD40LG |  |  |
|  |  | IL17A | TLR4 |  |  |
|  |  | IL17A | CCL5 |  |  |
|  |  | IL17A | CCL3 |  |  |
|  |  | IL17A | LCN2 |  |  |
|  |  | IL17A | MMP9 |  |  |
|  |  | IL17A | VEGFA |  |  |
|  |  | IL17A | TNF |  |  |
|  |  | IL17A | IL6 |  |  |
|  |  | IL17A | IL10 |  |  |
|  |  | IL17D | IL2RB |  |  |
|  |  | IL17D | IL1B |  |  |
|  |  | IL17D | IL10 |  |  |
|  |  | IL17D | CXCL8 |  |  |
|  |  | IL17D | IL6 |  |  |
|  |  | IL17D | IL2RA |  |  |
|  |  | IL18 | CX3CL1 |  |  |
|  |  | IL18 | TNFRSF1A | |  |
|  |  | IL18 | SIRT1 |  |  |
|  |  | IL18 | MIF |  |  |
|  |  | IL18 | HMOX1 |  |  |
|  |  | IL18 | IL2RB |  |  |
|  |  | IL18 | RIPK3 |  |  |
|  |  | IL18 | GZMB |  |  |
|  |  | IL18 | TIMP1 |  |  |
|  |  | IL18 | MMP2 |  |  |
|  |  | IL18 | RETN |  |  |
|  |  | IL18 | TGFB1 |  |  |
|  |  | IL18 | HGF |  |  |
|  |  | IL18 | SERPINE1 |  |  |
|  |  | IL18 | MPO |  |  |
|  |  | IL18 | CCL2 |  |  |
|  |  | IL18 | NFKB1 |  |  |
|  |  | IL18 | IL2 |  |  |
|  |  | IL18 | IFNG |  |  |
|  |  | IL18 | IL5 |  |  |
|  |  | IL18 | AHR |  |  |
|  |  | IL18 | CRP |  |  |
|  |  | IL18 | IL1RN |  |  |
|  |  | IL18 | RIPK1 |  |  |
|  |  | IL18 | TLR2 |  |  |
|  |  | IL18 | IL37 |  |  |
|  |  | IL18 | IL1A |  |  |
|  |  | IL18 | IL1B |  |  |
|  |  | IL18 | SELP |  |  |
|  |  | IL18 | IL7 |  |  |
|  |  | IL18 | ICAM1 |  |  |
|  |  | IL18 | FCGR2A |  |  |
|  |  | IL18 | IFNA1 |  |  |
|  |  | IL18 | MMP1 |  |  |
|  |  | IL18 | PLG |  |  |
|  |  | IL18 | HIF1A |  |  |
|  |  | IL18 | TNFSF4 |  |  |
|  |  | IL18 | CXCL16 |  |  |
|  |  | IL18 | IGF1 |  |  |
|  |  | IL18 | CYCS |  |  |
|  |  | IL18 | BDNF |  |  |
|  |  | IL18 | EDN1 |  |  |
|  |  | IL18 | PPBP |  |  |
|  |  | IL18 | FAS |  |  |
|  |  | IL18 | AGER |  |  |
|  |  | IL18 | CXCL13 |  |  |
|  |  | IL18 | CXCR4 |  |  |
|  |  | IL18 | MMP3 |  |  |
|  |  | IL18 | TNFRSF1B | |  |
|  |  | IL18 | CD163 |  |  |
|  |  | IL18 | PPARG |  |  |
|  |  | IL18 | MLKL |  |  |
|  |  | IL18 | CX3CR1 |  |  |
|  |  | IL18 | IL2RA |  |  |
|  |  | IL18 | SELE |  |  |
|  |  | IL18 | IL32 |  |  |
|  |  | IL18 | FASLG |  |  |
|  |  | IL18 | INS |  |  |
|  |  | IL18 | PTGS2 |  |  |
|  |  | IL18 | LEP |  |  |
|  |  | IL18 | CD28 |  |  |
|  |  | IL18 | CSF1 |  |  |
|  |  | IL18 | MMP9 |  |  |
|  |  | IL18 | CD40LG |  |  |
|  |  | IL18 | VCAM1 |  |  |
|  |  | IL18 | TXNIP |  |  |
|  |  | IL18 | CD86 |  |  |
|  |  | IL18 | VEGFA |  |  |
|  |  | IL18 | CST3 |  |  |
|  |  | IL18 | CCL11 |  |  |
|  |  | IL18 | LCN2 |  |  |
|  |  | IL18 | TLR4 |  |  |
|  |  | IL18 | IL33 |  |  |
|  |  | IL18 | IL17A |  |  |
|  |  | IL18 | MYD88 |  |  |
|  |  | IL18 | IL12A |  |  |
|  |  | IL18 | CCL5 |  |  |
|  |  | IL18 | CCL3 |  |  |
|  |  | IL18 | CXCL8 |  |  |
|  |  | IL18 | IL6 |  |  |
|  |  | IL18 | IL10 |  |  |
|  |  | IL18 | TNF |  |  |
|  |  | IL18 | IL18R1 |  |  |
|  |  | IL18R1 | IL2RB |  |  |
|  |  | IL18R1 | NFKB1 |  |  |
|  |  | IL18R1 | IL2 |  |  |
|  |  | IL18R1 | IFNG |  |  |
|  |  | IL18R1 | IL5 |  |  |
|  |  | IL18R1 | IL1RN |  |  |
|  |  | IL18R1 | IL37 |  |  |
|  |  | IL18R1 | IL1A |  |  |
|  |  | IL18R1 | IL1B |  |  |
|  |  | IL18R1 | IL18 |  |  |
|  |  | IL18R1 | IL17A |  |  |
|  |  | IL18R1 | IL2RA |  |  |
|  |  | IL18R1 | IL33 |  |  |
|  |  | IL18R1 | IL6 |  |  |
|  |  | IL18R1 | MYD88 |  |  |
|  |  | IL18R1 | TNF |  |  |
|  |  | IL18R1 | IL10 |  |  |
|  |  | IL1A | CX3CL1 |  |  |
|  |  | IL1A | TNFRSF1A | |  |
|  |  | IL1A | MIF |  |  |
|  |  | IL1A | HMOX1 |  |  |
|  |  | IL1A | TIMP1 |  |  |
|  |  | IL1A | MMP2 |  |  |
|  |  | IL1A | RETN |  |  |
|  |  | IL1A | TGFB1 |  |  |
|  |  | IL1A | HGF |  |  |
|  |  | IL1A | SERPINE1 |  |  |
|  |  | IL1A | MPO |  |  |
|  |  | IL1A | CCL2 |  |  |
|  |  | IL1A | NFKB1 |  |  |
|  |  | IL1A | IL2 |  |  |
|  |  | IL1A | IFNG |  |  |
|  |  | IL1A | IL5 |  |  |
|  |  | IL1A | MMP8 |  |  |
|  |  | IL1A | CRP |  |  |
|  |  | IL1A | IL1RN |  |  |
|  |  | IL1A | TLR2 |  |  |
|  |  | IL1A | IL37 |  |  |
|  |  | IL1A | IL18R1 |  |  |
|  |  | IL1A | S100A4 |  |  |
|  |  | IL1A | FCGR2A |  |  |
|  |  | IL1A | PF4 |  |  |
|  |  | IL1A | FASLG |  |  |
|  |  | IL1A | CD28 |  |  |
|  |  | IL1A | HIF1A |  |  |
|  |  | IL1A | PECAM1 |  |  |
|  |  | IL1A | PPARG |  |  |
|  |  | IL1A | F3 |  |  |
|  |  | IL1A | IL32 |  |  |
|  |  | IL1A | BDNF |  |  |
|  |  | IL1A | ADAMTS4 | |  |
|  |  | IL1A | LCN2 |  |  |
|  |  | IL1A | TNFRSF11B | |  |
|  |  | IL1A | CXCR4 |  |  |
|  |  | IL1A | IL2RA |  |  |
|  |  | IL1A | CX3CR1 |  |  |
|  |  | IL1A | SELP |  |  |
|  |  | IL1A | CD163 |  |  |
|  |  | IL1A | EDN1 |  |  |
|  |  | IL1A | PLG |  |  |
|  |  | IL1A | CXCL13 |  |  |
|  |  | IL1A | IL12A |  |  |
|  |  | IL1A | TNFRSF1B | |  |
|  |  | IL1A | INS |  |  |
|  |  | IL1A | IGF1 |  |  |
|  |  | IL1A | LEP |  |  |
|  |  | IL1A | CD86 |  |  |
|  |  | IL1A | SELE |  |  |
|  |  | IL1A | MMP3 |  |  |
|  |  | IL1A | MMP1 |  |  |
|  |  | IL1A | VCAM1 |  |  |
|  |  | IL1A | CD40LG |  |  |
|  |  | IL1A | MMP9 |  |  |
|  |  | IL1A | IFNA1 |  |  |
|  |  | IL1A | IL33 |  |  |
|  |  | IL1A | VEGFA |  |  |
|  |  | IL1A | IL7 |  |  |
|  |  | IL1A | CSF1 |  |  |
|  |  | IL1A | ICAM1 |  |  |
|  |  | IL1A | PTGS2 |  |  |
|  |  | IL1A | CCL11 |  |  |
|  |  | IL1A | IL17A |  |  |
|  |  | IL1A | TLR4 |  |  |
|  |  | IL1A | TNF |  |  |
|  |  | IL1A | MYD88 |  |  |
|  |  | IL1A | IL18 |  |  |
|  |  | IL1A | CCL5 |  |  |
|  |  | IL1A | CCL3 |  |  |
|  |  | IL1A | IL10 |  |  |
|  |  | IL1A | CXCL8 |  |  |
|  |  | IL1A | IL6 |  |  |
|  |  | IL1A | IL1B |  |  |
|  |  | IL1B | CX3CL1 |  |  |
|  |  | IL1B | TNFRSF1A | |  |
|  |  | IL1B | SIRT1 |  |  |
|  |  | IL1B | MIF |  |  |
|  |  | IL1B | HMOX1 |  |  |
|  |  | IL1B | RIPK3 |  |  |
|  |  | IL1B | GZMB |  |  |
|  |  | IL1B | TIMP1 |  |  |
|  |  | IL1B | MMP2 |  |  |
|  |  | IL1B | PLAT |  |  |
|  |  | IL1B | RETN |  |  |
|  |  | IL1B | TGFB1 |  |  |
|  |  | IL1B | HGF |  |  |
|  |  | IL1B | NAMPT |  |  |
|  |  | IL1B | SERPINE1 |  |  |
|  |  | IL1B | MPO |  |  |
|  |  | IL1B | CCL2 |  |  |
|  |  | IL1B | NFKB1 |  |  |
|  |  | IL1B | IL2 |  |  |
|  |  | IL1B | SELPLG |  |  |
|  |  | IL1B | IFNG |  |  |
|  |  | IL1B | IL5 |  |  |
|  |  | IL1B | APOB |  |  |
|  |  | IL1B | MMP8 |  |  |
|  |  | IL1B | APOA1 |  |  |
|  |  | IL1B | AHR |  |  |
|  |  | IL1B | EPO |  |  |
|  |  | IL1B | GDF15 |  |  |
|  |  | IL1B | LGALS3 |  |  |
|  |  | IL1B | CRP |  |  |
|  |  | IL1B | APCS |  |  |
|  |  | IL1B | CHI3L1 |  |  |
|  |  | IL1B | IL1RN |  |  |
|  |  | IL1B | RIPK1 |  |  |
|  |  | IL1B | TLR2 |  |  |
|  |  | IL1B | VWF |  |  |
|  |  | IL1B | CYBA |  |  |
|  |  | IL1B | ITGA2B |  |  |
|  |  | IL1B | IL37 |  |  |
|  |  | IL1B | IL1A |  |  |
|  |  | IL1B | CP |  |  |
|  |  | IL1B | ITGB3 |  |  |
|  |  | IL1B | S100A4 |  |  |
|  |  | IL1B | CD63 |  |  |
|  |  | IL1B | HSPD1 |  |  |
|  |  | IL1B | RNASE3 |  |  |
|  |  | IL1B | IGFBP1 |  |  |
|  |  | IL1B | HLA-DRB1 | |  |
|  |  | IL1B | VIP |  |  |
|  |  | IL1B | PARP1 |  |  |
|  |  | IL1B | SERPINC1 | |  |
|  |  | IL1B | TEK |  |  |
|  |  | IL1B | TNFSF4 |  |  |
|  |  | IL1B | FGF21 |  |  |
|  |  | IL1B | ADM |  |  |
|  |  | IL1B | IL34 |  |  |
|  |  | IL1B | DPP4 |  |  |
|  |  | IL1B | AVP |  |  |
|  |  | IL1B | ANGPT2 |  |  |
|  |  | IL1B | IL17D |  |  |
|  |  | IL1B | MBL2 |  |  |
|  |  | IL1B | CNR1 |  |  |
|  |  | IL1B | ANGPT1 |  |  |
|  |  | IL1B | PGF |  |  |
|  |  | IL1B | IL18R1 |  |  |
|  |  | IL1B | ENTPD1 |  |  |
|  |  | IL1B | PPARGC1A | |  |
|  |  | IL1B | PLA2G2A |  |  |
|  |  | IL1B | OLR1 |  |  |
|  |  | IL1B | AGT |  |  |
|  |  | IL1B | ADRB2 |  |  |
|  |  | IL1B | SERPINA1 | |  |
|  |  | IL1B | ITIH4 |  |  |
|  |  | IL1B | F2 |  |  |
|  |  | IL1B | BGLAP |  |  |
|  |  | IL1B | ITGB2 |  |  |
|  |  | IL1B | SMAD3 |  |  |
|  |  | IL1B | FAS |  |  |
|  |  | IL1B | REN |  |  |
|  |  | IL1B | MLKL |  |  |
|  |  | IL1B | PTX3 |  |  |
|  |  | IL1B | PLAU |  |  |
|  |  | IL1B | AGTR1 |  |  |
|  |  | IL1B | CTGF |  |  |
|  |  | IL1B | ACE |  |  |
|  |  | IL1B | PF4 |  |  |
|  |  | IL1B | IL32 |  |  |
|  |  | IL1B | THBD |  |  |
|  |  | IL1B | S100A9 |  |  |
|  |  | IL1B | CXCL13 |  |  |
|  |  | IL1B | CXCL16 |  |  |
|  |  | IL1B | HSPA4 |  |  |
|  |  | IL1B | TXNIP |  |  |
|  |  | IL1B | FASLG |  |  |
|  |  | IL1B | CYCS |  |  |
|  |  | IL1B | PLG |  |  |
|  |  | IL1B | IL2RA |  |  |
|  |  | IL1B | TNFRSF11B | |  |
|  |  | IL1B | AGER |  |  |
|  |  | IL1B | F3 |  |  |
|  |  | IL1B | CXCR4 |  |  |
|  |  | IL1B | KNG1 |  |  |
|  |  | IL1B | CD14 |  |  |
|  |  | IL1B | TNFRSF1B | |  |
|  |  | IL1B | PECAM1 |  |  |
|  |  | IL1B | ADAMTS4 | |  |
|  |  | IL1B | LCN2 |  |  |
|  |  | IL1B | S100A8 |  |  |
|  |  | IL1B | CD28 |  |  |
|  |  | IL1B | CD40LG |  |  |
|  |  | IL1B | NOS3 |  |  |
|  |  | IL1B | CD163 |  |  |
|  |  | IL1B | EDN1 |  |  |
|  |  | IL1B | CX3CR1 |  |  |
|  |  | IL1B | PPBP |  |  |
|  |  | IL1B | HIF1A |  |  |
|  |  | IL1B | FCGR2A |  |  |
|  |  | IL1B | SELP |  |  |
|  |  | IL1B | BDNF |  |  |
|  |  | IL1B | PPARG |  |  |
|  |  | IL1B | CSF1 |  |  |
|  |  | IL1B | SELE |  |  |
|  |  | IL1B | IGF1 |  |  |
|  |  | IL1B | IL33 |  |  |
|  |  | IL1B | MMP1 |  |  |
|  |  | IL1B | IL7 |  |  |
|  |  | IL1B | IFNA1 |  |  |
|  |  | IL1B | CD86 |  |  |
|  |  | IL1B | INS |  |  |
|  |  | IL1B | MMP3 |  |  |
|  |  | IL1B | CCL11 |  |  |
|  |  | IL1B | VCAM1 |  |  |
|  |  | IL1B | VEGFA |  |  |
|  |  | IL1B | ICAM1 |  |  |
|  |  | IL1B | MMP9 |  |  |
|  |  | IL1B | LEP |  |  |
|  |  | IL1B | TLR4 |  |  |
|  |  | IL1B | IL12A |  |  |
|  |  | IL1B | PTGS2 |  |  |
|  |  | IL1B | IL17A |  |  |
|  |  | IL1B | TNF |  |  |
|  |  | IL1B | CCL5 |  |  |
|  |  | IL1B | MYD88 |  |  |
|  |  | IL1B | IL18 |  |  |
|  |  | IL1B | IL10 |  |  |
|  |  | IL1B | CCL3 |  |  |
|  |  | IL1B | IL6 |  |  |
|  |  | IL1B | CXCL8 |  |  |
|  |  | IL1RN | TNFRSF1A | |  |
|  |  | IL1RN | TIMP1 |  |  |
|  |  | IL1RN | RETN |  |  |
|  |  | IL1RN | TGFB1 |  |  |
|  |  | IL1RN | HGF |  |  |
|  |  | IL1RN | SERPINE1 |  |  |
|  |  | IL1RN | CCL2 |  |  |
|  |  | IL1RN | IL2 |  |  |
|  |  | IL1RN | IFNG |  |  |
|  |  | IL1RN | IL5 |  |  |
|  |  | IL1RN | CRP |  |  |
|  |  | IL1RN | LEP |  |  |
|  |  | IL1RN | IGF1 |  |  |
|  |  | IL1RN | CD40LG |  |  |
|  |  | IL1RN | IFNA1 |  |  |
|  |  | IL1RN | LCN2 |  |  |
|  |  | IL1RN | FCGR2A |  |  |
|  |  | IL1RN | IL2RA |  |  |
|  |  | IL1RN | VCAM1 |  |  |
|  |  | IL1RN | CD163 |  |  |
|  |  | IL1RN | CSF1 |  |  |
|  |  | IL1RN | IL7 |  |  |
|  |  | IL1RN | CD14 |  |  |
|  |  | IL1RN | MMP1 |  |  |
|  |  | IL1RN | IL18R1 |  |  |
|  |  | IL1RN | INS |  |  |
|  |  | IL1RN | S100A9 |  |  |
|  |  | IL1RN | CD86 |  |  |
|  |  | IL1RN | CCL11 |  |  |
|  |  | IL1RN | MMP3 |  |  |
|  |  | IL1RN | VEGFA |  |  |
|  |  | IL1RN | SERPINA1 | |  |
|  |  | IL1RN | ICAM1 |  |  |
|  |  | IL1RN | TLR4 |  |  |
|  |  | IL1RN | TLR2 |  |  |
|  |  | IL1RN | PTGS2 |  |  |
|  |  | IL1RN | IL17A |  |  |
|  |  | IL1RN | MMP9 |  |  |
|  |  | IL1RN | CCL5 |  |  |
|  |  | IL1RN | S100A8 |  |  |
|  |  | IL1RN | IL18 |  |  |
|  |  | IL1RN | CCL3 |  |  |
|  |  | IL1RN | TNF |  |  |
|  |  | IL1RN | CXCL8 |  |  |
|  |  | IL1RN | IL6 |  |  |
|  |  | IL1RN | MYD88 |  |  |
|  |  | IL1RN | TNFRSF1B | |  |
|  |  | IL1RN | IL1B |  |  |
|  |  | IL1RN | IL10 |  |  |
|  |  | IL1RN | IL1A |  |  |
|  |  | IL2 | CX3CL1 |  |  |
|  |  | IL2 | TNFRSF1A | |  |
|  |  | IL2 | MIF |  |  |
|  |  | IL2 | HMOX1 |  |  |
|  |  | IL2 | IL2RB |  |  |
|  |  | IL2 | GZMB |  |  |
|  |  | IL2 | TIMP1 |  |  |
|  |  | IL2 | MMP2 |  |  |
|  |  | IL2 | TGFB1 |  |  |
|  |  | IL2 | HGF |  |  |
|  |  | IL2 | MPO |  |  |
|  |  | IL2 | CCL2 |  |  |
|  |  | IL2 | NFKB1 |  |  |
|  |  | IL2 | RNASE3 |  |  |
|  |  | IL2 | F3 |  |  |
|  |  | IL2 | MMP3 |  |  |
|  |  | IL2 | ITGB2 |  |  |
|  |  | IL2 | IL37 |  |  |
|  |  | IL2 | PARP1 |  |  |
|  |  | IL2 | IL18R1 |  |  |
|  |  | IL2 | CD163 |  |  |
|  |  | IL2 | IL32 |  |  |
|  |  | IL2 | PPBP |  |  |
|  |  | IL2 | PPARG |  |  |
|  |  | IL2 | SMAD3 |  |  |
|  |  | IL2 | DPP4 |  |  |
|  |  | IL2 | BDNF |  |  |
|  |  | IL2 | CYCS |  |  |
|  |  | IL2 | HLA-DRB1 | |  |
|  |  | IL2 | CX3CR1 |  |  |
|  |  | IL2 | AHR |  |  |
|  |  | IL2 | IGF1 |  |  |
|  |  | IL2 | PECAM1 |  |  |
|  |  | IL2 | HIF1A |  |  |
|  |  | IL2 | SELP |  |  |
|  |  | IL2 | TYR |  |  |
|  |  | IL2 | TNFRSF1B | |  |
|  |  | IL2 | SELE |  |  |
|  |  | IL2 | ITGA2 |  |  |
|  |  | IL2 | HSPA4 |  |  |
|  |  | IL2 | EPO |  |  |
|  |  | IL2 | CXCL13 |  |  |
|  |  | IL2 | MMP9 |  |  |
|  |  | IL2 | IL1RN |  |  |
|  |  | IL2 | LEP |  |  |
|  |  | IL2 | PTGS2 |  |  |
|  |  | IL2 | MYD88 |  |  |
|  |  | IL2 | VCAM1 |  |  |
|  |  | IL2 | ENTPD1 |  |  |
|  |  | IL2 | CSF1 |  |  |
|  |  | IL2 | INS |  |  |
|  |  | IL2 | CXCR4 |  |  |
|  |  | IL2 | FCGR2A |  |  |
|  |  | IL2 | ITIH4 |  |  |
|  |  | IL2 | TLR4 |  |  |
|  |  | IL2 | FAS |  |  |
|  |  | IL2 | CRP |  |  |
|  |  | IL2 | FASLG |  |  |
|  |  | IL2 | TNFSF4 |  |  |
|  |  | IL2 | ICAM1 |  |  |
|  |  | IL2 | TLR2 |  |  |
|  |  | IL2 | VEGFA |  |  |
|  |  | IL2 | CD40LG |  |  |
|  |  | IL2 | CCL11 |  |  |
|  |  | IL2 | CD86 |  |  |
|  |  | IL2 | CCL5 |  |  |
|  |  | IL2 | CCL3 |  |  |
|  |  | IL2 | IL1A |  |  |
|  |  | IL2 | IL33 |  |  |
|  |  | IL2 | CXCL8 |  |  |
|  |  | IL2 | IL1B |  |  |
|  |  | IL2 | IL7 |  |  |
|  |  | IL2 | IFNG |  |  |
|  |  | IL2 | CD28 |  |  |
|  |  | IL2 | IL6 |  |  |
|  |  | IL2 | IL10 |  |  |
|  |  | IL2 | TNF |  |  |
|  |  | IL2 | IL17A |  |  |
|  |  | IL2 | IFNA1 |  |  |
|  |  | IL2 | IL5 |  |  |
|  |  | IL2 | IL18 |  |  |
|  |  | IL2 | IL2RA |  |  |
|  |  | IL2RA | TNFRSF1A | |  |
|  |  | IL2RA | IL2RB |  |  |
|  |  | IL2RA | GZMB |  |  |
|  |  | IL2RA | TGFB1 |  |  |
|  |  | IL2RA | CCL2 |  |  |
|  |  | IL2RA | NFKB1 |  |  |
|  |  | IL2RA | IL2 |  |  |
|  |  | IL2RA | IFNG |  |  |
|  |  | IL2RA | IL5 |  |  |
|  |  | IL2RA | EPO |  |  |
|  |  | IL2RA | IL1RN |  |  |
|  |  | IL2RA | TLR2 |  |  |
|  |  | IL2RA | IL1A |  |  |
|  |  | IL2RA | IL1B |  |  |
|  |  | IL2RA | IL7 |  |  |
|  |  | IL2RA | ICAM1 |  |  |
|  |  | IL2RA | FCGR2A |  |  |
|  |  | IL2RA | IFNA1 |  |  |
|  |  | IL2RA | IL18 |  |  |
|  |  | IL2RA | TNFSF4 |  |  |
|  |  | IL2RA | ITGA2 |  |  |
|  |  | IL2RA | CCL11 |  |  |
|  |  | IL2RA | IL17D |  |  |
|  |  | IL2RA | IL12A |  |  |
|  |  | IL2RA | CD14 |  |  |
|  |  | IL2RA | CXCL8 |  |  |
|  |  | IL2RA | LEP |  |  |
|  |  | IL2RA | CD28 |  |  |
|  |  | IL2RA | CD86 |  |  |
|  |  | IL2RA | SMAD3 |  |  |
|  |  | IL2RA | IL17A |  |  |
|  |  | IL2RA | FAS |  |  |
|  |  | IL2RA | HLA-DRB1 | |  |
|  |  | IL2RA | FASLG |  |  |
|  |  | IL2RA | CD40LG |  |  |
|  |  | IL2RA | ENTPD1 |  |  |
|  |  | IL2RA | TLR4 |  |  |
|  |  | IL2RA | TNFRSF1B | |  |
|  |  | IL2RA | VEGFA |  |  |
|  |  | IL2RA | INS |  |  |
|  |  | IL2RA | IL33 |  |  |
|  |  | IL2RA | IL18R1 |  |  |
|  |  | IL2RA | CCL5 |  |  |
|  |  | IL2RA | CCL3 |  |  |
|  |  | IL2RA | TNF |  |  |
|  |  | IL2RA | IL6 |  |  |
|  |  | IL2RA | IL10 |  |  |
|  |  | IL2RB | CCL2 |  |  |
|  |  | IL2RB | HLA-DRB1 | |  |
|  |  | IL2RB | FCGR2A |  |  |
|  |  | IL2RB | TNFRSF1B | |  |
|  |  | IL2RB | IL18 |  |  |
|  |  | IL2RB | CCL5 |  |  |
|  |  | IL2RB | FAS |  |  |
|  |  | IL2RB | FASLG |  |  |
|  |  | IL2RB | ITGB2 |  |  |
|  |  | IL2RB | CD86 |  |  |
|  |  | IL2RB | IL17A |  |  |
|  |  | IL2RB | ITGA2 |  |  |
|  |  | IL2RB | TNF |  |  |
|  |  | IL2RB | VCAM1 |  |  |
|  |  | IL2RB | IL18R1 |  |  |
|  |  | IL2RB | LEP |  |  |
|  |  | IL2RB | IL17D |  |  |
|  |  | IL2RB | IL12A |  |  |
|  |  | IL2RB | EPO |  |  |
|  |  | IL2RB | CD28 |  |  |
|  |  | IL2RB | IFNA1 |  |  |
|  |  | IL2RB | CD40LG |  |  |
|  |  | IL2RB | IL5 |  |  |
|  |  | IL2RB | IL6 |  |  |
|  |  | IL2RB | GZMB |  |  |
|  |  | IL2RB | IL10 |  |  |
|  |  | IL2RB | IFNG |  |  |
|  |  | IL2RB | IL7 |  |  |
|  |  | IL2RB | IL2 |  |  |
|  |  | IL2RB | IL2RA |  |  |
|  |  | IL32 | HGF |  |  |
|  |  | IL32 | CCL2 |  |  |
|  |  | IL32 | IL2 |  |  |
|  |  | IL32 | IL1A |  |  |
|  |  | IL32 | IL1B |  |  |
|  |  | IL32 | IL18 |  |  |
|  |  | IL32 | CXCL8 |  |  |
|  |  | IL32 | IL17A |  |  |
|  |  | IL32 | IL33 |  |  |
|  |  | IL32 | IL6 |  |  |
|  |  | IL32 | IL34 |  |  |
|  |  | IL32 | TNF |  |  |
|  |  | IL32 | IL10 |  |  |
|  |  | IL32 | VEGFA |  |  |
|  |  | IL32 | CCL5 |  |  |
|  |  | IL33 | MPO |  |  |
|  |  | IL33 | CCL2 |  |  |
|  |  | IL33 | IL2 |  |  |
|  |  | IL33 | IFNG |  |  |
|  |  | IL33 | IL5 |  |  |
|  |  | IL33 | AHR |  |  |
|  |  | IL33 | CRP |  |  |
|  |  | IL33 | TLR2 |  |  |
|  |  | IL33 | IL37 |  |  |
|  |  | IL33 | IL1A |  |  |
|  |  | IL33 | IL1B |  |  |
|  |  | IL33 | IL7 |  |  |
|  |  | IL33 | ICAM1 |  |  |
|  |  | IL33 | FCGR2A |  |  |
|  |  | IL33 | IFNA1 |  |  |
|  |  | IL33 | IL18 |  |  |
|  |  | IL33 | TNFSF4 |  |  |
|  |  | IL33 | CXCL13 |  |  |
|  |  | IL33 | VCAM1 |  |  |
|  |  | IL33 | CCL11 |  |  |
|  |  | IL33 | RNASE3 |  |  |
|  |  | IL33 | CXCL8 |  |  |
|  |  | IL33 | CD28 |  |  |
|  |  | IL33 | CSF1 |  |  |
|  |  | IL33 | CD86 |  |  |
|  |  | IL33 | SMAD3 |  |  |
|  |  | IL33 | IL17A |  |  |
|  |  | IL33 | CD40LG |  |  |
|  |  | IL33 | MMP9 |  |  |
|  |  | IL33 | TLR4 |  |  |
|  |  | IL33 | IL2RA |  |  |
|  |  | IL33 | IL32 |  |  |
|  |  | IL33 | IL34 |  |  |
|  |  | IL33 | VEGFA |  |  |
|  |  | IL33 | CCL3 |  |  |
|  |  | IL33 | CCL5 |  |  |
|  |  | IL33 | IL10 |  |  |
|  |  | IL33 | TNF |  |  |
|  |  | IL33 | IL6 |  |  |
|  |  | IL33 | IL18R1 |  |  |
|  |  | IL33 | MYD88 |  |  |
|  |  | IL34 | CX3CL1 |  |  |
|  |  | IL34 | CCL2 |  |  |
|  |  | IL34 | IL1B |  |  |
|  |  | IL34 | CSF1 |  |  |
|  |  | IL34 | CX3CR1 |  |  |
|  |  | IL34 | CD163 |  |  |
|  |  | IL34 | IL33 |  |  |
|  |  | IL34 | IL6 |  |  |
|  |  | IL34 | TNF |  |  |
|  |  | IL34 | IL32 |  |  |
|  |  | IL34 | IL10 |  |  |
|  |  | IL37 | CCL2 |  |  |
|  |  | IL37 | IL2 |  |  |
|  |  | IL37 | IFNG |  |  |
|  |  | IL37 | CRP |  |  |
|  |  | IL37 | TLR2 |  |  |
|  |  | IL37 | MMP9 |  |  |
|  |  | IL37 | MMP1 |  |  |
|  |  | IL37 | VCAM1 |  |  |
|  |  | IL37 | ADAMTS4 | |  |
|  |  | IL37 | TLR4 |  |  |
|  |  | IL37 | PTGS2 |  |  |
|  |  | IL37 | MMP3 |  |  |
|  |  | IL37 | MYD88 |  |  |
|  |  | IL37 | IL17A |  |  |
|  |  | IL37 | CXCL8 |  |  |
|  |  | IL37 | IL10 |  |  |
|  |  | IL37 | IL1A |  |  |
|  |  | IL37 | IL33 |  |  |
|  |  | IL37 | IL1B |  |  |
|  |  | IL37 | TNF |  |  |
|  |  | IL37 | IL6 |  |  |
|  |  | IL37 | IL18 |  |  |
|  |  | IL37 | IL18R1 |  |  |
|  |  | IL37 | SMAD3 |  |  |
|  |  | IL5 | CX3CL1 |  |  |
|  |  | IL5 | TNFRSF1A | |  |
|  |  | IL5 | IL2RB |  |  |
|  |  | IL5 | GZMB |  |  |
|  |  | IL5 | TIMP1 |  |  |
|  |  | IL5 | RETN |  |  |
|  |  | IL5 | TGFB1 |  |  |
|  |  | IL5 | MPO |  |  |
|  |  | IL5 | CCL2 |  |  |
|  |  | IL5 | IL2 |  |  |
|  |  | IL5 | IFNG |  |  |
|  |  | IL5 | IL18R1 |  |  |
|  |  | IL5 | FAS |  |  |
|  |  | IL5 | AHR |  |  |
|  |  | IL5 | CXCR4 |  |  |
|  |  | IL5 | EPO |  |  |
|  |  | IL5 | INS |  |  |
|  |  | IL5 | IL1RN |  |  |
|  |  | IL5 | FASLG |  |  |
|  |  | IL5 | SELE |  |  |
|  |  | IL5 | PTGS2 |  |  |
|  |  | IL5 | PPBP |  |  |
|  |  | IL5 | PRG2 |  |  |
|  |  | IL5 | LEP |  |  |
|  |  | IL5 | CXCL13 |  |  |
|  |  | IL5 | FCGR2A |  |  |
|  |  | IL5 | MMP9 |  |  |
|  |  | IL5 | MYD88 |  |  |
|  |  | IL5 | CRP |  |  |
|  |  | IL5 | VCAM1 |  |  |
|  |  | IL5 | TNFSF4 |  |  |
|  |  | IL5 | CSF1 |  |  |
|  |  | IL5 | YWHAZ |  |  |
|  |  | IL5 | TLR2 |  |  |
|  |  | IL5 | ICAM1 |  |  |
|  |  | IL5 | TLR4 |  |  |
|  |  | IL5 | CD40LG |  |  |
|  |  | IL5 | CD86 |  |  |
|  |  | IL5 | IFNA1 |  |  |
|  |  | IL5 | VEGFA |  |  |
|  |  | IL5 | CD28 |  |  |
|  |  | IL5 | IL18 |  |  |
|  |  | IL5 | RNASE3 |  |  |
|  |  | IL5 | IL1A |  |  |
|  |  | IL5 | CCL3 |  |  |
|  |  | IL5 | IL7 |  |  |
|  |  | IL5 | IL1B |  |  |
|  |  | IL5 | CXCL8 |  |  |
|  |  | IL5 | IL2RA |  |  |
|  |  | IL5 | IL33 |  |  |
|  |  | IL5 | CCL5 |  |  |
|  |  | IL5 | IL6 |  |  |
|  |  | IL5 | IL17A |  |  |
|  |  | IL5 | TNF |  |  |
|  |  | IL5 | IL10 |  |  |
|  |  | IL5 | CCL11 |  |  |
|  |  | IL6 | CX3CL1 |  |  |
|  |  | IL6 | TNFRSF1A | |  |
|  |  | IL6 | CETP |  |  |
|  |  | IL6 | SIRT1 |  |  |
|  |  | IL6 | MIF |  |  |
|  |  | IL6 | HMOX1 |  |  |
|  |  | IL6 | IL2RB |  |  |
|  |  | IL6 | RIPK3 |  |  |
|  |  | IL6 | GZMB |  |  |
|  |  | IL6 | TIMP1 |  |  |
|  |  | IL6 | MMP2 |  |  |
|  |  | IL6 | PLAT |  |  |
|  |  | IL6 | RETN |  |  |
|  |  | IL6 | TGFB1 |  |  |
|  |  | IL6 | PON1 |  |  |
|  |  | IL6 | HGF |  |  |
|  |  | IL6 | NAMPT |  |  |
|  |  | IL6 | SERPINE1 |  |  |
|  |  | IL6 | MPO |  |  |
|  |  | IL6 | CCL2 |  |  |
|  |  | IL6 | NFKB1 |  |  |
|  |  | IL6 | IL2 |  |  |
|  |  | IL6 | IFNG |  |  |
|  |  | IL6 | IL5 |  |  |
|  |  | IL6 | HRG |  |  |
|  |  | IL6 | APOB |  |  |
|  |  | IL6 | MMP8 |  |  |
|  |  | IL6 | APOA1 |  |  |
|  |  | IL6 | AHR |  |  |
|  |  | IL6 | EPO |  |  |
|  |  | IL6 | GDF15 |  |  |
|  |  | IL6 | LGALS3 |  |  |
|  |  | IL6 | CRP |  |  |
|  |  | IL6 | APCS |  |  |
|  |  | IL6 | CHI3L1 |  |  |
|  |  | IL6 | FST |  |  |
|  |  | IL6 | IL1RN |  |  |
|  |  | IL6 | RIPK1 |  |  |
|  |  | IL6 | TLR2 |  |  |
|  |  | IL6 | VWF |  |  |
|  |  | IL6 | CYBA |  |  |
|  |  | IL6 | ITGA2B |  |  |
|  |  | IL6 | IL37 |  |  |
|  |  | IL6 | IL1A |  |  |
|  |  | IL6 | IL1B |  |  |
|  |  | IL6 | SELP |  |  |
|  |  | IL6 | IL7 |  |  |
|  |  | IL6 | CP |  |  |
|  |  | IL6 | ICAM1 |  |  |
|  |  | IL6 | PPARGC1A | |  |
|  |  | IL6 | KNG1 |  |  |
|  |  | IL6 | ITIH4 |  |  |
|  |  | IL6 | FCGR2A |  |  |
|  |  | IL6 | REN |  |  |
|  |  | IL6 | IGFBP1 |  |  |
|  |  | IL6 | IFNA1 |  |  |
|  |  | IL6 | IL18 |  |  |
|  |  | IL6 | TNFSF4 |  |  |
|  |  | IL6 | BMP6 |  |  |
|  |  | IL6 | CXCL13 |  |  |
|  |  | IL6 | PPARG |  |  |
|  |  | IL6 | HMGCR |  |  |
|  |  | IL6 | ACE |  |  |
|  |  | IL6 | CXCL16 |  |  |
|  |  | IL6 | VCAM1 |  |  |
|  |  | IL6 | PTX3 |  |  |
|  |  | IL6 | PPBP |  |  |
|  |  | IL6 | PF4 |  |  |
|  |  | IL6 | ITGA2 |  |  |
|  |  | IL6 | TNFRSF11B | |  |
|  |  | IL6 | NOS3 |  |  |
|  |  | IL6 | MMP3 |  |  |
|  |  | IL6 | CCL11 |  |  |
|  |  | IL6 | RNASE3 |  |  |
|  |  | IL6 | IGF1 |  |  |
|  |  | IL6 | IL17D |  |  |
|  |  | IL6 | HSPA4 |  |  |
|  |  | IL6 | PCSK9 |  |  |
|  |  | IL6 | IL12A |  |  |
|  |  | IL6 | CD14 |  |  |
|  |  | IL6 | ADRB2 |  |  |
|  |  | IL6 | FGA |  |  |
|  |  | IL6 | CXCL8 |  |  |
|  |  | IL6 | CYCS |  |  |
|  |  | IL6 | F2 |  |  |
|  |  | IL6 | PLG |  |  |
|  |  | IL6 | OLR1 |  |  |
|  |  | IL6 | LEP |  |  |
|  |  | IL6 | ANGPT2 |  |  |
|  |  | IL6 | MMP1 |  |  |
|  |  | IL6 | CD28 |  |  |
|  |  | IL6 | CSF1 |  |  |
|  |  | IL6 | SELE |  |  |
|  |  | IL6 | GAS6 |  |  |
|  |  | IL6 | CD86 |  |  |
|  |  | IL6 | SMAD3 |  |  |
|  |  | IL6 | F3 |  |  |
|  |  | IL6 | TNNI3 |  |  |
|  |  | IL6 | SERPINA12 | |  |
|  |  | IL6 | IL17A |  |  |
|  |  | IL6 | FAS |  |  |
|  |  | IL6 | CX3CR1 |  |  |
|  |  | IL6 | CD163 |  |  |
|  |  | IL6 | HLA-DRB1 | |  |
|  |  | IL6 | DPP4 |  |  |
|  |  | IL6 | AGT |  |  |
|  |  | IL6 | PARP1 |  |  |
|  |  | IL6 | CHIT1 |  |  |
|  |  | IL6 | VIP |  |  |
|  |  | IL6 | PTGS2 |  |  |
|  |  | IL6 | SERPINC1 | |  |
|  |  | IL6 | FASLG |  |  |
|  |  | IL6 | CTGF |  |  |
|  |  | IL6 | ADAMTS4 | |  |
|  |  | IL6 | BGLAP |  |  |
|  |  | IL6 | S100A4 |  |  |
|  |  | IL6 | S100A8 |  |  |
|  |  | IL6 | S100A9 |  |  |
|  |  | IL6 | CNR1 |  |  |
|  |  | IL6 | CD40LG |  |  |
|  |  | IL6 | ENTPD1 |  |  |
|  |  | IL6 | MMP9 |  |  |
|  |  | IL6 | PLAU |  |  |
|  |  | IL6 | LCN2 |  |  |
|  |  | IL6 | MBL2 |  |  |
|  |  | IL6 | TLR4 |  |  |
|  |  | IL6 | AGER |  |  |
|  |  | IL6 | F10 |  |  |
|  |  | IL6 | F7 |  |  |
|  |  | IL6 | TNFRSF1B | |  |
|  |  | IL6 | NPPB |  |  |
|  |  | IL6 | THBD |  |  |
|  |  | IL6 | EDN1 |  |  |
|  |  | IL6 | IL2RA |  |  |
|  |  | IL6 | TEK |  |  |
|  |  | IL6 | AVP |  |  |
|  |  | IL6 | IL33 |  |  |
|  |  | IL6 | HSPD1 |  |  |
|  |  | IL6 | INS |  |  |
|  |  | IL6 | MB |  |  |
|  |  | IL6 | ITGB2 |  |  |
|  |  | IL6 | CST3 |  |  |
|  |  | IL6 | PLA2G2A |  |  |
|  |  | IL6 | TXNIP |  |  |
|  |  | IL6 | TNFRSF11A | |  |
|  |  | IL6 | IL18R1 |  |  |
|  |  | IL6 | CD63 |  |  |
|  |  | IL6 | IL34 |  |  |
|  |  | IL6 | ITGB3 |  |  |
|  |  | IL6 | ADM |  |  |
|  |  | IL6 | ANGPT1 |  |  |
|  |  | IL6 | AGTR1 |  |  |
|  |  | IL6 | IL32 |  |  |
|  |  | IL6 | PGF |  |  |
|  |  | IL6 | FGF21 |  |  |
|  |  | IL6 | SERPINA1 | |  |
|  |  | IL6 | PECAM1 |  |  |
|  |  | IL6 | CXCR4 |  |  |
|  |  | IL6 | HIF1A |  |  |
|  |  | IL6 | BDNF |  |  |
|  |  | IL6 | MYD88 |  |  |
|  |  | IL6 | IL10 |  |  |
|  |  | IL6 | VEGFA |  |  |
|  |  | IL6 | CCL5 |  |  |
|  |  | IL6 | CCL3 |  |  |
|  |  | IL6 | TNF |  |  |
|  |  | IL7 | CX3CL1 |  |  |
|  |  | IL7 | TNFRSF1A | |  |
|  |  | IL7 | IL2RB |  |  |
|  |  | IL7 | GZMB |  |  |
|  |  | IL7 | TGFB1 |  |  |
|  |  | IL7 | HGF |  |  |
|  |  | IL7 | NAMPT |  |  |
|  |  | IL7 | CCL2 |  |  |
|  |  | IL7 | IL2 |  |  |
|  |  | IL7 | IFNG |  |  |
|  |  | IL7 | IL5 |  |  |
|  |  | IL7 | EPO |  |  |
|  |  | IL7 | CRP |  |  |
|  |  | IL7 | IL1RN |  |  |
|  |  | IL7 | TLR2 |  |  |
|  |  | IL7 | IL1A |  |  |
|  |  | IL7 | IL1B |  |  |
|  |  | IL7 | PPBP |  |  |
|  |  | IL7 | FCGR2A |  |  |
|  |  | IL7 | INS |  |  |
|  |  | IL7 | RPS27A |  |  |
|  |  | IL7 | TNFRSF11B | |  |
|  |  | IL7 | PECAM1 |  |  |
|  |  | IL7 | FASLG |  |  |
|  |  | IL7 | TLR4 |  |  |
|  |  | IL7 | TNFSF4 |  |  |
|  |  | IL7 | FAS |  |  |
|  |  | IL7 | VCAM1 |  |  |
|  |  | IL7 | CXCR4 |  |  |
|  |  | IL7 | CXCL13 |  |  |
|  |  | IL7 | ICAM1 |  |  |
|  |  | IL7 | CD86 |  |  |
|  |  | IL7 | CSF1 |  |  |
|  |  | IL7 | IL33 |  |  |
|  |  | IL7 | CD40LG |  |  |
|  |  | IL7 | IFNA1 |  |  |
|  |  | IL7 | VEGFA |  |  |
|  |  | IL7 | IL18 |  |  |
|  |  | IL7 | CD28 |  |  |
|  |  | IL7 | CCL5 |  |  |
|  |  | IL7 | CCL3 |  |  |
|  |  | IL7 | CXCL8 |  |  |
|  |  | IL7 | IGF1 |  |  |
|  |  | IL7 | TNF |  |  |
|  |  | IL7 | CCL11 |  |  |
|  |  | IL7 | IL6 |  |  |
|  |  | IL7 | IL10 |  |  |
|  |  | IL7 | IL17A |  |  |
|  |  | IL7 | IL2RA |  |  |
|  |  | INS | TNFRSF1A | |  |
|  |  | INS | CETP |  |  |
|  |  | INS | SIRT1 |  |  |
|  |  | INS | MIF |  |  |
|  |  | INS | HMOX1 |  |  |
|  |  | INS | CHGA |  |  |
|  |  | INS | TIMP1 |  |  |
|  |  | INS | MMP2 |  |  |
|  |  | INS | PLAT |  |  |
|  |  | INS | RETN |  |  |
|  |  | INS | TGFB1 |  |  |
|  |  | INS | PON1 |  |  |
|  |  | INS | HGF |  |  |
|  |  | INS | NAMPT |  |  |
|  |  | INS | SERPINE1 |  |  |
|  |  | INS | MPO |  |  |
|  |  | INS | CCL2 |  |  |
|  |  | INS | IL2 |  |  |
|  |  | INS | IFNG |  |  |
|  |  | INS | IL5 |  |  |
|  |  | INS | APOB |  |  |
|  |  | INS | APOA1 |  |  |
|  |  | INS | TNNT2 |  |  |
|  |  | INS | AHR |  |  |
|  |  | INS | EPO |  |  |
|  |  | INS | LGALS3 |  |  |
|  |  | INS | CRP |  |  |
|  |  | INS | FST |  |  |
|  |  | INS | IL1RN |  |  |
|  |  | INS | TLR2 |  |  |
|  |  | INS | VWF |  |  |
|  |  | INS | CYBA |  |  |
|  |  | INS | IL1A |  |  |
|  |  | INS | IL1B |  |  |
|  |  | INS | SELP |  |  |
|  |  | INS | IL7 |  |  |
|  |  | INS | CP |  |  |
|  |  | INS | ICAM1 |  |  |
|  |  | INS | PPARGC1A | |  |
|  |  | INS | KNG1 |  |  |
|  |  | INS | PIK3C2A |  |  |
|  |  | INS | REN |  |  |
|  |  | INS | IGFBP1 |  |  |
|  |  | INS | IFNA1 |  |  |
|  |  | INS | IL18 |  |  |
|  |  | INS | BMP6 |  |  |
|  |  | INS | PPARG |  |  |
|  |  | INS | HMGCR |  |  |
|  |  | INS | ACE |  |  |
|  |  | INS | VCAM1 |  |  |
|  |  | INS | TNFRSF11B | |  |
|  |  | INS | NOS3 |  |  |
|  |  | INS | MMP3 |  |  |
|  |  | INS | IGF1 |  |  |
|  |  | INS | HSPA4 |  |  |
|  |  | INS | PCSK9 |  |  |
|  |  | INS | ADRB2 |  |  |
|  |  | INS | FGA |  |  |
|  |  | INS | CXCL8 |  |  |
|  |  | INS | CYCS |  |  |
|  |  | INS | F2 |  |  |
|  |  | INS | PLG |  |  |
|  |  | INS | LEP |  |  |
|  |  | INS | MMP1 |  |  |
|  |  | INS | CD28 |  |  |
|  |  | INS | CSF1 |  |  |
|  |  | INS | PAPPA |  |  |
|  |  | INS | SELE |  |  |
|  |  | INS | CD86 |  |  |
|  |  | INS | SMAD3 |  |  |
|  |  | INS | F3 |  |  |
|  |  | INS | TNNI3 |  |  |
|  |  | INS | SERPINA12 | |  |
|  |  | INS | IL17A |  |  |
|  |  | INS | HLA-DRB1 | |  |
|  |  | INS | DPP4 |  |  |
|  |  | INS | AGT |  |  |
|  |  | INS | COG2 |  |  |
|  |  | INS | PARP1 |  |  |
|  |  | INS | VIP |  |  |
|  |  | INS | PTGS2 |  |  |
|  |  | INS | SERPINC1 | |  |
|  |  | INS | FASLG |  |  |
|  |  | INS | CTGF |  |  |
|  |  | INS | BGLAP |  |  |
|  |  | INS | CNR1 |  |  |
|  |  | INS | CD40LG |  |  |
|  |  | INS | MMP9 |  |  |
|  |  | INS | LCN2 |  |  |
|  |  | INS | FABP3 |  |  |
|  |  | INS | TLR4 |  |  |
|  |  | INS | AGER |  |  |
|  |  | INS | F7 |  |  |
|  |  | INS | NPPB |  |  |
|  |  | INS | EDN1 |  |  |
|  |  | INS | IL2RA |  |  |
|  |  | INS | TEK |  |  |
|  |  | INS | AVP |  |  |
|  |  | INS | HSPD1 |  |  |
|  |  | INS | ANGPT1 |  |  |
|  |  | INS | CCL3 |  |  |
|  |  | INS | ENHO |  |  |
|  |  | INS | MYD88 |  |  |
|  |  | INS | ADM |  |  |
|  |  | INS | BRCA1 |  |  |
|  |  | INS | SERPINA1 | |  |
|  |  | INS | CCL5 |  |  |
|  |  | INS | CXCR4 |  |  |
|  |  | INS | TXNIP |  |  |
|  |  | INS | AGTR1 |  |  |
|  |  | INS | MB |  |  |
|  |  | INS | HIF1A |  |  |
|  |  | INS | PECAM1 |  |  |
|  |  | INS | BDNF |  |  |
|  |  | INS | IL10 |  |  |
|  |  | INS | VEGFA |  |  |
|  |  | INS | CST3 |  |  |
|  |  | INS | FGF21 |  |  |
|  |  | INS | TNF |  |  |
|  |  | INS | IL6 |  |  |
|  |  | ITGA2 | IL2RB |  |  |
|  |  | ITGA2 | GZMB |  |  |
|  |  | ITGA2 | HGF |  |  |
|  |  | ITGA2 | SERPINE1 |  |  |
|  |  | ITGA2 | IL2 |  |  |
|  |  | ITGA2 | IFNG |  |  |
|  |  | ITGA2 | VWF |  |  |
|  |  | ITGA2 | ITGA2B |  |  |
|  |  | ITGA2 | SELP |  |  |
|  |  | ITGA2 | ICAM1 |  |  |
|  |  | ITGA2 | TNC |  |  |
|  |  | ITGA2 | FCGR2A |  |  |
|  |  | ITGA2 | VCAM1 |  |  |
|  |  | ITGA2 | MMP9 |  |  |
|  |  | ITGA2 | BSG |  |  |
|  |  | ITGA2 | FABP3 |  |  |
|  |  | ITGA2 | IL17A |  |  |
|  |  | ITGA2 | IL6 |  |  |
|  |  | ITGA2 | CD86 |  |  |
|  |  | ITGA2 | IL2RA |  |  |
|  |  | ITGA2 | TNF |  |  |
|  |  | ITGA2 | IL10 |  |  |
|  |  | ITGA2 | PECAM1 |  |  |
|  |  | ITGA2 | MMP1 |  |  |
|  |  | ITGA2 | GP1BA |  |  |
|  |  | ITGA2 | THBS4 |  |  |
|  |  | ITGA2 | COL6A2 |  |  |
|  |  | ITGA2 | CD28 |  |  |
|  |  | ITGA2 | ITGB2 |  |  |
|  |  | ITGA2 | ITGB3 |  |  |
|  |  | ITGA2B | PLAT |  |  |
|  |  | ITGA2B | SERPINE1 |  |  |
|  |  | ITGA2B | MPO |  |  |
|  |  | ITGA2B | SELPLG |  |  |
|  |  | ITGA2B | EPO |  |  |
|  |  | ITGA2B | CRP |  |  |
|  |  | ITGA2B | VWF |  |  |
|  |  | ITGA2B | IL1B |  |  |
|  |  | ITGA2B | TNF |  |  |
|  |  | ITGA2B | CXCR4 |  |  |
|  |  | ITGA2B | VEGFA |  |  |
|  |  | ITGA2B | CD28 |  |  |
|  |  | ITGA2B | ACE |  |  |
|  |  | ITGA2B | VCAM1 |  |  |
|  |  | ITGA2B | F10 |  |  |
|  |  | ITGA2B | IL6 |  |  |
|  |  | ITGA2B | TEK |  |  |
|  |  | ITGA2B | F7 |  |  |
|  |  | ITGA2B | FCGR2A |  |  |
|  |  | ITGA2B | SELE |  |  |
|  |  | ITGA2B | ITGA2 |  |  |
|  |  | ITGA2B | GP5 |  |  |
|  |  | ITGA2B | PLG |  |  |
|  |  | ITGA2B | SERPINC1 | |  |
|  |  | ITGA2B | THBS4 |  |  |
|  |  | ITGA2B | COL6A2 |  |  |
|  |  | ITGA2B | PPBP |  |  |
|  |  | ITGA2B | CD63 |  |  |
|  |  | ITGA2B | TNC |  |  |
|  |  | ITGA2B | ICAM1 |  |  |
|  |  | ITGA2B | F3 |  |  |
|  |  | ITGA2B | ITGB2 |  |  |
|  |  | ITGA2B | PF4 |  |  |
|  |  | ITGA2B | CD40LG |  |  |
|  |  | ITGA2B | F2 |  |  |
|  |  | ITGA2B | PECAM1 |  |  |
|  |  | ITGA2B | FERMT3 |  |  |
|  |  | ITGA2B | GP1BA |  |  |
|  |  | ITGA2B | FGA |  |  |
|  |  | ITGA2B | SELP |  |  |
|  |  | ITGA2B | ITGB3 |  |  |
|  |  | ITGB2 | CX3CL1 |  |  |
|  |  | ITGB2 | IL2RB |  |  |
|  |  | ITGB2 | MMP2 |  |  |
|  |  | ITGB2 | PLAT |  |  |
|  |  | ITGB2 | MPO |  |  |
|  |  | ITGB2 | CCL2 |  |  |
|  |  | ITGB2 | NFKB1 |  |  |
|  |  | ITGB2 | IL2 |  |  |
|  |  | ITGB2 | SELPLG |  |  |
|  |  | ITGB2 | APOB |  |  |
|  |  | ITGB2 | TLR2 |  |  |
|  |  | ITGB2 | VWF |  |  |
|  |  | ITGB2 | ITGA2B |  |  |
|  |  | ITGB2 | IL1B |  |  |
|  |  | ITGB2 | SELP |  |  |
|  |  | ITGB2 | ICAM1 |  |  |
|  |  | ITGB2 | KNG1 |  |  |
|  |  | ITGB2 | FCGR2A |  |  |
|  |  | ITGB2 | FERMT3 |  |  |
|  |  | ITGB2 | VCAM1 |  |  |
|  |  | ITGB2 | ITGA2 |  |  |
|  |  | ITGB2 | CD14 |  |  |
|  |  | ITGB2 | FGA |  |  |
|  |  | ITGB2 | CXCL8 |  |  |
|  |  | ITGB2 | PLG |  |  |
|  |  | ITGB2 | ANGPT2 |  |  |
|  |  | ITGB2 | CD28 |  |  |
|  |  | ITGB2 | GP1BA |  |  |
|  |  | ITGB2 | SELE |  |  |
|  |  | ITGB2 | CD86 |  |  |
|  |  | ITGB2 | CX3CR1 |  |  |
|  |  | ITGB2 | CD163 |  |  |
|  |  | ITGB2 | HLA-DRB1 | |  |
|  |  | ITGB2 | CTGF |  |  |
|  |  | ITGB2 | S100A8 |  |  |
|  |  | ITGB2 | S100A9 |  |  |
|  |  | ITGB2 | CTSS |  |  |
|  |  | ITGB2 | CD40LG |  |  |
|  |  | ITGB2 | MMP9 |  |  |
|  |  | ITGB2 | PLAU |  |  |
|  |  | ITGB2 | TLR4 |  |  |
|  |  | ITGB2 | AGER |  |  |
|  |  | ITGB2 | F10 |  |  |
|  |  | ITGB2 | TNFRSF1B | |  |
|  |  | ITGB2 | THBD |  |  |
|  |  | ITGB2 | YWHAZ |  |  |
|  |  | ITGB2 | IL10 |  |  |
|  |  | ITGB2 | IL6 |  |  |
|  |  | ITGB2 | CCL5 |  |  |
|  |  | ITGB2 | CCL3 |  |  |
|  |  | ITGB2 | CXCR4 |  |  |
|  |  | ITGB2 | PECAM1 |  |  |
|  |  | ITGB2 | TNF |  |  |
|  |  | ITGB2 | ITGB3 |  |  |
|  |  | ITGB3 | CX3CL1 |  |  |
|  |  | ITGB3 | TIMP1 |  |  |
|  |  | ITGB3 | MMP2 |  |  |
|  |  | ITGB3 | TGFB1 |  |  |
|  |  | ITGB3 | SERPINE1 |  |  |
|  |  | ITGB3 | MPO |  |  |
|  |  | ITGB3 | SELPLG |  |  |
|  |  | ITGB3 | LGALS3 |  |  |
|  |  | ITGB3 | TLR2 |  |  |
|  |  | ITGB3 | VWF |  |  |
|  |  | ITGB3 | ITGA2B |  |  |
|  |  | ITGB3 | IL1B |  |  |
|  |  | ITGB3 | SELP |  |  |
|  |  | ITGB3 | ICAM1 |  |  |
|  |  | ITGB3 | TNC |  |  |
|  |  | ITGB3 | PIK3C2A |  |  |
|  |  | ITGB3 | FERMT3 |  |  |
|  |  | ITGB3 | VCAM1 |  |  |
|  |  | ITGB3 | PPBP |  |  |
|  |  | ITGB3 | PF4 |  |  |
|  |  | ITGB3 | ITGA2 |  |  |
|  |  | ITGB3 | COL6A2 |  |  |
|  |  | ITGB3 | IGF1 |  |  |
|  |  | ITGB3 | FGA |  |  |
|  |  | ITGB3 | F2 |  |  |
|  |  | ITGB3 | CD28 |  |  |
|  |  | ITGB3 | CSF1 |  |  |
|  |  | ITGB3 | GP1BA |  |  |
|  |  | ITGB3 | SELE |  |  |
|  |  | ITGB3 | BSG |  |  |
|  |  | ITGB3 | F3 |  |  |
|  |  | ITGB3 | THBS4 |  |  |
|  |  | ITGB3 | PTGS2 |  |  |
|  |  | ITGB3 | MMP9 |  |  |
|  |  | ITGB3 | PLAU |  |  |
|  |  | ITGB3 | TLR4 |  |  |
|  |  | ITGB3 | YWHAZ |  |  |
|  |  | ITGB3 | ITGB2 |  |  |
|  |  | ITGB3 | PLA2G2A |  |  |
|  |  | ITGB3 | IL6 |  |  |
|  |  | ITGB3 | CXCR4 |  |  |
|  |  | ITGB3 | TNF |  |  |
|  |  | ITGB3 | CD63 |  |  |
|  |  | ITGB3 | VEGFA |  |  |
|  |  | ITGB3 | PECAM1 |  |  |
|  |  | ITIH4 | CCL2 |  |  |
|  |  | ITIH4 | IL2 |  |  |
|  |  | ITIH4 | IFNG |  |  |
|  |  | ITIH4 | HRG |  |  |
|  |  | ITIH4 | APOA1 |  |  |
|  |  | ITIH4 | CRP |  |  |
|  |  | ITIH4 | APCS |  |  |
|  |  | ITIH4 | TLR2 |  |  |
|  |  | ITIH4 | IL1B |  |  |
|  |  | ITIH4 | CP |  |  |
|  |  | ITIH4 | ICAM1 |  |  |
|  |  | ITIH4 | KNG1 |  |  |
|  |  | ITIH4 | TNC |  |  |
|  |  | ITIH4 | HABP2 |  |  |
|  |  | ITIH4 | CD40LG |  |  |
|  |  | ITIH4 | SERPING1 | |  |
|  |  | ITIH4 | CXCL8 |  |  |
|  |  | ITIH4 | SERPINF2 |  |  |
|  |  | ITIH4 | IFNA1 |  |  |
|  |  | ITIH4 | FGA |  |  |
|  |  | ITIH4 | F2 |  |  |
|  |  | ITIH4 | BDNF |  |  |
|  |  | ITIH4 | PLG |  |  |
|  |  | ITIH4 | CD28 |  |  |
|  |  | ITIH4 | IL10 |  |  |
|  |  | ITIH4 | CD86 |  |  |
|  |  | ITIH4 | CCL3 |  |  |
|  |  | ITIH4 | IL6 |  |  |
|  |  | ITIH4 | FCGR2A |  |  |
|  |  | ITIH4 | TLR4 |  |  |
|  |  | ITIH4 | TNF |  |  |
|  |  | ITIH4 | CCL5 |  |  |
|  |  | ITIH4 | MBL2 |  |  |
|  |  | ITIH4 | SERPINA1 | |  |
|  |  | ITIH4 | SERPINC1 | |  |
|  |  | ITIH4 | PF4 |  |  |
|  |  | ITIH4 | PTX3 |  |  |
|  |  | ITIH4 | DPP4 |  |  |
|  |  | ITIH4 | CXCR4 |  |  |
|  |  | KNG1 | CPB2 |  |  |
|  |  | KNG1 | PLAT |  |  |
|  |  | KNG1 | SERPINE1 |  |  |
|  |  | KNG1 | MPO |  |  |
|  |  | KNG1 | CCL2 |  |  |
|  |  | KNG1 | HRG |  |  |
|  |  | KNG1 | APOB |  |  |
|  |  | KNG1 | APOA1 |  |  |
|  |  | KNG1 | CRP |  |  |
|  |  | KNG1 | VWF |  |  |
|  |  | KNG1 | IL1B |  |  |
|  |  | KNG1 | SELP |  |  |
|  |  | KNG1 | CP |  |  |
|  |  | KNG1 | ICAM1 |  |  |
|  |  | KNG1 | PLA2G2A |  |  |
|  |  | KNG1 | IL10 |  |  |
|  |  | KNG1 | MMP9 |  |  |
|  |  | KNG1 | MBL2 |  |  |
|  |  | KNG1 | ANGPT1 |  |  |
|  |  | KNG1 | THBD |  |  |
|  |  | KNG1 | VCAM1 |  |  |
|  |  | KNG1 | DPP4 |  |  |
|  |  | KNG1 | MB |  |  |
|  |  | KNG1 | HABP2 |  |  |
|  |  | KNG1 | AVP |  |  |
|  |  | KNG1 | CXCL8 |  |  |
|  |  | KNG1 | F10 |  |  |
|  |  | KNG1 | ADM |  |  |
|  |  | KNG1 | F7 |  |  |
|  |  | KNG1 | PTGS2 |  |  |
|  |  | KNG1 | ITIH4 |  |  |
|  |  | KNG1 | C1S |  |  |
|  |  | KNG1 | VIP |  |  |
|  |  | KNG1 | VEGFA |  |  |
|  |  | KNG1 | IL6 |  |  |
|  |  | KNG1 | TNF |  |  |
|  |  | KNG1 | SERPINA1 | |  |
|  |  | KNG1 | SERPINF2 |  |  |
|  |  | KNG1 | GP5 |  |  |
|  |  | KNG1 | F3 |  |  |
|  |  | KNG1 | FGA |  |  |
|  |  | KNG1 | SERPINC1 | |  |
|  |  | KNG1 | AGT |  |  |
|  |  | KNG1 | INS |  |  |
|  |  | KNG1 | F2 |  |  |
|  |  | KNG1 | EDN1 |  |  |
|  |  | KNG1 | SERPING1 | |  |
|  |  | KNG1 | PLG |  |  |
|  |  | KNG1 | GP1BA |  |  |
|  |  | KNG1 | REN |  |  |
|  |  | KNG1 | ITGB2 |  |  |
|  |  | KNG1 | ACE |  |  |
|  |  | KNG1 | AGTR1 |  |  |
|  |  | KNG1 | NOS3 |  |  |
|  |  | LCN2 | HMOX1 |  |  |
|  |  | LCN2 | TIMP1 |  |  |
|  |  | LCN2 | MMP2 |  |  |
|  |  | LCN2 | RETN |  |  |
|  |  | LCN2 | TGFB1 |  |  |
|  |  | LCN2 | HGF |  |  |
|  |  | LCN2 | NAMPT |  |  |
|  |  | LCN2 | SERPINE1 |  |  |
|  |  | LCN2 | MPO |  |  |
|  |  | LCN2 | CCL2 |  |  |
|  |  | LCN2 | IFNG |  |  |
|  |  | LCN2 | MMP8 |  |  |
|  |  | LCN2 | GDF15 |  |  |
|  |  | LCN2 | LGALS3 |  |  |
|  |  | LCN2 | CRP |  |  |
|  |  | LCN2 | CHI3L1 |  |  |
|  |  | LCN2 | IL1RN |  |  |
|  |  | LCN2 | TLR2 |  |  |
|  |  | LCN2 | IL1A |  |  |
|  |  | LCN2 | IL1B |  |  |
|  |  | LCN2 | CP |  |  |
|  |  | LCN2 | ICAM1 |  |  |
|  |  | LCN2 | REN |  |  |
|  |  | LCN2 | IL18 |  |  |
|  |  | LCN2 | PPARG |  |  |
|  |  | LCN2 | ACE |  |  |
|  |  | LCN2 | VCAM1 |  |  |
|  |  | LCN2 | MMP3 |  |  |
|  |  | LCN2 | CXCL8 |  |  |
|  |  | LCN2 | LEP |  |  |
|  |  | LCN2 | MMP1 |  |  |
|  |  | LCN2 | IL17A |  |  |
|  |  | LCN2 | AGT |  |  |
|  |  | LCN2 | S100A8 |  |  |
|  |  | LCN2 | S100A9 |  |  |
|  |  | LCN2 | MMP9 |  |  |
|  |  | LCN2 | CCL3 |  |  |
|  |  | LCN2 | AVP |  |  |
|  |  | LCN2 | CCL5 |  |  |
|  |  | LCN2 | MB |  |  |
|  |  | LCN2 | FABP3 |  |  |
|  |  | LCN2 | SERPINA1 | |  |
|  |  | LCN2 | NPPB |  |  |
|  |  | LCN2 | TLR4 |  |  |
|  |  | LCN2 | INS |  |  |
|  |  | LCN2 | TNF |  |  |
|  |  | LCN2 | CST3 |  |  |
|  |  | LCN2 | VEGFA |  |  |
|  |  | LCN2 | IL10 |  |  |
|  |  | LCN2 | IL6 |  |  |
|  |  | LEP | CX3CL1 |  |  |
|  |  | LEP | TNFRSF1A | |  |
|  |  | LEP | SIRT1 |  |  |
|  |  | LEP | MIF |  |  |
|  |  | LEP | HMOX1 |  |  |
|  |  | LEP | IL2RB |  |  |
|  |  | LEP | TIMP1 |  |  |
|  |  | LEP | MMP2 |  |  |
|  |  | LEP | RETN |  |  |
|  |  | LEP | TGFB1 |  |  |
|  |  | LEP | PON1 |  |  |
|  |  | LEP | HGF |  |  |
|  |  | LEP | NAMPT |  |  |
|  |  | LEP | SERPINE1 |  |  |
|  |  | LEP | MPO |  |  |
|  |  | LEP | CCL2 |  |  |
|  |  | LEP | IL2 |  |  |
|  |  | LEP | IFNG |  |  |
|  |  | LEP | IL5 |  |  |
|  |  | LEP | APOB |  |  |
|  |  | LEP | APOA1 |  |  |
|  |  | LEP | EPO |  |  |
|  |  | LEP | CRP |  |  |
|  |  | LEP | FST |  |  |
|  |  | LEP | IL1RN |  |  |
|  |  | LEP | TLR2 |  |  |
|  |  | LEP | VWF |  |  |
|  |  | LEP | IL1A |  |  |
|  |  | LEP | IL1B |  |  |
|  |  | LEP | ICAM1 |  |  |
|  |  | LEP | PPARGC1A | |  |
|  |  | LEP | REN |  |  |
|  |  | LEP | IGFBP1 |  |  |
|  |  | LEP | IL18 |  |  |
|  |  | LEP | CXCL13 |  |  |
|  |  | LEP | PPARG |  |  |
|  |  | LEP | HMGCR |  |  |
|  |  | LEP | ACE |  |  |
|  |  | LEP | VCAM1 |  |  |
|  |  | LEP | TNFRSF11B | |  |
|  |  | LEP | NOS3 |  |  |
|  |  | LEP | MMP3 |  |  |
|  |  | LEP | CCL11 |  |  |
|  |  | LEP | IGF1 |  |  |
|  |  | LEP | ADRB2 |  |  |
|  |  | LEP | CXCL8 |  |  |
|  |  | LEP | PLG |  |  |
|  |  | LEP | VIP |  |  |
|  |  | LEP | ANGPT2 |  |  |
|  |  | LEP | AGTR1 |  |  |
|  |  | LEP | PECAM1 |  |  |
|  |  | LEP | ANGPTL2 |  |  |
|  |  | LEP | PGF |  |  |
|  |  | LEP | LCN2 |  |  |
|  |  | LEP | CCL3 |  |  |
|  |  | LEP | SELE |  |  |
|  |  | LEP | PTGS2 |  |  |
|  |  | LEP | HIF1A |  |  |
|  |  | LEP | CST3 |  |  |
|  |  | LEP | MMP9 |  |  |
|  |  | LEP | CCL5 |  |  |
|  |  | LEP | IL17A |  |  |
|  |  | LEP | EDN1 |  |  |
|  |  | LEP | TLR4 |  |  |
|  |  | LEP | IL2RA |  |  |
|  |  | LEP | SERPINA12 | |  |
|  |  | LEP | CNR1 |  |  |
|  |  | LEP | AGT |  |  |
|  |  | LEP | BGLAP |  |  |
|  |  | LEP | IL10 |  |  |
|  |  | LEP | BDNF |  |  |
|  |  | LEP | DPP4 |  |  |
|  |  | LEP | FGF21 |  |  |
|  |  | LEP | VEGFA |  |  |
|  |  | LEP | TNF |  |  |
|  |  | LEP | IL6 |  |  |
|  |  | LEP | INS |  |  |
|  |  | LGALS3 | TIMP1 |  |  |
|  |  | LGALS3 | MMP2 |  |  |
|  |  | LGALS3 | TGFB1 |  |  |
|  |  | LGALS3 | MPO |  |  |
|  |  | LGALS3 | CCL2 |  |  |
|  |  | LGALS3 | IFNG |  |  |
|  |  | LGALS3 | GDF15 |  |  |
|  |  | LGALS3 | TNNI3 |  |  |
|  |  | LGALS3 | ACE |  |  |
|  |  | LGALS3 | CCL5 |  |  |
|  |  | LGALS3 | SELP |  |  |
|  |  | LGALS3 | CCL3 |  |  |
|  |  | LGALS3 | GAS6 |  |  |
|  |  | LGALS3 | CSF1 |  |  |
|  |  | LGALS3 | ADM |  |  |
|  |  | LGALS3 | C1S |  |  |
|  |  | LGALS3 | SELE |  |  |
|  |  | LGALS3 | PTX3 |  |  |
|  |  | LGALS3 | FGA |  |  |
|  |  | LGALS3 | IL17A |  |  |
|  |  | LGALS3 | AGER |  |  |
|  |  | LGALS3 | CTGF |  |  |
|  |  | LGALS3 | CD163 |  |  |
|  |  | LGALS3 | PPARG |  |  |
|  |  | LGALS3 | S100A4 |  |  |
|  |  | LGALS3 | INS |  |  |
|  |  | LGALS3 | ICAM1 |  |  |
|  |  | LGALS3 | LCN2 |  |  |
|  |  | LGALS3 | MMP3 |  |  |
|  |  | LGALS3 | VEGFA |  |  |
|  |  | LGALS3 | CXCL8 |  |  |
|  |  | LGALS3 | CST3 |  |  |
|  |  | LGALS3 | PECAM1 |  |  |
|  |  | LGALS3 | FAS |  |  |
|  |  | LGALS3 | CRP |  |  |
|  |  | LGALS3 | CHI3L1 |  |  |
|  |  | LGALS3 | NPPB |  |  |
|  |  | LGALS3 | CD63 |  |  |
|  |  | LGALS3 | VCAM1 |  |  |
|  |  | LGALS3 | IL1B |  |  |
|  |  | LGALS3 | TNF |  |  |
|  |  | LGALS3 | IL10 |  |  |
|  |  | LGALS3 | MBL2 |  |  |
|  |  | LGALS3 | IL6 |  |  |
|  |  | LGALS3 | MMP9 |  |  |
|  |  | LGALS3 | ITGB3 |  |  |
|  |  | LGALS3 | FCGR2A |  |  |
|  |  | LGALS3 | VWF |  |  |
|  |  | LGALS3 | DPP4 |  |  |
|  |  | LGALS3 | BSG |  |  |
|  |  | LGALS3 | TLR2 |  |  |
|  |  | LGALS3 | TLR4 |  |  |
|  |  | LPXN | CETP |  |  |
|  |  | LPXN | APOB |  |  |
|  |  | LPXN | APOA1 |  |  |
|  |  | LPXN | CRP |  |  |
|  |  | LPXN | FERMT3 |  |  |
|  |  | MB | HMOX1 |  |  |
|  |  | MB | PYGB |  |  |
|  |  | MB | MPO |  |  |
|  |  | MB | TNNT2 |  |  |
|  |  | MB | EPO |  |  |
|  |  | MB | CRP |  |  |
|  |  | MB | CP |  |  |
|  |  | MB | PPARGC1A | |  |
|  |  | MB | KNG1 |  |  |
|  |  | MB | PIK3C2A |  |  |
|  |  | MB | ACE |  |  |
|  |  | MB | NOS3 |  |  |
|  |  | MB | HSPA4 |  |  |
|  |  | MB | CYCS |  |  |
|  |  | MB | F2 |  |  |
|  |  | MB | F3 |  |  |
|  |  | MB | TNNI3 |  |  |
|  |  | MB | TNNI1 |  |  |
|  |  | MB | LCN2 |  |  |
|  |  | MB | FABP3 |  |  |
|  |  | MB | NPPB |  |  |
|  |  | MB | AVP |  |  |
|  |  | MB | INS |  |  |
|  |  | MB | HIF1A |  |  |
|  |  | MB | CST3 |  |  |
|  |  | MB | TNF |  |  |
|  |  | MB | SERPINA1 | |  |
|  |  | MB | IL6 |  |  |
|  |  | MB | TNNT1 |  |  |
|  |  | MBL2 | CPB2 |  |  |
|  |  | MBL2 | HRG |  |  |
|  |  | MBL2 | APOB |  |  |
|  |  | MBL2 | APOA1 |  |  |
|  |  | MBL2 | LGALS3 |  |  |
|  |  | MBL2 | CRP |  |  |
|  |  | MBL2 | APCS |  |  |
|  |  | MBL2 | TLR2 |  |  |
|  |  | MBL2 | IL1B |  |  |
|  |  | MBL2 | KNG1 |  |  |
|  |  | MBL2 | ITIH4 |  |  |
|  |  | MBL2 | FCGR2A |  |  |
|  |  | MBL2 | HABP2 |  |  |
|  |  | MBL2 | SERPING1 | |  |
|  |  | MBL2 | PTX3 |  |  |
|  |  | MBL2 | FGA |  |  |
|  |  | MBL2 | CXCL8 |  |  |
|  |  | MBL2 | F2 |  |  |
|  |  | MBL2 | PLG |  |  |
|  |  | MBL2 | SERPINC1 | |  |
|  |  | MBL2 | SERPINA1 | |  |
|  |  | MBL2 | IL10 |  |  |
|  |  | MBL2 | TNF |  |  |
|  |  | MBL2 | IL6 |  |  |
|  |  | MBL2 | F7 |  |  |
|  |  | MBL2 | TLR4 |  |  |
|  |  | MBL2 | C1S |  |  |
|  |  | MIF | LEP |  |  |
|  |  | MIF | VCAM1 |  |  |
|  |  | MIF | IL1A |  |  |
|  |  | MIF | PTGS2 |  |  |
|  |  | MIF | HGF |  |  |
|  |  | MIF | INS |  |  |
|  |  | MIF | IL2 |  |  |
|  |  | MIF | SERPINE1 |  |  |
|  |  | MIF | MMP9 |  |  |
|  |  | MIF | ICAM1 |  |  |
|  |  | MIF | IL17A |  |  |
|  |  | MIF | TLR4 |  |  |
|  |  | MIF | CP |  |  |
|  |  | MIF | CCL3 |  |  |
|  |  | MIF | CCL5 |  |  |
|  |  | MIF | IFNG |  |  |
|  |  | MIF | IL18 |  |  |
|  |  | MIF | CRP |  |  |
|  |  | MIF | UBC |  |  |
|  |  | MIF | HIF1A |  |  |
|  |  | MIF | VEGFA |  |  |
|  |  | MIF | IL10 |  |  |
|  |  | MIF | IL1B |  |  |
|  |  | MIF | TNF |  |  |
|  |  | MIF | IL6 |  |  |
|  |  | MIF | CCL2 |  |  |
|  |  | MIF | CXCL8 |  |  |
|  |  | MIF | CXCR4 |  |  |
|  |  | MLKL | TNFRSF1A | |  |
|  |  | MLKL | RIPK3 |  |  |
|  |  | MLKL | RIPK1 |  |  |
|  |  | MLKL | IL1B |  |  |
|  |  | MLKL | IL18 |  |  |
|  |  | MLKL | CYCS |  |  |
|  |  | MLKL | PARP1 |  |  |
|  |  | MLKL | FAS |  |  |
|  |  | MLKL | MYD88 |  |  |
|  |  | MLKL | FASLG |  |  |
|  |  | MLKL | TLR4 |  |  |
|  |  | MLKL | TNF |  |  |
|  |  | MMP1 | TNFRSF1A | |  |
|  |  | MMP1 | HMOX1 |  |  |
|  |  | MMP1 | TIMP1 |  |  |
|  |  | MMP1 | MMP2 |  |  |
|  |  | MMP1 | PLAT |  |  |
|  |  | MMP1 | TGFB1 |  |  |
|  |  | MMP1 | HGF |  |  |
|  |  | MMP1 | SERPINE1 |  |  |
|  |  | MMP1 | MPO |  |  |
|  |  | MMP1 | CCL2 |  |  |
|  |  | MMP1 | IFNG |  |  |
|  |  | MMP1 | CRP |  |  |
|  |  | MMP1 | CHI3L1 |  |  |
|  |  | MMP1 | IL1RN |  |  |
|  |  | MMP1 | TLR2 |  |  |
|  |  | MMP1 | IL37 |  |  |
|  |  | MMP1 | IL1A |  |  |
|  |  | MMP1 | IL1B |  |  |
|  |  | MMP1 | ICAM1 |  |  |
|  |  | MMP1 | IL18 |  |  |
|  |  | MMP1 | ACE |  |  |
|  |  | MMP1 | VCAM1 |  |  |
|  |  | MMP1 | PTX3 |  |  |
|  |  | MMP1 | ITGA2 |  |  |
|  |  | MMP1 | TNFRSF11B | |  |
|  |  | MMP1 | MMP3 |  |  |
|  |  | MMP1 | IGF1 |  |  |
|  |  | MMP1 | CXCL8 |  |  |
|  |  | MMP1 | PLG |  |  |
|  |  | MMP1 | ANGPT2 |  |  |
|  |  | MMP1 | SMAD3 |  |  |
|  |  | MMP1 | CCL3 |  |  |
|  |  | MMP1 | PECAM1 |  |  |
|  |  | MMP1 | ANGPT1 |  |  |
|  |  | MMP1 | INS |  |  |
|  |  | MMP1 | TLR4 |  |  |
|  |  | MMP1 | SELE |  |  |
|  |  | MMP1 | CXCR4 |  |  |
|  |  | MMP1 | EDN1 |  |  |
|  |  | MMP1 | HIF1A |  |  |
|  |  | MMP1 | CCL5 |  |  |
|  |  | MMP1 | CD63 |  |  |
|  |  | MMP1 | CTGF |  |  |
|  |  | MMP1 | PTGS2 |  |  |
|  |  | MMP1 | PLAU |  |  |
|  |  | MMP1 | ADAMTS4 | |  |
|  |  | MMP1 | TNF |  |  |
|  |  | MMP1 | IL17A |  |  |
|  |  | MMP1 | LCN2 |  |  |
|  |  | MMP1 | IL10 |  |  |
|  |  | MMP1 | MMP9 |  |  |
|  |  | MMP1 | VEGFA |  |  |
|  |  | MMP1 | BSG |  |  |
|  |  | MMP1 | IL6 |  |  |
|  |  | MMP2 | TNFRSF1A | |  |
|  |  | MMP2 | SIRT1 |  |  |
|  |  | MMP2 | HMOX1 |  |  |
|  |  | MMP2 | TIMP1 |  |  |
|  |  | MMP2 | SERPINA1 | |  |
|  |  | MMP2 | HSPA4 |  |  |
|  |  | MMP2 | BDNF |  |  |
|  |  | MMP2 | CHI3L1 |  |  |
|  |  | MMP2 | DPP4 |  |  |
|  |  | MMP2 | BGLAP |  |  |
|  |  | MMP2 | IL18 |  |  |
|  |  | MMP2 | CCL3 |  |  |
|  |  | MMP2 | IL2 |  |  |
|  |  | MMP2 | CSF1 |  |  |
|  |  | MMP2 | F3 |  |  |
|  |  | MMP2 | PARP1 |  |  |
|  |  | MMP2 | FASLG |  |  |
|  |  | MMP2 | IFNG |  |  |
|  |  | MMP2 | AGT |  |  |
|  |  | MMP2 | AGTR1 |  |  |
|  |  | MMP2 | SELP |  |  |
|  |  | MMP2 | LEP |  |  |
|  |  | MMP2 | TNFRSF11B | |  |
|  |  | MMP2 | MYD88 |  |  |
|  |  | MMP2 | MMP3 |  |  |
|  |  | MMP2 | PGF |  |  |
|  |  | MMP2 | REN |  |  |
|  |  | MMP2 | VWF |  |  |
|  |  | MMP2 | CYCS |  |  |
|  |  | MMP2 | SELE |  |  |
|  |  | MMP2 | MPO |  |  |
|  |  | MMP2 | CCL5 |  |  |
|  |  | MMP2 | TLR4 |  |  |
|  |  | MMP2 | PPARG |  |  |
|  |  | MMP2 | CRP |  |  |
|  |  | MMP2 | IL1A |  |  |
|  |  | MMP2 | S100A4 |  |  |
|  |  | MMP2 | ANGPT2 |  |  |
|  |  | MMP2 | PCSK9 |  |  |
|  |  | MMP2 | ACE |  |  |
|  |  | MMP2 | INS |  |  |
|  |  | MMP2 | SMAD3 |  |  |
|  |  | MMP2 | LGALS3 |  |  |
|  |  | MMP2 | ADAMTS4 | |  |
|  |  | MMP2 | NOS3 |  |  |
|  |  | MMP2 | PECAM1 |  |  |
|  |  | MMP2 | ICAM1 |  |  |
|  |  | MMP2 | ITGB3 |  |  |
|  |  | MMP2 | PLAT |  |  |
|  |  | MMP2 | VCAM1 |  |  |
|  |  | MMP2 | CXCR4 |  |  |
|  |  | MMP2 | PTGS2 |  |  |
|  |  | MMP2 | CXCL8 |  |  |
|  |  | MMP2 | BSG |  |  |
|  |  | MMP2 | CCL2 |  |  |
|  |  | MMP2 | PLAU |  |  |
|  |  | MMP2 | CYBA |  |  |
|  |  | MMP2 | COL6A2 |  |  |
|  |  | MMP2 | SERPINE1 |  |  |
|  |  | MMP2 | TNF |  |  |
|  |  | MMP2 | CTGF |  |  |
|  |  | MMP2 | HIF1A |  |  |
|  |  | MMP2 | IL1B |  |  |
|  |  | MMP2 | TLR2 |  |  |
|  |  | MMP2 | PLG |  |  |
|  |  | MMP2 | MMP9 |  |  |
|  |  | MMP2 | EDN1 |  |  |
|  |  | MMP2 | MMP1 |  |  |
|  |  | MMP2 | ITGB2 |  |  |
|  |  | MMP2 | IL17A |  |  |
|  |  | MMP2 | TEK |  |  |
|  |  | MMP2 | ANGPT1 |  |  |
|  |  | MMP2 | IL10 |  |  |
|  |  | MMP2 | IGF1 |  |  |
|  |  | MMP2 | HGF |  |  |
|  |  | MMP2 | IL6 |  |  |
|  |  | MMP2 | LCN2 |  |  |
|  |  | MMP2 | VEGFA |  |  |
|  |  | MMP2 | TGFB1 |  |  |
|  |  | MMP28 | TIMP1 |  |  |
|  |  | MMP3 | TNFRSF1A | |  |
|  |  | MMP3 | HMOX1 |  |  |
|  |  | MMP3 | TIMP1 |  |  |
|  |  | MMP3 | MMP2 |  |  |
|  |  | MMP3 | PLAT |  |  |
|  |  | MMP3 | TGFB1 |  |  |
|  |  | MMP3 | HGF |  |  |
|  |  | MMP3 | SERPINE1 |  |  |
|  |  | MMP3 | MPO |  |  |
|  |  | MMP3 | CCL2 |  |  |
|  |  | MMP3 | IL2 |  |  |
|  |  | MMP3 | IFNG |  |  |
|  |  | MMP3 | LGALS3 |  |  |
|  |  | MMP3 | CRP |  |  |
|  |  | MMP3 | CHI3L1 |  |  |
|  |  | MMP3 | IL1RN |  |  |
|  |  | MMP3 | TLR2 |  |  |
|  |  | MMP3 | VWF |  |  |
|  |  | MMP3 | IL37 |  |  |
|  |  | MMP3 | IL1A |  |  |
|  |  | MMP3 | IL1B |  |  |
|  |  | MMP3 | ICAM1 |  |  |
|  |  | MMP3 | REN |  |  |
|  |  | MMP3 | IL18 |  |  |
|  |  | MMP3 | ACE |  |  |
|  |  | MMP3 | VCAM1 |  |  |
|  |  | MMP3 | PTX3 |  |  |
|  |  | MMP3 | TNFRSF11B | |  |
|  |  | MMP3 | CCL11 |  |  |
|  |  | MMP3 | AGT |  |  |
|  |  | MMP3 | SMAD3 |  |  |
|  |  | MMP3 | LEP |  |  |
|  |  | MMP3 | BGLAP |  |  |
|  |  | MMP3 | CXCR4 |  |  |
|  |  | MMP3 | EDN1 |  |  |
|  |  | MMP3 | FGF21 |  |  |
|  |  | MMP3 | CTSS |  |  |
|  |  | MMP3 | HIF1A |  |  |
|  |  | MMP3 | SELE |  |  |
|  |  | MMP3 | CCL3 |  |  |
|  |  | MMP3 | BSG |  |  |
|  |  | MMP3 | INS |  |  |
|  |  | MMP3 | TLR4 |  |  |
|  |  | MMP3 | PECAM1 |  |  |
|  |  | MMP3 | CCL5 |  |  |
|  |  | MMP3 | IGF1 |  |  |
|  |  | MMP3 | PLAU |  |  |
|  |  | MMP3 | CTGF |  |  |
|  |  | MMP3 | PTGS2 |  |  |
|  |  | MMP3 | CXCL8 |  |  |
|  |  | MMP3 | TNF |  |  |
|  |  | MMP3 | ADAMTS4 | |  |
|  |  | MMP3 | MMP9 |  |  |
|  |  | MMP3 | BDNF |  |  |
|  |  | MMP3 | LCN2 |  |  |
|  |  | MMP3 | MMP1 |  |  |
|  |  | MMP3 | IL17A |  |  |
|  |  | MMP3 | IL10 |  |  |
|  |  | MMP3 | VEGFA |  |  |
|  |  | MMP3 | IL6 |  |  |
|  |  | MMP3 | PLG |  |  |
|  |  | MMP8 | SIRT1 |  |  |
|  |  | MMP8 | TIMP1 |  |  |
|  |  | MMP8 | HGF |  |  |
|  |  | MMP8 | SERPINE1 |  |  |
|  |  | MMP8 | MPO |  |  |
|  |  | MMP8 | CCL2 |  |  |
|  |  | MMP8 | CCL5 |  |  |
|  |  | MMP8 | F2 |  |  |
|  |  | MMP8 | CTGF |  |  |
|  |  | MMP8 | ICAM1 |  |  |
|  |  | MMP8 | SERPINA1 | |  |
|  |  | MMP8 | IGF1 |  |  |
|  |  | MMP8 | TLR4 |  |  |
|  |  | MMP8 | VCAM1 |  |  |
|  |  | MMP8 | PECAM1 |  |  |
|  |  | MMP8 | IL17A |  |  |
|  |  | MMP8 | IL1A |  |  |
|  |  | MMP8 | PLAU |  |  |
|  |  | MMP8 | CCL3 |  |  |
|  |  | MMP8 | PTGS2 |  |  |
|  |  | MMP8 | CRP |  |  |
|  |  | MMP8 | IL10 |  |  |
|  |  | MMP8 | VEGFA |  |  |
|  |  | MMP8 | ADAMTS4 | |  |
|  |  | MMP8 | LCN2 |  |  |
|  |  | MMP8 | MMP9 |  |  |
|  |  | MMP8 | TNF |  |  |
|  |  | MMP8 | IL6 |  |  |
|  |  | MMP8 | CXCL8 |  |  |
|  |  | MMP8 | IL1B |  |  |
|  |  | MMP8 | PLG |  |  |
|  |  | MMP9 | CX3CL1 |  |  |
|  |  | MMP9 | TNFRSF1A | |  |
|  |  | MMP9 | SIRT1 |  |  |
|  |  | MMP9 | MIF |  |  |
|  |  | MMP9 | HMOX1 |  |  |
|  |  | MMP9 | TIMP1 |  |  |
|  |  | MMP9 | MMP2 |  |  |
|  |  | MMP9 | PLAT |  |  |
|  |  | MMP9 | RETN |  |  |
|  |  | MMP9 | TGFB1 |  |  |
|  |  | MMP9 | HGF |  |  |
|  |  | MMP9 | SERPINE1 |  |  |
|  |  | MMP9 | MPO |  |  |
|  |  | MMP9 | CCL2 |  |  |
|  |  | MMP9 | NFKB1 |  |  |
|  |  | MMP9 | IL2 |  |  |
|  |  | MMP9 | IFNG |  |  |
|  |  | MMP9 | IL5 |  |  |
|  |  | MMP9 | MMP8 |  |  |
|  |  | MMP9 | APOA1 |  |  |
|  |  | MMP9 | EPO |  |  |
|  |  | MMP9 | LGALS3 |  |  |
|  |  | MMP9 | CRP |  |  |
|  |  | MMP9 | CHI3L1 |  |  |
|  |  | MMP9 | IL1RN |  |  |
|  |  | MMP9 | TLR2 |  |  |
|  |  | MMP9 | VWF |  |  |
|  |  | MMP9 | CYBA |  |  |
|  |  | MMP9 | IL37 |  |  |
|  |  | MMP9 | IL1A |  |  |
|  |  | MMP9 | IL1B |  |  |
|  |  | MMP9 | SELP |  |  |
|  |  | MMP9 | ICAM1 |  |  |
|  |  | MMP9 | KNG1 |  |  |
|  |  | MMP9 | REN |  |  |
|  |  | MMP9 | IGFBP1 |  |  |
|  |  | MMP9 | IFNA1 |  |  |
|  |  | MMP9 | IL18 |  |  |
|  |  | MMP9 | BMP6 |  |  |
|  |  | MMP9 | CXCL13 |  |  |
|  |  | MMP9 | PPARG |  |  |
|  |  | MMP9 | ACE |  |  |
|  |  | MMP9 | CXCL16 |  |  |
|  |  | MMP9 | VCAM1 |  |  |
|  |  | MMP9 | PTX3 |  |  |
|  |  | MMP9 | PPBP |  |  |
|  |  | MMP9 | PF4 |  |  |
|  |  | MMP9 | ITGA2 |  |  |
|  |  | MMP9 | TNFRSF11B | |  |
|  |  | MMP9 | NOS3 |  |  |
|  |  | MMP9 | MMP3 |  |  |
|  |  | MMP9 | CCL11 |  |  |
|  |  | MMP9 | IGF1 |  |  |
|  |  | MMP9 | HSPA4 |  |  |
|  |  | MMP9 | CD14 |  |  |
|  |  | MMP9 | CXCL8 |  |  |
|  |  | MMP9 | CYCS |  |  |
|  |  | MMP9 | F2 |  |  |
|  |  | MMP9 | PLG |  |  |
|  |  | MMP9 | OLR1 |  |  |
|  |  | MMP9 | LEP |  |  |
|  |  | MMP9 | ANGPT2 |  |  |
|  |  | MMP9 | MMP1 |  |  |
|  |  | MMP9 | CSF1 |  |  |
|  |  | MMP9 | SELE |  |  |
|  |  | MMP9 | CD86 |  |  |
|  |  | MMP9 | SMAD3 |  |  |
|  |  | MMP9 | BSG |  |  |
|  |  | MMP9 | F3 |  |  |
|  |  | MMP9 | IL17A |  |  |
|  |  | MMP9 | CX3CR1 |  |  |
|  |  | MMP9 | CD163 |  |  |
|  |  | MMP9 | DPP4 |  |  |
|  |  | MMP9 | AGT |  |  |
|  |  | MMP9 | PTGS2 |  |  |
|  |  | MMP9 | FASLG |  |  |
|  |  | MMP9 | CTGF |  |  |
|  |  | MMP9 | ADAMTS4 | |  |
|  |  | MMP9 | BGLAP |  |  |
|  |  | MMP9 | S100A4 |  |  |
|  |  | MMP9 | S100A8 |  |  |
|  |  | MMP9 | S100A9 |  |  |
|  |  | MMP9 | CTSS |  |  |
|  |  | MMP9 | CD40LG |  |  |
|  |  | MMP9 | CST3 |  |  |
|  |  | MMP9 | THBD |  |  |
|  |  | MMP9 | TNFRSF1B | |  |
|  |  | MMP9 | CD63 |  |  |
|  |  | MMP9 | IL33 |  |  |
|  |  | MMP9 | AGTR1 |  |  |
|  |  | MMP9 | ITGB3 |  |  |
|  |  | MMP9 | AGER |  |  |
|  |  | MMP9 | MYD88 |  |  |
|  |  | MMP9 | SERPINA1 | |  |
|  |  | MMP9 | BDNF |  |  |
|  |  | MMP9 | PGF |  |  |
|  |  | MMP9 | TEK |  |  |
|  |  | MMP9 | ANGPT1 |  |  |
|  |  | MMP9 | INS |  |  |
|  |  | MMP9 | CCL3 |  |  |
|  |  | MMP9 | CCL5 |  |  |
|  |  | MMP9 | HIF1A |  |  |
|  |  | MMP9 | PECAM1 |  |  |
|  |  | MMP9 | CXCR4 |  |  |
|  |  | MMP9 | PLAU |  |  |
|  |  | MMP9 | TLR4 |  |  |
|  |  | MMP9 | TNF |  |  |
|  |  | MMP9 | EDN1 |  |  |
|  |  | MMP9 | ITGB2 |  |  |
|  |  | MMP9 | IL10 |  |  |
|  |  | MMP9 | IL6 |  |  |
|  |  | MMP9 | VEGFA |  |  |
|  |  | MMP9 | LCN2 |  |  |
|  |  | MON2 | CAD |  |  |
|  |  | MORF4L2 | HDAC9 |  |  |
|  |  | MPO | TNFRSF1A | |  |
|  |  | MPO | HMOX1 |  |  |
|  |  | MPO | TIMP1 |  |  |
|  |  | MPO | MMP2 |  |  |
|  |  | MPO | RETN |  |  |
|  |  | MPO | PON1 |  |  |
|  |  | MPO | SERPINE1 |  |  |
|  |  | MPO | CD86 |  |  |
|  |  | MPO | IL33 |  |  |
|  |  | MPO | GDF15 |  |  |
|  |  | MPO | MMP1 |  |  |
|  |  | MPO | HSPA4 |  |  |
|  |  | MPO | PAPPA |  |  |
|  |  | MPO | NPPB |  |  |
|  |  | MPO | REN |  |  |
|  |  | MPO | OLR1 |  |  |
|  |  | MPO | PRG2 |  |  |
|  |  | MPO | LGALS3 |  |  |
|  |  | MPO | CYCS |  |  |
|  |  | MPO | SELPLG |  |  |
|  |  | MPO | TNFRSF1B | |  |
|  |  | MPO | PPBP |  |  |
|  |  | MPO | PF4 |  |  |
|  |  | MPO | MMP3 |  |  |
|  |  | MPO | SERPINC1 | |  |
|  |  | MPO | CST3 |  |  |
|  |  | MPO | LEP |  |  |
|  |  | MPO | AGER |  |  |
|  |  | MPO | EDN1 |  |  |
|  |  | MPO | KNG1 |  |  |
|  |  | MPO | FCGR2A |  |  |
|  |  | MPO | PPARG |  |  |
|  |  | MPO | MB |  |  |
|  |  | MPO | INS |  |  |
|  |  | MPO | ITGB2 |  |  |
|  |  | MPO | F2 |  |  |
|  |  | MPO | CYBA |  |  |
|  |  | MPO | TNNI3 |  |  |
|  |  | MPO | CD40LG |  |  |
|  |  | MPO | S100A9 |  |  |
|  |  | MPO | IL2 |  |  |
|  |  | MPO | IL5 |  |  |
|  |  | MPO | S100A8 |  |  |
|  |  | MPO | PLG |  |  |
|  |  | MPO | MYD88 |  |  |
|  |  | MPO | CD163 |  |  |
|  |  | MPO | ACE |  |  |
|  |  | MPO | APOB |  |  |
|  |  | MPO | PECAM1 |  |  |
|  |  | MPO | CCL5 |  |  |
|  |  | MPO | VWF |  |  |
|  |  | MPO | ITGB3 |  |  |
|  |  | MPO | IL18 |  |  |
|  |  | MPO | IL1A |  |  |
|  |  | MPO | VEGFA |  |  |
|  |  | MPO | ITGA2B |  |  |
|  |  | MPO | NOS3 |  |  |
|  |  | MPO | IFNG |  |  |
|  |  | MPO | CCL3 |  |  |
|  |  | MPO | F3 |  |  |
|  |  | MPO | SERPINA1 | |  |
|  |  | MPO | TLR2 |  |  |
|  |  | MPO | VCAM1 |  |  |
|  |  | MPO | PTX3 |  |  |
|  |  | MPO | SELE |  |  |
|  |  | MPO | LCN2 |  |  |
|  |  | MPO | MMP8 |  |  |
|  |  | MPO | PTGS2 |  |  |
|  |  | MPO | IL17A |  |  |
|  |  | MPO | CCL2 |  |  |
|  |  | MPO | SELP |  |  |
|  |  | MPO | TLR4 |  |  |
|  |  | MPO | MMP9 |  |  |
|  |  | MPO | ICAM1 |  |  |
|  |  | MPO | IL10 |  |  |
|  |  | MPO | CXCL8 |  |  |
|  |  | MPO | CRP |  |  |
|  |  | MPO | IL1B |  |  |
|  |  | MPO | IL6 |  |  |
|  |  | MPO | TNF |  |  |
|  |  | MPO | RNASE3 |  |  |
|  |  | MPO | APOA1 |  |  |
|  |  | MPO | CP |  |  |
|  |  | MRAS | CD40LG |  |  |
|  |  | MRAS | SEMA4D |  |  |
|  |  | MYD88 | TNFRSF1A | |  |
|  |  | MYD88 | HMOX1 |  |  |
|  |  | MYD88 | RIPK3 |  |  |
|  |  | MYD88 | MMP2 |  |  |
|  |  | MYD88 | MPO |  |  |
|  |  | MYD88 | CCL2 |  |  |
|  |  | MYD88 | NFKB1 |  |  |
|  |  | MYD88 | IL2 |  |  |
|  |  | MYD88 | IFNG |  |  |
|  |  | MYD88 | IL5 |  |  |
|  |  | MYD88 | CRP |  |  |
|  |  | MYD88 | IL1RN |  |  |
|  |  | MYD88 | RIPK1 |  |  |
|  |  | MYD88 | TLR2 |  |  |
|  |  | MYD88 | IL37 |  |  |
|  |  | MYD88 | IL1A |  |  |
|  |  | MYD88 | IL1B |  |  |
|  |  | MYD88 | ICAM1 |  |  |
|  |  | MYD88 | FCGR2A |  |  |
|  |  | MYD88 | IFNA1 |  |  |
|  |  | MYD88 | IL18 |  |  |
|  |  | MYD88 | PPARG |  |  |
|  |  | MYD88 | VCAM1 |  |  |
|  |  | MYD88 | PTX3 |  |  |
|  |  | MYD88 | HSPA4 |  |  |
|  |  | MYD88 | CD14 |  |  |
|  |  | MYD88 | CXCL8 |  |  |
|  |  | MYD88 | MLKL |  |  |
|  |  | MYD88 | CD28 |  |  |
|  |  | MYD88 | CSF1 |  |  |
|  |  | MYD88 | SELE |  |  |
|  |  | MYD88 | CD86 |  |  |
|  |  | MYD88 | IL17A |  |  |
|  |  | MYD88 | FAS |  |  |
|  |  | MYD88 | CX3CR1 |  |  |
|  |  | MYD88 | PTGS2 |  |  |
|  |  | MYD88 | S100A8 |  |  |
|  |  | MYD88 | S100A9 |  |  |
|  |  | MYD88 | CD40LG |  |  |
|  |  | MYD88 | MMP9 |  |  |
|  |  | MYD88 | TLR4 |  |  |
|  |  | MYD88 | AGER |  |  |
|  |  | MYD88 | THBD |  |  |
|  |  | MYD88 | IL33 |  |  |
|  |  | MYD88 | HSPD1 |  |  |
|  |  | MYD88 | INS |  |  |
|  |  | MYD88 | IL6 |  |  |
|  |  | MYD88 | CXCR4 |  |  |
|  |  | MYD88 | IL18R1 |  |  |
|  |  | MYD88 | TNF |  |  |
|  |  | MYD88 | HIF1A |  |  |
|  |  | MYD88 | VEGFA |  |  |
|  |  | MYD88 | CCL3 |  |  |
|  |  | MYD88 | CCL5 |  |  |
|  |  | MYD88 | IL10 |  |  |
|  |  | NAMPT | SIRT1 |  |  |
|  |  | NAMPT | TIMP1 |  |  |
|  |  | NAMPT | RETN |  |  |
|  |  | NAMPT | NOS3 |  |  |
|  |  | NAMPT | LCN2 |  |  |
|  |  | NAMPT | PTGS2 |  |  |
|  |  | NAMPT | BGLAP |  |  |
|  |  | NAMPT | VEGFA |  |  |
|  |  | NAMPT | IGF1 |  |  |
|  |  | NAMPT | IL10 |  |  |
|  |  | NAMPT | AGT |  |  |
|  |  | NAMPT | HIF1A |  |  |
|  |  | NAMPT | FGF21 |  |  |
|  |  | NAMPT | PPARG |  |  |
|  |  | NAMPT | PARP1 |  |  |
|  |  | NAMPT | CCL2 |  |  |
|  |  | NAMPT | IL1B |  |  |
|  |  | NAMPT | CXCL8 |  |  |
|  |  | NAMPT | PPARGC1A | |  |
|  |  | NAMPT | CRP |  |  |
|  |  | NAMPT | SERPINE1 |  |  |
|  |  | NAMPT | IL6 |  |  |
|  |  | NAMPT | TNF |  |  |
|  |  | NAMPT | INS |  |  |
|  |  | NAMPT | TLR4 |  |  |
|  |  | NAMPT | SERPINA12 | |  |
|  |  | NAMPT | LEP |  |  |
|  |  | NAMPT | IL7 |  |  |
|  |  | NFKB1 | TNFRSF1A | |  |
|  |  | NFKB1 | SIRT1 |  |  |
|  |  | NFKB1 | RIPK3 |  |  |
|  |  | NFKB1 | TGFB1 |  |  |
|  |  | NFKB1 | CCL2 |  |  |
|  |  | NFKB1 | PPARGC1A | |  |
|  |  | NFKB1 | IL2 |  |  |
|  |  | NFKB1 | VCAM1 |  |  |
|  |  | NFKB1 | MMP9 |  |  |
|  |  | NFKB1 | HIF1A |  |  |
|  |  | NFKB1 | ICAM1 |  |  |
|  |  | NFKB1 | FASLG |  |  |
|  |  | NFKB1 | IL2RA |  |  |
|  |  | NFKB1 | IFNG |  |  |
|  |  | NFKB1 | HSPA4 |  |  |
|  |  | NFKB1 | IL10 |  |  |
|  |  | NFKB1 | PTGS2 |  |  |
|  |  | NFKB1 | IFNA1 |  |  |
|  |  | NFKB1 | UBC |  |  |
|  |  | NFKB1 | RPS27A |  |  |
|  |  | NFKB1 | PARP1 |  |  |
|  |  | NFKB1 | CCL5 |  |  |
|  |  | NFKB1 | TEK |  |  |
|  |  | NFKB1 | ANGPT1 |  |  |
|  |  | NFKB1 | EPO |  |  |
|  |  | NFKB1 | TLR2 |  |  |
|  |  | NFKB1 | IL18R1 |  |  |
|  |  | NFKB1 | TLR4 |  |  |
|  |  | NFKB1 | ITGB2 |  |  |
|  |  | NFKB1 | AGER |  |  |
|  |  | NFKB1 | IL18 |  |  |
|  |  | NFKB1 | CD40LG |  |  |
|  |  | NFKB1 | IL1A |  |  |
|  |  | NFKB1 | RIPK1 |  |  |
|  |  | NFKB1 | PPARG |  |  |
|  |  | NFKB1 | CXCL8 |  |  |
|  |  | NFKB1 | IL6 |  |  |
|  |  | NFKB1 | IL1B |  |  |
|  |  | NFKB1 | TNF |  |  |
|  |  | NFKB1 | MYD88 |  |  |
|  |  | NOS3 | SIRT1 |  |  |
|  |  | NOS3 | HMOX1 |  |  |
|  |  | NOS3 | TIMP1 |  |  |
|  |  | NOS3 | MMP2 |  |  |
|  |  | NOS3 | PLAT |  |  |
|  |  | NOS3 | RETN |  |  |
|  |  | NOS3 | TGFB1 |  |  |
|  |  | NOS3 | PON1 |  |  |
|  |  | NOS3 | HGF |  |  |
|  |  | NOS3 | NAMPT |  |  |
|  |  | NOS3 | SERPINE1 |  |  |
|  |  | NOS3 | MPO |  |  |
|  |  | NOS3 | CCL2 |  |  |
|  |  | NOS3 | IFNG |  |  |
|  |  | NOS3 | APOB |  |  |
|  |  | NOS3 | APOA1 |  |  |
|  |  | NOS3 | EPO |  |  |
|  |  | NOS3 | CRP |  |  |
|  |  | NOS3 | VWF |  |  |
|  |  | NOS3 | CYBA |  |  |
|  |  | NOS3 | IL1B |  |  |
|  |  | NOS3 | SELP |  |  |
|  |  | NOS3 | ICAM1 |  |  |
|  |  | NOS3 | PPARGC1A | |  |
|  |  | NOS3 | KNG1 |  |  |
|  |  | NOS3 | REN |  |  |
|  |  | NOS3 | PPARG |  |  |
|  |  | NOS3 | HMGCR |  |  |
|  |  | NOS3 | ACE |  |  |
|  |  | NOS3 | VCAM1 |  |  |
|  |  | NOS3 | F2 |  |  |
|  |  | NOS3 | AGER |  |  |
|  |  | NOS3 | NPPB |  |  |
|  |  | NOS3 | CTGF |  |  |
|  |  | NOS3 | ADM |  |  |
|  |  | NOS3 | DPP4 |  |  |
|  |  | NOS3 | CXCR4 |  |  |
|  |  | NOS3 | HSPA4 |  |  |
|  |  | NOS3 | ADRB2 |  |  |
|  |  | NOS3 | ANGPT2 |  |  |
|  |  | NOS3 | MB |  |  |
|  |  | NOS3 | PGF |  |  |
|  |  | NOS3 | PLG |  |  |
|  |  | NOS3 | TLR4 |  |  |
|  |  | NOS3 | BDNF |  |  |
|  |  | NOS3 | F3 |  |  |
|  |  | NOS3 | IGF1 |  |  |
|  |  | NOS3 | ANGPT1 |  |  |
|  |  | NOS3 | LEP |  |  |
|  |  | NOS3 | THBD |  |  |
|  |  | NOS3 | OLR1 |  |  |
|  |  | NOS3 | CXCL8 |  |  |
|  |  | NOS3 | CYCS |  |  |
|  |  | NOS3 | IL10 |  |  |
|  |  | NOS3 | AGT |  |  |
|  |  | NOS3 | MMP9 |  |  |
|  |  | NOS3 | TEK |  |  |
|  |  | NOS3 | SELE |  |  |
|  |  | NOS3 | PTGS2 |  |  |
|  |  | NOS3 | IL6 |  |  |
|  |  | NOS3 | PECAM1 |  |  |
|  |  | NOS3 | TNF |  |  |
|  |  | NOS3 | INS |  |  |
|  |  | NOS3 | EDN1 |  |  |
|  |  | NOS3 | AGTR1 |  |  |
|  |  | NOS3 | HIF1A |  |  |
|  |  | NOS3 | VEGFA |  |  |
|  |  | NPPB | SERPINE1 |  |  |
|  |  | NPPB | MPO |  |  |
|  |  | NPPB | TNNT2 |  |  |
|  |  | NPPB | GDF15 |  |  |
|  |  | NPPB | LGALS3 |  |  |
|  |  | NPPB | CRP |  |  |
|  |  | NPPB | PPARGC1A | |  |
|  |  | NPPB | PIK3C2A |  |  |
|  |  | NPPB | REN |  |  |
|  |  | NPPB | ACE |  |  |
|  |  | NPPB | NOS3 |  |  |
|  |  | NPPB | F2 |  |  |
|  |  | NPPB | TNNI3 |  |  |
|  |  | NPPB | DPP4 |  |  |
|  |  | NPPB | AGT |  |  |
|  |  | NPPB | CTGF |  |  |
|  |  | NPPB | LCN2 |  |  |
|  |  | NPPB | FABP3 |  |  |
|  |  | NPPB | TNF |  |  |
|  |  | NPPB | INS |  |  |
|  |  | NPPB | IL6 |  |  |
|  |  | NPPB | MB |  |  |
|  |  | NPPB | AVP |  |  |
|  |  | NPPB | ADM |  |  |
|  |  | NPPB | CST3 |  |  |
|  |  | NPPB | TNNT1 |  |  |
|  |  | NPPB | EDN1 |  |  |
|  |  | OLR1 | PON1 |  |  |
|  |  | OLR1 | MPO |  |  |
|  |  | OLR1 | CCL2 |  |  |
|  |  | OLR1 | APOB |  |  |
|  |  | OLR1 | APOA1 |  |  |
|  |  | OLR1 | CRP |  |  |
|  |  | OLR1 | TLR2 |  |  |
|  |  | OLR1 | IL1B |  |  |
|  |  | OLR1 | SELP |  |  |
|  |  | OLR1 | ICAM1 |  |  |
|  |  | OLR1 | PPARG |  |  |
|  |  | OLR1 | CXCL16 |  |  |
|  |  | OLR1 | VCAM1 |  |  |
|  |  | OLR1 | NOS3 |  |  |
|  |  | OLR1 | HSPA4 |  |  |
|  |  | OLR1 | PCSK9 |  |  |
|  |  | OLR1 | CXCL8 |  |  |
|  |  | OLR1 | IL10 |  |  |
|  |  | OLR1 | F3 |  |  |
|  |  | OLR1 | MMP9 |  |  |
|  |  | OLR1 | EDN1 |  |  |
|  |  | OLR1 | SELE |  |  |
|  |  | OLR1 | IL6 |  |  |
|  |  | OLR1 | AGTR1 |  |  |
|  |  | OLR1 | TNF |  |  |
|  |  | OLR1 | TLR4 |  |  |
|  |  | OLR1 | HSPD1 |  |  |
|  |  | PAPPA | MPO |  |  |
|  |  | PAPPA | CRP |  |  |
|  |  | PAPPA | IGFBP1 |  |  |
|  |  | PAPPA | IGF1 |  |  |
|  |  | PAPPA | PRG2 |  |  |
|  |  | PAPPA | INS |  |  |
|  |  | PAPPA | TNNI3 |  |  |
|  |  | PAPPA | PGF |  |  |
|  |  | PARK2 | SIRT1 |  |  |
|  |  | PARK2 | RIPK1 |  |  |
|  |  | PARK2 | PPARGC1A | |  |
|  |  | PARK2 | RPS27A |  |  |
|  |  | PARK2 | HSPA4 |  |  |
|  |  | PARK2 | CYCS |  |  |
|  |  | PARK2 | BDNF |  |  |
|  |  | PARK2 | HSPD1 |  |  |
|  |  | PARK2 | HIF1A |  |  |
|  |  | PARK2 | UBC |  |  |
|  |  | PARP1 | SIRT1 |  |  |
|  |  | PARP1 | RIPK3 |  |  |
|  |  | PARP1 | GZMB |  |  |
|  |  | PARP1 | MMP2 |  |  |
|  |  | PARP1 | NAMPT |  |  |
|  |  | PARP1 | NFKB1 |  |  |
|  |  | PARP1 | IL2 |  |  |
|  |  | PARP1 | RIPK1 |  |  |
|  |  | PARP1 | IL1B |  |  |
|  |  | PARP1 | PPARGC1A | |  |
|  |  | PARP1 | RPS27A |  |  |
|  |  | PARP1 | HSPA4 |  |  |
|  |  | PARP1 | CYCS |  |  |
|  |  | PARP1 | MLKL |  |  |
|  |  | PARP1 | SMAD3 |  |  |
|  |  | PARP1 | HDAC9 |  |  |
|  |  | PARP1 | VEGFA |  |  |
|  |  | PARP1 | IL6 |  |  |
|  |  | PARP1 | RAB3IL1 |  |  |
|  |  | PARP1 | INS |  |  |
|  |  | PARP1 | IL10 |  |  |
|  |  | PARP1 | TNF |  |  |
|  |  | PARP1 | UBC |  |  |
|  |  | PARP1 | HIF1A |  |  |
|  |  | PARP1 | BRCA1 |  |  |
|  |  | PCSK9 | CETP |  |  |
|  |  | PCSK9 | MMP2 |  |  |
|  |  | PCSK9 | RETN |  |  |
|  |  | PCSK9 | APOB |  |  |
|  |  | PCSK9 | APOA1 |  |  |
|  |  | PCSK9 | CRP |  |  |
|  |  | PCSK9 | REN |  |  |
|  |  | PCSK9 | HMGCR |  |  |
|  |  | PCSK9 | ACE |  |  |
|  |  | PCSK9 | IL6 |  |  |
|  |  | PCSK9 | OLR1 |  |  |
|  |  | PCSK9 | INS |  |  |
|  |  | PECAM1 | CX3CL1 |  |  |
|  |  | PECAM1 | HMOX1 |  |  |
|  |  | PECAM1 | GZMB |  |  |
|  |  | PECAM1 | TIMP1 |  |  |
|  |  | PECAM1 | MMP2 |  |  |
|  |  | PECAM1 | TGFB1 |  |  |
|  |  | PECAM1 | HGF |  |  |
|  |  | PECAM1 | SERPINE1 |  |  |
|  |  | PECAM1 | MPO |  |  |
|  |  | PECAM1 | CCL2 |  |  |
|  |  | PECAM1 | IL2 |  |  |
|  |  | PECAM1 | SELPLG |  |  |
|  |  | PECAM1 | IFNG |  |  |
|  |  | PECAM1 | APOB |  |  |
|  |  | PECAM1 | MMP8 |  |  |
|  |  | PECAM1 | TNNT2 |  |  |
|  |  | PECAM1 | EPO |  |  |
|  |  | PECAM1 | LGALS3 |  |  |
|  |  | PECAM1 | CRP |  |  |
|  |  | PECAM1 | TLR2 |  |  |
|  |  | PECAM1 | VWF |  |  |
|  |  | PECAM1 | ITGA2B |  |  |
|  |  | PECAM1 | IL1A |  |  |
|  |  | PECAM1 | IL1B |  |  |
|  |  | PECAM1 | SELP |  |  |
|  |  | PECAM1 | IL7 |  |  |
|  |  | PECAM1 | ICAM1 |  |  |
|  |  | PECAM1 | FCGR2A |  |  |
|  |  | PECAM1 | PPARG |  |  |
|  |  | PECAM1 | ACE |  |  |
|  |  | PECAM1 | VCAM1 |  |  |
|  |  | PECAM1 | PF4 |  |  |
|  |  | PECAM1 | ITGA2 |  |  |
|  |  | PECAM1 | NOS3 |  |  |
|  |  | PECAM1 | MMP3 |  |  |
|  |  | PECAM1 | IGF1 |  |  |
|  |  | PECAM1 | CD14 |  |  |
|  |  | PECAM1 | CXCL8 |  |  |
|  |  | PECAM1 | F2 |  |  |
|  |  | PECAM1 | PLG |  |  |
|  |  | PECAM1 | LEP |  |  |
|  |  | PECAM1 | ANGPT2 |  |  |
|  |  | PECAM1 | MMP1 |  |  |
|  |  | PECAM1 | CD28 |  |  |
|  |  | PECAM1 | CSF1 |  |  |
|  |  | PECAM1 | GP1BA |  |  |
|  |  | PECAM1 | SELE |  |  |
|  |  | PECAM1 | CD86 |  |  |
|  |  | PECAM1 | SMAD3 |  |  |
|  |  | PECAM1 | F3 |  |  |
|  |  | PECAM1 | TNNI3 |  |  |
|  |  | PECAM1 | IL17A |  |  |
|  |  | PECAM1 | CX3CR1 |  |  |
|  |  | PECAM1 | CD163 |  |  |
|  |  | PECAM1 | DPP4 |  |  |
|  |  | PECAM1 | PTGS2 |  |  |
|  |  | PECAM1 | CTGF |  |  |
|  |  | PECAM1 | BGLAP |  |  |
|  |  | PECAM1 | S100A4 |  |  |
|  |  | PECAM1 | S100A9 |  |  |
|  |  | PECAM1 | CD40LG |  |  |
|  |  | PECAM1 | ENTPD1 |  |  |
|  |  | PECAM1 | MMP9 |  |  |
|  |  | PECAM1 | PLAU |  |  |
|  |  | PECAM1 | TLR4 |  |  |
|  |  | PECAM1 | THBD |  |  |
|  |  | PECAM1 | EDN1 |  |  |
|  |  | PECAM1 | TEK |  |  |
|  |  | PECAM1 | INS |  |  |
|  |  | PECAM1 | ITGB2 |  |  |
|  |  | PECAM1 | IL6 |  |  |
|  |  | PECAM1 | CXCR4 |  |  |
|  |  | PECAM1 | TNF |  |  |
|  |  | PECAM1 | TREML1 |  |  |
|  |  | PECAM1 | IL10 |  |  |
|  |  | PECAM1 | SEMA4D |  |  |
|  |  | PECAM1 | ANGPT1 |  |  |
|  |  | PECAM1 | HIF1A |  |  |
|  |  | PECAM1 | CD63 |  |  |
|  |  | PECAM1 | PGF |  |  |
|  |  | PECAM1 | ITGB3 |  |  |
|  |  | PECAM1 | CCL3 |  |  |
|  |  | PECAM1 | FGF21 |  |  |
|  |  | PECAM1 | CCL5 |  |  |
|  |  | PECAM1 | VEGFA |  |  |
|  |  | PF4 | CX3CL1 |  |  |
|  |  | PF4 | TIMP1 |  |  |
|  |  | PF4 | SERPINE1 |  |  |
|  |  | PF4 | MPO |  |  |
|  |  | PF4 | CCL2 |  |  |
|  |  | PF4 | SELPLG |  |  |
|  |  | PF4 | HRG |  |  |
|  |  | PF4 | CRP |  |  |
|  |  | PF4 | VWF |  |  |
|  |  | PF4 | ITGA2B |  |  |
|  |  | PF4 | IL1A |  |  |
|  |  | PF4 | IL1B |  |  |
|  |  | PF4 | SELP |  |  |
|  |  | PF4 | ICAM1 |  |  |
|  |  | PF4 | ITIH4 |  |  |
|  |  | PF4 | FCGR2A |  |  |
|  |  | PF4 | CXCL13 |  |  |
|  |  | PF4 | CXCL16 |  |  |
|  |  | PF4 | VCAM1 |  |  |
|  |  | PF4 | PPBP |  |  |
|  |  | PF4 | TLR4 |  |  |
|  |  | PF4 | F10 |  |  |
|  |  | PF4 | F7 |  |  |
|  |  | PF4 | SELE |  |  |
|  |  | PF4 | ANGPT1 |  |  |
|  |  | PF4 | PECAM1 |  |  |
|  |  | PF4 | MMP9 |  |  |
|  |  | PF4 | IL10 |  |  |
|  |  | PF4 | TREML1 |  |  |
|  |  | PF4 | ITGB3 |  |  |
|  |  | PF4 | SERPINF2 |  |  |
|  |  | PF4 | PLG |  |  |
|  |  | PF4 | TNF |  |  |
|  |  | PF4 | IL6 |  |  |
|  |  | PF4 | CCL11 |  |  |
|  |  | PF4 | CD40LG |  |  |
|  |  | PF4 | CCL3 |  |  |
|  |  | PF4 | F2 |  |  |
|  |  | PF4 | THBD |  |  |
|  |  | PF4 | F3 |  |  |
|  |  | PF4 | GP1BA |  |  |
|  |  | PF4 | CX3CR1 |  |  |
|  |  | PF4 | VEGFA |  |  |
|  |  | PF4 | CXCL8 |  |  |
|  |  | PF4 | CXCR4 |  |  |
|  |  | PF4 | SERPINC1 | |  |
|  |  | PF4 | CCL5 |  |  |
|  |  | PGF | TIMP1 |  |  |
|  |  | PGF | MMP2 |  |  |
|  |  | PGF | TGFB1 |  |  |
|  |  | PGF | HGF |  |  |
|  |  | PGF | SERPINE1 |  |  |
|  |  | PGF | CCL2 |  |  |
|  |  | PGF | HRG |  |  |
|  |  | PGF | EPO |  |  |
|  |  | PGF | CRP |  |  |
|  |  | PGF | VWF |  |  |
|  |  | PGF | IL1B |  |  |
|  |  | PGF | ICAM1 |  |  |
|  |  | PGF | REN |  |  |
|  |  | PGF | IGFBP1 |  |  |
|  |  | PGF | VCAM1 |  |  |
|  |  | PGF | NOS3 |  |  |
|  |  | PGF | IGF1 |  |  |
|  |  | PGF | CXCL8 |  |  |
|  |  | PGF | PLG |  |  |
|  |  | PGF | LEP |  |  |
|  |  | PGF | ANGPT2 |  |  |
|  |  | PGF | CSF1 |  |  |
|  |  | PGF | PAPPA |  |  |
|  |  | PGF | SELE |  |  |
|  |  | PGF | F3 |  |  |
|  |  | PGF | MMP9 |  |  |
|  |  | PGF | EDN1 |  |  |
|  |  | PGF | TEK |  |  |
|  |  | PGF | IL6 |  |  |
|  |  | PGF | CXCR4 |  |  |
|  |  | PGF | TNF |  |  |
|  |  | PGF | IL10 |  |  |
|  |  | PGF | SEMA4D |  |  |
|  |  | PGF | ANGPT1 |  |  |
|  |  | PGF | HIF1A |  |  |
|  |  | PGF | FGF21 |  |  |
|  |  | PGF | PECAM1 |  |  |
|  |  | PGF | VEGFA |  |  |
|  |  | PIK3C2A | CRP |  |  |
|  |  | PIK3C2A | ACE |  |  |
|  |  | PIK3C2A | NPPB |  |  |
|  |  | PIK3C2A | F3 |  |  |
|  |  | PIK3C2A | INS |  |  |
|  |  | PIK3C2A | F2 |  |  |
|  |  | PIK3C2A | TNNI3 |  |  |
|  |  | PIK3C2A | MB |  |  |
|  |  | PIK3C2A | ITGB3 |  |  |
|  |  | PLA2G2A | APOB |  |  |
|  |  | PLA2G2A | APOA1 |  |  |
|  |  | PLA2G2A | CRP |  |  |
|  |  | PLA2G2A | IL1B |  |  |
|  |  | PLA2G2A | KNG1 |  |  |
|  |  | PLA2G2A | PLB1 |  |  |
|  |  | PLA2G2A | PTGS2 |  |  |
|  |  | PLA2G2A | CYP2C19 |  |  |
|  |  | PLA2G2A | F10 |  |  |
|  |  | PLA2G2A | IL6 |  |  |
|  |  | PLA2G2A | ITGB3 |  |  |
|  |  | PLA2G2A | TNF |  |  |
|  |  | PLAT | CPB2 |  |  |
|  |  | PLAT | TIMP1 |  |  |
|  |  | PLAT | MMP2 |  |  |
|  |  | PLAT | CTGF |  |  |
|  |  | PLAT | PLAU |  |  |
|  |  | PLAT | F7 |  |  |
|  |  | PLAT | CD163 |  |  |
|  |  | PLAT | CCL2 |  |  |
|  |  | PLAT | REN |  |  |
|  |  | PLAT | CXCL8 |  |  |
|  |  | PLAT | NOS3 |  |  |
|  |  | PLAT | IL1B |  |  |
|  |  | PLAT | ICAM1 |  |  |
|  |  | PLAT | PPBP |  |  |
|  |  | PLAT | VCAM1 |  |  |
|  |  | PLAT | SERPINA1 | |  |
|  |  | PLAT | SELE |  |  |
|  |  | PLAT | INS |  |  |
|  |  | PLAT | MMP1 |  |  |
|  |  | PLAT | BDNF |  |  |
|  |  | PLAT | EDN1 |  |  |
|  |  | PLAT | VEGFA |  |  |
|  |  | PLAT | TNF |  |  |
|  |  | PLAT | F10 |  |  |
|  |  | PLAT | ITGA2B |  |  |
|  |  | PLAT | SELP |  |  |
|  |  | PLAT | MMP3 |  |  |
|  |  | PLAT | CRP |  |  |
|  |  | PLAT | ACE |  |  |
|  |  | PLAT | KNG1 |  |  |
|  |  | PLAT | IL6 |  |  |
|  |  | PLAT | FGA |  |  |
|  |  | PLAT | SERPING1 | |  |
|  |  | PLAT | SERPINA5 | |  |
|  |  | PLAT | THBD |  |  |
|  |  | PLAT | MMP9 |  |  |
|  |  | PLAT | HRG |  |  |
|  |  | PLAT | VWF |  |  |
|  |  | PLAT | F3 |  |  |
|  |  | PLAT | SERPINC1 | |  |
|  |  | PLAT | ITGB2 |  |  |
|  |  | PLAT | PLG |  |  |
|  |  | PLAT | F2 |  |  |
|  |  | PLAT | SERPINF2 |  |  |
|  |  | PLAT | SERPINE1 |  |  |
|  |  | PLAU | CPB2 |  |  |
|  |  | PLAU | TIMP1 |  |  |
|  |  | PLAU | MMP2 |  |  |
|  |  | PLAU | PLAT |  |  |
|  |  | PLAU | TGFB1 |  |  |
|  |  | PLAU | HGF |  |  |
|  |  | PLAU | SERPINE1 |  |  |
|  |  | PLAU | CCL2 |  |  |
|  |  | PLAU | HRG |  |  |
|  |  | PLAU | MMP8 |  |  |
|  |  | PLAU | VWF |  |  |
|  |  | PLAU | IL1B |  |  |
|  |  | PLAU | ICAM1 |  |  |
|  |  | PLAU | VCAM1 |  |  |
|  |  | PLAU | MMP3 |  |  |
|  |  | PLAU | IGF1 |  |  |
|  |  | PLAU | FGA |  |  |
|  |  | PLAU | CXCL8 |  |  |
|  |  | PLAU | F2 |  |  |
|  |  | PLAU | PLG |  |  |
|  |  | PLAU | SERPINF2 |  |  |
|  |  | PLAU | MMP1 |  |  |
|  |  | PLAU | CSF1 |  |  |
|  |  | PLAU | SERPINA5 | |  |
|  |  | PLAU | F3 |  |  |
|  |  | PLAU | PTGS2 |  |  |
|  |  | PLAU | SERPINC1 | |  |
|  |  | PLAU | CTGF |  |  |
|  |  | PLAU | MMP9 |  |  |
|  |  | PLAU | PECAM1 |  |  |
|  |  | PLAU | EDN1 |  |  |
|  |  | PLAU | CXCR4 |  |  |
|  |  | PLAU | SERP1 |  |  |
|  |  | PLAU | HIF1A |  |  |
|  |  | PLAU | TNF |  |  |
|  |  | PLAU | THBD |  |  |
|  |  | PLAU | IL6 |  |  |
|  |  | PLAU | VEGFA |  |  |
|  |  | PLAU | ITGB2 |  |  |
|  |  | PLAU | ITGB3 |  |  |
|  |  | PLB1 | CYP2C19 |  |  |
|  |  | PLB1 | PTGS2 |  |  |
|  |  | PLB1 | PLA2G2A |  |  |
|  |  | PLG | CPB2 |  |  |
|  |  | PLG | CHGA |  |  |
|  |  | PLG | TIMP1 |  |  |
|  |  | PLG | MMP2 |  |  |
|  |  | PLG | PLAT |  |  |
|  |  | PLG | RETN |  |  |
|  |  | PLG | TGFB1 |  |  |
|  |  | PLG | PON1 |  |  |
|  |  | PLG | SERPINE1 |  |  |
|  |  | PLG | MPO |  |  |
|  |  | PLG | CCL2 |  |  |
|  |  | PLG | IFNG |  |  |
|  |  | PLG | HRG |  |  |
|  |  | PLG | APOB |  |  |
|  |  | PLG | MMP8 |  |  |
|  |  | PLG | APOA1 |  |  |
|  |  | PLG | EPO |  |  |
|  |  | PLG | CRP |  |  |
|  |  | PLG | VWF |  |  |
|  |  | PLG | ITGA2B |  |  |
|  |  | PLG | IL1A |  |  |
|  |  | PLG | IL1B |  |  |
|  |  | PLG | SELP |  |  |
|  |  | PLG | CP |  |  |
|  |  | PLG | ICAM1 |  |  |
|  |  | PLG | KNG1 |  |  |
|  |  | PLG | ITIH4 |  |  |
|  |  | PLG | REN |  |  |
|  |  | PLG | SERPING1 | |  |
|  |  | PLG | IL18 |  |  |
|  |  | PLG | PPARG |  |  |
|  |  | PLG | ACE |  |  |
|  |  | PLG | VCAM1 |  |  |
|  |  | PLG | PTX3 |  |  |
|  |  | PLG | PPBP |  |  |
|  |  | PLG | PF4 |  |  |
|  |  | PLG | NOS3 |  |  |
|  |  | PLG | MMP3 |  |  |
|  |  | PLG | IGF1 |  |  |
|  |  | PLG | FGA |  |  |
|  |  | PLG | CXCL8 |  |  |
|  |  | PLG | F2 |  |  |
|  |  | PLG | BSG |  |  |
|  |  | PLG | PGF |  |  |
|  |  | PLG | CD40LG |  |  |
|  |  | PLG | AGTR1 |  |  |
|  |  | PLG | ANGPT2 |  |  |
|  |  | PLG | CXCR4 |  |  |
|  |  | PLG | MBL2 |  |  |
|  |  | PLG | SMAD3 |  |  |
|  |  | PLG | TLR4 |  |  |
|  |  | PLG | F7 |  |  |
|  |  | PLG | HIF1A |  |  |
|  |  | PLG | PECAM1 |  |  |
|  |  | PLG | F10 |  |  |
|  |  | PLG | CTGF |  |  |
|  |  | PLG | PTGS2 |  |  |
|  |  | PLG | IL10 |  |  |
|  |  | PLG | LEP |  |  |
|  |  | PLG | SERPINA5 | |  |
|  |  | PLG | SELE |  |  |
|  |  | PLG | EDN1 |  |  |
|  |  | PLG | AGT |  |  |
|  |  | PLG | HSPD1 |  |  |
|  |  | PLG | TNF |  |  |
|  |  | PLG | VEGFA |  |  |
|  |  | PLG | IL6 |  |  |
|  |  | PLG | INS |  |  |
|  |  | PLG | SERPINA1 | |  |
|  |  | PLG | S100A4 |  |  |
|  |  | PLG | THBD |  |  |
|  |  | PLG | F3 |  |  |
|  |  | PLG | ITGB2 |  |  |
|  |  | PLG | PLAU |  |  |
|  |  | PLG | TEK |  |  |
|  |  | PLG | ANGPT1 |  |  |
|  |  | PLG | DPP4 |  |  |
|  |  | PLG | MMP1 |  |  |
|  |  | PLG | BDNF |  |  |
|  |  | PLG | SERPINC1 | |  |
|  |  | PLG | MMP9 |  |  |
|  |  | PLG | SERPINF2 |  |  |
|  |  | PON1 | CETP |  |  |
|  |  | PON1 | SERPINE1 |  |  |
|  |  | PON1 | VCAM1 |  |  |
|  |  | PON1 | PLG |  |  |
|  |  | PON1 | CCL2 |  |  |
|  |  | PON1 | TNF |  |  |
|  |  | PON1 | PPARG |  |  |
|  |  | PON1 | SERPINA1 | |  |
|  |  | PON1 | OLR1 |  |  |
|  |  | PON1 | IL6 |  |  |
|  |  | PON1 | ACE |  |  |
|  |  | PON1 | INS |  |  |
|  |  | PON1 | NOS3 |  |  |
|  |  | PON1 | LEP |  |  |
|  |  | PON1 | CP |  |  |
|  |  | PON1 | CYP2C19 |  |  |
|  |  | PON1 | CRP |  |  |
|  |  | PON1 | VIMP |  |  |
|  |  | PON1 | SAA4 |  |  |
|  |  | PON1 | MPO |  |  |
|  |  | PON1 | APOB |  |  |
|  |  | PON1 | APOA1 |  |  |
|  |  | PPARG | TNFRSF1A | |  |
|  |  | PPARG | CETP |  |  |
|  |  | PPARG | SIRT1 |  |  |
|  |  | PPARG | HMOX1 |  |  |
|  |  | PPARG | TIMP1 |  |  |
|  |  | PPARG | MMP2 |  |  |
|  |  | PPARG | RETN |  |  |
|  |  | PPARG | TGFB1 |  |  |
|  |  | PPARG | PON1 |  |  |
|  |  | PPARG | HGF |  |  |
|  |  | PPARG | NAMPT |  |  |
|  |  | PPARG | SERPINE1 |  |  |
|  |  | PPARG | MPO |  |  |
|  |  | PPARG | CCL2 |  |  |
|  |  | PPARG | NFKB1 |  |  |
|  |  | PPARG | IL2 |  |  |
|  |  | PPARG | IFNG |  |  |
|  |  | PPARG | APOB |  |  |
|  |  | PPARG | APOA1 |  |  |
|  |  | PPARG | AHR |  |  |
|  |  | PPARG | LGALS3 |  |  |
|  |  | PPARG | CRP |  |  |
|  |  | PPARG | TLR2 |  |  |
|  |  | PPARG | IL1A |  |  |
|  |  | PPARG | IL1B |  |  |
|  |  | PPARG | ICAM1 |  |  |
|  |  | PPARG | PPARGC1A | |  |
|  |  | PPARG | REN |  |  |
|  |  | PPARG | IL18 |  |  |
|  |  | PPARG | CCL3 |  |  |
|  |  | PPARG | CXCR4 |  |  |
|  |  | PPARG | LCN2 |  |  |
|  |  | PPARG | PLG |  |  |
|  |  | PPARG | CSF1 |  |  |
|  |  | PPARG | CD163 |  |  |
|  |  | PPARG | ADRB2 |  |  |
|  |  | PPARG | MYD88 |  |  |
|  |  | PPARG | CCL5 |  |  |
|  |  | PPARG | SELE |  |  |
|  |  | PPARG | OLR1 |  |  |
|  |  | PPARG | BDNF |  |  |
|  |  | PPARG | IL17A |  |  |
|  |  | PPARG | PECAM1 |  |  |
|  |  | PPARG | BRCA1 |  |  |
|  |  | PPARG | AGT |  |  |
|  |  | PPARG | CTGF |  |  |
|  |  | PPARG | CYCS |  |  |
|  |  | PPARG | HMGCR |  |  |
|  |  | PPARG | TNFRSF11B | |  |
|  |  | PPARG | DPP4 |  |  |
|  |  | PPARG | FABP3 |  |  |
|  |  | PPARG | VCAM1 |  |  |
|  |  | PPARG | CXCL8 |  |  |
|  |  | PPARG | MMP9 |  |  |
|  |  | PPARG | EDN1 |  |  |
|  |  | PPARG | VEGFA |  |  |
|  |  | PPARG | TLR4 |  |  |
|  |  | PPARG | NOS3 |  |  |
|  |  | PPARG | IL10 |  |  |
|  |  | PPARG | FGF21 |  |  |
|  |  | PPARG | BGLAP |  |  |
|  |  | PPARG | CNR1 |  |  |
|  |  | PPARG | HIF1A |  |  |
|  |  | PPARG | AGTR1 |  |  |
|  |  | PPARG | IGF1 |  |  |
|  |  | PPARG | ACE |  |  |
|  |  | PPARG | IL6 |  |  |
|  |  | PPARG | SMAD3 |  |  |
|  |  | PPARG | PTGS2 |  |  |
|  |  | PPARG | INS |  |  |
|  |  | PPARG | TNF |  |  |
|  |  | PPARG | LEP |  |  |
|  |  | PPARGC1A | SIRT1 |  |  |
|  |  | PPARGC1A | HMOX1 |  |  |
|  |  | PPARGC1A | RETN |  |  |
|  |  | PPARGC1A | NAMPT |  |  |
|  |  | PPARGC1A | CCL2 |  |  |
|  |  | PPARGC1A | NFKB1 |  |  |
|  |  | PPARGC1A | APOB |  |  |
|  |  | PPARGC1A | APOA1 |  |  |
|  |  | PPARGC1A | CRP |  |  |
|  |  | PPARGC1A | IL1B |  |  |
|  |  | PPARGC1A | NPPB |  |  |
|  |  | PPARGC1A | TLR4 |  |  |
|  |  | PPARGC1A | ACE |  |  |
|  |  | PPARGC1A | ADRB2 |  |  |
|  |  | PPARGC1A | HMGCR |  |  |
|  |  | PPARGC1A | MB |  |  |
|  |  | PPARGC1A | FABP3 |  |  |
|  |  | PPARGC1A | VEGFA |  |  |
|  |  | PPARGC1A | BDNF |  |  |
|  |  | PPARGC1A | IL6 |  |  |
|  |  | PPARGC1A | TNF |  |  |
|  |  | PPARGC1A | NOS3 |  |  |
|  |  | PPARGC1A | IGF1 |  |  |
|  |  | PPARGC1A | PARP1 |  |  |
|  |  | PPARGC1A | PARK2 |  |  |
|  |  | PPARGC1A | CYCS |  |  |
|  |  | PPARGC1A | HIF1A |  |  |
|  |  | PPARGC1A | FGF21 |  |  |
|  |  | PPARGC1A | LEP |  |  |
|  |  | PPARGC1A | INS |  |  |
|  |  | PPARGC1A | PPARG |  |  |
|  |  | PPBP | CX3CL1 |  |  |
|  |  | PPBP | GZMB |  |  |
|  |  | PPBP | PLAT |  |  |
|  |  | PPBP | SERPINE1 |  |  |
|  |  | PPBP | MPO |  |  |
|  |  | PPBP | CCL2 |  |  |
|  |  | PPBP | IL2 |  |  |
|  |  | PPBP | IFNG |  |  |
|  |  | PPBP | IL5 |  |  |
|  |  | PPBP | TLR2 |  |  |
|  |  | PPBP | VWF |  |  |
|  |  | PPBP | ITGA2B |  |  |
|  |  | PPBP | IL1B |  |  |
|  |  | PPBP | SELP |  |  |
|  |  | PPBP | IL7 |  |  |
|  |  | PPBP | IL18 |  |  |
|  |  | PPBP | CXCL13 |  |  |
|  |  | PPBP | CXCL16 |  |  |
|  |  | PPBP | VCAM1 |  |  |
|  |  | PPBP | ITGB3 |  |  |
|  |  | PPBP | S100A9 |  |  |
|  |  | PPBP | CCL11 |  |  |
|  |  | PPBP | TLR4 |  |  |
|  |  | PPBP | F7 |  |  |
|  |  | PPBP | MMP9 |  |  |
|  |  | PPBP | GP1BA |  |  |
|  |  | PPBP | IL17A |  |  |
|  |  | PPBP | THBD |  |  |
|  |  | PPBP | VEGFA |  |  |
|  |  | PPBP | TNF |  |  |
|  |  | PPBP | CXCL8 |  |  |
|  |  | PPBP | CD40LG |  |  |
|  |  | PPBP | TREML1 |  |  |
|  |  | PPBP | F3 |  |  |
|  |  | PPBP | SERPINF2 |  |  |
|  |  | PPBP | CCL3 |  |  |
|  |  | PPBP | PLG |  |  |
|  |  | PPBP | F2 |  |  |
|  |  | PPBP | IL10 |  |  |
|  |  | PPBP | IL6 |  |  |
|  |  | PPBP | CX3CR1 |  |  |
|  |  | PPBP | CCL5 |  |  |
|  |  | PPBP | SERPINC1 | |  |
|  |  | PPBP | CXCR4 |  |  |
|  |  | PPBP | PF4 |  |  |
|  |  | PRG2 | MPO |  |  |
|  |  | PRG2 | IL5 |  |  |
|  |  | PRG2 | CRP |  |  |
|  |  | PRG2 | CCL11 |  |  |
|  |  | PRG2 | RNASE3 |  |  |
|  |  | PRG2 | TNNI3 |  |  |
|  |  | PRG2 | AGT |  |  |
|  |  | PRG2 | PAPPA |  |  |
|  |  | PTGS2 | CX3CL1 |  |  |
|  |  | PTGS2 | TNFRSF1A | |  |
|  |  | PTGS2 | SIRT1 |  |  |
|  |  | PTGS2 | MIF |  |  |
|  |  | PTGS2 | HMOX1 |  |  |
|  |  | PTGS2 | TIMP1 |  |  |
|  |  | PTGS2 | MMP2 |  |  |
|  |  | PTGS2 | TGFB1 |  |  |
|  |  | PTGS2 | HGF |  |  |
|  |  | PTGS2 | NAMPT |  |  |
|  |  | PTGS2 | SERPINE1 |  |  |
|  |  | PTGS2 | MPO |  |  |
|  |  | PTGS2 | CCL2 |  |  |
|  |  | PTGS2 | NFKB1 |  |  |
|  |  | PTGS2 | IL2 |  |  |
|  |  | PTGS2 | IFNG |  |  |
|  |  | PTGS2 | IL5 |  |  |
|  |  | PTGS2 | MMP8 |  |  |
|  |  | PTGS2 | AHR |  |  |
|  |  | PTGS2 | CRP |  |  |
|  |  | PTGS2 | IL1RN |  |  |
|  |  | PTGS2 | TLR2 |  |  |
|  |  | PTGS2 | CYBA |  |  |
|  |  | PTGS2 | IL37 |  |  |
|  |  | PTGS2 | IL1A |  |  |
|  |  | PTGS2 | IL1B |  |  |
|  |  | PTGS2 | SELP |  |  |
|  |  | PTGS2 | ICAM1 |  |  |
|  |  | PTGS2 | KNG1 |  |  |
|  |  | PTGS2 | REN |  |  |
|  |  | PTGS2 | IFNA1 |  |  |
|  |  | PTGS2 | IL18 |  |  |
|  |  | PTGS2 | PPARG |  |  |
|  |  | PTGS2 | ACE |  |  |
|  |  | PTGS2 | VCAM1 |  |  |
|  |  | PTGS2 | PTX3 |  |  |
|  |  | PTGS2 | TNFRSF11B | |  |
|  |  | PTGS2 | NOS3 |  |  |
|  |  | PTGS2 | MMP3 |  |  |
|  |  | PTGS2 | IGF1 |  |  |
|  |  | PTGS2 | HSPA4 |  |  |
|  |  | PTGS2 | CXCL8 |  |  |
|  |  | PTGS2 | CYCS |  |  |
|  |  | PTGS2 | PLG |  |  |
|  |  | PTGS2 | LEP |  |  |
|  |  | PTGS2 | MMP1 |  |  |
|  |  | PTGS2 | CSF1 |  |  |
|  |  | PTGS2 | PLB1 |  |  |
|  |  | PTGS2 | SELE |  |  |
|  |  | PTGS2 | CD86 |  |  |
|  |  | PTGS2 | SMAD3 |  |  |
|  |  | PTGS2 | F3 |  |  |
|  |  | PTGS2 | IL17A |  |  |
|  |  | PTGS2 | CD163 |  |  |
|  |  | PTGS2 | AGT |  |  |
|  |  | PTGS2 | CD40LG |  |  |
|  |  | PTGS2 | ITGB3 |  |  |
|  |  | PTGS2 | BGLAP |  |  |
|  |  | PTGS2 | FASLG |  |  |
|  |  | PTGS2 | AGER |  |  |
|  |  | PTGS2 | S100A8 |  |  |
|  |  | PTGS2 | ADAMTS4 | |  |
|  |  | PTGS2 | PLAU |  |  |
|  |  | PTGS2 | CXCR4 |  |  |
|  |  | PTGS2 | CTGF |  |  |
|  |  | PTGS2 | PECAM1 |  |  |
|  |  | PTGS2 | BDNF |  |  |
|  |  | PTGS2 | CCL3 |  |  |
|  |  | PTGS2 | CCL5 |  |  |
|  |  | PTGS2 | MYD88 |  |  |
|  |  | PTGS2 | INS |  |  |
|  |  | PTGS2 | EDN1 |  |  |
|  |  | PTGS2 | CNR1 |  |  |
|  |  | PTGS2 | AGTR1 |  |  |
|  |  | PTGS2 | HIF1A |  |  |
|  |  | PTGS2 | IL10 |  |  |
|  |  | PTGS2 | MMP9 |  |  |
|  |  | PTGS2 | TLR4 |  |  |
|  |  | PTGS2 | PLA2G2A |  |  |
|  |  | PTGS2 | TNF |  |  |
|  |  | PTGS2 | VEGFA |  |  |
|  |  | PTGS2 | IL6 |  |  |
|  |  | PTGS2 | CYP2C19 |  |  |
|  |  | PTX3 | TIMP1 |  |  |
|  |  | PTX3 | SERPINE1 |  |  |
|  |  | PTX3 | MPO |  |  |
|  |  | PTX3 | CCL2 |  |  |
|  |  | PTX3 | GDF15 |  |  |
|  |  | PTX3 | LGALS3 |  |  |
|  |  | PTX3 | CRP |  |  |
|  |  | PTX3 | CHI3L1 |  |  |
|  |  | PTX3 | TLR2 |  |  |
|  |  | PTX3 | IL1B |  |  |
|  |  | PTX3 | SELP |  |  |
|  |  | PTX3 | ICAM1 |  |  |
|  |  | PTX3 | ITIH4 |  |  |
|  |  | PTX3 | FCGR2A |  |  |
|  |  | PTX3 | IGFBP1 |  |  |
|  |  | PTX3 | VCAM1 |  |  |
|  |  | PTX3 | MMP1 |  |  |
|  |  | PTX3 | TLR4 |  |  |
|  |  | PTX3 | MMP3 |  |  |
|  |  | PTX3 | MMP9 |  |  |
|  |  | PTX3 | MYD88 |  |  |
|  |  | PTX3 | F3 |  |  |
|  |  | PTX3 | EDN1 |  |  |
|  |  | PTX3 | PTGS2 |  |  |
|  |  | PTX3 | IL10 |  |  |
|  |  | PTX3 | TNF |  |  |
|  |  | PTX3 | CXCL8 |  |  |
|  |  | PTX3 | VEGFA |  |  |
|  |  | PTX3 | C1S |  |  |
|  |  | PTX3 | PLG |  |  |
|  |  | PTX3 | IL6 |  |  |
|  |  | PTX3 | MBL2 |  |  |
|  |  | PYGB | RIPK3 |  |  |
|  |  | PYGB | THBD |  |  |
|  |  | PYGB | TNNT2 |  |  |
|  |  | PYGB | MB |  |  |
|  |  | PYGB | TNNI3 |  |  |
|  |  | PYGB | FABP3 |  |  |
|  |  | RAB3IL1 | GZMB |  |  |
|  |  | RAB3IL1 | PARP1 |  |  |
|  |  | REN | SIRT1 |  |  |
|  |  | REN | HMOX1 |  |  |
|  |  | REN | TIMP1 |  |  |
|  |  | REN | MMP2 |  |  |
|  |  | REN | PLAT |  |  |
|  |  | REN | RETN |  |  |
|  |  | REN | TGFB1 |  |  |
|  |  | REN | SERPINE1 |  |  |
|  |  | REN | MPO |  |  |
|  |  | REN | CCL2 |  |  |
|  |  | REN | APOB |  |  |
|  |  | REN | APOA1 |  |  |
|  |  | REN | EPO |  |  |
|  |  | REN | CRP |  |  |
|  |  | REN | TLR2 |  |  |
|  |  | REN | VWF |  |  |
|  |  | REN | CYBA |  |  |
|  |  | REN | IL1B |  |  |
|  |  | REN | SELP |  |  |
|  |  | REN | ICAM1 |  |  |
|  |  | REN | KNG1 |  |  |
|  |  | REN | MMP3 |  |  |
|  |  | REN | PGF |  |  |
|  |  | REN | TNNI3 |  |  |
|  |  | REN | CXCL8 |  |  |
|  |  | REN | SERPINC1 | |  |
|  |  | REN | SMAD3 |  |  |
|  |  | REN | TLR4 |  |  |
|  |  | REN | SELE |  |  |
|  |  | REN | F2 |  |  |
|  |  | REN | F3 |  |  |
|  |  | REN | HIF1A |  |  |
|  |  | REN | SERPINA1 | |  |
|  |  | REN | IL10 |  |  |
|  |  | REN | ADRB2 |  |  |
|  |  | REN | PCSK9 |  |  |
|  |  | REN | MMP9 |  |  |
|  |  | REN | VCAM1 |  |  |
|  |  | REN | LCN2 |  |  |
|  |  | REN | DPP4 |  |  |
|  |  | REN | PPARG |  |  |
|  |  | REN | CTGF |  |  |
|  |  | REN | PLG |  |  |
|  |  | REN | PTGS2 |  |  |
|  |  | REN | ADM |  |  |
|  |  | REN | CST3 |  |  |
|  |  | REN | VEGFA |  |  |
|  |  | REN | TNF |  |  |
|  |  | REN | NPPB |  |  |
|  |  | REN | NOS3 |  |  |
|  |  | REN | LEP |  |  |
|  |  | REN | IGF1 |  |  |
|  |  | REN | IL6 |  |  |
|  |  | REN | INS |  |  |
|  |  | REN | AVP |  |  |
|  |  | REN | EDN1 |  |  |
|  |  | REN | AGTR1 |  |  |
|  |  | REN | ACE |  |  |
|  |  | REN | AGT |  |  |
|  |  | RETN | IL5 |  |  |
|  |  | RETN | S100A8 |  |  |
|  |  | RETN | IL1A |  |  |
|  |  | RETN | ACE |  |  |
|  |  | RETN | HGF |  |  |
|  |  | RETN | S100A9 |  |  |
|  |  | RETN | IFNG |  |  |
|  |  | RETN | DPP4 |  |  |
|  |  | RETN | RNASE3 |  |  |
|  |  | RETN | IL1RN |  |  |
|  |  | RETN | CCL5 |  |  |
|  |  | RETN | BGLAP |  |  |
|  |  | RETN | TLR2 |  |  |
|  |  | RETN | APOA1 |  |  |
|  |  | RETN | NOS3 |  |  |
|  |  | RETN | APOB |  |  |
|  |  | RETN | SELE |  |  |
|  |  | RETN | IL17A |  |  |
|  |  | RETN | REN |  |  |
|  |  | RETN | EDN1 |  |  |
|  |  | RETN | CHI3L1 |  |  |
|  |  | RETN | MMP9 |  |  |
|  |  | RETN | PCSK9 |  |  |
|  |  | RETN | VEGFA |  |  |
|  |  | RETN | IL18 |  |  |
|  |  | RETN | PPARGC1A | |  |
|  |  | RETN | ICAM1 |  |  |
|  |  | RETN | FGF21 |  |  |
|  |  | RETN | PLG |  |  |
|  |  | RETN | CCL3 |  |  |
|  |  | RETN | VCAM1 |  |  |
|  |  | RETN | IGF1 |  |  |
|  |  | RETN | LCN2 |  |  |
|  |  | RETN | MPO |  |  |
|  |  | RETN | CXCL8 |  |  |
|  |  | RETN | AGT |  |  |
|  |  | RETN | IL10 |  |  |
|  |  | RETN | IL1B |  |  |
|  |  | RETN | CCL2 |  |  |
|  |  | RETN | PPARG |  |  |
|  |  | RETN | SERPINA12 | |  |
|  |  | RETN | SERPINE1 |  |  |
|  |  | RETN | CRP |  |  |
|  |  | RETN | IL6 |  |  |
|  |  | RETN | TNF |  |  |
|  |  | RETN | INS |  |  |
|  |  | RETN | NAMPT |  |  |
|  |  | RETN | LEP |  |  |
|  |  | RETN | TLR4 |  |  |
|  |  | RIPK1 | TNFRSF1A | |  |
|  |  | RIPK1 | RIPK3 |  |  |
|  |  | RIPK1 | NFKB1 |  |  |
|  |  | RIPK1 | PARK2 |  |  |
|  |  | RIPK1 | IL18 |  |  |
|  |  | RIPK1 | IL6 |  |  |
|  |  | RIPK1 | PARP1 |  |  |
|  |  | RIPK1 | TLR2 |  |  |
|  |  | RIPK1 | CYCS |  |  |
|  |  | RIPK1 | CD14 |  |  |
|  |  | RIPK1 | IL1B |  |  |
|  |  | RIPK1 | MYD88 |  |  |
|  |  | RIPK1 | MLKL |  |  |
|  |  | RIPK1 | TLR4 |  |  |
|  |  | RIPK1 | TNFRSF1B | |  |
|  |  | RIPK1 | RPS27A |  |  |
|  |  | RIPK1 | FASLG |  |  |
|  |  | RIPK1 | UBC |  |  |
|  |  | RIPK1 | FAS |  |  |
|  |  | RIPK1 | TNF |  |  |
|  |  | RIPK3 | TNFRSF1A | |  |
|  |  | RIPK3 | IL6 |  |  |
|  |  | RIPK3 | PARP1 |  |  |
|  |  | RIPK3 | TLR2 |  |  |
|  |  | RIPK3 | CYCS |  |  |
|  |  | RIPK3 | IL18 |  |  |
|  |  | RIPK3 | MYD88 |  |  |
|  |  | RIPK3 | IL1B |  |  |
|  |  | RIPK3 | CD14 |  |  |
|  |  | RIPK3 | NFKB1 |  |  |
|  |  | RIPK3 | TNF |  |  |
|  |  | RIPK3 | PYGB |  |  |
|  |  | RIPK3 | TLR4 |  |  |
|  |  | RIPK3 | UBC |  |  |
|  |  | RIPK3 | RPS27A |  |  |
|  |  | RIPK3 | FAS |  |  |
|  |  | RIPK3 | FASLG |  |  |
|  |  | RIPK3 | MLKL |  |  |
|  |  | RIPK3 | RIPK1 |  |  |
|  |  | RNASE3 | RETN |  |  |
|  |  | RNASE3 | MPO |  |  |
|  |  | RNASE3 | IL2 |  |  |
|  |  | RNASE3 | IL5 |  |  |
|  |  | RNASE3 | CRP |  |  |
|  |  | RNASE3 | IL1B |  |  |
|  |  | RNASE3 | ICAM1 |  |  |
|  |  | RNASE3 | CCL11 |  |  |
|  |  | RNASE3 | IL17A |  |  |
|  |  | RNASE3 | IL33 |  |  |
|  |  | RNASE3 | IL10 |  |  |
|  |  | RNASE3 | IL6 |  |  |
|  |  | RNASE3 | TNF |  |  |
|  |  | RNASE3 | CCL5 |  |  |
|  |  | RNASE3 | CXCL8 |  |  |
|  |  | RNASE3 | PRG2 |  |  |
|  |  | RPS27A | TNFRSF1A | |  |
|  |  | RPS27A | RIPK3 |  |  |
|  |  | RPS27A | TGFB1 |  |  |
|  |  | RPS27A | HGF |  |  |
|  |  | RPS27A | NFKB1 |  |  |
|  |  | RPS27A | RIPK1 |  |  |
|  |  | RPS27A | IL7 |  |  |
|  |  | RPS27A | TAF1 |  |  |
|  |  | RPS27A | HSPA4 |  |  |
|  |  | RPS27A | SERP1 |  |  |
|  |  | RPS27A | VIMP |  |  |
|  |  | RPS27A | UBAP1 |  |  |
|  |  | RPS27A | HIF1A |  |  |
|  |  | RPS27A | PARP1 |  |  |
|  |  | RPS27A | SMAD3 |  |  |
|  |  | RPS27A | CD14 |  |  |
|  |  | RPS27A | TLR4 |  |  |
|  |  | RPS27A | TNF |  |  |
|  |  | RPS27A | BRCA1 |  |  |
|  |  | RPS27A | PARK2 |  |  |
|  |  | RPS27A | UBC |  |  |
|  |  | S100A4 | TIMP1 |  |  |
|  |  | S100A4 | MMP2 |  |  |
|  |  | S100A4 | TGFB1 |  |  |
|  |  | S100A4 | HGF |  |  |
|  |  | S100A4 | SERPINE1 |  |  |
|  |  | S100A4 | CCL2 |  |  |
|  |  | S100A4 | LGALS3 |  |  |
|  |  | S100A4 | IL1A |  |  |
|  |  | S100A4 | IL1B |  |  |
|  |  | S100A4 | HSPA4 |  |  |
|  |  | S100A4 | CXCL8 |  |  |
|  |  | S100A4 | PLG |  |  |
|  |  | S100A4 | SMAD3 |  |  |
|  |  | S100A4 | CTGF |  |  |
|  |  | S100A4 | CXCR4 |  |  |
|  |  | S100A4 | TNF |  |  |
|  |  | S100A4 | IL6 |  |  |
|  |  | S100A4 | VEGFA |  |  |
|  |  | S100A4 | TLR4 |  |  |
|  |  | S100A4 | PECAM1 |  |  |
|  |  | S100A4 | MMP9 |  |  |
|  |  | S100A4 | CCL5 |  |  |
|  |  | S100A4 | AGER |  |  |
|  |  | S100A8 | RETN |  |  |
|  |  | S100A8 | SERPINE1 |  |  |
|  |  | S100A8 | MPO |  |  |
|  |  | S100A8 | IL1RN |  |  |
|  |  | S100A8 | TLR2 |  |  |
|  |  | S100A8 | CYBA |  |  |
|  |  | S100A8 | IL1B |  |  |
|  |  | S100A8 | CD14 |  |  |
|  |  | S100A8 | CXCL8 |  |  |
|  |  | S100A8 | SMAD3 |  |  |
|  |  | S100A8 | HLA-DRB1 | |  |
|  |  | S100A8 | PTGS2 |  |  |
|  |  | S100A8 | CTSS |  |  |
|  |  | S100A8 | LCN2 |  |  |
|  |  | S100A8 | MMP9 |  |  |
|  |  | S100A8 | IL10 |  |  |
|  |  | S100A8 | IL6 |  |  |
|  |  | S100A8 | TNF |  |  |
|  |  | S100A8 | ITGB2 |  |  |
|  |  | S100A8 | AGER |  |  |
|  |  | S100A8 | MYD88 |  |  |
|  |  | S100A8 | TLR4 |  |  |
|  |  | S100A8 | S100A9 |  |  |
|  |  | S100A9 | RETN |  |  |
|  |  | S100A9 | MPO |  |  |
|  |  | S100A9 | CCL2 |  |  |
|  |  | S100A9 | CRP |  |  |
|  |  | S100A9 | IL1RN |  |  |
|  |  | S100A9 | TLR2 |  |  |
|  |  | S100A9 | CYBA |  |  |
|  |  | S100A9 | IL1B |  |  |
|  |  | S100A9 | PPBP |  |  |
|  |  | S100A9 | CD14 |  |  |
|  |  | S100A9 | CXCL8 |  |  |
|  |  | S100A9 | CD86 |  |  |
|  |  | S100A9 | CD163 |  |  |
|  |  | S100A9 | S100A8 |  |  |
|  |  | S100A9 | PECAM1 |  |  |
|  |  | S100A9 | IL10 |  |  |
|  |  | S100A9 | IL6 |  |  |
|  |  | S100A9 | LCN2 |  |  |
|  |  | S100A9 | TNF |  |  |
|  |  | S100A9 | AGER |  |  |
|  |  | S100A9 | CTSS |  |  |
|  |  | S100A9 | MMP9 |  |  |
|  |  | S100A9 | ITGB2 |  |  |
|  |  | S100A9 | SERPINA1 | |  |
|  |  | S100A9 | MYD88 |  |  |
|  |  | S100A9 | TLR4 |  |  |
|  |  | SAA4 | CETP |  |  |
|  |  | SAA4 | PON1 |  |  |
|  |  | SAA4 | APOB |  |  |
|  |  | SAA4 | APOA1 |  |  |
|  |  | SAA4 | CRP |  |  |
|  |  | SAA4 | APCS |  |  |
|  |  | SAA4 | SERPINC1 | |  |
|  |  | SAA4 | F2 |  |  |
|  |  | SAA4 | SERPINA1 | |  |
|  |  | SAA4 | FGA |  |  |
|  |  | SAA4 | VIMP |  |  |
|  |  | SCUBE1 | C1S |  |  |
|  |  | SELE | CX3CL1 |  |  |
|  |  | SELE | TNFRSF1A | |  |
|  |  | SELE | HMOX1 |  |  |
|  |  | SELE | TIMP1 |  |  |
|  |  | SELE | MMP2 |  |  |
|  |  | SELE | PLAT |  |  |
|  |  | SELE | RETN |  |  |
|  |  | SELE | HGF |  |  |
|  |  | SELE | SERPINE1 |  |  |
|  |  | SELE | MPO |  |  |
|  |  | SELE | CCL2 |  |  |
|  |  | SELE | IL2 |  |  |
|  |  | SELE | SELPLG |  |  |
|  |  | SELE | IFNG |  |  |
|  |  | SELE | IL5 |  |  |
|  |  | SELE | APOB |  |  |
|  |  | SELE | APOA1 |  |  |
|  |  | SELE | LGALS3 |  |  |
|  |  | SELE | CRP |  |  |
|  |  | SELE | TLR2 |  |  |
|  |  | SELE | VWF |  |  |
|  |  | SELE | ITGA2B |  |  |
|  |  | SELE | IL1A |  |  |
|  |  | SELE | IL1B |  |  |
|  |  | SELE | ICAM1 |  |  |
|  |  | SELE | REN |  |  |
|  |  | SELE | SERPING1 | |  |
|  |  | SELE | IL18 |  |  |
|  |  | SELE | PPARG |  |  |
|  |  | SELE | ACE |  |  |
|  |  | SELE | VCAM1 |  |  |
|  |  | SELE | PF4 |  |  |
|  |  | SELE | NOS3 |  |  |
|  |  | SELE | MMP3 |  |  |
|  |  | SELE | CCL11 |  |  |
|  |  | SELE | CXCL8 |  |  |
|  |  | SELE | F2 |  |  |
|  |  | SELE | PLG |  |  |
|  |  | SELE | OLR1 |  |  |
|  |  | SELE | LEP |  |  |
|  |  | SELE | ANGPT2 |  |  |
|  |  | SELE | MMP1 |  |  |
|  |  | SELE | GP1BA |  |  |
|  |  | SELE | AGT |  |  |
|  |  | SELE | MYD88 |  |  |
|  |  | SELE | PGF |  |  |
|  |  | SELE | TNFRSF1B | |  |
|  |  | SELE | ITGB3 |  |  |
|  |  | SELE | CD86 |  |  |
|  |  | SELE | IL17A |  |  |
|  |  | SELE | SERPINC1 | |  |
|  |  | SELE | ANGPT1 |  |  |
|  |  | SELE | HIF1A |  |  |
|  |  | SELE | CCL3 |  |  |
|  |  | SELE | PTGS2 |  |  |
|  |  | SELE | CD40LG |  |  |
|  |  | SELE | TEK |  |  |
|  |  | SELE | CXCR4 |  |  |
|  |  | SELE | INS |  |  |
|  |  | SELE | CCL5 |  |  |
|  |  | SELE | TLR4 |  |  |
|  |  | SELE | MMP9 |  |  |
|  |  | SELE | IL10 |  |  |
|  |  | SELE | ITGB2 |  |  |
|  |  | SELE | EDN1 |  |  |
|  |  | SELE | VEGFA |  |  |
|  |  | SELE | THBD |  |  |
|  |  | SELE | F3 |  |  |
|  |  | SELE | PECAM1 |  |  |
|  |  | SELE | IL6 |  |  |
|  |  | SELE | TNF |  |  |
|  |  | SELE | BSG |  |  |
|  |  | SELP | CX3CL1 |  |  |
|  |  | SELP | CPB2 |  |  |
|  |  | SELP | HMOX1 |  |  |
|  |  | SELP | TIMP1 |  |  |
|  |  | SELP | MMP2 |  |  |
|  |  | SELP | PLAT |  |  |
|  |  | SELP | SERPINE1 |  |  |
|  |  | SELP | MPO |  |  |
|  |  | SELP | CCL2 |  |  |
|  |  | SELP | IL2 |  |  |
|  |  | SELP | SELPLG |  |  |
|  |  | SELP | IFNG |  |  |
|  |  | SELP | APOB |  |  |
|  |  | SELP | APOA1 |  |  |
|  |  | SELP | LGALS3 |  |  |
|  |  | SELP | CRP |  |  |
|  |  | SELP | TLR2 |  |  |
|  |  | SELP | VWF |  |  |
|  |  | SELP | ITGA2B |  |  |
|  |  | SELP | IL1A |  |  |
|  |  | SELP | IL1B |  |  |
|  |  | SELP | CD28 |  |  |
|  |  | SELP | REN |  |  |
|  |  | SELP | PTGS2 |  |  |
|  |  | SELP | OLR1 |  |  |
|  |  | SELP | CX3CR1 |  |  |
|  |  | SELP | FERMT3 |  |  |
|  |  | SELP | SERPING1 | |  |
|  |  | SELP | IL18 |  |  |
|  |  | SELP | GP5 |  |  |
|  |  | SELP | KNG1 |  |  |
|  |  | SELP | IL17A |  |  |
|  |  | SELP | ACE |  |  |
|  |  | SELP | CD86 |  |  |
|  |  | SELP | ANGPT1 |  |  |
|  |  | SELP | INS |  |  |
|  |  | SELP | CCL3 |  |  |
|  |  | SELP | F10 |  |  |
|  |  | SELP | TEK |  |  |
|  |  | SELP | ENTPD1 |  |  |
|  |  | SELP | FCGR2A |  |  |
|  |  | SELP | ANGPT2 |  |  |
|  |  | SELP | F7 |  |  |
|  |  | SELP | ITGA2 |  |  |
|  |  | SELP | CXCR4 |  |  |
|  |  | SELP | MMP9 |  |  |
|  |  | SELP | EDN1 |  |  |
|  |  | SELP | IL10 |  |  |
|  |  | SELP | NOS3 |  |  |
|  |  | SELP | VEGFA |  |  |
|  |  | SELP | PLG |  |  |
|  |  | SELP | THBD |  |  |
|  |  | SELP | TREML1 |  |  |
|  |  | SELP | CCL5 |  |  |
|  |  | SELP | SERPINC1 | |  |
|  |  | SELP | IL6 |  |  |
|  |  | SELP | CXCL8 |  |  |
|  |  | SELP | TLR4 |  |  |
|  |  | SELP | TNF |  |  |
|  |  | SELP | PPBP |  |  |
|  |  | SELP | F2 |  |  |
|  |  | SELP | CD40LG |  |  |
|  |  | SELP | PF4 |  |  |
|  |  | SELP | PECAM1 |  |  |
|  |  | SELP | ITGB3 |  |  |
|  |  | SELP | VCAM1 |  |  |
|  |  | SELP | F3 |  |  |
|  |  | SELP | ICAM1 |  |  |
|  |  | SELP | ITGB2 |  |  |
|  |  | SELP | CD63 |  |  |
|  |  | SELP | GP1BA |  |  |
|  |  | SELP | PTX3 |  |  |
|  |  | SELPLG | MPO |  |  |
|  |  | SELPLG | TLR4 |  |  |
|  |  | SELPLG | ITGB3 |  |  |
|  |  | SELPLG | CXCL8 |  |  |
|  |  | SELPLG | CCL5 |  |  |
|  |  | SELPLG | CX3CR1 |  |  |
|  |  | SELPLG | IL1B |  |  |
|  |  | SELPLG | CD86 |  |  |
|  |  | SELPLG | PF4 |  |  |
|  |  | SELPLG | PECAM1 |  |  |
|  |  | SELPLG | TNF |  |  |
|  |  | SELPLG | CXCR4 |  |  |
|  |  | SELPLG | FERMT3 |  |  |
|  |  | SELPLG | ITGA2B |  |  |
|  |  | SELPLG | CD40LG |  |  |
|  |  | SELPLG | VWF |  |  |
|  |  | SELPLG | F3 |  |  |
|  |  | SELPLG | VCAM1 |  |  |
|  |  | SELPLG | GP1BA |  |  |
|  |  | SELPLG | ICAM1 |  |  |
|  |  | SELPLG | ITGB2 |  |  |
|  |  | SELPLG | SELE |  |  |
|  |  | SELPLG | SELP |  |  |
|  |  | SEMA4D | HGF |  |  |
|  |  | SEMA4D | VWF |  |  |
|  |  | SEMA4D | MRAS |  |  |
|  |  | SEMA4D | BDNF |  |  |
|  |  | SEMA4D | PGF |  |  |
|  |  | SEMA4D | PECAM1 |  |  |
|  |  | SEMA4D | VEGFA |  |  |
|  |  | SERP1 | HMOX1 |  |  |
|  |  | SERP1 | RPS27A |  |  |
|  |  | SERP1 | PLAU |  |  |
|  |  | SERPINA1 | CPB2 |  |  |
|  |  | SERPINA1 | CHGA |  |  |
|  |  | SERPINA1 | TIMP1 |  |  |
|  |  | SERPINA1 | MMP2 |  |  |
|  |  | SERPINA1 | PLAT |  |  |
|  |  | SERPINA1 | PON1 |  |  |
|  |  | SERPINA1 | HGF |  |  |
|  |  | SERPINA1 | MPO |  |  |
|  |  | SERPINA1 | HRG |  |  |
|  |  | SERPINA1 | APOB |  |  |
|  |  | SERPINA1 | MMP8 |  |  |
|  |  | SERPINA1 | APOA1 |  |  |
|  |  | SERPINA1 | EPO |  |  |
|  |  | SERPINA1 | CRP |  |  |
|  |  | SERPINA1 | APCS |  |  |
|  |  | SERPINA1 | IL1RN |  |  |
|  |  | SERPINA1 | VWF |  |  |
|  |  | SERPINA1 | IL1B |  |  |
|  |  | SERPINA1 | CP |  |  |
|  |  | SERPINA1 | ICAM1 |  |  |
|  |  | SERPINA1 | KNG1 |  |  |
|  |  | SERPINA1 | ITIH4 |  |  |
|  |  | SERPINA1 | REN |  |  |
|  |  | SERPINA1 | SAA4 |  |  |
|  |  | SERPINA1 | IGF1 |  |  |
|  |  | SERPINA1 | HSPA4 |  |  |
|  |  | SERPINA1 | FGA |  |  |
|  |  | SERPINA1 | CXCL8 |  |  |
|  |  | SERPINA1 | F2 |  |  |
|  |  | SERPINA1 | PLG |  |  |
|  |  | SERPINA1 | SERPINF2 |  |  |
|  |  | SERPINA1 | F3 |  |  |
|  |  | SERPINA1 | SERPINC1 | |  |
|  |  | SERPINA1 | ADAMTS4 | |  |
|  |  | SERPINA1 | S100A9 |  |  |
|  |  | SERPINA1 | MMP9 |  |  |
|  |  | SERPINA1 | LCN2 |  |  |
|  |  | SERPINA1 | MBL2 |  |  |
|  |  | SERPINA1 | F10 |  |  |
|  |  | SERPINA1 | F7 |  |  |
|  |  | SERPINA1 | INS |  |  |
|  |  | SERPINA1 | MB |  |  |
|  |  | SERPINA1 | CST3 |  |  |
|  |  | SERPINA1 | C1S |  |  |
|  |  | SERPINA1 | IL6 |  |  |
|  |  | SERPINA1 | TNF |  |  |
|  |  | SERPINA1 | IL10 |  |  |
|  |  | SERPINA1 | VEGFA |  |  |
|  |  | SERPINA12 | RETN |  |  |
|  |  | SERPINA12 | NAMPT |  |  |
|  |  | SERPINA12 | CRP |  |  |
|  |  | SERPINA12 | LEP |  |  |
|  |  | SERPINA12 | TNF |  |  |
|  |  | SERPINA12 | FGF21 |  |  |
|  |  | SERPINA12 | IL6 |  |  |
|  |  | SERPINA12 | INS |  |  |
|  |  | SERPINA5 | CPB2 |  |  |
|  |  | SERPINA5 | PLAT |  |  |
|  |  | SERPINA5 | APOA1 |  |  |
|  |  | SERPINA5 | VWF |  |  |
|  |  | SERPINA5 | FGA |  |  |
|  |  | SERPINA5 | F2 |  |  |
|  |  | SERPINA5 | PLG |  |  |
|  |  | SERPINA5 | SERPINF2 |  |  |
|  |  | SERPINA5 | GP1BA |  |  |
|  |  | SERPINA5 | F3 |  |  |
|  |  | SERPINA5 | THBD |  |  |
|  |  | SERPINA5 | SERPINC1 | |  |
|  |  | SERPINA5 | PLAU |  |  |
|  |  | SERPINA5 | F10 |  |  |
|  |  | SERPINA5 | GP5 |  |  |
|  |  | SERPINC1 | CPB2 |  |  |
|  |  | SERPINC1 | PLAT |  |  |
|  |  | SERPINC1 | MPO |  |  |
|  |  | SERPINC1 | CCL2 |  |  |
|  |  | SERPINC1 | HRG |  |  |
|  |  | SERPINC1 | APOB |  |  |
|  |  | SERPINC1 | APOA1 |  |  |
|  |  | SERPINC1 | EPO |  |  |
|  |  | SERPINC1 | CRP |  |  |
|  |  | SERPINC1 | APCS |  |  |
|  |  | SERPINC1 | VWF |  |  |
|  |  | SERPINC1 | ITGA2B |  |  |
|  |  | SERPINC1 | IL1B |  |  |
|  |  | SERPINC1 | SELP |  |  |
|  |  | SERPINC1 | CP |  |  |
|  |  | SERPINC1 | ICAM1 |  |  |
|  |  | SERPINC1 | KNG1 |  |  |
|  |  | SERPINC1 | ITIH4 |  |  |
|  |  | SERPINC1 | REN |  |  |
|  |  | SERPINC1 | IGFBP1 |  |  |
|  |  | SERPINC1 | HABP2 |  |  |
|  |  | SERPINC1 | SAA4 |  |  |
|  |  | SERPINC1 | SERPING1 | |  |
|  |  | SERPINC1 | ACE |  |  |
|  |  | SERPINC1 | VCAM1 |  |  |
|  |  | SERPINC1 | PPBP |  |  |
|  |  | SERPINC1 | PF4 |  |  |
|  |  | SERPINC1 | FGA |  |  |
|  |  | SERPINC1 | CXCL8 |  |  |
|  |  | SERPINC1 | F2 |  |  |
|  |  | SERPINC1 | PLG |  |  |
|  |  | SERPINC1 | SERPINF2 |  |  |
|  |  | SERPINC1 | GP1BA |  |  |
|  |  | SERPINC1 | SELE |  |  |
|  |  | SERPINC1 | SERPINA5 | |  |
|  |  | SERPINC1 | F3 |  |  |
|  |  | SERPINC1 | AGT |  |  |
|  |  | SERPINC1 | EDN1 |  |  |
|  |  | SERPINC1 | IL10 |  |  |
|  |  | SERPINC1 | CD40LG |  |  |
|  |  | SERPINC1 | VEGFA |  |  |
|  |  | SERPINC1 | SERPINA1 | |  |
|  |  | SERPINC1 | TNF |  |  |
|  |  | SERPINC1 | C1S |  |  |
|  |  | SERPINC1 | PLAU |  |  |
|  |  | SERPINC1 | IL6 |  |  |
|  |  | SERPINC1 | INS |  |  |
|  |  | SERPINC1 | MBL2 |  |  |
|  |  | SERPINC1 | THBD |  |  |
|  |  | SERPINC1 | GP5 |  |  |
|  |  | SERPINC1 | F7 |  |  |
|  |  | SERPINC1 | F10 |  |  |
|  |  | SERPINE1 | TNFRSF1A | |  |
|  |  | SERPINE1 | CPB2 |  |  |
|  |  | SERPINE1 | SIRT1 |  |  |
|  |  | SERPINE1 | MIF |  |  |
|  |  | SERPINE1 | HMOX1 |  |  |
|  |  | SERPINE1 | TIMP1 |  |  |
|  |  | SERPINE1 | MMP2 |  |  |
|  |  | SERPINE1 | PLAT |  |  |
|  |  | SERPINE1 | RETN |  |  |
|  |  | SERPINE1 | TGFB1 |  |  |
|  |  | SERPINE1 | PON1 |  |  |
|  |  | SERPINE1 | HGF |  |  |
|  |  | SERPINE1 | NAMPT |  |  |
|  |  | SERPINE1 | NPPB |  |  |
|  |  | SERPINE1 | IL1RN |  |  |
|  |  | SERPINE1 | ITGA2 |  |  |
|  |  | SERPINE1 | ITGA2B |  |  |
|  |  | SERPINE1 | BDNF |  |  |
|  |  | SERPINE1 | SERPINF2 |  |  |
|  |  | SERPINE1 | S100A4 |  |  |
|  |  | SERPINE1 | GDF15 |  |  |
|  |  | SERPINE1 | CST3 |  |  |
|  |  | SERPINE1 | AGER |  |  |
|  |  | SERPINE1 | ADM |  |  |
|  |  | SERPINE1 | LCN2 |  |  |
|  |  | SERPINE1 | IL18 |  |  |
|  |  | SERPINE1 | IFNG |  |  |
|  |  | SERPINE1 | CD40LG |  |  |
|  |  | SERPINE1 | ANGPT1 |  |  |
|  |  | SERPINE1 | CCL3 |  |  |
|  |  | SERPINE1 | DPP4 |  |  |
|  |  | SERPINE1 | PGF |  |  |
|  |  | SERPINE1 | MMP8 |  |  |
|  |  | SERPINE1 | PF4 |  |  |
|  |  | SERPINE1 | IGFBP1 |  |  |
|  |  | SERPINE1 | S100A8 |  |  |
|  |  | SERPINE1 | TLR4 |  |  |
|  |  | SERPINE1 | PPBP |  |  |
|  |  | SERPINE1 | MPO |  |  |
|  |  | SERPINE1 | ANGPT2 |  |  |
|  |  | SERPINE1 | PECAM1 |  |  |
|  |  | SERPINE1 | IL1A |  |  |
|  |  | SERPINE1 | HRG |  |  |
|  |  | SERPINE1 | KNG1 |  |  |
|  |  | SERPINE1 | IGF1 |  |  |
|  |  | SERPINE1 | PTX3 |  |  |
|  |  | SERPINE1 | PTGS2 |  |  |
|  |  | SERPINE1 | HABP2 |  |  |
|  |  | SERPINE1 | IL10 |  |  |
|  |  | SERPINE1 | CCL5 |  |  |
|  |  | SERPINE1 | APOA1 |  |  |
|  |  | SERPINE1 | HIF1A |  |  |
|  |  | SERPINE1 | APOB |  |  |
|  |  | SERPINE1 | F10 |  |  |
|  |  | SERPINE1 | PPARG |  |  |
|  |  | SERPINE1 | SELP |  |  |
|  |  | SERPINE1 | IL1B |  |  |
|  |  | SERPINE1 | MMP1 |  |  |
|  |  | SERPINE1 | SELE |  |  |
|  |  | SERPINE1 | NOS3 |  |  |
|  |  | SERPINE1 | VCAM1 |  |  |
|  |  | SERPINE1 | EDN1 |  |  |
|  |  | SERPINE1 | ICAM1 |  |  |
|  |  | SERPINE1 | VEGFA |  |  |
|  |  | SERPINE1 | ACE |  |  |
|  |  | SERPINE1 | CXCL8 |  |  |
|  |  | SERPINE1 | REN |  |  |
|  |  | SERPINE1 | INS |  |  |
|  |  | SERPINE1 | TNF |  |  |
|  |  | SERPINE1 | CCL2 |  |  |
|  |  | SERPINE1 | MMP9 |  |  |
|  |  | SERPINE1 | LEP |  |  |
|  |  | SERPINE1 | MMP3 |  |  |
|  |  | SERPINE1 | THBD |  |  |
|  |  | SERPINE1 | CTGF |  |  |
|  |  | SERPINE1 | VWF |  |  |
|  |  | SERPINE1 | F7 |  |  |
|  |  | SERPINE1 | CRP |  |  |
|  |  | SERPINE1 | IL6 |  |  |
|  |  | SERPINE1 | F3 |  |  |
|  |  | SERPINE1 | F2 |  |  |
|  |  | SERPINE1 | AGTR1 |  |  |
|  |  | SERPINE1 | ITGB3 |  |  |
|  |  | SERPINE1 | SMAD3 |  |  |
|  |  | SERPINE1 | PLG |  |  |
|  |  | SERPINE1 | PLAU |  |  |
|  |  | SERPINF2 | CPB2 |  |  |
|  |  | SERPINF2 | PLAT |  |  |
|  |  | SERPINF2 | SERPINE1 |  |  |
|  |  | SERPINF2 | HRG |  |  |
|  |  | SERPINF2 | APOB |  |  |
|  |  | SERPINF2 | APOA1 |  |  |
|  |  | SERPINF2 | CRP |  |  |
|  |  | SERPINF2 | VWF |  |  |
|  |  | SERPINF2 | KNG1 |  |  |
|  |  | SERPINF2 | ITIH4 |  |  |
|  |  | SERPINF2 | HABP2 |  |  |
|  |  | SERPINF2 | PPBP |  |  |
|  |  | SERPINF2 | PF4 |  |  |
|  |  | SERPINF2 | FGA |  |  |
|  |  | SERPINF2 | F2 |  |  |
|  |  | SERPINF2 | PLG |  |  |
|  |  | SERPINF2 | SERPINA5 | |  |
|  |  | SERPINF2 | C1S |  |  |
|  |  | SERPINF2 | SERPINA1 | |  |
|  |  | SERPINF2 | F10 |  |  |
|  |  | SERPINF2 | SERPINC1 | |  |
|  |  | SERPINF2 | THBD |  |  |
|  |  | SERPINF2 | F7 |  |  |
|  |  | SERPINF2 | F3 |  |  |
|  |  | SERPINF2 | PLAU |  |  |
|  |  | SERPING1 | PLAT |  |  |
|  |  | SERPING1 | CRP |  |  |
|  |  | SERPING1 | VWF |  |  |
|  |  | SERPING1 | SELP |  |  |
|  |  | SERPING1 | CP |  |  |
|  |  | SERPING1 | KNG1 |  |  |
|  |  | SERPING1 | ITIH4 |  |  |
|  |  | SERPING1 | COL6A2 |  |  |
|  |  | SERPING1 | SELE |  |  |
|  |  | SERPING1 | VCAM1 |  |  |
|  |  | SERPING1 | ADAMTS7 | |  |
|  |  | SERPING1 | F3 |  |  |
|  |  | SERPING1 | SERPINC1 | |  |
|  |  | SERPING1 | ACE |  |  |
|  |  | SERPING1 | F2 |  |  |
|  |  | SERPING1 | PLG |  |  |
|  |  | SERPING1 | MBL2 |  |  |
|  |  | SERPING1 | C1S |  |  |
|  |  | SH2D1A | CD28 |  |  |
|  |  | SH2D1A | CD40LG |  |  |
|  |  | SIRT1 | IL18 |  |  |
|  |  | SIRT1 | MMP2 |  |  |
|  |  | SIRT1 | TXNIP |  |  |
|  |  | SIRT1 | EDN1 |  |  |
|  |  | SIRT1 | TLR2 |  |  |
|  |  | SIRT1 | ACE |  |  |
|  |  | SIRT1 | MMP8 |  |  |
|  |  | SIRT1 | VCAM1 |  |  |
|  |  | SIRT1 | SERPINE1 |  |  |
|  |  | SIRT1 | ENSP00000485396 | |  |
|  |  | SIRT1 | ICAM1 |  |  |
|  |  | SIRT1 | PARK2 |  |  |
|  |  | SIRT1 | IL10 |  |  |
|  |  | SIRT1 | CRP |  |  |
|  |  | SIRT1 | REN |  |  |
|  |  | SIRT1 | CXCL8 |  |  |
|  |  | SIRT1 | HMGCR |  |  |
|  |  | SIRT1 | CCL2 |  |  |
|  |  | SIRT1 | PTGS2 |  |  |
|  |  | SIRT1 | TLR4 |  |  |
|  |  | SIRT1 | MMP9 |  |  |
|  |  | SIRT1 | VEGFA |  |  |
|  |  | SIRT1 | BDNF |  |  |
|  |  | SIRT1 | HSPA4 |  |  |
|  |  | SIRT1 | YWHAZ |  |  |
|  |  | SIRT1 | SMAD3 |  |  |
|  |  | SIRT1 | IL1B |  |  |
|  |  | SIRT1 | FGF21 |  |  |
|  |  | SIRT1 | HMOX1 |  |  |
|  |  | SIRT1 | IL6 |  |  |
|  |  | SIRT1 | TNF |  |  |
|  |  | SIRT1 | CYCS |  |  |
|  |  | SIRT1 | IGF1 |  |  |
|  |  | SIRT1 | BRCA1 |  |  |
|  |  | SIRT1 | LEP |  |  |
|  |  | SIRT1 | INS |  |  |
|  |  | SIRT1 | NOS3 |  |  |
|  |  | SIRT1 | HDAC9 |  |  |
|  |  | SIRT1 | NFKB1 |  |  |
|  |  | SIRT1 | PARP1 |  |  |
|  |  | SIRT1 | HIF1A |  |  |
|  |  | SIRT1 | NAMPT |  |  |
|  |  | SIRT1 | PPARG |  |  |
|  |  | SIRT1 | PPARGC1A | |  |
|  |  | SMAD3 | SIRT1 |  |  |
|  |  | SMAD3 | TIMP1 |  |  |
|  |  | SMAD3 | MMP2 |  |  |
|  |  | SMAD3 | TGFB1 |  |  |
|  |  | SMAD3 | HGF |  |  |
|  |  | SMAD3 | SERPINE1 |  |  |
|  |  | SMAD3 | CCL2 |  |  |
|  |  | SMAD3 | IL2 |  |  |
|  |  | SMAD3 | IFNG |  |  |
|  |  | SMAD3 | AHR |  |  |
|  |  | SMAD3 | FST |  |  |
|  |  | SMAD3 | IL37 |  |  |
|  |  | SMAD3 | IL1B |  |  |
|  |  | SMAD3 | REN |  |  |
|  |  | SMAD3 | RPS27A |  |  |
|  |  | SMAD3 | BMP6 |  |  |
|  |  | SMAD3 | PPARG |  |  |
|  |  | SMAD3 | ACE |  |  |
|  |  | SMAD3 | MMP3 |  |  |
|  |  | SMAD3 | IGF1 |  |  |
|  |  | SMAD3 | HSPA4 |  |  |
|  |  | SMAD3 | CXCL8 |  |  |
|  |  | SMAD3 | PLG |  |  |
|  |  | SMAD3 | MMP1 |  |  |
|  |  | SMAD3 | IL33 |  |  |
|  |  | SMAD3 | PTGS2 |  |  |
|  |  | SMAD3 | ANGPT1 |  |  |
|  |  | SMAD3 | PECAM1 |  |  |
|  |  | SMAD3 | AGT |  |  |
|  |  | SMAD3 | EDN1 |  |  |
|  |  | SMAD3 | IL17A |  |  |
|  |  | SMAD3 | TLR4 |  |  |
|  |  | SMAD3 | IL10 |  |  |
|  |  | SMAD3 | INS |  |  |
|  |  | SMAD3 | IL2RA |  |  |
|  |  | SMAD3 | VEGFA |  |  |
|  |  | SMAD3 | TNF |  |  |
|  |  | SMAD3 | IL6 |  |  |
|  |  | SMAD3 | MMP9 |  |  |
|  |  | SMAD3 | UBC |  |  |
|  |  | SMAD3 | BRCA1 |  |  |
|  |  | SMAD3 | S100A8 |  |  |
|  |  | SMAD3 | PARP1 |  |  |
|  |  | SMAD3 | CTGF |  |  |
|  |  | SMAD3 | S100A4 |  |  |
|  |  | SMAD3 | HIF1A |  |  |
|  |  | SULT1E1 | YWHAZ |  |  |
|  |  | TAF1 | RPS27A |  |  |
|  |  | TAF1 | UBC |  |  |
|  |  | TEK | TIMP1 |  |  |
|  |  | TEK | MMP2 |  |  |
|  |  | TEK | HGF |  |  |
|  |  | TEK | CCL2 |  |  |
|  |  | TEK | NFKB1 |  |  |
|  |  | TEK | EPO |  |  |
|  |  | TEK | VWF |  |  |
|  |  | TEK | ITGA2B |  |  |
|  |  | TEK | IL1B |  |  |
|  |  | TEK | SELP |  |  |
|  |  | TEK | ICAM1 |  |  |
|  |  | TEK | VCAM1 |  |  |
|  |  | TEK | NOS3 |  |  |
|  |  | TEK | IGF1 |  |  |
|  |  | TEK | CXCL8 |  |  |
|  |  | TEK | PLG |  |  |
|  |  | TEK | ANGPT2 |  |  |
|  |  | TEK | CSF1 |  |  |
|  |  | TEK | SELE |  |  |
|  |  | TEK | CTGF |  |  |
|  |  | TEK | MMP9 |  |  |
|  |  | TEK | ANGPTL2 |  |  |
|  |  | TEK | THBD |  |  |
|  |  | TEK | EDN1 |  |  |
|  |  | TEK | IL10 |  |  |
|  |  | TEK | IL6 |  |  |
|  |  | TEK | TNF |  |  |
|  |  | TEK | CXCR4 |  |  |
|  |  | TEK | HIF1A |  |  |
|  |  | TEK | BDNF |  |  |
|  |  | TEK | FGF21 |  |  |
|  |  | TEK | INS |  |  |
|  |  | TEK | PECAM1 |  |  |
|  |  | TEK | PGF |  |  |
|  |  | TEK | VEGFA |  |  |
|  |  | TEK | ANGPT1 |  |  |
|  |  | TGFB1 | HMOX1 |  |  |
|  |  | TGFB1 | TIMP1 |  |  |
|  |  | TGFB1 | MMP2 |  |  |
|  |  | TGFB1 | LGALS3 |  |  |
|  |  | TGFB1 | BSG |  |  |
|  |  | TGFB1 | F2 |  |  |
|  |  | TGFB1 | NFKB1 |  |  |
|  |  | TGFB1 | PGF |  |  |
|  |  | TGFB1 | FGF21 |  |  |
|  |  | TGFB1 | PLAU |  |  |
|  |  | TGFB1 | TNFRSF11B | |  |
|  |  | TGFB1 | LEP |  |  |
|  |  | TGFB1 | IL7 |  |  |
|  |  | TGFB1 | ANGPT1 |  |  |
|  |  | TGFB1 | VWF |  |  |
|  |  | TGFB1 | CSF1 |  |  |
|  |  | TGFB1 | CD86 |  |  |
|  |  | TGFB1 | BDNF |  |  |
|  |  | TGFB1 | NOS3 |  |  |
|  |  | TGFB1 | S100A4 |  |  |
|  |  | TGFB1 | FST |  |  |
|  |  | TGFB1 | IL18 |  |  |
|  |  | TGFB1 | CRP |  |  |
|  |  | TGFB1 | CXCR4 |  |  |
|  |  | TGFB1 | TLR2 |  |  |
|  |  | TGFB1 | IL1RN |  |  |
|  |  | TGFB1 | AGTR1 |  |  |
|  |  | TGFB1 | VCAM1 |  |  |
|  |  | TGFB1 | IL5 |  |  |
|  |  | TGFB1 | CD28 |  |  |
|  |  | TGFB1 | ICAM1 |  |  |
|  |  | TGFB1 | PECAM1 |  |  |
|  |  | TGFB1 | IL1A |  |  |
|  |  | TGFB1 | BGLAP |  |  |
|  |  | TGFB1 | TLR4 |  |  |
|  |  | TGFB1 | CCL5 |  |  |
|  |  | TGFB1 | CCL3 |  |  |
|  |  | TGFB1 | IL2RA |  |  |
|  |  | TGFB1 | PTGS2 |  |  |
|  |  | TGFB1 | RPS27A |  |  |
|  |  | TGFB1 | AGT |  |  |
|  |  | TGFB1 | ACE |  |  |
|  |  | TGFB1 | INS |  |  |
|  |  | TGFB1 | UBC |  |  |
|  |  | TGFB1 | HIF1A |  |  |
|  |  | TGFB1 | CCL2 |  |  |
|  |  | TGFB1 | CXCL8 |  |  |
|  |  | TGFB1 | IL2 |  |  |
|  |  | TGFB1 | EDN1 |  |  |
|  |  | TGFB1 | SERPINE1 |  |  |
|  |  | TGFB1 | ITGB3 |  |  |
|  |  | TGFB1 | IFNG |  |  |
|  |  | TGFB1 | IL1B |  |  |
|  |  | TGFB1 | REN |  |  |
|  |  | TGFB1 | TNF |  |  |
|  |  | TGFB1 | HGF |  |  |
|  |  | TGFB1 | VEGFA |  |  |
|  |  | TGFB1 | IGF1 |  |  |
|  |  | TGFB1 | LCN2 |  |  |
|  |  | TGFB1 | MMP3 |  |  |
|  |  | TGFB1 | PPARG |  |  |
|  |  | TGFB1 | PLG |  |  |
|  |  | TGFB1 | IL17A |  |  |
|  |  | TGFB1 | MMP1 |  |  |
|  |  | TGFB1 | IL10 |  |  |
|  |  | TGFB1 | SMAD3 |  |  |
|  |  | TGFB1 | CTGF |  |  |
|  |  | TGFB1 | IL6 |  |  |
|  |  | TGFB1 | MMP9 |  |  |
|  |  | THBD | CPB2 |  |  |
|  |  | THBD | HMOX1 |  |  |
|  |  | THBD | PYGB |  |  |
|  |  | THBD | PLAT |  |  |
|  |  | THBD | SERPINE1 |  |  |
|  |  | THBD | CCL2 |  |  |
|  |  | THBD | HRG |  |  |
|  |  | THBD | CRP |  |  |
|  |  | THBD | TLR2 |  |  |
|  |  | THBD | VWF |  |  |
|  |  | THBD | IL1B |  |  |
|  |  | THBD | SELP |  |  |
|  |  | THBD | ICAM1 |  |  |
|  |  | THBD | KNG1 |  |  |
|  |  | THBD | IFNA1 |  |  |
|  |  | THBD | HABP2 |  |  |
|  |  | THBD | ACE |  |  |
|  |  | THBD | VCAM1 |  |  |
|  |  | THBD | PPBP |  |  |
|  |  | THBD | PF4 |  |  |
|  |  | THBD | NOS3 |  |  |
|  |  | THBD | HSPA4 |  |  |
|  |  | THBD | CXCL8 |  |  |
|  |  | THBD | F2 |  |  |
|  |  | THBD | PLG |  |  |
|  |  | THBD | ANGPT2 |  |  |
|  |  | THBD | SERPINF2 |  |  |
|  |  | THBD | GP1BA |  |  |
|  |  | THBD | SELE |  |  |
|  |  | THBD | CD86 |  |  |
|  |  | THBD | SERPINA5 | |  |
|  |  | THBD | F3 |  |  |
|  |  | THBD | CD163 |  |  |
|  |  | THBD | SERPINC1 | |  |
|  |  | THBD | CD40LG |  |  |
|  |  | THBD | ENTPD1 |  |  |
|  |  | THBD | MMP9 |  |  |
|  |  | THBD | PLAU |  |  |
|  |  | THBD | TLR4 |  |  |
|  |  | THBD | AGER |  |  |
|  |  | THBD | F10 |  |  |
|  |  | THBD | F7 |  |  |
|  |  | THBD | TEK |  |  |
|  |  | THBD | ITGB2 |  |  |
|  |  | THBD | MYD88 |  |  |
|  |  | THBD | ANGPT1 |  |  |
|  |  | THBD | IL10 |  |  |
|  |  | THBD | VEGFA |  |  |
|  |  | THBD | PECAM1 |  |  |
|  |  | THBD | EDN1 |  |  |
|  |  | THBD | TNF |  |  |
|  |  | THBD | IL6 |  |  |
|  |  | THBS4 | VWF |  |  |
|  |  | THBS4 | ITGA2B |  |  |
|  |  | THBS4 | ITGA2 |  |  |
|  |  | THBS4 | COL6A2 |  |  |
|  |  | THBS4 | CXCL8 |  |  |
|  |  | THBS4 | ITGB3 |  |  |
|  |  | TIMP1 | CX3CL1 |  |  |
|  |  | TIMP1 | TNFRSF1A | |  |
|  |  | TIMP1 | HMOX1 |  |  |
|  |  | TIMP1 | TEK |  |  |
|  |  | TIMP1 | TNFRSF1B | |  |
|  |  | TIMP1 | AGT |  |  |
|  |  | TIMP1 | F3 |  |  |
|  |  | TIMP1 | CXCL13 |  |  |
|  |  | TIMP1 | TLR2 |  |  |
|  |  | TIMP1 | ITGB3 |  |  |
|  |  | TIMP1 | SELP |  |  |
|  |  | TIMP1 | NAMPT |  |  |
|  |  | TIMP1 | IL1RN |  |  |
|  |  | TIMP1 | GDF15 |  |  |
|  |  | TIMP1 | CXCR4 |  |  |
|  |  | TIMP1 | IL5 |  |  |
|  |  | TIMP1 | SELE |  |  |
|  |  | TIMP1 | IL2 |  |  |
|  |  | TIMP1 | NOS3 |  |  |
|  |  | TIMP1 | TNFRSF11B | |  |
|  |  | TIMP1 | PF4 |  |  |
|  |  | TIMP1 | PGF |  |  |
|  |  | TIMP1 | ANGPT1 |  |  |
|  |  | TIMP1 | REN |  |  |
|  |  | TIMP1 | IL18 |  |  |
|  |  | TIMP1 | CHI3L1 |  |  |
|  |  | TIMP1 | CCL11 |  |  |
|  |  | TIMP1 | SERPINA1 | |  |
|  |  | TIMP1 | TLR4 |  |  |
|  |  | TIMP1 | CSF1 |  |  |
|  |  | TIMP1 | PTX3 |  |  |
|  |  | TIMP1 | VWF |  |  |
|  |  | TIMP1 | ANGPT2 |  |  |
|  |  | TIMP1 | PPARG |  |  |
|  |  | TIMP1 | S100A4 |  |  |
|  |  | TIMP1 | INS |  |  |
|  |  | TIMP1 | HIF1A |  |  |
|  |  | TIMP1 | IFNG |  |  |
|  |  | TIMP1 | BSG |  |  |
|  |  | TIMP1 | MPO |  |  |
|  |  | TIMP1 | LEP |  |  |
|  |  | TIMP1 | PECAM1 |  |  |
|  |  | TIMP1 | ACE |  |  |
|  |  | TIMP1 | IGFBP1 |  |  |
|  |  | TIMP1 | CST3 |  |  |
|  |  | TIMP1 | EDN1 |  |  |
|  |  | TIMP1 | IGF1 |  |  |
|  |  | TIMP1 | SMAD3 |  |  |
|  |  | TIMP1 | VCAM1 |  |  |
|  |  | TIMP1 | CCL3 |  |  |
|  |  | TIMP1 | PTGS2 |  |  |
|  |  | TIMP1 | PLAT |  |  |
|  |  | TIMP1 | MMP28 |  |  |
|  |  | TIMP1 | CRP |  |  |
|  |  | TIMP1 | CCL5 |  |  |
|  |  | TIMP1 | IL1A |  |  |
|  |  | TIMP1 | ICAM1 |  |  |
|  |  | TIMP1 | ADAMTS4 | |  |
|  |  | TIMP1 | LGALS3 |  |  |
|  |  | TIMP1 | CXCL8 |  |  |
|  |  | TIMP1 | PLAU |  |  |
|  |  | TIMP1 | PLG |  |  |
|  |  | TIMP1 | CTGF |  |  |
|  |  | TIMP1 | TNF |  |  |
|  |  | TIMP1 | CCL2 |  |  |
|  |  | TIMP1 | IL1B |  |  |
|  |  | TIMP1 | SERPINE1 |  |  |
|  |  | TIMP1 | HGF |  |  |
|  |  | TIMP1 | VEGFA |  |  |
|  |  | TIMP1 | TGFB1 |  |  |
|  |  | TIMP1 | MMP8 |  |  |
|  |  | TIMP1 | IL17A |  |  |
|  |  | TIMP1 | IL10 |  |  |
|  |  | TIMP1 | LCN2 |  |  |
|  |  | TIMP1 | IL6 |  |  |
|  |  | TIMP1 | CD63 |  |  |
|  |  | TIMP1 | MMP1 |  |  |
|  |  | TIMP1 | MMP9 |  |  |
|  |  | TIMP1 | MMP2 |  |  |
|  |  | TIMP1 | MMP3 |  |  |
|  |  | TLR2 | CX3CL1 |  |  |
|  |  | TLR2 | TNFRSF1A | |  |
|  |  | TLR2 | SIRT1 |  |  |
|  |  | TLR2 | HMOX1 |  |  |
|  |  | TLR2 | RIPK3 |  |  |
|  |  | TLR2 | GZMB |  |  |
|  |  | TLR2 | TIMP1 |  |  |
|  |  | TLR2 | MMP2 |  |  |
|  |  | TLR2 | RETN |  |  |
|  |  | TLR2 | TGFB1 |  |  |
|  |  | TLR2 | MPO |  |  |
|  |  | TLR2 | CCL2 |  |  |
|  |  | TLR2 | NFKB1 |  |  |
|  |  | TLR2 | IL2 |  |  |
|  |  | TLR2 | IFNG |  |  |
|  |  | TLR2 | IL5 |  |  |
|  |  | TLR2 | APOB |  |  |
|  |  | TLR2 | LGALS3 |  |  |
|  |  | TLR2 | CRP |  |  |
|  |  | TLR2 | IL1RN |  |  |
|  |  | TLR2 | RIPK1 |  |  |
|  |  | TLR2 | TNFSF4 |  |  |
|  |  | TLR2 | IL2RA |  |  |
|  |  | TLR2 | PECAM1 |  |  |
|  |  | TLR2 | PTX3 |  |  |
|  |  | TLR2 | PPBP |  |  |
|  |  | TLR2 | FASLG |  |  |
|  |  | TLR2 | CXCL13 |  |  |
|  |  | TLR2 | ENTPD1 |  |  |
|  |  | TLR2 | REN |  |  |
|  |  | TLR2 | LEP |  |  |
|  |  | TLR2 | F3 |  |  |
|  |  | TLR2 | CXCL16 |  |  |
|  |  | TLR2 | VWF |  |  |
|  |  | TLR2 | IL37 |  |  |
|  |  | TLR2 | IL7 |  |  |
|  |  | TLR2 | AGTR1 |  |  |
|  |  | TLR2 | LCN2 |  |  |
|  |  | TLR2 | MMP1 |  |  |
|  |  | TLR2 | HIF1A |  |  |
|  |  | TLR2 | CYBA |  |  |
|  |  | TLR2 | TNFRSF1B | |  |
|  |  | TLR2 | EDN1 |  |  |
|  |  | TLR2 | MMP3 |  |  |
|  |  | TLR2 | CCL11 |  |  |
|  |  | TLR2 | SELP |  |  |
|  |  | TLR2 | ITGB3 |  |  |
|  |  | TLR2 | CTSS |  |  |
|  |  | TLR2 | VEGFA |  |  |
|  |  | TLR2 | CD28 |  |  |
|  |  | TLR2 | PPARG |  |  |
|  |  | TLR2 | VCAM1 |  |  |
|  |  | TLR2 | SELE |  |  |
|  |  | TLR2 | CSF1 |  |  |
|  |  | TLR2 | CX3CR1 |  |  |
|  |  | TLR2 | THBD |  |  |
|  |  | TLR2 | CD40LG |  |  |
|  |  | TLR2 | IL33 |  |  |
|  |  | TLR2 | INS |  |  |
|  |  | TLR2 | PTGS2 |  |  |
|  |  | TLR2 | S100A9 |  |  |
|  |  | TLR2 | ICAM1 |  |  |
|  |  | TLR2 | IFNA1 |  |  |
|  |  | TLR2 | IL1A |  |  |
|  |  | TLR2 | S100A8 |  |  |
|  |  | TLR2 | OLR1 |  |  |
|  |  | TLR2 | ITGB2 |  |  |
|  |  | TLR2 | CCL5 |  |  |
|  |  | TLR2 | MBL2 |  |  |
|  |  | TLR2 | FCGR2A |  |  |
|  |  | TLR2 | IL18 |  |  |
|  |  | TLR2 | CCL3 |  |  |
|  |  | TLR2 | CD163 |  |  |
|  |  | TLR2 | MMP9 |  |  |
|  |  | TLR2 | CD86 |  |  |
|  |  | TLR2 | AGER |  |  |
|  |  | TLR2 | ITIH4 |  |  |
|  |  | TLR2 | IL17A |  |  |
|  |  | TLR2 | CXCL8 |  |  |
|  |  | TLR2 | IL6 |  |  |
|  |  | TLR2 | IL10 |  |  |
|  |  | TLR2 | TLR4 |  |  |
|  |  | TLR2 | IL1B |  |  |
|  |  | TLR2 | TNF |  |  |
|  |  | TLR2 | CXCR4 |  |  |
|  |  | TLR2 | HSPA4 |  |  |
|  |  | TLR2 | MYD88 |  |  |
|  |  | TLR2 | CD14 |  |  |
|  |  | TLR2 | HSPD1 |  |  |
|  |  | TLR4 | CX3CL1 |  |  |
|  |  | TLR4 | TNFRSF1A | |  |
|  |  | TLR4 | SIRT1 |  |  |
|  |  | TLR4 | MIF |  |  |
|  |  | TLR4 | HMOX1 |  |  |
|  |  | TLR4 | RIPK3 |  |  |
|  |  | TLR4 | GZMB |  |  |
|  |  | TLR4 | TIMP1 |  |  |
|  |  | TLR4 | MMP2 |  |  |
|  |  | TLR4 | RETN |  |  |
|  |  | TLR4 | TGFB1 |  |  |
|  |  | TLR4 | HGF |  |  |
|  |  | TLR4 | NAMPT |  |  |
|  |  | TLR4 | SERPINE1 |  |  |
|  |  | TLR4 | MPO |  |  |
|  |  | TLR4 | CCL2 |  |  |
|  |  | TLR4 | NFKB1 |  |  |
|  |  | TLR4 | IL2 |  |  |
|  |  | TLR4 | SELPLG |  |  |
|  |  | TLR4 | IFNG |  |  |
|  |  | TLR4 | IL5 |  |  |
|  |  | TLR4 | APOB |  |  |
|  |  | TLR4 | MMP8 |  |  |
|  |  | TLR4 | APOA1 |  |  |
|  |  | TLR4 | AHR |  |  |
|  |  | TLR4 | LGALS3 |  |  |
|  |  | TLR4 | CRP |  |  |
|  |  | TLR4 | IL1RN |  |  |
|  |  | TLR4 | RIPK1 |  |  |
|  |  | TLR4 | TLR2 |  |  |
|  |  | TLR4 | VWF |  |  |
|  |  | TLR4 | CYBA |  |  |
|  |  | TLR4 | IL37 |  |  |
|  |  | TLR4 | IL1A |  |  |
|  |  | TLR4 | IL1B |  |  |
|  |  | TLR4 | SELP |  |  |
|  |  | TLR4 | IL7 |  |  |
|  |  | TLR4 | ICAM1 |  |  |
|  |  | TLR4 | PPARGC1A | |  |
|  |  | TLR4 | ITIH4 |  |  |
|  |  | TLR4 | FCGR2A |  |  |
|  |  | TLR4 | REN |  |  |
|  |  | TLR4 | RPS27A |  |  |
|  |  | TLR4 | IFNA1 |  |  |
|  |  | TLR4 | IL18 |  |  |
|  |  | TLR4 | TNFSF4 |  |  |
|  |  | TLR4 | CXCL13 |  |  |
|  |  | TLR4 | PPARG |  |  |
|  |  | TLR4 | ACE |  |  |
|  |  | TLR4 | VCAM1 |  |  |
|  |  | TLR4 | PTX3 |  |  |
|  |  | TLR4 | PPBP |  |  |
|  |  | TLR4 | PF4 |  |  |
|  |  | TLR4 | NOS3 |  |  |
|  |  | TLR4 | MMP3 |  |  |
|  |  | TLR4 | CCL11 |  |  |
|  |  | TLR4 | IGF1 |  |  |
|  |  | TLR4 | HSPA4 |  |  |
|  |  | TLR4 | IL12A |  |  |
|  |  | TLR4 | CD14 |  |  |
|  |  | TLR4 | FGA |  |  |
|  |  | TLR4 | CXCL8 |  |  |
|  |  | TLR4 | CYCS |  |  |
|  |  | TLR4 | MLKL |  |  |
|  |  | TLR4 | F2 |  |  |
|  |  | TLR4 | PLG |  |  |
|  |  | TLR4 | OLR1 |  |  |
|  |  | TLR4 | LEP |  |  |
|  |  | TLR4 | MMP1 |  |  |
|  |  | TLR4 | CD28 |  |  |
|  |  | TLR4 | CSF1 |  |  |
|  |  | TLR4 | SELE |  |  |
|  |  | TLR4 | CD86 |  |  |
|  |  | TLR4 | SMAD3 |  |  |
|  |  | TLR4 | F3 |  |  |
|  |  | TLR4 | IL17A |  |  |
|  |  | TLR4 | FAS |  |  |
|  |  | TLR4 | CX3CR1 |  |  |
|  |  | TLR4 | CD163 |  |  |
|  |  | TLR4 | PTGS2 |  |  |
|  |  | TLR4 | FASLG |  |  |
|  |  | TLR4 | S100A4 |  |  |
|  |  | TLR4 | S100A8 |  |  |
|  |  | TLR4 | S100A9 |  |  |
|  |  | TLR4 | CTSS |  |  |
|  |  | TLR4 | CNR1 |  |  |
|  |  | TLR4 | CD40LG |  |  |
|  |  | TLR4 | ENTPD1 |  |  |
|  |  | TLR4 | MMP9 |  |  |
|  |  | TLR4 | LCN2 |  |  |
|  |  | TLR4 | MBL2 |  |  |
|  |  | TLR4 | IL2RA |  |  |
|  |  | TLR4 | TXNIP |  |  |
|  |  | TLR4 | EDN1 |  |  |
|  |  | TLR4 | TNFRSF1B | |  |
|  |  | TLR4 | PECAM1 |  |  |
|  |  | TLR4 | BDNF |  |  |
|  |  | TLR4 | THBD |  |  |
|  |  | TLR4 | HIF1A |  |  |
|  |  | TLR4 | VEGFA |  |  |
|  |  | TLR4 | IL33 |  |  |
|  |  | TLR4 | INS |  |  |
|  |  | TLR4 | ITGB3 |  |  |
|  |  | TLR4 | CCL3 |  |  |
|  |  | TLR4 | ITGB2 |  |  |
|  |  | TLR4 | CCL5 |  |  |
|  |  | TLR4 | CXCR4 |  |  |
|  |  | TLR4 | IL10 |  |  |
|  |  | TLR4 | UBC |  |  |
|  |  | TLR4 | TNF |  |  |
|  |  | TLR4 | IL6 |  |  |
|  |  | TLR4 | AGER |  |  |
|  |  | TLR4 | MYD88 |  |  |
|  |  | TLR4 | HSPD1 |  |  |
|  |  | TNC | ITGA2B |  |  |
|  |  | TNC | ITIH4 |  |  |
|  |  | TNC | ITGB3 |  |  |
|  |  | TNC | ITGA2 |  |  |
|  |  | TNF | CX3CL1 |  |  |
|  |  | TNF | TNFRSF1A | |  |
|  |  | TNF | SIRT1 |  |  |
|  |  | TNF | MIF |  |  |
|  |  | TNF | HMOX1 |  |  |
|  |  | TNF | IL2RB |  |  |
|  |  | TNF | RIPK3 |  |  |
|  |  | TNF | GZMB |  |  |
|  |  | TNF | TIMP1 |  |  |
|  |  | TNF | MMP2 |  |  |
|  |  | TNF | PLAT |  |  |
|  |  | TNF | RETN |  |  |
|  |  | TNF | TGFB1 |  |  |
|  |  | TNF | PON1 |  |  |
|  |  | TNF | HGF |  |  |
|  |  | TNF | NAMPT |  |  |
|  |  | TNF | SERPINE1 |  |  |
|  |  | TNF | MPO |  |  |
|  |  | TNF | CCL2 |  |  |
|  |  | TNF | NFKB1 |  |  |
|  |  | TNF | IL2 |  |  |
|  |  | TNF | SELPLG |  |  |
|  |  | TNF | IFNG |  |  |
|  |  | TNF | IL5 |  |  |
|  |  | TNF | APOB |  |  |
|  |  | TNF | MMP8 |  |  |
|  |  | TNF | APOA1 |  |  |
|  |  | TNF | AHR |  |  |
|  |  | TNF | EPO |  |  |
|  |  | TNF | GDF15 |  |  |
|  |  | TNF | LGALS3 |  |  |
|  |  | TNF | CRP |  |  |
|  |  | TNF | APCS |  |  |
|  |  | TNF | CHI3L1 |  |  |
|  |  | TNF | IL1RN |  |  |
|  |  | TNF | RIPK1 |  |  |
|  |  | TNF | TLR2 |  |  |
|  |  | TNF | VWF |  |  |
|  |  | TNF | CYBA |  |  |
|  |  | TNF | ITGA2B |  |  |
|  |  | TNF | TYR |  |  |
|  |  | TNF | IL37 |  |  |
|  |  | TNF | IL1A |  |  |
|  |  | TNF | IL1B |  |  |
|  |  | TNF | SELP |  |  |
|  |  | TNF | IL7 |  |  |
|  |  | TNF | CP |  |  |
|  |  | TNF | ICAM1 |  |  |
|  |  | TNF | PPARGC1A | |  |
|  |  | TNF | KNG1 |  |  |
|  |  | TNF | ITIH4 |  |  |
|  |  | TNF | FCGR2A |  |  |
|  |  | TNF | REN |  |  |
|  |  | TNF | RPS27A |  |  |
|  |  | TNF | IGFBP1 |  |  |
|  |  | TNF | IFNA1 |  |  |
|  |  | TNF | IL18 |  |  |
|  |  | TNF | TNFSF4 |  |  |
|  |  | TNF | CXCL13 |  |  |
|  |  | TNF | PPARG |  |  |
|  |  | TNF | HMGCR |  |  |
|  |  | TNF | ACE |  |  |
|  |  | TNF | CXCL16 |  |  |
|  |  | TNF | VCAM1 |  |  |
|  |  | TNF | PTX3 |  |  |
|  |  | TNF | PPBP |  |  |
|  |  | TNF | PF4 |  |  |
|  |  | TNF | ITGA2 |  |  |
|  |  | TNF | TNFRSF11B | |  |
|  |  | TNF | NOS3 |  |  |
|  |  | TNF | MMP3 |  |  |
|  |  | TNF | CCL11 |  |  |
|  |  | TNF | RNASE3 |  |  |
|  |  | TNF | IGF1 |  |  |
|  |  | TNF | HSPA4 |  |  |
|  |  | TNF | IL12A |  |  |
|  |  | TNF | CD14 |  |  |
|  |  | TNF | ADRB2 |  |  |
|  |  | TNF | CXCL8 |  |  |
|  |  | TNF | CYCS |  |  |
|  |  | TNF | MLKL |  |  |
|  |  | TNF | F2 |  |  |
|  |  | TNF | PLG |  |  |
|  |  | TNF | OLR1 |  |  |
|  |  | TNF | LEP |  |  |
|  |  | TNF | ANGPT2 |  |  |
|  |  | TNF | MMP1 |  |  |
|  |  | TNF | CD28 |  |  |
|  |  | TNF | CSF1 |  |  |
|  |  | TNF | SELE |  |  |
|  |  | TNF | GAS6 |  |  |
|  |  | TNF | CD86 |  |  |
|  |  | TNF | SMAD3 |  |  |
|  |  | TNF | F3 |  |  |
|  |  | TNF | TNNI3 |  |  |
|  |  | TNF | SERPINA12 | |  |
|  |  | TNF | IL17A |  |  |
|  |  | TNF | FAS |  |  |
|  |  | TNF | CX3CR1 |  |  |
|  |  | TNF | CD163 |  |  |
|  |  | TNF | HLA-DRB1 | |  |
|  |  | TNF | DPP4 |  |  |
|  |  | TNF | AGT |  |  |
|  |  | TNF | PARP1 |  |  |
|  |  | TNF | CHIT1 |  |  |
|  |  | TNF | VIP |  |  |
|  |  | TNF | PTGS2 |  |  |
|  |  | TNF | SERPINC1 | |  |
|  |  | TNF | CTGF |  |  |
|  |  | TNF | ADAMTS4 | |  |
|  |  | TNF | BGLAP |  |  |
|  |  | TNF | S100A4 |  |  |
|  |  | TNF | S100A8 |  |  |
|  |  | TNF | S100A9 |  |  |
|  |  | TNF | CTSS |  |  |
|  |  | TNF | CNR1 |  |  |
|  |  | TNF | CD40LG |  |  |
|  |  | TNF | ENTPD1 |  |  |
|  |  | TNF | MMP9 |  |  |
|  |  | TNF | PLAU |  |  |
|  |  | TNF | LCN2 |  |  |
|  |  | TNF | MBL2 |  |  |
|  |  | TNF | TLR4 |  |  |
|  |  | TNF | AGER |  |  |
|  |  | TNF | F10 |  |  |
|  |  | TNF | F7 |  |  |
|  |  | TNF | TNFRSF1B | |  |
|  |  | TNF | NPPB |  |  |
|  |  | TNF | THBD |  |  |
|  |  | TNF | EDN1 |  |  |
|  |  | TNF | IL2RA |  |  |
|  |  | TNF | TEK |  |  |
|  |  | TNF | IL33 |  |  |
|  |  | TNF | ADAMTS7 | |  |
|  |  | TNF | HSPD1 |  |  |
|  |  | TNF | INS |  |  |
|  |  | TNF | MB |  |  |
|  |  | TNF | ITGB2 |  |  |
|  |  | TNF | CST3 |  |  |
|  |  | TNF | PLA2G2A |  |  |
|  |  | TNF | IL6 |  |  |
|  |  | TNF | CXCR4 |  |  |
|  |  | TNF | IL18R1 |  |  |
|  |  | TNF | IL34 |  |  |
|  |  | TNF | TXNIP |  |  |
|  |  | TNF | ITGB3 |  |  |
|  |  | TNF | ADM |  |  |
|  |  | TNF | CD63 |  |  |
|  |  | TNF | AGTR1 |  |  |
|  |  | TNF | FGF21 |  |  |
|  |  | TNF | PGF |  |  |
|  |  | TNF | ANGPT1 |  |  |
|  |  | TNF | IL32 |  |  |
|  |  | TNF | SERPINA1 | |  |
|  |  | TNF | TNFSF14 |  |  |
|  |  | TNF | HIF1A |  |  |
|  |  | TNF | PECAM1 |  |  |
|  |  | TNF | BDNF |  |  |
|  |  | TNF | TNFRSF11A | |  |
|  |  | TNF | VEGFA |  |  |
|  |  | TNF | MYD88 |  |  |
|  |  | TNF | UBC |  |  |
|  |  | TNF | CCL5 |  |  |
|  |  | TNF | CCL3 |  |  |
|  |  | TNF | IL10 |  |  |
|  |  | TNFRSF11A | TNFRSF1A | |  |
|  |  | TNFRSF11A | TNFRSF11B | |  |
|  |  | TNFRSF11A | CSF1 |  |  |
|  |  | TNFRSF11A | BGLAP |  |  |
|  |  | TNFRSF11A | CD40LG |  |  |
|  |  | TNFRSF11A | TNFRSF1B | |  |
|  |  | TNFRSF11A | IL6 |  |  |
|  |  | TNFRSF11A | TNF |  |  |
|  |  | TNFRSF11A | TNFSF14 |  |  |
|  |  | TNFRSF11B | TNFRSF1A | |  |
|  |  | TNFRSF11B | TIMP1 |  |  |
|  |  | TNFRSF11B | MMP2 |  |  |
|  |  | TNFRSF11B | TGFB1 |  |  |
|  |  | TNFRSF11B | CCL2 |  |  |
|  |  | TNFRSF11B | CRP |  |  |
|  |  | TNFRSF11B | VWF |  |  |
|  |  | TNFRSF11B | IL1A |  |  |
|  |  | TNFRSF11B | IL1B |  |  |
|  |  | TNFRSF11B | IL7 |  |  |
|  |  | TNFRSF11B | ICAM1 |  |  |
|  |  | TNFRSF11B | BMP6 |  |  |
|  |  | TNFRSF11B | PPARG |  |  |
|  |  | TNFRSF11B | FASLG |  |  |
|  |  | TNFRSF11B | CD40LG |  |  |
|  |  | TNFRSF11B | CCL3 |  |  |
|  |  | TNFRSF11B | MMP1 |  |  |
|  |  | TNFRSF11B | PTGS2 |  |  |
|  |  | TNFRSF11B | CTGF |  |  |
|  |  | TNFRSF11B | IL10 |  |  |
|  |  | TNFRSF11B | INS |  |  |
|  |  | TNFRSF11B | MMP3 |  |  |
|  |  | TNFRSF11B | CXCL8 |  |  |
|  |  | TNFRSF11B | IL17A |  |  |
|  |  | TNFRSF11B | MMP9 |  |  |
|  |  | TNFRSF11B | VEGFA |  |  |
|  |  | TNFRSF11B | LEP |  |  |
|  |  | TNFRSF11B | IGF1 |  |  |
|  |  | TNFRSF11B | TNFRSF11A | |  |
|  |  | TNFRSF11B | CSF1 |  |  |
|  |  | TNFRSF11B | IL6 |  |  |
|  |  | TNFRSF11B | TNF |  |  |
|  |  | TNFRSF11B | BGLAP |  |  |
|  |  | TNFRSF1A | CXCL13 |  |  |
|  |  | TNFRSF1A | SERPINE1 |  |  |
|  |  | TNFRSF1A | BDNF |  |  |
|  |  | TNFRSF1A | MMP1 |  |  |
|  |  | TNFRSF1A | LEP |  |  |
|  |  | TNFRSF1A | MPO |  |  |
|  |  | TNFRSF1A | IGF1 |  |  |
|  |  | TNFRSF1A | MMP2 |  |  |
|  |  | TNFRSF1A | GZMB |  |  |
|  |  | TNFRSF1A | TNFRSF11A | |  |
|  |  | TNFRSF1A | MMP3 |  |  |
|  |  | TNFRSF1A | CXCR4 |  |  |
|  |  | TNFRSF1A | CSF1 |  |  |
|  |  | TNFRSF1A | IL5 |  |  |
|  |  | TNFRSF1A | TNFSF14 |  |  |
|  |  | TNFRSF1A | INS |  |  |
|  |  | TNFRSF1A | PTGS2 |  |  |
|  |  | TNFRSF1A | HMOX1 |  |  |
|  |  | TNFRSF1A | IL2RA |  |  |
|  |  | TNFRSF1A | IFNA1 |  |  |
|  |  | TNFRSF1A | IL7 |  |  |
|  |  | TNFRSF1A | CD86 |  |  |
|  |  | TNFRSF1A | CCL3 |  |  |
|  |  | TNFRSF1A | SELE |  |  |
|  |  | TNFRSF1A | TNFRSF11B | |  |
|  |  | TNFRSF1A | IL1RN |  |  |
|  |  | TNFRSF1A | MMP9 |  |  |
|  |  | TNFRSF1A | VEGFA |  |  |
|  |  | TNFRSF1A | TNFSF4 |  |  |
|  |  | TNFRSF1A | CCL5 |  |  |
|  |  | TNFRSF1A | CD28 |  |  |
|  |  | TNFRSF1A | IL18 |  |  |
|  |  | TNFRSF1A | IL17A |  |  |
|  |  | TNFRSF1A | IFNG |  |  |
|  |  | TNFRSF1A | VCAM1 |  |  |
|  |  | TNFRSF1A | CRP |  |  |
|  |  | TNFRSF1A | IL1A |  |  |
|  |  | TNFRSF1A | CYBA |  |  |
|  |  | TNFRSF1A | IL2 |  |  |
|  |  | TNFRSF1A | CCL2 |  |  |
|  |  | TNFRSF1A | TLR2 |  |  |
|  |  | TNFRSF1A | ICAM1 |  |  |
|  |  | TNFRSF1A | CYCS |  |  |
|  |  | TNFRSF1A | TLR4 |  |  |
|  |  | TNFRSF1A | MLKL |  |  |
|  |  | TNFRSF1A | MYD88 |  |  |
|  |  | TNFRSF1A | CXCL8 |  |  |
|  |  | TNFRSF1A | IL6 |  |  |
|  |  | TNFRSF1A | CD40LG |  |  |
|  |  | TNFRSF1A | RIPK3 |  |  |
|  |  | TNFRSF1A | IL1B |  |  |
|  |  | TNFRSF1A | HSPA4 |  |  |
|  |  | TNFRSF1A | RPS27A |  |  |
|  |  | TNFRSF1A | PPARG |  |  |
|  |  | TNFRSF1A | UBC |  |  |
|  |  | TNFRSF1A | TIMP1 |  |  |
|  |  | TNFRSF1A | NFKB1 |  |  |
|  |  | TNFRSF1A | IL10 |  |  |
|  |  | TNFRSF1A | FAS |  |  |
|  |  | TNFRSF1A | FASLG |  |  |
|  |  | TNFRSF1A | TNFRSF1B | |  |
|  |  | TNFRSF1A | TNF |  |  |
|  |  | TNFRSF1A | RIPK1 |  |  |
|  |  | TNFRSF1B | TNFRSF1A | |  |
|  |  | TNFRSF1B | IL2RB |  |  |
|  |  | TNFRSF1B | CHGA |  |  |
|  |  | TNFRSF1B | TIMP1 |  |  |
|  |  | TNFRSF1B | MPO |  |  |
|  |  | TNFRSF1B | CCL2 |  |  |
|  |  | TNFRSF1B | IL2 |  |  |
|  |  | TNFRSF1B | IFNG |  |  |
|  |  | TNFRSF1B | CRP |  |  |
|  |  | TNFRSF1B | IL1RN |  |  |
|  |  | TNFRSF1B | RIPK1 |  |  |
|  |  | TNFRSF1B | TLR2 |  |  |
|  |  | TNFRSF1B | IL1A |  |  |
|  |  | TNFRSF1B | IL1B |  |  |
|  |  | TNFRSF1B | ICAM1 |  |  |
|  |  | TNFRSF1B | IL18 |  |  |
|  |  | TNFRSF1B | VCAM1 |  |  |
|  |  | TNFRSF1B | CD14 |  |  |
|  |  | TNFRSF1B | CXCL8 |  |  |
|  |  | TNFRSF1B | CD28 |  |  |
|  |  | TNFRSF1B | CSF1 |  |  |
|  |  | TNFRSF1B | SELE |  |  |
|  |  | TNFRSF1B | CD86 |  |  |
|  |  | TNFRSF1B | IL17A |  |  |
|  |  | TNFRSF1B | FAS |  |  |
|  |  | TNFRSF1B | FASLG |  |  |
|  |  | TNFRSF1B | CD40LG |  |  |
|  |  | TNFRSF1B | MMP9 |  |  |
|  |  | TNFRSF1B | TLR4 |  |  |
|  |  | TNFRSF1B | VEGFA |  |  |
|  |  | TNFRSF1B | ITGB2 |  |  |
|  |  | TNFRSF1B | IL2RA |  |  |
|  |  | TNFRSF1B | CCL3 |  |  |
|  |  | TNFRSF1B | CCL5 |  |  |
|  |  | TNFRSF1B | TNFRSF11A | |  |
|  |  | TNFRSF1B | IL6 |  |  |
|  |  | TNFRSF1B | TNFSF14 |  |  |
|  |  | TNFRSF1B | IL10 |  |  |
|  |  | TNFRSF1B | TNF |  |  |
|  |  | TNFSF14 | TNFRSF1A | |  |
|  |  | TNFSF14 | TNFSF4 |  |  |
|  |  | TNFSF14 | CD40LG |  |  |
|  |  | TNFSF14 | TNFRSF1B | |  |
|  |  | TNFSF14 | TNF |  |  |
|  |  | TNFSF14 | TNFRSF11A | |  |
|  |  | TNFSF4 | TNFRSF1A | |  |
|  |  | TNFSF4 | GZMB |  |  |
|  |  | TNFSF4 | IL2 |  |  |
|  |  | TNFSF4 | IFNG |  |  |
|  |  | TNFSF4 | IL5 |  |  |
|  |  | TNFSF4 | TLR2 |  |  |
|  |  | TNFSF4 | IL1B |  |  |
|  |  | TNFSF4 | IL7 |  |  |
|  |  | TNFSF4 | ICAM1 |  |  |
|  |  | TNFSF4 | FCGR2A |  |  |
|  |  | TNFSF4 | IFNA1 |  |  |
|  |  | TNFSF4 | IL18 |  |  |
|  |  | TNFSF4 | TNFSF14 |  |  |
|  |  | TNFSF4 | CCL5 |  |  |
|  |  | TNFSF4 | IL2RA |  |  |
|  |  | TNFSF4 | FASLG |  |  |
|  |  | TNFSF4 | IL17A |  |  |
|  |  | TNFSF4 | IL6 |  |  |
|  |  | TNFSF4 | TLR4 |  |  |
|  |  | TNFSF4 | IL10 |  |  |
|  |  | TNFSF4 | IL33 |  |  |
|  |  | TNFSF4 | CD28 |  |  |
|  |  | TNFSF4 | TNF |  |  |
|  |  | TNFSF4 | CD40LG |  |  |
|  |  | TNFSF4 | CD86 |  |  |
|  |  | TNNI1 | TNNT2 |  |  |
|  |  | TNNI1 | TNNI3 |  |  |
|  |  | TNNI1 | MB |  |  |
|  |  | TNNI1 | TNNT1 |  |  |
|  |  | TNNI3 | PYGB |  |  |
|  |  | TNNI3 | MPO |  |  |
|  |  | TNNI3 | TNNT2 |  |  |
|  |  | TNNI3 | GDF15 |  |  |
|  |  | TNNI3 | LGALS3 |  |  |
|  |  | TNNI3 | CRP |  |  |
|  |  | TNNI3 | VWF |  |  |
|  |  | TNNI3 | PIK3C2A |  |  |
|  |  | TNNI3 | REN |  |  |
|  |  | TNNI3 | ACE |  |  |
|  |  | TNNI3 | PRG2 |  |  |
|  |  | TNNI3 | PAPPA |  |  |
|  |  | TNNI3 | INS |  |  |
|  |  | TNNI3 | PECAM1 |  |  |
|  |  | TNNI3 | CST3 |  |  |
|  |  | TNNI3 | EDN1 |  |  |
|  |  | TNNI3 | VEGFA |  |  |
|  |  | TNNI3 | TNF |  |  |
|  |  | TNNI3 | IL6 |  |  |
|  |  | TNNI3 | AVP |  |  |
|  |  | TNNI3 | FABP3 |  |  |
|  |  | TNNI3 | NPPB |  |  |
|  |  | TNNI3 | MB |  |  |
|  |  | TNNI3 | TNNI1 |  |  |
|  |  | TNNI3 | TNNT1 |  |  |
|  |  | TNNT1 | TNNT2 |  |  |
|  |  | TNNT1 | GDF15 |  |  |
|  |  | TNNT1 | CRP |  |  |
|  |  | TNNT1 | ACE |  |  |
|  |  | TNNT1 | TNNI3 |  |  |
|  |  | TNNT1 | TNNI1 |  |  |
|  |  | TNNT1 | FABP3 |  |  |
|  |  | TNNT1 | NPPB |  |  |
|  |  | TNNT1 | MB |  |  |
|  |  | TNNT1 | CST3 |  |  |
|  |  | TNNT2 | PYGB |  |  |
|  |  | TNNT2 | VEGFA |  |  |
|  |  | TNNT2 | VWF |  |  |
|  |  | TNNT2 | CST3 |  |  |
|  |  | TNNT2 | INS |  |  |
|  |  | TNNT2 | GDF15 |  |  |
|  |  | TNNT2 | ACE |  |  |
|  |  | TNNT2 | AVP |  |  |
|  |  | TNNT2 | PECAM1 |  |  |
|  |  | TNNT2 | FABP3 |  |  |
|  |  | TNNT2 | CRP |  |  |
|  |  | TNNT2 | NPPB |  |  |
|  |  | TNNT2 | MB |  |  |
|  |  | TNNT2 | TNNT1 |  |  |
|  |  | TNNT2 | TNNI1 |  |  |
|  |  | TNNT2 | TNNI3 |  |  |
|  |  | TREML1 | SELP |  |  |
|  |  | TREML1 | PPBP |  |  |
|  |  | TREML1 | PF4 |  |  |
|  |  | TREML1 | GP1BA |  |  |
|  |  | TREML1 | PECAM1 |  |  |
|  |  | TXNIP | CETP |  |  |
|  |  | TXNIP | SIRT1 |  |  |
|  |  | TXNIP | HMOX1 |  |  |
|  |  | TXNIP | APOB |  |  |
|  |  | TXNIP | IL1B |  |  |
|  |  | TXNIP | IL18 |  |  |
|  |  | TXNIP | TLR4 |  |  |
|  |  | TXNIP | INS |  |  |
|  |  | TXNIP | IL6 |  |  |
|  |  | TXNIP | TNF |  |  |
|  |  | TXNIP | HIF1A |  |  |
|  |  | TYR | IL2 |  |  |
|  |  | TYR | APOB |  |  |
|  |  | TYR | DPP4 |  |  |
|  |  | TYR | EDN1 |  |  |
|  |  | TYR | TNF |  |  |
|  |  | TYR | CP |  |  |
|  |  | TYR | F2 |  |  |
|  |  | TYR | IGFBP1 |  |  |
|  |  | UBAP1 | RPS27A |  |  |
|  |  | UBAP1 | UBC |  |  |
|  |  | UBC | TNFRSF1A | |  |
|  |  | UBC | MIF |  |  |
|  |  | UBC | RIPK3 |  |  |
|  |  | UBC | TGFB1 |  |  |
|  |  | UBC | HGF |  |  |
|  |  | UBC | NFKB1 |  |  |
|  |  | UBC | RIPK1 |  |  |
|  |  | UBC | RPS27A |  |  |
|  |  | UBC | UBAP1 |  |  |
|  |  | UBC | CD14 |  |  |
|  |  | UBC | SMAD3 |  |  |
|  |  | UBC | PARP1 |  |  |
|  |  | UBC | PARK2 |  |  |
|  |  | UBC | TLR4 |  |  |
|  |  | UBC | YWHAZ |  |  |
|  |  | UBC | VIMP |  |  |
|  |  | UBC | TNF |  |  |
|  |  | UBC | TAF1 |  |  |
|  |  | UBC | BRCA1 |  |  |
|  |  | UBC | HIF1A |  |  |
|  |  | VCAM1 | CX3CL1 |  |  |
|  |  | VCAM1 | TNFRSF1A | |  |
|  |  | VCAM1 | CETP |  |  |
|  |  | VCAM1 | SIRT1 |  |  |
|  |  | VCAM1 | MIF |  |  |
|  |  | VCAM1 | HMOX1 |  |  |
|  |  | VCAM1 | IL2RB |  |  |
|  |  | VCAM1 | TIMP1 |  |  |
|  |  | VCAM1 | MMP2 |  |  |
|  |  | VCAM1 | PLAT |  |  |
|  |  | VCAM1 | RETN |  |  |
|  |  | VCAM1 | TGFB1 |  |  |
|  |  | VCAM1 | PON1 |  |  |
|  |  | VCAM1 | HGF |  |  |
|  |  | VCAM1 | SERPINE1 |  |  |
|  |  | VCAM1 | MPO |  |  |
|  |  | VCAM1 | CCL2 |  |  |
|  |  | VCAM1 | NFKB1 |  |  |
|  |  | VCAM1 | IL2 |  |  |
|  |  | VCAM1 | SELPLG |  |  |
|  |  | VCAM1 | IFNG |  |  |
|  |  | VCAM1 | IL5 |  |  |
|  |  | VCAM1 | APOB |  |  |
|  |  | VCAM1 | MMP8 |  |  |
|  |  | VCAM1 | APOA1 |  |  |
|  |  | VCAM1 | EPO |  |  |
|  |  | VCAM1 | LGALS3 |  |  |
|  |  | VCAM1 | CRP |  |  |
|  |  | VCAM1 | CHI3L1 |  |  |
|  |  | VCAM1 | IL1RN |  |  |
|  |  | VCAM1 | TLR2 |  |  |
|  |  | VCAM1 | VWF |  |  |
|  |  | VCAM1 | CYBA |  |  |
|  |  | VCAM1 | ITGA2B |  |  |
|  |  | VCAM1 | IL37 |  |  |
|  |  | VCAM1 | IL1A |  |  |
|  |  | VCAM1 | IL1B |  |  |
|  |  | VCAM1 | SELP |  |  |
|  |  | VCAM1 | IL7 |  |  |
|  |  | VCAM1 | ICAM1 |  |  |
|  |  | VCAM1 | KNG1 |  |  |
|  |  | VCAM1 | REN |  |  |
|  |  | VCAM1 | IFNA1 |  |  |
|  |  | VCAM1 | SERPING1 | |  |
|  |  | VCAM1 | IL18 |  |  |
|  |  | VCAM1 | CXCL13 |  |  |
|  |  | VCAM1 | PPARG |  |  |
|  |  | VCAM1 | ACE |  |  |
|  |  | VCAM1 | CXCL16 |  |  |
|  |  | VCAM1 | BGLAP |  |  |
|  |  | VCAM1 | PLAU |  |  |
|  |  | VCAM1 | FASLG |  |  |
|  |  | VCAM1 | CD63 |  |  |
|  |  | VCAM1 | PTX3 |  |  |
|  |  | VCAM1 | DPP4 |  |  |
|  |  | VCAM1 | CD28 |  |  |
|  |  | VCAM1 | LCN2 |  |  |
|  |  | VCAM1 | IL33 |  |  |
|  |  | VCAM1 | SERPINC1 | |  |
|  |  | VCAM1 | PPBP |  |  |
|  |  | VCAM1 | ITGA2 |  |  |
|  |  | VCAM1 | TNFRSF1B | |  |
|  |  | VCAM1 | MYD88 |  |  |
|  |  | VCAM1 | AGTR1 |  |  |
|  |  | VCAM1 | PF4 |  |  |
|  |  | VCAM1 | PGF |  |  |
|  |  | VCAM1 | AGT |  |  |
|  |  | VCAM1 | F2 |  |  |
|  |  | VCAM1 | CTGF |  |  |
|  |  | VCAM1 | CX3CR1 |  |  |
|  |  | VCAM1 | BRCA1 |  |  |
|  |  | VCAM1 | HIF1A |  |  |
|  |  | VCAM1 | AGER |  |  |
|  |  | VCAM1 | MMP1 |  |  |
|  |  | VCAM1 | IGF1 |  |  |
|  |  | VCAM1 | CSF1 |  |  |
|  |  | VCAM1 | MMP3 |  |  |
|  |  | VCAM1 | ITGB3 |  |  |
|  |  | VCAM1 | CD86 |  |  |
|  |  | VCAM1 | LEP |  |  |
|  |  | VCAM1 | CTSS |  |  |
|  |  | VCAM1 | ANGPT2 |  |  |
|  |  | VCAM1 | CD163 |  |  |
|  |  | VCAM1 | IL17A |  |  |
|  |  | VCAM1 | ANGPT1 |  |  |
|  |  | VCAM1 | OLR1 |  |  |
|  |  | VCAM1 | PLG |  |  |
|  |  | VCAM1 | CD40LG |  |  |
|  |  | VCAM1 | CCL3 |  |  |
|  |  | VCAM1 | INS |  |  |
|  |  | VCAM1 | TEK |  |  |
|  |  | VCAM1 | CCL11 |  |  |
|  |  | VCAM1 | PTGS2 |  |  |
|  |  | VCAM1 | THBD |  |  |
|  |  | VCAM1 | TLR4 |  |  |
|  |  | VCAM1 | IL10 |  |  |
|  |  | VCAM1 | F3 |  |  |
|  |  | VCAM1 | EDN1 |  |  |
|  |  | VCAM1 | MMP9 |  |  |
|  |  | VCAM1 | CCL5 |  |  |
|  |  | VCAM1 | CXCR4 |  |  |
|  |  | VCAM1 | NOS3 |  |  |
|  |  | VCAM1 | VEGFA |  |  |
|  |  | VCAM1 | CXCL8 |  |  |
|  |  | VCAM1 | PECAM1 |  |  |
|  |  | VCAM1 | IL6 |  |  |
|  |  | VCAM1 | SELE |  |  |
|  |  | VCAM1 | TNF |  |  |
|  |  | VCAM1 | ITGB2 |  |  |
|  |  | VEGFA | CX3CL1 |  |  |
|  |  | VEGFA | TNFRSF1A | |  |
|  |  | VEGFA | SIRT1 |  |  |
|  |  | VEGFA | MIF |  |  |
|  |  | VEGFA | HMOX1 |  |  |
|  |  | VEGFA | GZMB |  |  |
|  |  | VEGFA | CHGA |  |  |
|  |  | VEGFA | TIMP1 |  |  |
|  |  | VEGFA | MMP2 |  |  |
|  |  | VEGFA | PLAT |  |  |
|  |  | VEGFA | RETN |  |  |
|  |  | VEGFA | TGFB1 |  |  |
|  |  | VEGFA | HGF |  |  |
|  |  | VEGFA | NAMPT |  |  |
|  |  | VEGFA | SERPINE1 |  |  |
|  |  | VEGFA | MPO |  |  |
|  |  | VEGFA | CCL2 |  |  |
|  |  | VEGFA | IL2 |  |  |
|  |  | VEGFA | IFNG |  |  |
|  |  | VEGFA | IL5 |  |  |
|  |  | VEGFA | APOB |  |  |
|  |  | VEGFA | MMP8 |  |  |
|  |  | VEGFA | TNNT2 |  |  |
|  |  | VEGFA | AHR |  |  |
|  |  | VEGFA | EPO |  |  |
|  |  | VEGFA | GDF15 |  |  |
|  |  | VEGFA | LGALS3 |  |  |
|  |  | VEGFA | CRP |  |  |
|  |  | VEGFA | CHI3L1 |  |  |
|  |  | VEGFA | FST |  |  |
|  |  | VEGFA | IL1RN |  |  |
|  |  | VEGFA | TLR2 |  |  |
|  |  | VEGFA | VWF |  |  |
|  |  | VEGFA | ITGA2B |  |  |
|  |  | VEGFA | IL1A |  |  |
|  |  | VEGFA | IL1B |  |  |
|  |  | VEGFA | SELP |  |  |
|  |  | VEGFA | IL7 |  |  |
|  |  | VEGFA | ICAM1 |  |  |
|  |  | VEGFA | PPARGC1A | |  |
|  |  | VEGFA | KNG1 |  |  |
|  |  | VEGFA | FCGR2A |  |  |
|  |  | VEGFA | REN |  |  |
|  |  | VEGFA | IGFBP1 |  |  |
|  |  | VEGFA | IFNA1 |  |  |
|  |  | VEGFA | IL18 |  |  |
|  |  | VEGFA | BMP6 |  |  |
|  |  | VEGFA | CXCL13 |  |  |
|  |  | VEGFA | PPARG |  |  |
|  |  | VEGFA | ACE |  |  |
|  |  | VEGFA | VCAM1 |  |  |
|  |  | VEGFA | PTX3 |  |  |
|  |  | VEGFA | PPBP |  |  |
|  |  | VEGFA | PF4 |  |  |
|  |  | VEGFA | TNFRSF11B | |  |
|  |  | VEGFA | NOS3 |  |  |
|  |  | VEGFA | MMP3 |  |  |
|  |  | VEGFA | CCL11 |  |  |
|  |  | VEGFA | IGF1 |  |  |
|  |  | VEGFA | HSPA4 |  |  |
|  |  | VEGFA | CXCL8 |  |  |
|  |  | VEGFA | CYCS |  |  |
|  |  | VEGFA | F2 |  |  |
|  |  | VEGFA | PLG |  |  |
|  |  | VEGFA | LEP |  |  |
|  |  | VEGFA | ANGPT2 |  |  |
|  |  | VEGFA | MMP1 |  |  |
|  |  | VEGFA | CD28 |  |  |
|  |  | VEGFA | CSF1 |  |  |
|  |  | VEGFA | SELE |  |  |
|  |  | VEGFA | CD86 |  |  |
|  |  | VEGFA | SMAD3 |  |  |
|  |  | VEGFA | BSG |  |  |
|  |  | VEGFA | F3 |  |  |
|  |  | VEGFA | TNNI3 |  |  |
|  |  | VEGFA | IL17A |  |  |
|  |  | VEGFA | FAS |  |  |
|  |  | VEGFA | CX3CR1 |  |  |
|  |  | VEGFA | CD163 |  |  |
|  |  | VEGFA | DPP4 |  |  |
|  |  | VEGFA | AGT |  |  |
|  |  | VEGFA | PARP1 |  |  |
|  |  | VEGFA | PTGS2 |  |  |
|  |  | VEGFA | SERPINC1 | |  |
|  |  | VEGFA | FASLG |  |  |
|  |  | VEGFA | CTGF |  |  |
|  |  | VEGFA | ADAMTS4 | |  |
|  |  | VEGFA | BGLAP |  |  |
|  |  | VEGFA | S100A4 |  |  |
|  |  | VEGFA | CD40LG |  |  |
|  |  | VEGFA | MMP9 |  |  |
|  |  | VEGFA | PLAU |  |  |
|  |  | VEGFA | LCN2 |  |  |
|  |  | VEGFA | TLR4 |  |  |
|  |  | VEGFA | AGER |  |  |
|  |  | VEGFA | TNFRSF1B | |  |
|  |  | VEGFA | THBD |  |  |
|  |  | VEGFA | EDN1 |  |  |
|  |  | VEGFA | IL2RA |  |  |
|  |  | VEGFA | TEK |  |  |
|  |  | VEGFA | IL33 |  |  |
|  |  | VEGFA | INS |  |  |
|  |  | VEGFA | CST3 |  |  |
|  |  | VEGFA | IL6 |  |  |
|  |  | VEGFA | CXCR4 |  |  |
|  |  | VEGFA | TNF |  |  |
|  |  | VEGFA | MYD88 |  |  |
|  |  | VEGFA | IL10 |  |  |
|  |  | VEGFA | BDNF |  |  |
|  |  | VEGFA | SERPINA1 | |  |
|  |  | VEGFA | SEMA4D |  |  |
|  |  | VEGFA | BRCA1 |  |  |
|  |  | VEGFA | AGTR1 |  |  |
|  |  | VEGFA | ANGPT1 |  |  |
|  |  | VEGFA | IL32 |  |  |
|  |  | VEGFA | ADM |  |  |
|  |  | VEGFA | HIF1A |  |  |
|  |  | VEGFA | CD63 |  |  |
|  |  | VEGFA | PGF |  |  |
|  |  | VEGFA | ITGB3 |  |  |
|  |  | VEGFA | PECAM1 |  |  |
|  |  | VEGFA | FGF21 |  |  |
|  |  | VEGFA | CCL5 |  |  |
|  |  | VEGFA | CCL3 |  |  |
|  |  | VIMP | CETP |  |  |
|  |  | VIMP | PON1 |  |  |
|  |  | VIMP | APOB |  |  |
|  |  | VIMP | APOA1 |  |  |
|  |  | VIMP | RPS27A |  |  |
|  |  | VIMP | SAA4 |  |  |
|  |  | VIMP | UBC |  |  |
|  |  | VIP | CHGA |  |  |
|  |  | VIP | IFNG |  |  |
|  |  | VIP | IL1B |  |  |
|  |  | VIP | KNG1 |  |  |
|  |  | VIP | IGF1 |  |  |
|  |  | VIP | LEP |  |  |
|  |  | VIP | DPP4 |  |  |
|  |  | VIP | EDN1 |  |  |
|  |  | VIP | IL10 |  |  |
|  |  | VIP | TNF |  |  |
|  |  | VIP | BDNF |  |  |
|  |  | VIP | IL6 |  |  |
|  |  | VIP | INS |  |  |
|  |  | VIP | AVP |  |  |
|  |  | VIP | ADM |  |  |
|  |  | VWF | CPB2 |  |  |
|  |  | VWF | TIMP1 |  |  |
|  |  | VWF | MMP2 |  |  |
|  |  | VWF | PLAT |  |  |
|  |  | VWF | TGFB1 |  |  |
|  |  | VWF | HGF |  |  |
|  |  | VWF | SERPINE1 |  |  |
|  |  | VWF | MPO |  |  |
|  |  | VWF | CCL2 |  |  |
|  |  | VWF | SELPLG |  |  |
|  |  | VWF | IFNG |  |  |
|  |  | VWF | APOB |  |  |
|  |  | VWF | APOA1 |  |  |
|  |  | VWF | TNNT2 |  |  |
|  |  | VWF | EPO |  |  |
|  |  | VWF | LGALS3 |  |  |
|  |  | VWF | CRP |  |  |
|  |  | VWF | TLR2 |  |  |
|  |  | VWF | LEP |  |  |
|  |  | VWF | BRCA1 |  |  |
|  |  | VWF | MMP3 |  |  |
|  |  | VWF | AGER |  |  |
|  |  | VWF | SERPING1 | |  |
|  |  | VWF | CCL5 |  |  |
|  |  | VWF | BGLAP |  |  |
|  |  | VWF | ENTPD1 |  |  |
|  |  | VWF | PLAU |  |  |
|  |  | VWF | TNNI3 |  |  |
|  |  | VWF | SERPINA1 | |  |
|  |  | VWF | PGF |  |  |
|  |  | VWF | REN |  |  |
|  |  | VWF | CD63 |  |  |
|  |  | VWF | ITGB2 |  |  |
|  |  | VWF | IL10 |  |  |
|  |  | VWF | FCGR2A |  |  |
|  |  | VWF | C1S |  |  |
|  |  | VWF | HIF1A |  |  |
|  |  | VWF | TLR4 |  |  |
|  |  | VWF | SEMA4D |  |  |
|  |  | VWF | CTGF |  |  |
|  |  | VWF | THBS4 |  |  |
|  |  | VWF | CXCR4 |  |  |
|  |  | VWF | IGF1 |  |  |
|  |  | VWF | CNR1 |  |  |
|  |  | VWF | KNG1 |  |  |
|  |  | VWF | CD40LG |  |  |
|  |  | VWF | IL1B |  |  |
|  |  | VWF | CD163 |  |  |
|  |  | VWF | MMP9 |  |  |
|  |  | VWF | CXCL8 |  |  |
|  |  | VWF | ANGPT1 |  |  |
|  |  | VWF | ACE |  |  |
|  |  | VWF | INS |  |  |
|  |  | VWF | SERPINF2 |  |  |
|  |  | VWF | TNF |  |  |
|  |  | VWF | PPBP |  |  |
|  |  | VWF | IL6 |  |  |
|  |  | VWF | FGA |  |  |
|  |  | VWF | EDN1 |  |  |
|  |  | VWF | YWHAZ |  |  |
|  |  | VWF | NOS3 |  |  |
|  |  | VWF | ANGPT2 |  |  |
|  |  | VWF | TNFRSF11B | |  |
|  |  | VWF | ICAM1 |  |  |
|  |  | VWF | ITGA2 |  |  |
|  |  | VWF | VCAM1 |  |  |
|  |  | VWF | TEK |  |  |
|  |  | VWF | SELE |  |  |
|  |  | VWF | VEGFA |  |  |
|  |  | VWF | THBD |  |  |
|  |  | VWF | PLG |  |  |
|  |  | VWF | F3 |  |  |
|  |  | VWF | PF4 |  |  |
|  |  | VWF | F7 |  |  |
|  |  | VWF | PECAM1 |  |  |
|  |  | VWF | SERPINA5 | |  |
|  |  | VWF | F10 |  |  |
|  |  | VWF | ITGB3 |  |  |
|  |  | VWF | GP5 |  |  |
|  |  | VWF | F2 |  |  |
|  |  | VWF | SERPINC1 | |  |
|  |  | VWF | SELP |  |  |
|  |  | VWF | ITGA2B |  |  |
|  |  | VWF | GP1BA |  |  |
|  |  | YWHAZ | SIRT1 |  |  |
|  |  | YWHAZ | HMOX1 |  |  |
|  |  | YWHAZ | SULT1E1 |  |  |
|  |  | YWHAZ | IL5 |  |  |
|  |  | YWHAZ | VWF |  |  |
|  |  | YWHAZ | HSPA4 |  |  |
|  |  | YWHAZ | GP1BA |  |  |
|  |  | YWHAZ | ITGB3 |  |  |
|  |  | YWHAZ | HIF1A |  |  |
|  |  | YWHAZ | UBC |  |  |
|  |  | YWHAZ | ITGB2 |  |  |
|  |  | YWHAZ | GP5 |  |  |
