# Supplementary material for: A Complex Heterogeneous Network Model of Disease Regulated by Noncoding RNAs: A Case Study of Unstable Angina Pectoris
Source: Comput Intell Neurosci. 2022 Dec 23;2022:5852089. doi: 10.1155/2022/5852089 (PMC9803582; doi:10.1155/2022/5852089)
Supplement: Supplementary Materials — Table S1: Acronym explanation table. Table S2: MTP network. Table S3: Results of network analysis. Table S4: Results of network modelling. Table S5: Case studies. [file 5852089.f1.zip › Results of Network Analysis.docx]

| Network clustering algorithms | modularity |
| --- | --- |
| fast_greedy | 0.8170166 |
| label_propagation | 0.7788093 |
| asyn_lpa | 0.7701726 |
| kernighan_lin_bisection | 0.2491273 |

| WGCNA hub miRNA | FC_hub miRNA | SimCluster_1 hub miRNA | WGCNA & SimCluster_1 hub miRNA | SimCluster_2 hub miRNA | WGCNA & SimCluster_2 hub miRNA |
| --- | --- | --- | --- | --- | --- |
| hsa-miR-513a-5p | hsa-miR-124-3p | hsa-miR-4318 | hsa-miR-183-3p | hsa-miR-16-5p | hsa-miR-675-3p |
| hsa-miRPlus-A1015 | hsa-miRPlus-A1025 | hsa-miR-3163 | hsa-miR-363-5p | hsa-miR-27a-3p | hsa-miR-892a |
| hsa-miR-631 | hsa-miR-3168 | hsa-miR-892a | hsa-miR-181b-5p | hsa-miR-26b-5p | hsa-miR-3664-5p |
| hsa-miR-652-5p | hsa-miR-1 | hsa-miR-3166 | hsa-miR-204-5p | hsa-miR-146a-5p | hsa-miR-454-3p |
| hsa-miRPlus-A1072 | hsa-miR-599 | hsa-miR-3915 | hsa-let-7a-5p | hsa-miR-3166 | hsa-miR-374a-3p |
| hsa-miR-642b-3p | hsa-miR-199a-5p | hsa-miR-3668 | hsa-miR-320b | hsa-miR-224-3p |  |
| hsa-miR-183-3p | hsa-miR-20a-5p | hsa-miR-337-5p | hsa-miR-20a-5p | hsa-miR-3915 |  |
| hsa-miR-1275 | hsa-miR-21-5p | hsa-miR-20a-5p | hsa-miR-30a-5p | hsa-miR-522-3p |  |
| hsa-miRPlus-C1089 | hsa-miR-3132 | hsa-miR-938 | hsa-miR-675-3p | hsa-miR-4318 |  |
| ebv-miR-BART9* | hsa-miR-1537 | hsa-miR-675-3p | hsa-miR-892a | hsa-miR-892a |  |
| hsa-miR-363-5p | hsa-miR-421 | hsa-miR-3120-3p | hsa-miR-502-3p | hsa-miR-17-5p |  |
| hsa-miR-525-5p | hsa-let-7f-5p | hsa-miR-561-3p |  | hsa-miR-106a-5p |  |
| hsa-miR-219-2-3p | hsa-miR-199a-3p,hsa-miR-199b-3p | hsa-miR-711 |  | hsa-miR-3197 |  |
| hsa-miR-874 | hsa-miR-144-5p | hsa-miR-502-3p |  | hsa-miR-223-5p |  |
| bkv-miR-B1-5p | hsa-miR-3199 | hsa-miR-3197 |  | hsa-miR-190b |  |
| hsa-miR-323a-3p | hsa-miRPlus-A1083 | hsa-let-7g-5p |  | hsa-miR-675-3p |  |
| hsa-miR-668 | hsa-miR-20b-5p | hsa-let-7i-5p |  | hsa-miR-660-5p |  |
| hsa-miRPlus-D1120 | hsa-miRPlus-J98* | hsa-let-7f-5p |  | hsa-miR-3120-3p |  |
| hsa-miR-181b-5p | hsa-miR-223-3p | hsa-let-7d-5p |  | hsa-miR-3186-3p |  |
| hsa-miR-204-5p | hsa-miR-17-5p | hsa-let-7a-5p |  | hsa-miR-3188 |  |
| hsa-let-7a-5p |  | hsa-miR-9-5p |  | hsa-miR-641 |  |
| hsa-miR-106b-3p |  | hsa-miR-29c-3p |  | hsa-miR-320d |  |
| hsa-miR-1297 |  | hsa-miR-29a-3p |  | hsa-miR-3668 |  |
| hsa-miR-320b |  | hsa-miR-186-5p |  | hsa-miR-509-3-5p |  |
| hsa-miR-20a-5p |  | hsa-miR-940 |  | hsa-miR-337-5p |  |
| hsa-miR-26a-5p |  | hsa-miR-320a |  | hsa-miR-4330 |  |
| hsa-miR-92a-3p |  | hsa-miR-320d |  | hsa-miR-491-3p |  |
| hsa-miR-27b-3p |  | hsa-miR-320c |  | hsa-miR-711 |  |
| hsa-miR-30a-5p |  | hsa-miR-320b |  | hsa-miR-652-3p |  |
| hsa-miR-675-3p |  | hsa-miR-3612 |  | hsa-miR-1185-5p |  |
| hsa-miR-892a |  | hsa-miR-1269a |  | hsa-miR-124-5p |  |
| hsa-miR-3664-5p |  | hsa-miR-1299 |  | hsa-miR-30a-3p |  |
| hsv1-miR-H4-3p |  | hsa-miR-1273a |  | hsa-miR-1913 |  |
| hsa-miR-454-3p |  | hsa-miR-30a-5p |  | hsa-miR-1253 |  |
| hsa-miR-374a-3p |  | hsa-miR-30d-5p |  | hsa-miR-1260a |  |
| hsa-miR-548l |  | hsa-miR-30e-5p |  | hsa-miR-1260b |  |
| hsa-miR-505-3p |  | hsa-miR-30c-2-3p |  | hsa-miR-1299 |  |
| hsa-miR-500a-3p |  | hsa-miR-1273c |  | hsa-miR-1273a |  |
| hsa-miR-664-5p |  | hsa-miR-4311 |  | hsa-miR-583 |  |
| hsa-miR-502-3p |  | hsa-miR-133b |  | hsa-miR-299-5p |  |
|  |  | hsa-miR-9-3p |  | hsa-miR-3664-5p |  |
|  |  | hsa-miR-181b-5p |  | hsa-miR-3152-3p |  |
|  |  | hsa-miR-181c-5p |  | hsa-miR-3163 |  |
|  |  | hsa-miR-411-3p |  | hsa-miR-340-3p |  |
|  |  | hsa-miR-183-3p |  | hsa-miR-3168 |  |
|  |  | hsa-miR-1912 |  | hsa-miR-374a-3p |  |
|  |  | hsa-miR-448 |  | hsa-miR-3183 |  |
|  |  | hsa-miR-204-5p |  | hsa-miR-4262 |  |
|  |  | hsa-miR-211-5p |  | hsa-miR-4325 |  |
|  |  | hsa-miR-222-3p |  | hsa-miR-363-3p |  |
|  |  | hsa-miR-641 |  | hsa-miR-4295 |  |
|  |  | hsa-miR-223-3p |  | hsa-miR-454-3p |  |
|  |  | hsa-miR-451a |  |  |  |
|  |  | hsa-miR-2355-5p |  |  |  |
|  |  | hsa-miR-744-5p |  |  |  |
|  |  | hsa-miR-4301 |  |  |  |
|  |  | hsa-miR-300 |  |  |  |
|  |  | hsa-miR-652-3p |  |  |  |
|  |  | hsa-miR-3605-5p |  |  |  |
|  |  | hsa-miR-873-5p |  |  |  |
|  |  | hsa-miR-363-5p |  |  |  |
|  |  | hsa-miR-421 |  |  |  |
|  |  | hsa-miR-486-5p |  |  |  |

|  | Regression_model |
| --- | --- |
| SimCluster_1 | lg(pK)=-1.9030lg(K)-0.1396 |
| SimCluster_2 | lg(pK)=-1.3288lg(K)-0.3571 |
