# Supplementary material for: A Complex Heterogeneous Network Model of Disease Regulated by Noncoding RNAs: A Case Study of Unstable Angina Pectoris
Source: Comput Intell Neurosci. 2022 Dec 23;2022:5852089. doi: 10.1155/2022/5852089 (PMC9803582; doi:10.1155/2022/5852089)
Supplement: Supplementary Materials — Table S1: Acronym explanation table. Table S2: MTP network. Table S3: Results of network analysis. Table S4: Results of network modelling. Table S5: Case studies. [file 5852089.f1.zip › Results of Network Modelling.docx]

| Model | Link | [Hits@5](mailto:Hits@5) | [Hits@10](mailto:Hits@10) | [Hits@20](mailto:Hits@20) | [Hits@50](mailto:Hits@50) | MR | MRR |
| --- | --- | --- | --- | --- | --- | --- | --- |
| RotatE | MT | 0.1789±0.0167 | 0.2710±0.0284 | 0.4078±0.0275 | 0.6482±0.0163 | 7.9155±0.7216 | 0.1274±0.0134 |
| TransE | MT | 0.0990±0.0162 | 0.1688±0.0154 | 0.2828±0.0189 | 0.5389±0.0303 | 12.7434±1.581 | 0.0795±0.0090 |
| KG2E | MT | 0.0741±0.0095 | 0.1367±0.0169 | 0.2303±0.0176 | 0.4504±0.0226 | 16.6473±1.9285 | 0.0608±0.0071 |
| DistMult | MT | 0.0781±0.0486 | 0.1341±0.0772 | 0.2391±0.1365 | 0.4467±0.2288 | 29.1253±31.6744 | 0.0638±0.0343 |
| RGCN | MT | 0.1408±0.0239 | 0.2313±0.0346 | 0.3778±0.0379 | 0.6251±0.0379 | 9.9069±1.3969 | 0.1031±0.0171 |
| CompGCN | MT | 0.0997±0.0144 | 0.1719±0.0212 | 0.2801±0.0254 | 0.5063±0.0209 | 12.7025±1.3974 | 0.0796±0.0087 |
|  |  |  |  |  |  |  |  |
|  |  |  |  |  |  |  |  |
| RotatE | TT | 0.5600±0.0185 | 0.6925±0.0149 | 0.8203±0.0095 | 0.9403±0.0063 | 3.3667±0.1477 | 0.2976±0.0135 |
| TransE | TT | 0.4368±0.0247 | 0.5977±0.0285 | 0.7596±0.0213 | 0.9248±0.0089 | 8.9800±0.2236 | 0.1402±0.0099 |
| KG2E | TT | 0.3453±0.0243 | 0.5131±0.0286 | 0.7052±0.0249 | 0.9169±0.0132 | 4.2304±0.2909 | 0.2373±0.0154 |
| DistMult | TT | 0.2669±0.1412 | 0.3749±0.1944 | 0.5128±0.2552 | 0.6993±0.3112 | 16.3310±24.3477 | 0.1675±0.0847 |
| RGCN | TT | 0.5030±0.0309 | 0.6644±0.0229 | 0.8102±0.0094 | 0.9444±0.0078 | 3.3050±0.1418 | 0.3031±0.0132 |
| CompGCN | TT | 0.5751±0.0227 | 0.7558±0.0258 | 0.8905±0.0137 | 0.9812±0.0050 | 2.9691±0.1183 | 0.3373±0.0134 |
|  |  |  |  |  |  |  |  |
|  |  |  |  |  |  |  |  |
| RotatE | TP | 0.3220±0.0377 | 0.4712±0.0439 | 0.6353±0.0429 | 0.8533±0.0269 | 4.4050±0.4242 | 0.2288±0.0210 |
| TransE | TP | 0.1710±0.0324 | 0.2876±0.0344 | 0.4666±0.0343 | 0.7742±0.0205 | 7.8392±1.0865 | 0.1297±0.0174 |
| KG2E | TP | 0.0968±0.0302 | 0.1927±0.033 | 0.3392±0.0371 | 0.6215±0.037 | 12.4661±2.2471 | 0.0826±0.0151 |
| DistMult | TP | 0.1190±0.0684 | 0.2058±0.1138 | 0.3251±0.1695 | 0.5627±0.2735 | 24.3245±31.8611 | 0.0941±0.0501 |
| RGCN | TP | 0.3179±0.0346 | 0.4672±0.0377 | 0.6590±0.0372 | 0.8962±0.0231 | 4.4954±0.3793 | 0.2239±0.0189 |
| CompGCN | TP | 0.2720±0.0451 | 0.4050±0.0479 | 0.5722±0.0403 | 0.8065±0.0318 | 5.2599±0.6200 | 0.1926±0.0234 |

| Model | Link | Epoch | Hits@5 | Hits@10 | Hits@20 | Hits@50 | MR | MRR |
| --- | --- | --- | --- | --- | --- | --- | --- | --- |
| RotatE | MT | 25 | 0.1473±0.0295 | 0.2134±0.0392 | 0.2946±0.0556 | 0.4340±0.0632 | 10.3123±1.9440 | 0.1000±0.0177 |
| RotatE | MT | 50 | 0.1789±0.0167 | 0.2710±0.0284 | 0.4078±0.0275 | 0.6482±0.0163 | 7.9155±0.7216 | 0.1274±0.0134 |
| RotatE | MT | 100 | 0.1675±0.0162 | 0.2606±0.0186 | 0.4085±0.0204 | 0.6520±0.0237 | 8.3054±0.8194 | 0.1215±0.0123 |
| RotatE | MT | 200 | 0.1634±0.0173 | 0.2560±0.0249 | 0.3957±0.0235 | 0.6316±0.0222 | 8.155±0.7939 | 0.1236±0.0115 |
|  |  |  |  |  |  |  |  |  |
|  |  |  |  |  |  |  |  |  |
| Model | Link | Embedding | Hits@5 | Hits@10 | Hits@20 | Hits@50 | MR | MRR |
| RotatE | MT | 32 | 0.1587±0.0207 | 0.2513±0.0291 | 0.3691±0.0257 | 0.5914±0.0222 | 9.0563±0.7624 | 0.1111±0.0091 |
| RotatE | MT | 64 | 0.1789±0.0167 | 0.2710±0.0284 | 0.4078±0.0275 | 0.6482±0.0163 | 7.9155±0.7216 | 0.1274±0.0134 |
| RotatE | MT | 96 | 0.1702±0.0203 | 0.2733±0.0223 | 0.4166±0.0243 | 0.6578±0.0186 | 8.1209±0.8165 | 0.1243±0.0131 |
| RotatE | MT | 128 | 0.1747±0.0185 | 0.2758±0.0311 | 0.4208±0.0279 | 0.6691±0.0201 | 7.9137±0.8641 | 0.1276±0.0131 |
|  |  |  |  |  |  |  |  |  |
|  |  |  |  |  |  |  |  |  |
| Model | Link | Learning Rate | Hits@5 | Hits@10 | Hits@20 | Hits@50 | MR | MRR |
| RotatE | MT | 0.0001 | 0.0214±0.0086 | 0.0379±0.0111 | 0.0739±0.0184 | 0.1631±0.0174 | 44.1148±7.67 | 0.0234±0.0046 |
| RotatE | MT | 0.001 | 0.1789±0.0167 | 0.2710±0.0284 | 0.4078±0.0275 | 0.6482±0.0163 | 7.9155±0.7216 | 0.1274±0.0134 |
| RotatE | MT | 0.01 | 0.1437±0.0137 | 0.2215±0.0248 | 0.3451±0.0272 | 0.5839±0.0193 | 9.0002±0.8692 | 0.1120±0.0107 |
| RotatE | MT | 0.1 | 0.1176±0.0172 | 0.1886±0.0206 | 0.3056±0.0329 | 0.5305±0.0237 | 10.4498±1.0269 | 0.0966±0.0101 |

|  |  | RotatE | TransE | KG2E | DistMult | RGCN | CompGCN |
| --- | --- | --- | --- | --- | --- | --- | --- |
| Accuracy | T | 0.7902±0.0115 | 0.7766±0.0188 | 0.7704±0.0235 | 0.8200±0.0201 | 0.8312±0.0220 | 0.8205±0.0248 |
|  | P | 0.8245±0.0400 | 0.8047±0.0630 | 0.8127±0.0459 | 0.8167±0.0634 | 0.8356±0.0404 | 0.8307±0.0336 |
